# Global, regional, and national mortality trends in older children and young adolescents (5–14 years) from 1990 to 2016: an analysis of empirical data

**DOI:** 10.1016/S2214-109X(18)30353-X

**Published:** 2018-09-14

**Authors:** Bruno Masquelier, Lucia Hug, David Sharrow, Danzhen You, Daniel Hogan, Kenneth Hill, Jing Liu, Jon Pedersen, Leontine Alkema, United Nations Inter-agency Group for Child Mortality Estimation

**Affiliations:** a Centre of Demographic Research, Université Catholique de Louvain, Louvain-la-Neuve, Belgium; b Division of Data, Research, and Policy, UNICEF, New York, NY, USA; c Department of Health Statistics and Information Systems, World Health Organization, Geneva, Switzerland; d Johns Hopkins Bloomberg School of Public Health, Baltimore, MD, USA; e Fafo Technical Consulting, Beijing, China; f Fafo Institute for Labour and Social Research, Oslo, Norway; g Department of Biostatistics and Epidemiology, University of Massachusetts Amherst, Amherst, MA, USA

## Abstract

**Background:**

From 1990 to 2016, the mortality of children younger than 5 years decreased by more than half, and there are plentiful data regarding mortality in this age group through which we can track global progress in reducing the under-5 mortality rate. By contrast, little is known on how the mortality risk among older children (5–9 years) and young adolescents (10–14 years) has changed in this time. We aimed to estimate levels and trends in mortality of children aged 5–14 years in 195 countries from 1990 to 2016.

**Methods:**

In this analysis of empirical data, we expanded the United Nations Inter-agency Group for Child Mortality Estimation database containing data on children younger than 5 years with 5530 data points regarding children aged 5–14 years. Mortality rates from 1990 to 2016 were obtained from nationally representative birth histories, data on household deaths reported in population censuses, and nationwide systems of civil registration and vital statistics. These data were used in a Bayesian B-spline bias-reduction model to generate smoothed trends with 90% uncertainty intervals, to determine the probability of a child aged 5 years dying before reaching age 15 years.

**Findings:**

Globally, the probability of a child dying between the ages 5 years and 15 years was 7·5 deaths (90% uncertainty interval 7·2–8·3) per 1000 children in 2016, which was less than a fifth of the risk of dying between birth and age 5 years, which was 41 deaths (39–44) per 1000 children. The mortality risk in children aged 5–14 years decreased by 51% (46–54) between 1990 and 2016, despite not being specifically targeted by health interventions. The annual number of deaths in this age group decreased from 1·7 million (1·7 million–1·8 million) to 1 million (0·9 million–1·1 million) in 1990–2016. In 1990–2000, mortality rates in children aged 5–14 years decreased faster than among children aged 0–4 years. However, since 2000, mortality rates in children younger than 5 years have decreased faster than mortality rates in children aged 5–14 years. The annual rate of reduction in mortality among children younger than 5 years has been 4·0% (3·6–4·3) since 2000, versus 2·7% (2·3–3·0) in children aged 5–14 years. Older children and young adolescents in sub-Saharan Africa are disproportionately more likely to die than those in other regions; 55% (51–58) of deaths of children of this age occur in sub-Saharan Africa, despite having only 21% of the global population of children aged 5–14 years. In 2016, 98% (98–99) of all deaths of children aged 5–14 years occurred in low-income and middle-income countries, and seven countries alone accounted for more than half of the total number of deaths of these children.

**Interpretation:**

Increased efforts are required to accelerate reductions in mortality among older children and to ensure that they benefit from health policies and interventions as much as younger children.

**Funding:**

UN Children's Fund, Bill & Melinda Gates Foundation, United States Agency for International Development.

## Introduction

For decades, the global health community has largely focused on mortality in children younger than 5 years. This attention was reflected in the fourth of the Millennium Development Goals (MDGs), which called for a reduction of two-thirds in the mortality rate of children younger than 5 years between 1990 and 2015. The new Sustainable Development Goals also target mortality of children younger than 5 years and neonates. By contrast, deaths among older children (5–9 years) and young adolescents (10–14 years) have received little attention. This neglect is presumably associated with the fact that this is the age range in which the risk of mortality is lowest.^1^ However, WHO estimated that there were approximately 1 million deaths of older children and young adolescents in 2015, predominantly from avoidable causes. According to WHO, the top five global causes of death in children aged 5–14 years in 2015 were lower respiratory tract infections, diarrhoeal diseases, drowning, meningitis, and road injuries.^2^ This finding indicates that substantial progress could still be achieved with public health interventions covering this age group.

**Research in context**

**Evidence before this study**

We searched PubMed with the terms “child mortality”, “mortality”, “adolescent” for papers published between Jan 1, 1970, and April 1, 2018, in English or French. In most low-income and middle-income countries, civil registration and vital statistics systems are not sufficiently complete to provide timely and accurate child mortality rates. Every year, the United Nations Inter-agency Group for Child Mortality Estimation (UN IGME) does a systematic analysis of mortality of children younger than 5 years, which is based on a comprehensive database that compiles data from surveys, censuses, and vital statistics systems. Thus far, this effort has been limited to mortality in children younger than 5 years, to monitor progress in improving survival of children. Estimates of mortality rates among older children (age 5–9 years) and young adolescents (10–14 years) have previously been derived from model life tables or relational models in countries without complete vital statistics. This approach was taken in the Global Burden of Disease Study (2016) and the World Population Prospects report (2017). In 2015, Kenneth Hill and colleagues provided data-driven estimates of mortality and number of deaths in this age group, but their analysis was limited to 85 countries with Demographic and Health Surveys (and censuses in China), and was based on expected associations between mortality in children younger than 5 years and older children at the regional level.

**Added value of this study**

To our knowledge, this is the first study to provide estimates of mortality in children aged 5–14 years in all countries in the world that were made by use of a comprehensive database of measurements from nationally representative surveys, censuses, and vital registration. We extended the database used by the UN IGME to include older children, and we used a Bayesian penalised B-spline regression model to produce smoothed time series of risks of dying and numbers of deaths, with 90% uncertainty intervals. Our estimates fall between model-based estimates from the Global Burden of Disease Study and the World Population Prospects report. However, in some countries, large deviations were observed from these alternative data sources.

**Implications of all the available evidence**

Although substantial progress has been made in improving the survival of older children since 1990, about 1 million children aged between 5 and 14 years died globally in 2016. This age group is one in which most deaths are preventable. By comparison, there were 2·6 million neonatal deaths, and 3 million deaths of children aged 1–59 months that year. Since 2000, the pace of progress in reducing mortality among older children and young adolescents has been slower than that for children younger than 5 years, which calls for increased attention towards this age group.

To date, little emphasis has been placed on developing robust mortality estimates for children aged 5–14 years. In most low-income and middle-income countries, vital statistics systems that record births and deaths on a continuous basis are not sufficiently complete to generate accurate mortality rates. In the absence of vital statistics data, estimates from the World Population Prospects (WPP) report^3^ and WHO are mainly derived from model life tables, which represent the typical patterns of mortality by age that are observed in countries with accurate data. Similarly, in the 2016 Global Burden of Disease (GBD) Study,^4^ mortality rates among older children and young adolescents were calculated by use of a relational model with two entry parameters: the under-five mortality rate (ie, the probability of a child dying between birth and her fifth birthday, denoted here as _5_q_0_) and the probability of dying between ages 15 years and 60 years (_45_q_15_). Although conceptually similar, these approaches resulted in large differences in mortality estimates. At the global level, the numbers of deaths of children aged 5–14 years for 2010 that were estimated by an earlier GBD study^5^, ^6^ were 40% less than those estimated by a WPP report, despite both being published in 2012. These differences have narrowed in the latest revisions of the GBD study^4^ and WPP report,^3^ but the largest relative difference between age-specific estimates of the two sources is still found in the 5–14 age category (27% in 2010–15, corresponding to an absolute difference of about 330 000 deaths each year between GBD and WPP).

Model life tables or relational models are needed to infer age-specific mortality rates when data are insufficient or inaccurate. However, selection of the model to use is always difficult to justify, and one needs to assume that the historical experience of countries with reliable data adequately reflects age-related patterns of mortality in the populations under consideration. Use of available data for children aged 5–14 years to directly compute mortality rates offers a way to reduce reliance on model life tables. Large-scale household surveys, such as the Demographic and Health Surveys (DHS) and Multiple Indicator Cluster surveys, usually collect full birth histories, in which women of reproductive age provide an exhaustive list of all their children, with information on their sex, date of birth, and survival status. The current age of surviving children is recorded and age at death is collected for deceased children. These full birth histories have become the cornerstone of efforts to track progress in reducing the mortality of children younger than 5 years in low-income and middle-income countries. However, these birth histories have not widely been used to assess the likelihood of survival of children older than 5 years. In 2015, Kenneth Hill and colleagues^5^ estimated the risk of dying between ages 5 years and 15 years by use of 194 DHS surveys and census data from China. Their analysis suggested that mortality in this age range could be higher than expected if extrapolating rates from model life tables for a given level of mortality in children younger than 5 years. Hill and colleagues speculated that this finding could be explained by specific interventions being targeted at children younger than 5 years. However, their estimates were obtained by modelling the association between mortality in children aged 0–4 years and 5–9 years or 10–14 years at the regional level. Linear regression coefficients were used to predict regional estimates of the number of deaths for the years 1990 and 2010. Apart from China, no country-specific estimates were provided.

We aimed to determine global, regional, and national trends in mortality among children aged 5–14 years between 1990 and 2016. Our indicator, denoted _10_q_5_, indicated the probability that a child aged 5 years in a given year would die before reaching age 15 years if they were exposed to the age-specific mortality rates of that year.

## Methods

### Database construction

The United Nations Inter-agency Group for Child Mortality Estimation (UN IGME) is led and coordinated by the United Nations Children's Fund (UNICEF) and also comprises representatives of WHO, the UN Population Division, and the World Bank Group. Since 2004, the UN IGME has compiled a comprehensive database of mortality measurements to estimate under-5 mortality.^7^ In this international cross-sectional study, we expanded this database by adding data on mortality among children aged 5–14 years. These data were sourced from civil registration and vital statistics systems, birth histories collected in sample surveys, data on household deaths reported in censuses, and other sources (all of which are subsequently described), to compile all publicly available nationally representative data on _10_q_5_. Mortality rates were recalculated from the survey microdata, when available, or they were based on published tabulations in census or vital statistics reports. The complete list of data series used in the analyses for each country is shown in the appendix. The UN IGME data portal CMEInfo hosts the full set of empirical data and estimates.

### Vital statistics data

WHO member states regularly provide nationally representative vital statistics data. Through reviews, the UN IGME group establish whether these data are sufficiently complete to estimate child mortality. For our analysis, we retained vital statistics estimates for 87 countries. In some countries, such as South Africa, vital statistics data were included when fitting the trend for _10_q_5_, despite being excluded for UN IGME estimates of _5_q_0_. There are reasons to believe that civil registration systems capture a larger percentage of deaths of children aged 5–14 years than deaths of younger children, which are more likely to be unreported, particularly when they occur in the first few days after birth.^8^ Hence, when WHO estimated that at least 90% of deaths of adults were reported, vital statistics data were also used to estimate _10_q_5_ even if they were excluded for _5_q_0_. Estimates for successive years were pooled together to ensure that the coefficient of variation (the ratio of the standard deviation to the mean) was less than 10%. The stochastic standard error of the observation was calculated with a Poisson approximation, which was based on the population becoming age 5 years in a given year, and these data were estimated from the 2017 WPP report.^3^ Estimates from sample vital registration systems were also used for India, Bangladesh, and Pakistan by use of the same approach. Sample registration systems are systems of data collection, compilation and analysis of vital statistics in a representative sample of the population rather than in the population as a whole.

### Full birth histories

Birth histories were obtained from 272 DHS from 85 countries. We also included estimates from the Pan Arab Project for Child Development and Pan Arab Project for Family Health surveys (17 surveys), Multiple Indicator Cluster surveys (30 surveys), reproductive health surveys (23 surveys), World Fertility surveys (36 surveys), and other country-specific surveys. Surveys that were excluded from the UN IGME estimation process for mortality of children younger than 5 years due to concerns about data quality were also excluded when we estimated mortality among older children.^7^

Estimates of _10_q_5_ obtained from full birth histories might be affected by a truncation bias.^9^ Since full birth histories are typically collected from women aged 15–49 years, the information referring to the 5 years before data collection will refer to women who were aged 10–44 years at that time, only a fraction of whom would have had children aged 5–14 years to report on. Therefore, child mortality estimates of the time before the survey will disproportionately represent high-risk first-born children and children born to younger mothers. This truncation bias, which probably results in overestimation of mortality, might be counterbalanced by more frequent omissions of deaths in the past. Because of the truncation bias, we limited our analysis to a maximum of 12 years before each survey. This limit was set to obtain three data points. We used 4-year periods (instead of 5-year periods) to reduce possible biases due to rounding. Hence, we computed mortality rates from the full birth history data for three reference periods: 0–3 years before the survey, 4–7 years, and 8–11 years. Because of large random errors, we did not use shorter time periods. Standard errors were calculated with a jackknife variance estimation method.^10^

Reports on ever-born and surviving children without their exact ages at death (often called summary birth histories) also contain useful information on child mortality. To estimate the mortality of children younger than 5 years, the UN IGME converts the proportion of children who are deceased out of those ever-born, as reported in surveys or censuses, into mortality indices with indirect demographic methods.^11^ However, there is a need to assess the performance of indirect approaches when applied to older children. Since we do not have the results of such an evaluation, we did not use data on ever-born and surviving children.

### Household deaths

We also included mortality rates that were calculated from reports on household deaths from 34 censuses or large-scale surveys. Tabulations were obtained from the UN Demographic Yearbook^12^ or published census reports (appendix). In China, several censuses were available and estimates were adjusted for the incompleteness of death reporting with the General Growth Balance method (GGB).^13^ The GGB compares the age distribution of reported deaths with the age distribution of the population that was enumerated in the two censuses, to estimate the fraction of deaths that are reported. This method assumes that net migration is negligible (as is the case in China), that reporting of age is accurate, and that the completeness of the population enumeration and reporting of deaths are invariant by age.^14^ In 1990–2000, 90% of male deaths were estimated to have been reported in China, which improved to 98% in 2000–10. 85% of deaths of women were estimated to be reported in 1990–2000, which improved to 92% in 2000–10. Hence, mortality rates are only slightly adjusted upwards in China for the period after 1990, and our results are unlikely to be affected by breaches of the assumptions underpinning the GGB method.

### Statistical analysis

Large-scale sample surveys or census data can be subject to sampling and non-sampling errors. Both types of errors need to be considered when reconstructing mortality trends. To account for these biases, we used the Bayesian B-spline bias-reduction model (referred to as the B3 model), which was developed by Alkema and New,^15^ to calculate country-specific trends from 1990 and short-term projections in mortality until 2016. This model has been used since 2013 by UN IGME to assess trends in mortality of infants and children younger than 5 years.^16^ We applied it to children aged 5 to 14 years.

In the B3 model, observed measurements of _10_q_5_ are modelled on the log-scale as a function of the true risk of dying between ages 5 years and 15 years and an error term, which is specified depending on the type of data source. Time trends in log(_10_q_5_) are modelled with a Bayesian spline regression model. The B3 model includes a data quality model to adjust for biases in data series. For example, mean biases within surveys are modelled as a linear function of the retrospective time of the observation. The data quality model also adjusts for differences in sampling and non-sampling variance across observations. Validation results indicate that the B3 model performs reasonably well for estimating mortality in children aged 5–14 years (appendix).

In 37 countries, adjustments were made to account for abrupt rises in mortality due to conflicts or disasters, which would otherwise not be apparent in the smoothed mortality curves generated by the B3 model and might not be captured in the birth history data because of an increased correlation between the likelihood of survival of a mother and her child in these contexts. Adjustments were based on estimates of crisis-related deaths developed by WHO from 1990 to 2010. Various data sources were used by WHO for these estimates, including EM-DAT, the International Disaster Database maintained by the Centre for Research on the Epidemiology of Disasters, the Uppsala Conflict Data Program dataset, and reports prepared by the UN and other organisations.^17^ From these data on crisis-related deaths, we estimated excess mortality rates. We excluded the input data referring to years affected by crises from our database, fitted the B3 model to the remaining data, then added back the excess mortality rates to the smoothed trend. The full list of crises explicitly incorporated in the estimates is shown in the appendix. Although this adjustment is of great importance in some countries heavily affected by crises, the number of excess deaths added through the crisis adjustment corresponded to less than 1% of the total deaths in the 5–14 years age group since 1990. This percentage does not include all crisis-related deaths, however, because we did not adjust for crises that were spread over several years and resulted in stalls in the mortality decline, which was already well captured by the B3 model.

Unlike the processes for estimation of mortality of children younger than 5 years, we did not account for HIV-related survivor biases in full birth histories.^18^ Because of the vertical transmission of the virus, child mortality estimates obtained from mothers' reports are biased downwards, since seropositive mothers are less likely to survive to the survey and report on the increased mortality of their children. However, no method to estimate the effects of this bias on _10_q_5_ exists. There is likely to be less bias when estimating mortality in children aged 5–14 years than mortality in children younger than 5 years because, in the absence of treatment, most children who are vertically infected will die before reaching age 5 years.^19^

For 39 countries, corresponding to 4·5% of the global population of children aged 5–14 years in 2016, mortality trends could not be estimated from the aforementioned sources with the B3 model because they had insufficient data. For these countries, _10_q_5_ was modelled by use of the association between mortality of children aged 0–4 years and 5–14 years, as observed in the 156 countries for which the B3 model was used. A linear model was used to regress log(_10_q_5_) against log(_5_q_0_), with region-specific dummies. _10_q_5_ between 1990 and 2016 was predicted for countries with inadequate mortality measurements by use of the country-specific _5_q_10_ estimates and the coefficients of this regression (appendix).

To calculate the number of deaths of children aged 5–14 years, we accounted for the age structure of the population aged 5–14 in each country. _10_q_5_ was split into two segments: children aged 5–9 years (_5_q_5_), and those aged 10–14 years (_5_q_10_). A specific database of mortality measurements in children aged 5–9 years was constructed from the same data sources, but only to calculate mortality rates of children aged 5–9 years. A penalised spline regression model that was similar to the B3 model was used to estimate country-specific trends in the logit transformation of r—ie, (log(r/(1 – r)))—where r is the ratio of the probability _5_q_5_ to the median B3 estimate of _10_q_5_. This approach ensures that the _5_q_5_ is constrained to be lower than _10_q_5_. When the database did not include enough data series of _5_q_5_, we used region-specific coefficients to relate _5_q_0_ and _5_q_5_. When _10_q_5_ (for children aged 5–14 years) and _5_q_5_ (for children aged 5–9 years) were estimated, we derived _5_q_10_ (for children aged 10–14 years). The age-specific probabilities were then converted into mortality rates,^20^ and multiplied by the population estimates from the WPP report.^3^ The estimated numbers of deaths are presented with 90% uncertainty intervals, which were obtained by sampling from the posterior distribution of the parameters of the Bayesian model. Our uncertainty intervals do not reflect uncertainties around population counts because these uncertainties are not available in the WPP estimates.

A joint WHO/UNICEF country consultation was done in 2017 to give each country's ministry of health, national statistics office, or other responsible government agency the opportunity to review the data inputs, the methods, and the draft estimates and to provide additional data.

### Role of the funding source

The funders of the study had no role in the study design, data collection, data analysis, data interpretation, or writing of the report. The corresponding author had full access to all the data in the study and had final responsibility for the decision to submit for publication.

## Results

**Figure 1:**
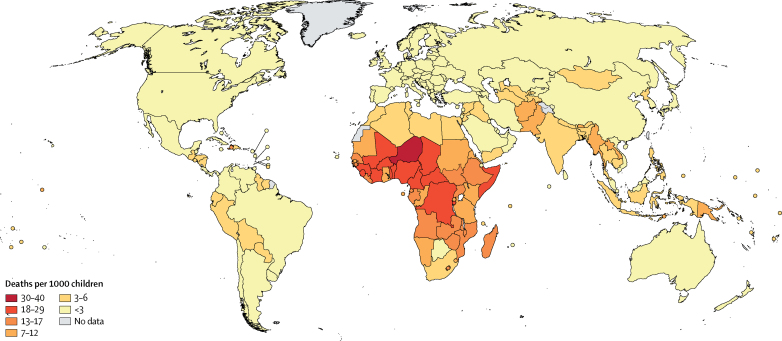
Probability of dying among children aged 5–14 years in 2016

Globally, the probability of a child aged 5 years dying between the ages of 5 years and 15 years (_10_q_5_) was 7·5 (90% uncertainty interval 7·2–8·3) deaths per 1000 children in 2016. This probability varied widely between countries, ranging from 0·5 deaths per 1000 children in Luxembourg (0·3–0·7) to 40 deaths per 1000 children in Niger (28–58; figure 1). Sub-Saharan Africa had the highest burden of mortality in older children and young adolescents: the top 26 countries with the highest mortality rates in this age group were all located in this region. The average _10_q_5_ for a child in sub-Saharan Africa (18·4 [17·0–21·0]) was 17 times higher than that for children in high-income countries (1·1 [1·0–1·1]).

The risk of mortality of children aged 5–14 years has substantially decreased since 1990, despite this age range not being specifically targeted by health interventions. Globally, the _10_q_5_ decreased from 15·2 (90% uncertainty interval 14·9–15·9) deaths per 1000 children in 1990 to 7·5 deaths (7·2–8·3) per 1000 children in 2016, a decrease of 51% (46–54; table 1). In 129 of the 195 countries analysed, the mortality in children aged 5–14 years was reduced by more than half (based on point estimates) between 1990 and 2016. 32 countries, including Bangladesh, Cambodia, Nepal, Vietnam, and Ethiopia, reduced the mortality rate of children aged 5–14 years by two-thirds between 1990 and 2016 (appendix).

**Table 1:** Global and regional probabilities of death among children aged 5–10 years, 10–15 years, and 5–15 years

		**Probability _5_q_5_**			**Probability _5_q_10_**			**Probability _10_q_5_**			**Annual rate of reduction in _10_q_5_, %**
		1990	2000	2016	1990	2000	2016	1990	2000	2016	1990–2016
Sub-Saharan Africa		30·3 (28·8–32·0)	22·9 (22·1–23·9)	11·9 (11·0–12·8)	11·7 (8·9–15·4)	9·7 (8·4–11·4)	6·6 (5·0–9·3)	41·6 (39·4–45·0)	32·4 (31·4–33·8)	18·4 (17·0–21·0)	3·1% (2·6–3·6)
	Eastern and southern Africa	29·5 (27·4–32)	21·0 (20·0–22·2)	9·1 (8·2–10·0)	12·0 (8·4–16·1)	9·1 (7·5–10·9)	4·7 (3·0–7·3)	41·1 (38·4–44·6)	29·9 (28·6–31·6)	13·8 (12·2–16·2)	4·2% (3·5–4·8)
	West and central Africa	31·2 (28·9–33·5)	25·0 (23·7–26·5)	14·7 (13·2–16·4)	11·3 (6·8–17·8)	10·6 (8·4–13·1)	8·7 (5·5–13·2)	42·1 (38·3–47·8)	35·3 (33·7–37·5)	23·3 (20·5–27·6)	2·3% (1·5–3·0)
Middle East and North Africa		5·8 (5·5–6·3)	3·7 (3·5–3·9)	2·2 (2·0–2·5)	3·7 (3·2–4·5)	2·4 (2·2–2·8)	1·8 (1·4–2·4)	9·5 (8·9–10·5)	6·1 (5·8–6·5)	4·0 (3·7–4·7)	3·3% (2·7–3·8)
South Asia		13·3 (13·0–13·8)	8·5 (8·3–8·8)	3·7 (3·4–4·0)	7·4 (6·4–8·2)	5·4 (4·8–6·0)	3·1 (2·3–4·4)	20·6 (19·8–21·4)	13·9 (13·4–14·5)	6·8 (6·1–8·1)	4·3% (3·6–4·7)
East Asia and Pacific		5·9 (5·4–6·5)	3·7 (3·4–4·0)	1·9 (1·6–2·2)	3·1 (2·2–4·0)	2·5 (2·0–3·1)	1·7 (1·2–2·7)	9·0 (8·3–9·8)	6·2 (5·8–6·7)	3·6 (3·0–4·6)	3·5% (2·5–4·3)
Latin America and Caribbean		3·5 (3·4–3·6)	2·3 (2·2–2·4)	1·4 (1·3–1·5)	2·7 (2·4–3·2)	2·1 (2·0–2·3)	1·6 (1·5–1·9)	6·2 (5·8–6·7)	4·4 (4·3–4·6)	3·0 (2·9–3·3)	2·7% (2·3–3·1)
North America		1·1 (1·1–1·1)	0·8 (0·8–0·8)	0·6 (0·5–0·6)	1·3 (1·3–1·3)	1·0 (1·0–1·1)	0·6 (0·6–0·7)	2·4 (2·4–2·4)	1·8 (1·8–1·8)	1·2 (1·2–1·3)	2·5% (2·3–2·7)
Europe and central Asia		2·5 (2·4–3·1)	1·8 (1·7–2·0)	0·8 (0·8–0·9)	1·8 (1·6–2·5)	1·5 (1·4–1·7)	0·9 (0·8–0·9)	4·3 (4·1–5·6)	3·3 (3·2–3·5)	1·7 (1·6–1·8)	3·7% (3·3–4·7)
	Western Europe	1·1 (1·1–1·1)	0·7 (0·7–0·7)	0·4 (0·4–0·4)	1·1 (1·1–1·1)	0·8 (0·8–0·8)	0·4 (0·4–0·5)	2·2 (2·2–2·2)	1·5 (1·5–1·5)	0·8 (0·8–0·8)	3·8% (3·7–3·9)
	Eastern Europe and central Asia	3·6 (3·4–4·8)	2·8 (2·6–3·1)	1·2 (1·2–1·4)	2·5 (2·0–3·8)	2·1 (1·9–2·4)	1·3 (1·2–1·3)	6·1 (5·7–8·5)	4·9 (4·7–5·2)	2·5 (2·4–2·6)	3·5% (3·0–4·8)
World		10·3 (10·1–10·7)	7·7 (7·5–7·9)	4·5 (4·3–4·7)	5·0 (4·5–5·6)	4·0 (3·8–4·4)	3·0 (2·6–3·8)	15·2 (14·9–15·9)	11·7 (11·4–12)	7·5 (7·2–8·3)	2·7% (2·3–3·0)

5q_5_ is the probability of a child alive at age 5 years dying between ages 5 years and 10 years, _5_q_10_ is the probability between ages 10 and 15 years, and _10_q_5_ is the probability between ages 5 years and 15 years, all expressed per 1000 children in 1990, 2000, and 2016, with 90% uncertainty intervals. The appendix shows the definitions of the regional classifications.

Worldwide, almost 1 million (0·9 million–1·1 million) children aged 5–14 years died in 2016 (table 2). Most deaths occurred in west and central Africa (321 000 [90% uncertainty interval 286 000–377 000] deaths), followed by south Asia (238 000 [212 000–283 000] deaths), eastern and southern Africa (201 000 [179 000–234 000] deaths), and east Asia and Pacific (109 000 [92 000–138 000] deaths). Other regions accounted for less than 10% of global deaths. Overall, 98% (98–99) of all deaths of children aged 5–14 years occurred in low-income and middle-income countries, which comprise 89·5% of the global population of children of this age group. Seven countries accounted for 52% of all deaths in this age group (India, Nigeria, the Democratic Republic of the Congo, Ethiopia, Pakistan, China, and Niger).

**Table 2:** Global and regional numbers of deaths among children aged 5–9 years, 10–14 years, and 5–14 years

		**Deaths among children aged 5–9 years (thousands)**			**Deaths among children aged 10–14 years (thousands)**			**Deaths among children aged 5–14 years (thousands)**			**Share of global deaths in children aged 5–14 years, %**
		1990	2000	2016	1990	2000	2016	1990	2000	2016	1990–2016
Sub-Saharan Africa		470 (446–496)	448 (432–467)	351 (325–379)	151 (115–201)	166 (143–193)	171 (128–240)	621 (592–667)	613 (596–638)	522 (483–590)	54·5% (50·9–57·8)
	Eastern and southern Africa	245 (227–266)	218 (208–231)	137 (123–150)	83 (59–112)	82 (68–99)	64 (40–98)	328 (308–354)	300 (288–316)	201 (179–234)	20·9% (18·4–23·6)
	West and central Africa	225 (208–242)	230 (218–244)	214 (192–238)	68 (41–108)	83 (66–103)	107 (68–163)	293 (269–329)	313 (300–331)	321 (286–377)	33·5% (30–37·1)
Middle East and North Africa		43 (41–47)	29 (27–30)	19 (18–22)	23 (21–29)	19 (17–22)	14 (11–19)	67 (63–73)	48 (45–51)	33 (31–38)	3·5% (3·1–4·0)
South Asia		396 (386–410)	282 (275–291)	131 (121–139)	192 (166–214)	172 (153–191)	107 (81–153)	587 (566–610)	454 (437–472)	238 (212–283)	24·6% (21·9–28·2)
East Asia and Pacific		207 (192–230)	129 (119–139)	57 (49–67)	10 (73–135)	101 (82–124)	52 (35–79)	312 (288–339)	230 (213–250)	109 (92–138)	11·3% (9·5–13·8)
Latin America and Caribbean		37 (36–39)	26 (25–27)	15 (14–16)	28 (24–33)	24 (22–25)	18 (16–21)	65 (61–70)	49 (48–51)	33 (31–36)	3·4% (3·1–3·7)
North America		4 (4–4)	4 (4–4)	3 (3–3)	5 (5–5)	5 (5–5)	3 (3–3)	9 (9–10)	8 (8–8)	6 (5–6)	0·6% (0·5–0·6)
Europe and central Asia		32 (30–40)	20 (19–22)	9 (9–10)	23 (20–32)	20 (18–21)	9 (8–9)	55 (52–72)	40 (38–42)	18 (17–19)	1·8% (1·7–2·0)
	Western Europe	6 (6–6)	4 (4–4)	2 (2–2)	7 (6–7)	5 (4–5)	2 (2–2)	13 (13–13)	8 (8–8)	4 (4–4)	0·4% (0·4–0·5)
	Eastern Europe and central Asia	26 (24–34)	17 (16–18)	7 (7–8)	17 (14–26)	15 (13–17)	6 (6–7)	42 (39–59)	32 (30–34)	13 (13–14)	1·4% (1·3–1·5)
World		1189 (1162–1232)	936 (918–961)	585 (555–616)	527 (475–593)	506 (471–548)	373 (326–469)	1716 (1677–1784)	1443 (1415–1482)	958 (914–1052)	100%

Data are thousands of deaths (90% uncertainty intervals), unless otherwise indicated. The appendix shows the definitions of the regional classifications.

**Figure 2:**
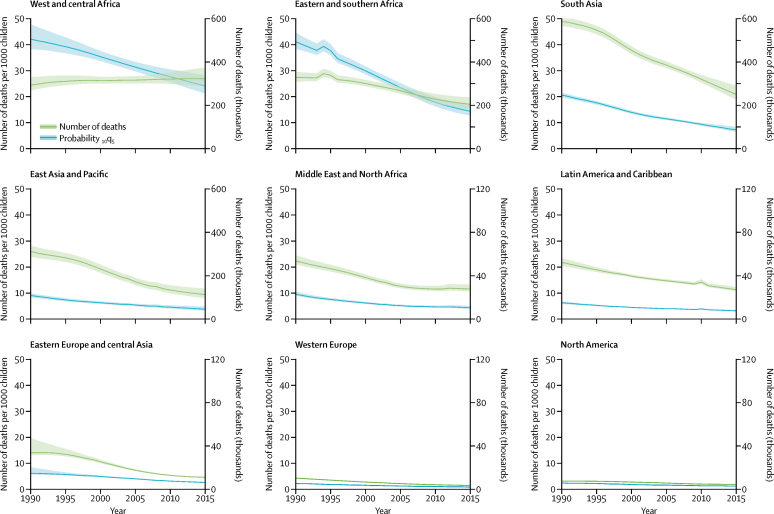
Regional trends in the probability of a child aged 5 years dying between ages 5 years and 15 years and the numbers of deaths in children aged 5–14 years, from 1990 to 2016 Data are number of deaths (thousands) and probability (_10_q_5_) of dying per 1000 children, with 90% uncertainty intervals.

The number of deaths decreased from 1·7 million (1·7 million–1·8 million) in 1990 to 1·0 million (0·9 million–1·1 million) in 2016 (figure 2). All regions showed a decrease in deaths among children aged 5–14 years, except west and central Africa, where mortality rates decreased but the absolute number of deaths has increased since 1990 because of rapid population growth. From 1990 to 2016, the number of children aged 5–14 years doubled in this region.^3^ Peaks in the number of deaths in eastern and southern Africa and in Latin America and Caribbean reflect the crises of the Rwandan genocide and the 2010 earthquake in Haiti and the resultant increases in mortality.

**Figure 3:**
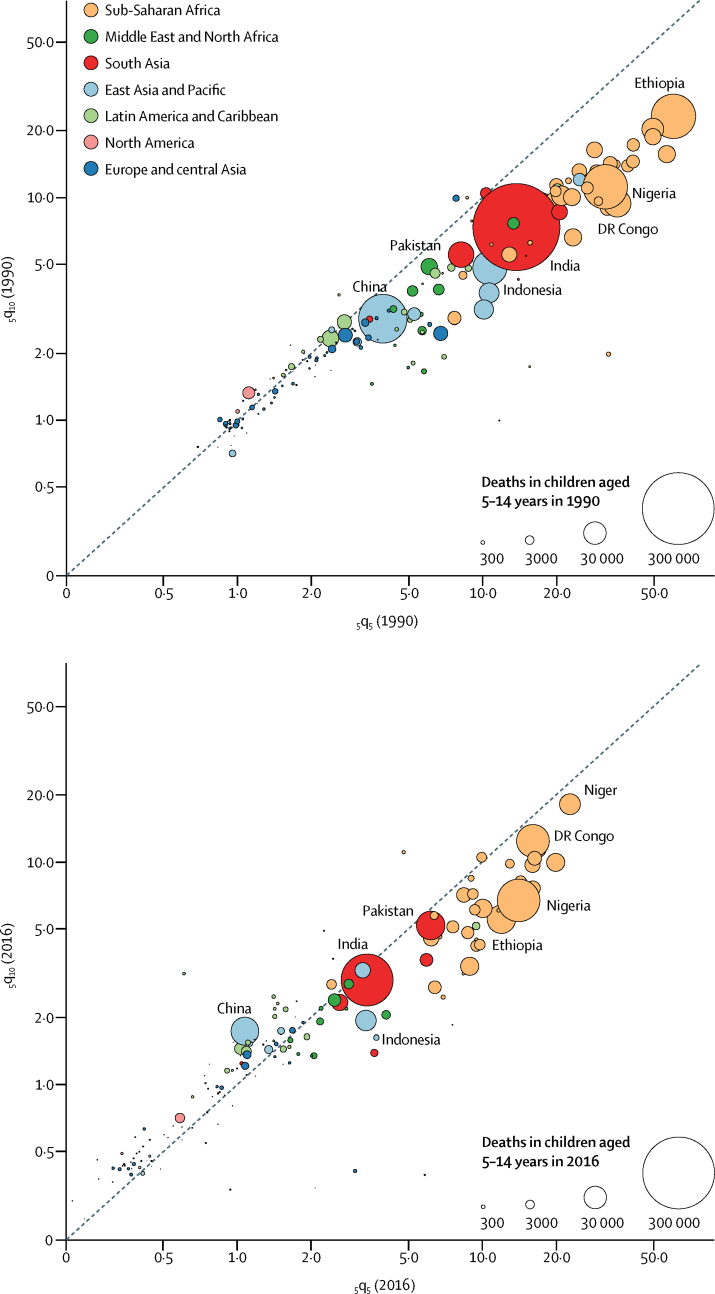
Probability of a child aged 5 years dying between ages 5 years and 10 years or 10 years and 15 years in 1990 and 2016 _5_q_5_ is the probability of dying between ages 5 years and 10 years, and _5_q_10_ is the probability of dying between ages 10 years and 15 years. Both are expressed per 1000 children alive, stratified by country. Each bubble represents a country. The size of bubbles represents the number of deaths in each year. Countries for which the B3 model was not used are not shown.

Deaths among children aged 5–9 years accounted for 60% (90% uncertainty interval 55–65) of all deaths of children between the ages of 5 years and 15 years, which is slightly higher than the proportion of this population who are aged 5–9 years (51%). This discrepancy is because mortality is generally higher in children aged 5–9 years than those aged 10–14 years, except in countries with low mortality. In 2016, the _5_q_10_ globally was 3·0 (2·6–3·8) deaths per 1000 children aged 5 years, corresponding to two-thirds of the value of _5_q_5_, which was 4·5 (4·3–4·7) deaths per 1000 children aged 5 years. There were noticeable regional differences in this ratio of _5_q_10_ to _5_q_5_. Sub-Saharan Africa had the lowest ratio of _5_q_10_ to _5_q_5_ in 2016 (58·8% [based on unadjusted data]; 40·4–80·8), and the highest ratio was observed in Latin America and the Caribbean (121% [based on unadjusted data]; 99·6–138·1). The _5_q_10_ to _5_q_5_ ratio increased as mortality decreased: from 1990 to 2016, this ratio increased from 48·5 (42·9–55·2) to 66·7 (57·5–86·0; figure 3). This increase is a consequence of a more rapid mortality decline in children aged 5–9 years than in young adolescents aged 10–14 years. Over 1990–2016, the annual rate of reduction (ARR) in children aged 5–9 years was 3·2% (3·0–3·4) versus 1·9% (0·9–2·6) in young adolescents. In 1990, the risk of dying (based on point estimates) among young adolescents (_5_q_10_) was higher than among children aged 5–9 years (_5_q_5_) in 38 countries (appendix); in 2016, this difference in mortality was present in 91 countries.

**Figure 4:**
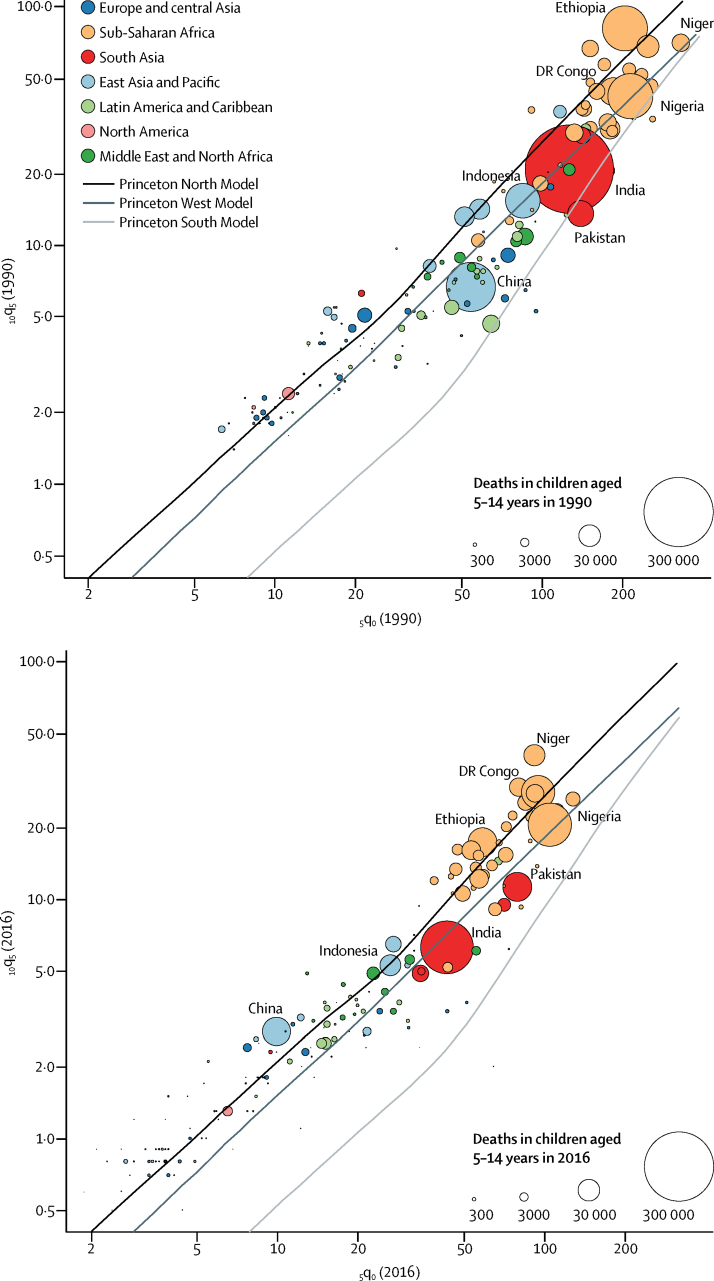
Probability of a child dying younger than 5 years and of a child aged 5 years dying between ages 5 years and 15 years in 1990 and 2016 _5_q_0_ is the probability of dying between ages 0 years and 5 years, and _10_q_5_ is the probability of dying between ages 5 years and 15 years. Both are expressed per 1000 children, stratified by country. Each bubble represents a country. The size of bubbles represents the number of deaths in each year. Countries for which the B3 model was not used are not shown. The solid lines represent the _10_q_5_ to _5_q_10_ ratio expected from three different model life tables from the Coale-Demeny system.^21^

Children aged 5–14 years have a higher likelihood of survival than children younger than 5 years. The global _10_q_5_ in 2016 was 18% of the mortality rate of children younger than 5 years, even though the length of time is twice as long (ie, between the fifth and the 15th birthday). There was a large variability in the _10_q_5_ to _5_q_0_ ratio nationally (figure 4). Our data-driven estimates were broadly contained between the North and the South pattern.^21^ However, inference of _10_q_5_ from _5_q_0_ values and these different model life tables would result in misleading estimates. Even the selection of one specific model life table among the different models available is difficult to justify. Overall, countries in sub-Saharan Africa, east Asia, and the Pacific region show higher _10_q_5_ values at a given _5_q_0_ than other regions. By contrast, the ratio of _10_q_5_ to _5_q_0_ tends to be lower in Europe, central Asia, and North America. There are also important variations in the _5_q_0_ to _10_q_5_ ratio, even within some regions, as shown in Ethiopia and Nigeria. Additionally, when comparing data from 1990 and 2016, we note that the _10_q_5_ to _5_q_0_ ratio is changing. In 1990, most estimates of _10_q_5_ were less than what would be predicted from the North model of Coale-Demeny life tables^21^ when indexed on _5_q_0_. In 2016, more estimates were greater than the _10_q_5_ that was expected based on the North pattern. This convergence towards a North pattern is a natural consequence of more rapid decreases in _5_q_0_ than in the _10_q_5_ during 1990–2016.

**Figure 5:**
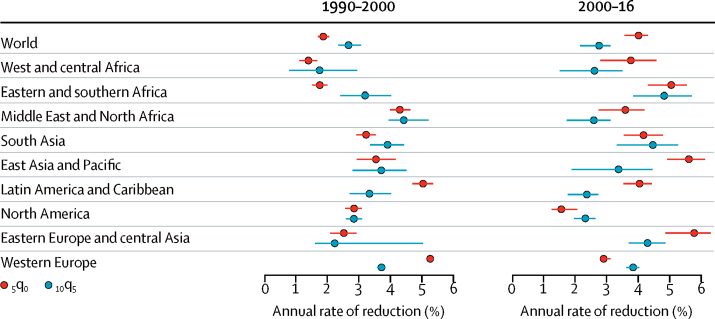
Comparison of the annual rate of reduction in _5_q_0_ and _10_q_5_ in 1990–2000 and 2000–16, by region _5_q_0_ is the probability of dying before age 5 years, and _10_q_5_ is the probability of dying between ages 5 years and 15 years.

Over 1990–2016, the _5_q_0_ decreased more rapidly than the _10_q_5_. Between 1990 and 2016, the global ARR in _10_q_5_ was 2·7% (90% uncertainty interval 2·3–3·0), compared with the ARR in _5_q_0_, which was 3·2% (2·9–3·4). However, notable temporal patterns emerge when contrasting the mortality declines in _5_q_0_ with those of _10_q_5_ in 1990–2000 and in 2000–16. In 1990–2000, mortality decreased faster among older children and young adolescents than _5_q_0_ did: five of nine regions showed higher ARRs in children aged 5–14 years than in children younger than 5 years (figure 5). From 2000 to 2016, the decrease in _5_q_0_ accelerated and exceeded the rate of decline of _10_q_5_. Although the ARR in _5_q_0_ more than doubled from 1990–2000 to 2000–16 (from 1·9% to 4·0%), the change in ARR among older children and young adolescents was not substantial at the global level (from 2·7% [2·4–3·1] to 2·8% [2·2–3·1]). The increase in the _10_q_5_ ARR was greater than 1% in two regions: eastern and southern Africa and eastern Europe and central Asia. In the other regions, there was either a very modest acceleration or a slower decline in ARR.

Table 3 compares the global and regional numbers of deaths from the 2016 GBD Study,^4^ the WPP report^3^ from 2017, and our study. Instead of using the UNICEF regional classification, we used the GBD regional classification for this comparison so that GBD estimates can be presented alongside their publicly available uncertainty intervals. Estimates of deaths by age in the WPP are not provided with uncertainty intervals and are available by 5-year periods. We calculated the annual numbers of deaths by use of Beers' six-term modified formula^20^ and aggregated them by GBD region. Our global estimates fall between the numbers of deaths from the GBD 2016 and the 2017 WPP. Our estimate of mortality of children aged 5–14 years for 2016 was 958 000, which is 182 000 (19%) more deaths than the GBD 2016 estimate of 776 000 deaths, and 201 000 (21%) deaths fewer than the WPP 2017 estimate of 1 159 000 deaths. Beyond sub-Saharan Africa, North Africa and the Middle East, and South Asia, the differences between our regional estimates and the GBD or WPP estimates were fewer than 15 000 deaths in 2016. The main discrepancies between estimates were concentrated in sub-Saharan Africa in both 1990 and 2016, where we found a greater number of deaths than the GBD estimates, but fewer than those estimated in the WPP. Our estimates of the number of deaths of children aged 5–14 years in sub-Saharan Africa were about 190 000 deaths more than the GBD estimates for 2016, and 150 000 deaths fewer than WPP estimates.

**Table 3:** Numbers of deaths of children aged 5–14 years (in thousands) in Global Burden of Disease Study regions

	**Global Burden of Disease Study,**^4^**2016**		**World Population Prospects,**^3^**2017**		**This study**	
	1990	2016	1990	2016	1990	2016
High-income North America	9·2 (9·2–9·3)	5·7 (5·7–5·8)	10·0	5·4	9·4 (9·3–9·6)	5·7 (5·4–6)
Australasia	0·6 (0·6–0·6)	0·3 (0·3–0·3)	0·6	0·3	0·6 (0·6–0·6)	0·3 (0·3–0·4)
High-income Asia-Pacific	5·6 (5·1–6·2)	1·4 (1·3–1·4)	6·7	1·5	6·9 (6·8–7)	1·3 (1·3–1·4)
Western Europe	9·7 (9·6–9·7)	3·8 (3·7–4)	9·6	3·5	9·5 (9·4–9·6)	3·5 (3·4–3·7)
Southern Latin America	3·4 (3·4–3·5)	2·0 (2·0–2·1)	3·7	2·2	3·2 (3·2–3·3)	2·0 (1·9–2·0)
Eastern Europe	17·0 (16·4–17·6)	5·2 (4·8–5·6)	15·7	5·2	16·5 (16·2–16·7)	4·9 (4·6–5·2)
Central Europe	6·8 (6·7–6·9)	1·6 (1·5–1·8)	6·5	1·6	6·8 (6·7–6·9)	1·5 (1·4–1·5)
Central Asia	11·2 (10·7–11·6)	6·6 (5·8–7·2)	11·6	5·2	11·7 (9·6–27·8)	5·0 (4·6–5·9)
Central Latin America	32·2 (31·7–32·8)	15·4 (14·8–15·9)	28·8	17·4	25·4 (22·7–28·8)	13·1 (12·1–14·5)
Andean Latin America	10·1 (9·8–10·4)	4·7 (4·3–5·2)	12·8	7·8	10·4 (9·6–11·2)	4·6 (3·6–6·5)
Caribbean	6·6 (6·2–7·0)	4·5 (3·9–5·1)	8·5	6·9	8·9 (8·1–9·9)	4·8 (3·7–6·4)
Tropical Latin America	20·9 (20·4–21·3)	10·1 (9·7–10·6)	21·8	10·6	17·3 (14·7–20·4)	8·2 (7·8–8·8)
East Asia	204·2 (200·7–208·1)	54·9 (53·9–56·1)	150·3	42·0	141·5 (118·4–165·5)	46·7 (31·9–70·1)
Southeast Asia	128·1 (125·1–131·3)	53·0 (50·5–55·6)	134·6	62·0	161·9 (153·1–173·5)	59·2 (50·5–73·9)
Oceania	1·8 (1·6–2·1)	2·0 (1·6–2·6)	2·5	1·9	2·2 (1·8–2·7)	1·9 (1·6–2·4)
North Africa and Middle East	100·3 (92·2–108·2)	71·4 (63·9–78·6)	133·5	74·3	103·4 (97·8–112·6)	55·5 (50·8–64·2)
South Asia	441·9 (436·3–447·7)	210·1 (203·8–216·5)	574·2	247·0	577·4 (556·1–599·4)	227·3 (201·5–271·7)
Southern sub-Saharan Africa	13·5 (13·1–14·0)	23·8 (22·5–25·2)	16·4	18·3	16·3 (14·0–19·6)	14·1 (12·1–16·6)
Western sub-Saharan Africa	120·4 (117·1–123·9)	147·5 (139·5–156·5)	260·8	368·8	242·5 (226·0–262·5)	251·9 (220·6–298·8)
Eastern sub-Saharan Africa	115·0 (112·7–117·1)	113·6 (109–117·9)	273·4	189·7	277·1 (258·0–301·6)	163·8 (141·2–195·8)
Central sub-Saharan Africa	29·5 (27·4–31·7)	38·4 (32·3–45·1)	83·8	87·3	67·5 (50·7–97·8)	82·7 (63·7–111)
World	1288·5 (1276·1–1301)	776·2 (761·8–792)	1765·5	1158·9	1716·5 (1677·3–1784·4)	957·8 (914·0–1052·4)

Data are thousands of deaths (90% uncertainty intervals).

At the country level, we note substantial differences between these three sources. Our estimates of _10_q_5_ for 2016 were only half the WPP estimates in 12 countries and double those of the GBD 2016 in eight countries (all located in sub-Saharan Africa). Such country-specific differences between the UN IGME, WPP, and GBD estimates necessitate concerted efforts to improve the collection and analysis of data on deaths in this age group.

## Discussion

Globally, 1 million (90% uncertainty interval 0·9 million–1·1 million) children aged 5–14 years died in 2016. These deaths were disproportionately concentrated in sub-Saharan Africa and South Asia. 55% (51–58) of deaths in this age group were in sub-Saharan Africa, an increase from 36% (35–38) in 1990. 25% (22–28) of these deaths occurred in South Asia, including 160 000 (142 000–178 000) deaths in India in that year.

Information on the underlying diseases that contribute most directly to these deaths is required to guide health programmes and interventions. Unfortunately, there is an extreme dearth of such data in this age group, partly because 98% (90% uncertainty interval 98–99) of deaths in children aged 5–14 years occur in low-income and middle-income countries, where civil registration systems are often rudimentary. Additionally, efforts to quantify mortality that is attributable to the leading causes of death have largely focused on children younger than 5 years or people aged 15 years and above, with less attention devoted to children aged 5–14 years. Evidence indicates that most of these deaths are preventable. In India, a nationally representative survey indicated that, in children aged 5 to 14 years, infectious diseases caused 58% of all deaths between 2001 and 2003 (including 18% of deaths due to diarrhoeal diseases and 10% due to pneumonia) and injuries caused 22% of deaths.^22^ In another study^23^ based on verbal autopsies in Health and Demographic Surveillance Sites, malaria and acute respiratory infections (including pneumonia) caused a large proportion of deaths in children aged 5–14 years; more than a quarter of deaths were due to these two causes at seven sites in sub-Saharan Africa and southeast Asia. Model-based estimates also confirm the increased burden of infectious diseases in this age group relative to adults and the elderly. According to WHO's Global Health Estimates,^2^ lower respiratory infections caused 11% of deaths of children aged 5–14 years globally, other communicable diseases caused 36% of such deaths, and injuries accounted for 27% of such deaths in 2015. Cost-effective and life-saving interventions are available for most infectious diseases. Scaling up these interventions in children aged 5–14 years is required to accelerate decreases in mortality. Addressing the growing burden of injuries as children get older is also needed to secure sustained mortality improvements. According to Bundy and colleagues,^24^ a package of health services can be effectively delivered through the school system (including, among others, deworming, insecticide-treated net promotion, school meals programmes, and healthy lifestyle education) because most children aged 5–14 years attend school.

Substantial progress has already been made in reducing mortality of children aged 5–14 years since 1990. Globally, the risk of dying in this age range was reduced by 51% (46–54) between 1990 and 2016. However, children aged 5–14 years did not show a similar rate of reduction in mortality rates to that observed in children younger than 5 years after the introduction of the MDGs in 2000. The ARR did not significantly increase, changing from 2·7% (2·4–3·1) in 1990–2000 to 2·8% (2·2–3·1) in 2000–16. This finding suggests that efforts to accelerate improvements in child survival in the context of the MDGs might not have benefited older children and young adolescents as much as they helped children younger than 5 years. We also found that mortality in young adolescents aged 10–14 decreased less than mortality in children aged 5–9 years. As posited by Hill and colleagues,^5^ public health interventions that were delivered to improve health in infants and young children might have provided benefits to the 5–9 years age group, with smaller spillover effects in adolescents aged 10–14 years. It is also possible that this slower decline in mortality in young adolescents is due to a higher burden of non-communicable diseases and external causes of death (such as road injuries, drowing, and falls), which are often harder to prevent than communicable diseases. The increased ratios of _5_q_10_ to _5_q_5_ in low-mortality relative to high-mortality countries support this hypothesis.

Our results were between model-based estimates from the 2016 GBD Study^4^ and the 2017 WPP report.^3^ Our global estimate of the number of deaths of children aged 5–14 years in 2016 is 19% higher than the GBD estimates and 21% lower than the WPP estimate. Our uncertainty intervals around the number of deaths for 2016 do not contain the WPP estimate and do not overlap with the GBD uncertainty intervals. Discrepancies in mortality rates in sub-Saharan Africa account for most of these differences. The use of standard age patterns of mortality in the case of the WPP could overestimate risks of dying among older children in this region. It is also possible that, because we did not adjust for HIV selection biases, we underestimated mortality in African countries severely affected by HIV. The lower estimates obtained in the GBD study for sub-Saharan Africa relative to our estimates with the B3 model could be linked to low estimates of the probability of dying in adulthood (_45_q_15_), which are used as an entry parameter in the GBD relational models, and the choice of the reference set of empirical life tables used as standard for the mortality age pattern.^25^ Nonetheless, these deviations highlight the need to deviate from model life tables or relational models and to develop data-driven estimates which can better capture local peculiarities.

Our results also differ from previous work by Hill and colleagues,^5^ which estimated the number of deaths of children aged 5–14 years in low-income and middle-income countries at 2·4 million (90% uncertainty interval 1·9 million–2·7 million) in 1990 and 1·5 million (1·2 million–1·8 million) in 2010. According to our results, there were 1·7 million deaths in 1990 (1·6 million–1·7 million) and 1·1 million in 2010 (1·1–1·1). We can ascribe these differences to the use of regional coefficients by Hill and colleagues and to potential differences in estimating life table probabilities of dying from full birth histories. Overall, discrepancies across series of estimates are likely to decline in the coming years as methods improve and databases are expanded.

An important limitation of our analysis was that estimates are still derived from an expected association of _10_q_5_ with _5_q_0_ for 39 countries. Although we did not use conventional model life tables, these estimates are still inferred from the data of countries with good vital registration data or numerous sample surveys, and they might mask distinctive features of countries without sufficient data. Although large-scale survey programmes such as the DHS and Multiple Indicator Cluster surveys are useful for estimating mortality in many countries, some countries have preferred to conduct their own programmes and do not necessarily share the microdata attained. Because we estimated mortality in an unconventional age group, the public availability of microdata is key for further analysis. Additional efforts should be made to enhance collaborations with country partners to identify additional data sources, foster a constructive dialogue on the methods, and improve country ownership and use of the final estimates.^26^, ^27^ The inclusion of estimates related to children aged 5–14 years in the consultation process organised by UNICEF and the WHO with government ministries of health and national statistics offices is an important step in this direction.

Efforts to estimate mortality in children aged 5–14 years from surveys, censuses, and vital statistics should be pursued in the future, and there remains room for improvement by future iterations. We suggest at least five areas for development. First, we did not use summary birth histories. More research is required to evaluate the validity of indirect methods when used to estimate mortality among older children. These indirect estimates would enable a better estimation of mortality in countries where the primary data sources are still population censuses. Second, an increasing number of countries are reporting their vital statistics estimates to WHO but not all country-years are used because of the likely incompleteness of death reporting. There is no well established method to assess the completeness of death reporting of children, except through comparison between estimates from vital statistics and full birth histories.^28^ As a result, the decision to include or exclude vital registration estimates remains partly based on expert assessments done on a country-by-country basis. Improvements in methods are required to better assess the percentage of deaths that are registered and to potentially include partial vital statistics data. Third, we assumed that mortality rates in children aged 5–14 years estimated from full birth histories are unaffected by HIV-related survivor biases. More research is needed to evaluate the magnitude of these biases and develop adjustment methods, which should account for the coverage of antiretroviral therapy and prevention of mother-to-child transmission of HIV. Fourth, the modelling strategy could be reassessed. At present, estimates of mortality among children younger than 5 years and children aged 5–14 years are mostly obtained independently (except for in countries with insufficient _10_q_5_ data points), but with the same B3 model and similar data sources. An alternative method would be to use pairs of measurements of _10_q_5_ and under-5 mortality rate when both indicators are available from the same sources and to model the association between the two indicators. This strategy is currently used by UN IGME to estimate neonatal mortality, whereby an overall expected ratio between neonatal mortality and under-5 mortality rate (given the level of under-5 mortality) is combined with a country-specific multiplier to capture deviations. Further work is required to establish if this alternative modelling approach outperforms the current approach for _10_q_5_ in validation exercises.^29^ Finally, we restricted our study to children younger than 15 years, and we did not compute mortality rates for adolescents aged 15–19 years. This restriction is based on a concern that full birth histories could be too affected by truncation bias because of women only being interviewed when they are aged 15–49 years. The use of sibling histories, which provide information on the survival of brothers and sisters, could be explored to cover older age groups.^25^ Beyond these methodological developments, better primary data are required for developing reliable and timely country-specific estimates. Ultimately, the only way to ensure that all deaths are counted on a continuous basis and causes of death are certified by a medical practitioner is to strengthen vital statistics systems. Vital statistics systems are particularly useful to measure rare events, as is the case in deaths of children aged 5–14 years.

Policy interest in mortality in adolescents has increased in recent years. For example, the Global Strategy for Women's, Children's, and Adolescents' Health (2016–30), which was launched in 2015 to support the Sustainable Development Goals, now includes adolescent mortality (age 10–19 years) among its key indicators. It will be important to quantify mortality in this age range on the basis of empirical analyses of surveys, censuses, and vital statistics rather than inference from relational models. Our study did not specifically target adolescents aged 10–19 years and focused on children aged 5–14 years, but it showed that mortality rates in this age range have not declined as fast as among children younger than 5 years. A previous study^30^ in countries with good-quality vital statistics suggested that improvements in mortality in young adults aged 15–24 years were less than among children aged 5–14 years. Together, these results necessitate detailed assessments of age-specific mortality trends in children and adolescents. They also highlight the need for increased attention of the global health community to health outcomes after age 5 years.
