# Supplementary material for: Global, regional, and national mortality trends in older children and young adolescents (5–14 years) from 1990 to 2016: an analysis of empirical data
Source: Lancet Glob Health. 2018 Sep 14;6(10):e1087–99. doi: 10.1016/S2214-109X(18)30353-X (PMC6139755; doi:10.1016/S2214-109X(18)30353-X)
Supplement: Supplementary appendix [file mmc1.pdf]

THE LANCET
Global Health

Supplementary appendix

This appendix formed part of the original submission and has been peer reviewed. We post it as supplied by the authors.

Supplement to: Masquelier B, Hug L, Sharrow D, et al. Global, regional, and national mortality trends in older children and young adolescents (5–14 years) from 1990 to 2016: an analysis of empirical data. *Lancet Glob Health* 2018; **6:** e1087–99.

Appendix

Global, regional, and national levels and trends in mortality among older children (5-9) and young adolescents (10-14) from 1990 to 2016

Contents

1 Database 1

2 Estimation model 6
2.1 Statistical model . . . 6
2.2 Bayesian penalized splines regression . . . 6
2.3 Data model . . . 7
2.4 Extrapolations . . . 10
2.5 Computation . . . 10
2.6 Adjustments . . . 10
2.7 Model validation . . . 13
2.8 Deriving estimates for countries with insufficient data . . . 16
2.9 Estimation of the number of deaths in children aged 5-9, 10-14 and 5-14 . . 17

3 Regional classification 19

4 List of data series 20

5 Mortality levels and trends 33

6 Country-specific plots 52

---

1 Database

Data sources for the mortality rates were nationally representative birth histories from surveys, recent household deaths reported in censuses, nationwide vital registration and sample registration systems:

1. *Civil registration and sample vital registration (SVR):* Vital registration data and mortality trends obtained from the model are presented in Figure S1 for France and Egypt. The calculation of the probability ${}_{10}q_5$ is derived from a standard period abridged life table. The inputs are number of deaths for age groups 5-9 (noted ${}_5D_5$) and for the age group 10-14 (${}_5D_{10}$), as well as the mid-year population for the same age groups (${}_5P_5$ and ${}_5P_{10}$).

   - The death rate for age group 5-9, ${}_5M_5$ is obtained by dividing ${}_5D_5$ by ${}_5P_5$.

- The probability ${}_5q_5$, which is the risk of dying between age 5 and age 10, is obtained as ${}_5q_5 = (5 \times {}_5M_5)/[1 + (5 - {}_5a_5) \times {}_5M_5]$, where ${}_5a_5$ is the average number of years lived by children who died in the age group 5-9 - set at 2.5 for all countries for this study.
- The same calculation is applied for ${}_5q_{10}$.
- The probability ${}_{10}q_5$ is computed as ${}_{10}q_5 = 1000 \times (1 - (1 - {}_5q_5)(1 - {}_5q_{10}))$.

The numbers of deaths and mid-year populations refer to both sexes. They were recalculated by pooling data from successive years together such that the coefficient of variation (the standard error of estimates divided by the estimates) is lower than 10%. The standard errors of estimates were calculated using a Poisson approximation, based on the number of children turning 5 in each year, as estimated by the Population Division of the United Nations (1).

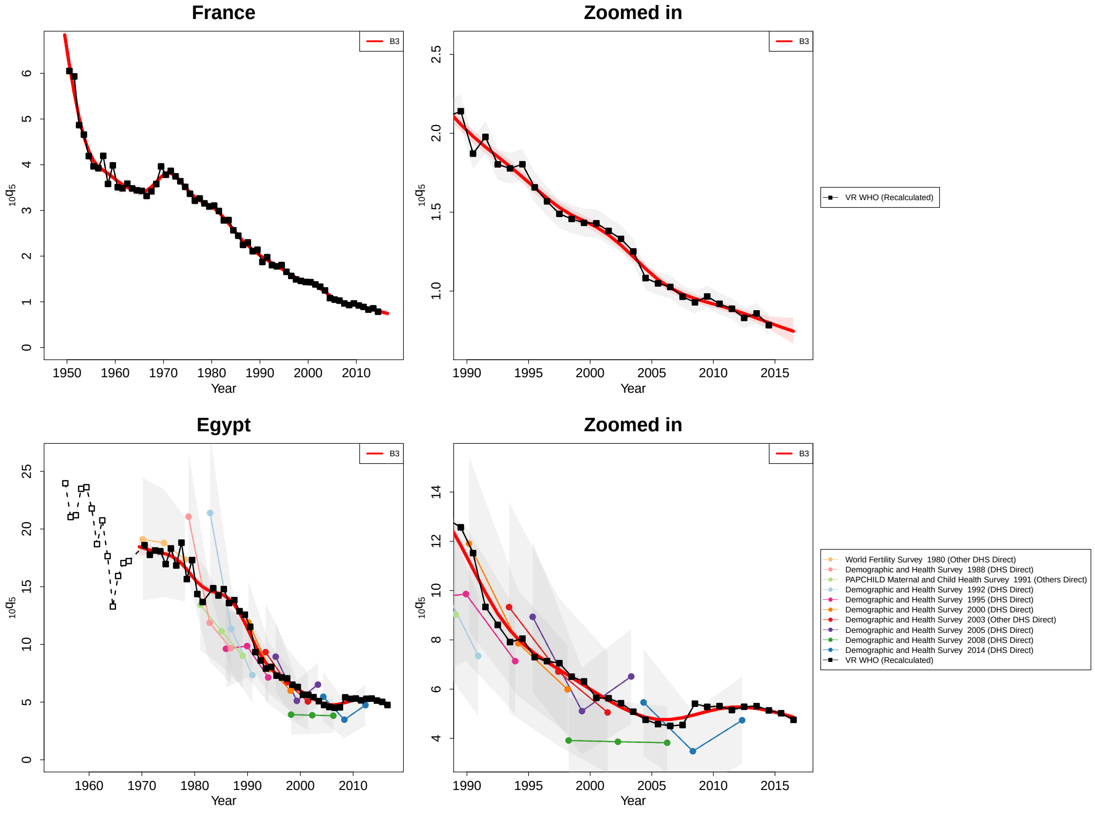

Figure S1 – ${}_{10}q_5$ *data series and estimates for France and Egypt.*
Note: B3 estimates are represented by the solid red lines and 90% UIs are displayed with the red shaded areas. The grey shaded areas represent the standard errors of the observations. In Egypt, vital registration estimates for the period before 1970 are not included in the statistical model due to incompleteness of death registration.

In 7 countries (Azerbaijan, Egypt, Guatemala, Panama, Turkmenistan, Trinidad and Tobago, South Africa), vital registration data were incorporated to estimate mortality among older children, despite being deemed to be too incomplete to be used for under-five mortality. When the completeness of death reporting among adults was estimated by WHO to be at 90% or more, vital registration data were included to estimate the probability ${}_{10}q_5$.

2. *Census*: Censuses often include questions on household deaths in the last 12 months, which can be used to calculate mortality estimates for children aged 5-14. The calculation of the probability ${}_{10}q_5$ is again derived from a standard period abridged life table. In China, we used published estimates from census data from 1982 to 2000, adjusted for incompleteness of death reporting with the General Growth Balance method (2). We also evaluated the completeness of death reporting in the 2010 census with the General Growth Balance method (3).

3. *Surveys*: Mortality estimates of children aged 5-14 can be derived from the full birth history (FBH) module which contains a series of detailed questions on each child a woman has given birth to during her lifetime. The full birth histories were used to estimate the probability of dying in children aged 5-14 (${}_{10}q_5$) for three reference periods prior to each survey (0-3 years prior to the survey, 4-7 years, and 8-11 years). Examples of Demographic and Health Surveys (DHS) series are presented alongside mortality trends obtained from the B3 model in Figure S2 for Bolivia and Cambodia. The original data sets, which have one row for each woman aged 15-49, were reshaped into files containing one row for each child. Then, using the reported dates of birth and ages at death, each life course was split into different spells; a new spell was created at every birthday and every time the number of completed years preceding the survey changes (see the Lexis diagram in Fig. S3). These spells were then aggregated to form a new dataset in which each row corresponds to a person-period, that is, a unique combination of a given age, sex, and time preceding the survey. For each person-period, the follow-up time and the number of deaths were computed and weighted by the sample weights. Annual mortality rates were converted into probabilities ${}_1q_x$ and chained together to form the probability ${}_{10}q_5$.

Fewer sources were available for estimating mortality in the age group 5-14 than for children under age 5. Some surveys or censuses could not be included, because estimates of the probability of dying in children aged 5-14 (${}_{10}q_5$) were not reported in published reports, or the microdata were not available.

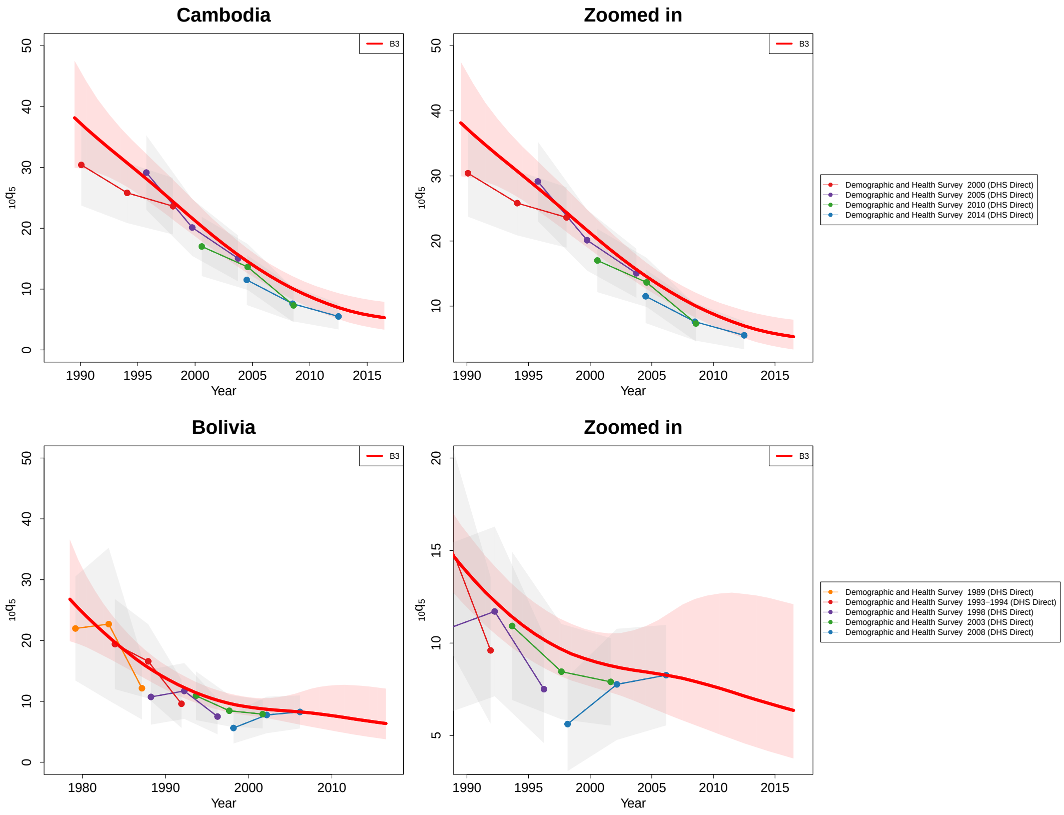

Figure S2 – $_{10}q_5$ *data series and estimates for Bolivia and Cambodia.*
Note: B3 estimates are represented by the solid red lines and 90% UIs are displayed with the red shaded areas.

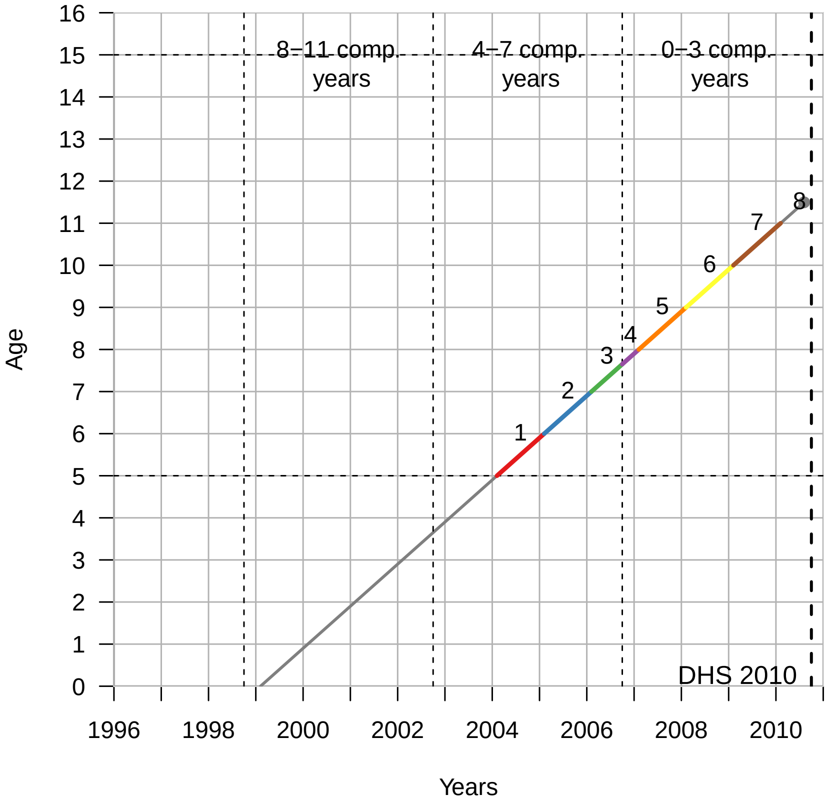

Figure S3 – *Lexis diagram illustrating how life courses are split into different spells to generate person-periods.*
Note: This example is based on the DHS conducted in Rwanda in 2010, with a child born in January 1999 who died in July 2010 at 11.5 years. This child would contribute to 8 different person-periods. The thick vertical dashed line shows the time of the survey, and the three dashed lines identify the three reference periods before data collection.

2 Estimation model

2.1 Statistical model

To obtain a smooth trend in the probability ${}_{10}q_5$, we used the model used by the UN IGME to monitor trends in under-five mortality and applied it to the 5-14 age group. This is a Bayesian penalized B-splines bias-reduction model, referred to as the B3 model. It was developed by Alkema & New (2014). We repeat below the main features of the B3 model to highlight a few changes implemented for the 5-14 age group.

We let u_i denote the observed probability ${}_{10}q_5$ for the observation i in country $c[i]$ and year $t[i]$: $u_i = U_{c[i]}(t[i]) \cdot \varepsilon_i$, where $U_c(t)$ denotes the true probability ${}_{10}q_5$ and $\varepsilon_i > 0$ is the error multiplier. On the natural log-scale, this corresponds to

$$y_i = f_{c[i]}(t[i]) + \delta_i, \tag{1}$$

where $y_i = \log(u_i)$, $f_c(t) = \log(U_c(t))$, and $\delta_i = \log(\varepsilon_i)$.

2.2 Bayesian penalized splines regression

The regression spline model for $f_c(t)$ from Eq.(1) is given by:

$$f_c(t) = \sum_{k=1}^{K_c} B_{c,k}(t)\alpha_{c,k}, \tag{2}$$

where $\alpha_{c,k}$ refers to splines coefficient k in country c and $B_{c,k}(t)$ the k-th spline, evaluated in year t, given by a third order B-spline (5; 6). Equally spaced knots were used such that the resulting splines are non-zero for a total of $4 \cdot I$ years, where I refers to the in-between knots interval length. We chose $I = 2.5$ years such that each spline is non-zero for 10 years. In each country, the knots were placed such that the largest two splines $B_{c,K_c-2}(t)$ and $B_{c,K_c-1}(t)$ in the most recent observation year $t = t_{n_c}$ have equal height while $B_{c,K_c}(t)$ is close to zero.

When fitting the splines model from Eq.(2) to the observations, second-order differences in adjacent spline coefficients ($\Delta^2\alpha_k = \alpha_k - 2\alpha_{k-1} + \alpha_{k-2}$) are penalized to guarantee smoothness of the resulting ${}_{10}q_5$ trajectory. Let $\boldsymbol{t}_c = (t_{c,1}, \ldots, t_{c,n_c})'$ refer to the vector of country-specific observation years with spline model estimate $f_c(\boldsymbol{t}_c) = (f(t_{c,1}), \ldots, f(t_{c,n_c}))'$. The splines model $f_c(\boldsymbol{t}_c) = \boldsymbol{B}_c(\boldsymbol{t}_c)\boldsymbol{\alpha}_c$, with $\boldsymbol{B}_c(\boldsymbol{t}_c) = (B_{c,1}(\boldsymbol{t}_c), \ldots, B_{c,K_c}(\boldsymbol{t}_c))$, $\boldsymbol{\alpha}_c = (\alpha_{c,1}, \ldots, \alpha_{c,K_c})'$, can be written as follows (7; 6):

$$\begin{aligned} f_c(\boldsymbol{t}_c) &= \boldsymbol{B}_c(\boldsymbol{t}_c)\boldsymbol{G}_{K_c}\boldsymbol{b}_c + \boldsymbol{Z}_c(\boldsymbol{t}_c)\boldsymbol{e}_c, \\ \boldsymbol{G}_{K_c} &= (\boldsymbol{1}_{K_c}, \boldsymbol{g}_{K_c})', \text{ where } \boldsymbol{g}_{K_c} = (1 - K_c/2, \ldots, K_c - K_c/2)', \\ \boldsymbol{Z}_c(\boldsymbol{t}_c) &= \boldsymbol{B}_c(\boldsymbol{t}_c)\boldsymbol{D}'_{K_c}(\boldsymbol{D}_{K_c}\boldsymbol{D}'_{K_c})^{-1}, \end{aligned} \tag{3}$$

where in difference matrix $\boldsymbol{D}_{K_c}$, $D_{K_c,i,i} = D_{K_c,i,i+2} = 1$, $D_{K_c,i,i+1} = -2$ and $D_{K_c,i,j} = 0$ otherwise.

The first part in Eq.(3), $\boldsymbol{B}_c(\boldsymbol{t}_c)\boldsymbol{G}_{K_c}\boldsymbol{b}_c$, describes the linear trend during the observation period, and the second part $\boldsymbol{Z}_c(\boldsymbol{t}_c)\boldsymbol{e}_c$ describes the fluctuations around the main trend. The unknown parameters are given by $\boldsymbol{b}_c = (b_{c,0}, b_{c,1})'$, and $\boldsymbol{e}_c = \boldsymbol{D}_{K_c}\boldsymbol{\alpha}_c$, where $\boldsymbol{e}_c = (e_{c,1}, \ldots, e_{c,Q_c})'$, with $Q_c = K_c - 2$ and $e_{c,q} = \Delta^2\alpha_{c,q+2}$ for $q = 1, \ldots, Q_c$. Second-order differences are penalized by imposing

$$e_{c,q} \sim N(0, \sigma_c^2), \text{ for } q = 1, \ldots, Q_c, \tag{4}$$

where variance σ_c^2 determines the extent of smoothing; a smaller variance corresponds to smoother trajectories. In the limit when σ_c decreases to zero, a linear fit for $\log({}_{10}q_5)$ is obtained.

The model is fit in the Bayesian framework. No information on levels or trends during the observation period is exchanged across countries when estimating the spline coefficients. Information exchanged across countries only concerns the variability of the (second order) difference in the spline coefficients through a multilevel model. The variance of $e_{c,q} = \alpha_{c,q+2}$ - $2\alpha_{c,q+1} + \alpha_{c,q}$ is estimated hierarchically:

$$\log(\sigma_c) \sim N(\chi, \varphi_\sigma^2). \tag{5}$$

Vague prior distributions are used for the $\boldsymbol{b}_c$'s and the hyper parameters for the hierarchical model for the σ_c's (4).

2.3 Data model

The error distribution for observations from complete VR or SVR is given by

$$\delta_i \sim N(0, \tau_i^2/u_i^2),$$

where τ_i/u_i is the stochastic standard error. These errors are calculated using a Poisson approximation (using the numbers of children turning 5 from the World Population Prospects 2017) and set to a minimum of 2.5%. If the Poisson sampling standard error cannot be calculated (e.g. for Sample Registration Systems), it is set to 10%.

For non-VR data, δ_i, the error term on the log-scale, is specified as follows:

$$\delta_i = E_i + S_i \cdot X_i,$$

where E_i is the mean bias, S_i the scale parameter and X_i determines the distribution for observation i.

For all non-VR data series with repeated observations, mean biases were modeled as a linear function of the retrospective period of the observation in the survey:

$$E_i = \beta_{0,s[i]} + \beta_{1,s[i]} \cdot \pi_i, \tag{6}$$

where $\beta_{0,s[i]}+\beta_{1,s[i]}\cdot\pi_i$ represents the bias in level and trend as a function of the retrospective period π_i for observation i in data series $s[i]$. The retrospective period π_i was centered at

5 years for this study. The bias in the level of the series, $\beta_{0,s}$ is estimated with a multilevel model:

$$\beta_{0,s} \sim N(\mu_{0,d[s]}, \gamma^2_{0,d[s]}), \tag{7}$$

where $d[s]$ refers to the source type of series s, based on data source (the source types with multiple observations per series are given by (Standard) DHS Direct, Other DHS Direct (including Special, Interim and National DHS, Malaria Indicator Surveys, AIDS Indicator Surveys and World Fertility Surveys), and Others Direct), and $\mu_{0,d}$ and $\gamma^2_{0,d}$ represent source type-specific mean bias and between-series variance respectively. A similar approach is used to estimate the slope $\beta_{1,s}$:

$$\beta_{1,s} \sim N(\mu_{1,d[s]}, \gamma^2_{1,d[s]}), \tag{8}$$

where $\mu_{1,d}$ and $\gamma^2_{1,d}$ represent the mean slope and the between-series variance for source type d. For single observations constructed from reported household deaths, and single observations obtained from reported life tables, we assume that $E_i = \mu_{0,d[s[i]]}$.

Scale parameter S_i is modeled as a combination of sampling variance τ_i^2/u_i^2 (based on sampling variance τ_i^2 for ${}_{10}q_5$) and non-sampling variance $\omega^2_{d'[s[i]]}$:

$$S_i^2 = \omega^2_{d'[s[i]]} + \tau_i^2/u_i^2, \tag{9}$$

where source type $d'[s]$ for series s refers to a further breakdown of source types to distinguish between DHS, Other DHS, MICS surveys and recent household deaths. Where the sampling variance for non-VR data is not reported, we assume a sampling standard error of 10%.

Finally, the distribution for δ_i is given by:

$$X_i \sim \begin{cases} N(0,1) & \text{for Standard and Other DHS direct,} \\ t_\nu & \text{otherwise, with } \nu \sim U(2,30). \end{cases} \tag{10}$$

A t-distribution with ν degrees of freedom is used for observations that are not obtained from Standard or Other DHS, in accordance with the model used by UN IGME for under-five mortality.

All model parameters in Eq.(6)–(10) were assigned vague prior distributions. In the UN IGME model for under-five mortality, an informative prior distribution was used for the mean bias $\mu_{0,d}$ for the DHS Direct series, but here we used vague prior distributions for all parameters.

Figure S4 presents mean biases and 90% prediction intervals for "new" data points by type of data source. This visualization shows that for a "true" level of the probability ${}_{10}q_5$ of 7 deaths before age 15 per 1000 children at age 5, the mean bias will vary across data sources and with the length of the retrospective period (in the survey estimates). The dark lines refer to prediction intervals based on uncertainty in the bias parameters only (excluding sampling and non-sampling variability), while the intervals in light colors are based on uncertainty excluding the sampling variability. The intervals represented with

dark colors are large, indicating that there is substantial variability in biases across data series of the same source type. Mean biases tend to be negative in sample surveys and slightly positive for data on recent household deaths (represented in pink). The median of the predicted ${}_{10}q_5$ for a retrospective period of 2 years (our first estimate from DHS) is 6.1 per thousand (5.1-7.3), for a true level of 7 per thousand. As a result, in countries where the trend in mortality is largely informed by DHS surveys, the final estimates are adjusted upwards as compared to the data and therefore the final estimated series may fall slightly above the original survey data points (see Fig. S2). Interestingly, there is no significant change in biases from DHS surveys as the reference moves further back in time.

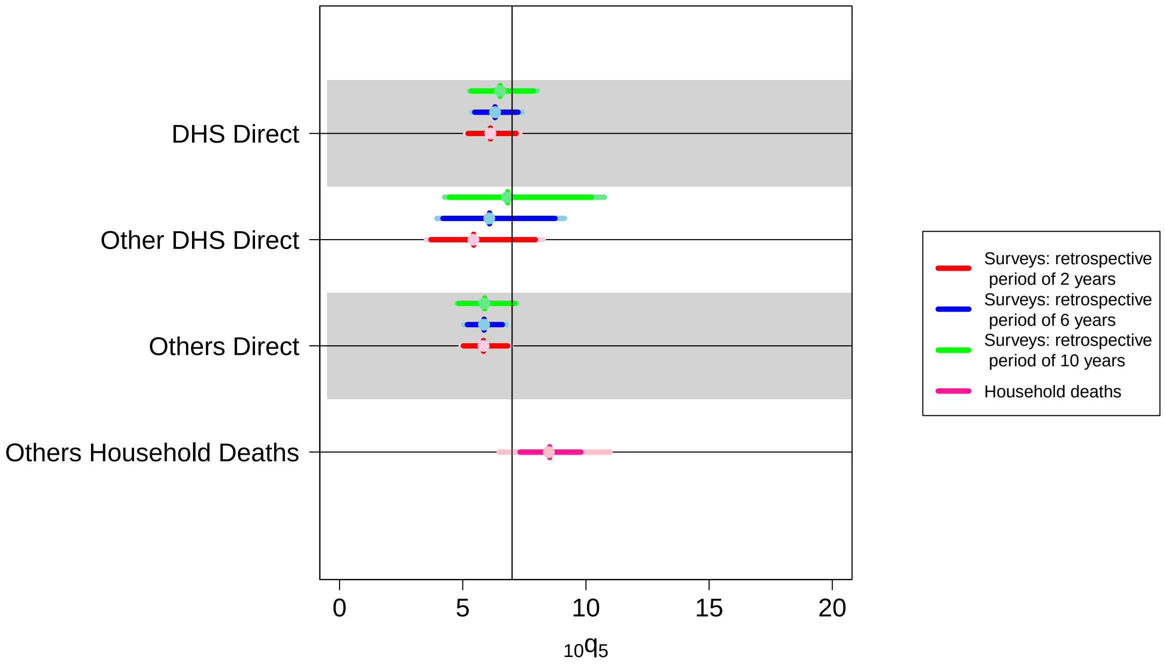

Figure S4 – *90% prediction intervals for new data points by source type and length of the retrospective period.*
Note: For a "true" probability ${}_{10}q_5$ *of 7 deaths per 1,000 children aged 5 (represented by the vertical black line), the predicted mean observed* ${}_{10}q_5$ *is represented by the colored vertical lines. The difference between the mean* ${}_{10}q_5$ *and 7 represents the mean bias. The dark horizontal lines represent the 90% prediction intervals for an observation based on uncertainty in the bias parameters only (excluding sampling and non-sampling variability), while the 90% prediction intervals excluding the sampling variability are in light colors.*

A computationally cheaper model was implemented to allow for updates of country-specific estimates with additional data without the need to re-run the global model. Non-country-specific parameters were fixed at the posterior medians from the global model run. Both models resulted in very similar estimates. One model adjustment was later incorporated in the country-specific models. In some countries, there was little smoothing of the splines fit, resulting in unrealistic short-term changes for a subset of country-years without VR data. For these countries, instead of using country-specific smoothing determined by

variance parameter σ_c^2 from Eq.(5), we set $\sigma_c = \exp(\hat{\chi})$, where $\hat{\chi}$ refers to the posterior median of μ, which is referred to as the global smoothing level. This subset includes 1) countries with both VR and non-VR data, 2) countries that have VR data with gaps more than 5 years in the data, as well as 3) small countries with less than 10,000 children aged 5 in 2015 (based on the World Population Prospects 2017).

2.4 Extrapolations

For extrapolations, we implemented a logarithmic pooling procedure to combine country-specific posterior predictive distributions for changes in spline coefficients with a global posterior predictive distribution. This procedure was applied to modify the posterior predictive distributions for $\alpha_{c,k}$ for $k = K_c, K_c + 1, \ldots, P_c$, where P_c refers to the last spline in the projection period of interest. While α_{c,K_c} was among the spline coefficients that were included in the observation period up to year t_{n_c}, it was included in the set of "projected" coefficients to be pooled because its estimate is based mainly on an extrapolation of past changes. The logarithmic pooling weight κ, which determines the extent of pooling, was set at 0.5 for this study. Further details on the logarithmic pooling procedure are provided in Alkema & New (2014)(4).

2.5 Computation

A Markov Chain Monte Carlo (MCMC) algorithm was employed to sample from the posterior distribution of the parameters in the global and country-specific models with the use of the software JAGS. For the global run, 10 parallel chains with different starting points were run with a total of 75,000 iterations in each chain. Of these, the first 25,000 iterations in each chain were discarded as burn-in and every 20th iteration after was retained. The resulting chains contained 2,500 samples each. For the country-specific runs, we ran 10 chains with a total of 62,500 iterations in each chain. Of these, the first 25,000 iterations in each chain were discarded as burn-in and every 30th iteration after was retained. The resulting chains contained 1,250 samples each.

Standard diagnostics checks were used to check convergence. Estimates of relevant quantities are given by the posterior medians while 90% uncertainty intervals (UIs) were constructed from the 5% and 95% percentiles of the posterior sample.

2.6 Adjustments

Adjustments were made to account for abrupt increases in mortality due to conflicts and/or disasters, which would otherwise not be present in the smoothed mortality curves obtained from the statistical model. The approach is identical to the one taken for under-five mortality: the splines regression model is fitted to "extreme event-free" observations (obtained as the observed values of the probability ${}_{10}q_5$ minus the extreme events). Uncertainty intervals for ${}_{10}q_5$ for the crises-years are based on the uncertainty in crisis-free ${}_{10}q_5$. The following criteria were used to identify crises:

1. The crisis was isolated to a few years,
2. Crisis deaths among children 5-14 were >10 % of non-crisis deaths in this age group,
3. The crisis ${}_{10}q_5$ was greater than 0.2 per 1,000,
4. The number of crisis deaths among children 5-14 was greater than 10.

Because the background mortality rates are relatively low in this age group, crisis deaths will represent a larger share of deaths, and therefore there were more crises meeting these criteria than for under-five mortality. In total, adjustments for crises were made in 37 countries, as detailed in Table S1 below.

In populations severely affected by HIV/AIDS, HIV-positive (HIV+) children will be more likely to die than other children, and will also be less likely to be reported since their mothers will have been more likely to die also. However, no adjustment was included for HIV-related biases in the age group 5-14, since no method currently exists to estimate the magnitude of this bias in the probability ${}_{10}q_5$.

Countries	Years	Type of event
Azerbaijan	1992	Nagorno-Karabakh conflict
Bangladesh	1991	Cyclone Gorky
Bosnia & Herzegovina	1992-1995	Bosnian war
China	2008	Sichuan earthquake
Congo	1997-1998	Civil war
Croatia	1993-1995	Croatian war of independence
El Salvador	2001	Earthquake
Georgia	1991-1993	Civil war
Greece	1999	Earthquake
Haiti	2010	Earthquake
Honduras	1998	Hurricane Mitch and landslides
Indonesia	2004	Indian Ocean earthquake and tsunami
Iran	2003	Bam earthquake
Japan	2011	Earthquake
Kuwait	1990-1991	First Gulf War
Lebanon	1990	Civil war
Liberia	2003	Second Liberian Civil War
Libya	2011-2015	Civil War
Macedonia	2001	2001 Insurgency
Maldives	2004	Indian Ocean earthquake and tsunami
Moldova	1992	Transnistria War
Myanmar	2008	Cyclone Nargis
Nepal	2015	Earthquake
Nicaragua	1998	Hurricane Mitch and landslides
Pakistan	2005	Earthquake
Papua New Guinea	1998	Tsunami
Rwanda	1993-1995	Genocide
Samoa	2009	Earthquake and tsunami
Sri Lanka	2004	Indian Ocean earthquake and tsunami
Syria	2011-2015	Civil war
Tajikistan	1992-1996	Civil war
Thailand	2004	Indian Ocean earthquake and tsunami
Turkey	1999	Earthquake
Ukraine	2014-2015	Ukraine crisis
Vanuatu	1997	Earthquake
Venezuela	1999	Vargas flood and mudslides
Yemen	1991	Earthquake

Table S1 – *Countries and years referring to crises being explicitly incorporated into the 5-14 mortality estimates*

2.7 Model validation

We assessed B3 model performance for ${}_{10}q_5$ through an out-of-sample validation exercise. Instead of leaving out observations at random, a training set was constructed by removing all data collected in or after 2008 (i.e. about 20% of observations were left out). In other words, all retrospective observations from a survey carried out in 2008 were left out of the training set, even if they referred to periods before 2008 (see Figure S5 for Ghana).

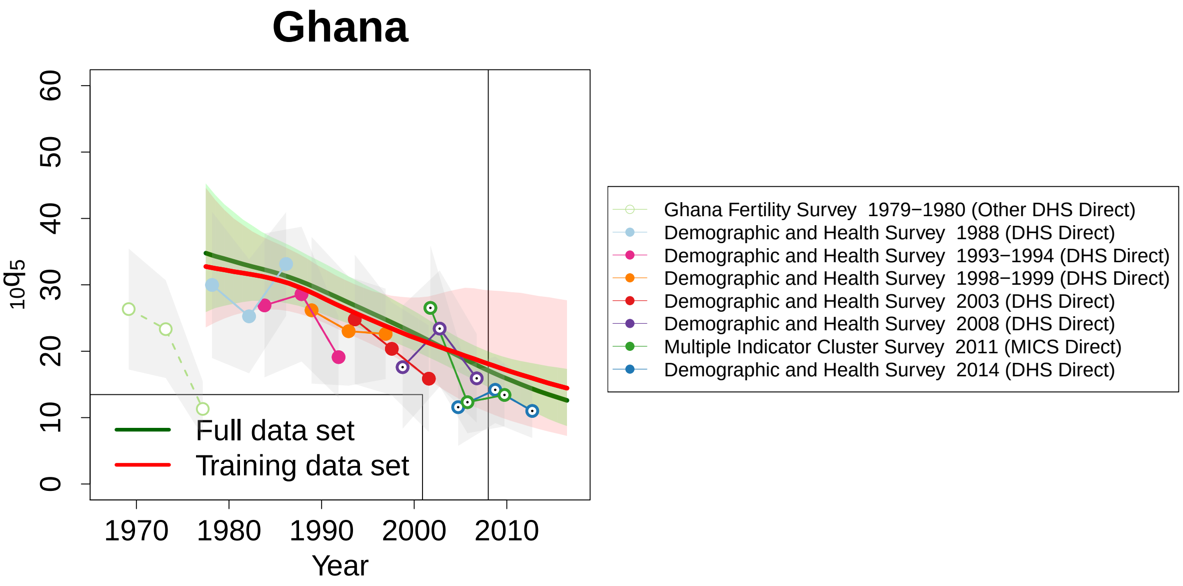

Figure S5 – *Probability of dying between ages 5 and 15 years from various surveys in Ghana and trend obtained from the B3 model.*
Note: The last surveys from 2008, 2011 and 2014 are left out to construct the training set because they were conducted in or after 2008.

We present below two sets of validation results: based on left-out observations (Tables S2 and S3), and based on the comparison between estimates obtained from the training and full data set (Table S4). In all cases, results are presented only for estimates without crises in high mortality countries, defined here as countries with a probability ${}_{10}q_5$ above 8 per thousand children aged 5 in 1990. We present validation measures for ${}_{10}q_5$ alongside those obtained by Alkema and New (2014) for U5MR (these results refer to high mortality countries with U5MR above 40 per thousand in 1990, without HIV adjustments).

Table S2 presents the percentage of observations falling below and above the 90% uncertainty intervals based on the training set. These percentages were calculated for 100 sets of left-out observations, where each set consisted of a random sample of one left-out observation per country. The results presented in Table S2 are the median and standard deviation of these percentages based on the outcomes in the 100 sets. In both periods (before and after 2007), there is a larger percentage of ${}_{10}q_5$ observations falling below the 90% uncertainty intervals than the percentage falling above. If the model is well calibrated, we

expect to observe around 5% of observations falling below and above the 90% uncertainty intervals based on the training set, and Table S2 suggests that there is some asymmetry in coverage. The upper bound for the period after 2007 is conservative, as no observation falls above it.

The results reported in Table S3 refer to the mean and median relative error and absolute relative error. Errors are defined as $\epsilon_i = u_i - \tilde{u}_i$, where $\tilde{u}_i$ is the posterior median of the predictive distribution for a left-out observation u_i based on the training set. The median or mean relative error (MRE) and the median or mean absolute relative error (MARE) are twice larger than those of under-five mortality.

Table S2 – *Validation results based on left-out observations: Median and SD of percentage of observations below and above 90% predictions intervals based on the training set, from 100 sets of left-out observations, in countries with data in both the training and test set and left-out observations in the period of interest.*

	$_{10}q_5$ (this study)		
	% Below	% Above	Nb countries
Year $\leq$ 2007	2 (2.1)	2 (1.3)	49
Year $>$ 2007	5.8 (1.2)	0 (0.7)	52

	$_5q_0$ (Sc: Alkema & New 2014)		
	% Below	% Above	Nb countries
Year $\leq$ 2005	7.0 (2.4)	7.0 (1.8)	71
Year $>$ 2005	6.2 (1.5)	1.5 (1.0)	65

Table S4 presents the results based on a comparison between estimates based on the training and full data set. The error in the estimate based on the training sample is defined as $\epsilon_{c,t} = \widehat{U}_c(t) - \widetilde{U}_c(t)$, where $\widetilde{U}_c(t)$ refers to the posterior median estimate based on the training sample, and $\widehat{U}_c(t)$ refers to the $_{10}q_5$ estimate obtained from the full data set for country c in year t. Relative error is defined as $\epsilon_{c,t}/\widehat{U}_c(t) \cdot 100$. Again, the validation measures are the mean and median relative error and absolute relative error, and the percentage of estimates obtained from the full dataset that are falling below or above the 90% uncertainty intervals obtained from the training set only.

Together, these results suggest that the model performs reasonably well, although there is still room for improvement when estimating mortality in the age group 5-14.

Table S3 – *Validation results based on left-out observations: median or mean error (ME), median or mean relative error (MRE), median or mean absolute relative error (MARE) based on the training set.*

$_{10}q_5$ (this study)

**Median**	ME	MAE	MRE	MARE
Year ≤ 2007	-0.4 (0.5)	4.4 (0.5)	-5.0 (3.6)	33.3 (3.9)
Year > 2007	0 (0.4)	3.5 (0.6)	0.9 (3.2)	31.4 (2.9)
**Mean**	ME	MAE	MRE	MARE
Year ≤ 2007	1 (0.5)	5.6 (0.5)	-23.2 (6.6)	47.8 (6)
Year > 2007	-0.4 (0.1)	2.4 (0.1)	-32.1 (1.5)	50.1 (1.5)

$_5q_0$ (Sc: Alkema & New 2014)

**Median**	ME	MAE	MRE	MARE
Year ≤ 2005	-1.7 (1.3)	10.7 (1.3)	-1.9 (1.3)	12.9 (1.4)
Year > 2005	-3.7 (0.1)	7.6 (1.2)	-10.7 (1.1)	17.5 (1.5)
**Mean**	ME	MAE	MRE	MARE
Year≤ 2005	-1.9 (1.4)	15.8 (1.1)	-3.8 (1.6)	17.6 (1.3)
Year > 2005	-6.0 (0.5)	14.2 (0.5)	-14.8 (1.2)	25.8 (1.1)

Table S4 – *Validation measures based on the comparison between estimates based on the training and full data set.*

$_{10}q_5$

	Mean			Median				
	MRE	MARE	S	MRE	MARE	S	% below	% above
1990	6.4	8.2	0.6	5.7	6.0	0.4	0.0	1.9
2000	-4.1	11.5	0.6	-1.1	6.0	0.4	1.9	5.6
2007	-14.7	23.0	1.0	-6.6	14.4	0.9	5.6	3.7

$_5q_0$ (Sc: Alkema and New 2014)

	Mean			Median				
	MRE	MARE	S	MRE	MARE	S	% below	% above
2000	-2.4	4.4	1.2	-4.1	9	1.6	3.8	5.1
2005	-6.1	8.1	1.2	-8.9	14.7	1.6	7.7	1.3

2.8 Deriving estimates for countries with insufficient data

In 39 countries with insufficient data sources, we estimated mortality in children aged 5-14 based on the relationship observed between U5MR and the probability ${}_{10}q_5$ in countries for which the B3 model was used.

We did not use conventional model life tables such as the Coale-Demeny life table system (8), because, as we argue in the main text, the selection of the appropriate pattern is difficult to justify in the absence of accurate age-specific data. In addition, the pattern to retain should vary over time, as Figure 4 (in the main text) suggests. When comparing 1990 and 2016, we note that the ${}_5q_0$-to-${}_{10}q_5$ relationship is changing. In 1990, most estimates of the probability ${}_{10}q_5$ were below what would be predicted from the North pattern of Coale-Demeny life tables when indexed on U5MR . In 2016, more estimates were falling above the line of the expected ${}_{10}q_5$ based on the North pattern. This is confirmed by Table S5, below, which displays the median ratios of mortality rates between predictions based on the North model and estimates from the B3 model, in 1990 (columns 1-2), and 2016 (columns 3-4). In 1990, the median ratios were significantly below 1 in all regions in 1990 except East Asia and Pacific, and North America. By contrast, in 2016, the median ratios were not significantly different from 1, except in Latin America and Caribbean ($p = 0.002$).

	1990		2016	
	median ratios	Sign.	median ratios	Sign.
East Asia and Pacific	1.10		1.17	
Europe and Central Asia	0.95	*	1.07	
Latin America and Caribbean	0.66	***	0.88	**
Middle East and North Africa	0.77	**	0.95	
North America	1.12		0.89	
South Asia	0.55	*	0.67	
Sub-Saharan Africa	0.78	***	0.97	

Table S5 – *Median ratios between estimates inferred from the North pattern of Coale-Demeny life tables and B3 estimates of the probability* ${}_{10}q_5$
Note: significance levels in columns 2 and 4 were obtained from Wilcoxon signed-rank tests of the null hypothesis that the distribution of differences between (rank-transformed) model-based and B3 estimates are symmetric around 0.

In order not to use model life tables but still predict the probability ${}_{10}q_5$ for countries without sufficient data, we used a ordinary least-squares regression model to predict log(${}_{10}q_5$) from log(U5MR). Our model was of a form similar to the one used by Hill and colleagues (9):

$$log({}_{10}q_5) = \beta_0 + \beta_1(\text{Region}) + \beta_2\log(\text{U5MR}) + \beta_3\log(\text{U5MR})^2 + \beta_4\text{time}$$

where time refers to the number of years since 1990 and β_1(Region) are region-specific dummies.

The regression results presented in Table S6 confirm our observations based on Figure 4 (in the main text): Sub-Saharan Africa and East Asia and Pacific (the reference category)

have higher levels of ${}_{10}q_5$ for a given level of U5MR than other regions. By contrast, estimates of ${}_{10}q_5$ tend to be lower in Europe and Central Asia, and North America, even after controlling for the level of under-five mortality.

Table S6 – *Regression results for log(${}_{10}q_5$)*

	Dependent variable:
	log(${}_{10}q_5$)
log(${}_5q_0$)	0.355*** (0.026)
log(${}_5q_0$)^2	0.063*** (0.004)
Europe and Central Asia	−0.331*** (0.018)
Latin America and Caribbean	−0.190*** (0.020)
Middle East and North Africa	−0.167*** (0.022)
North America	−0.348*** (0.044)
South Asia	−0.085*** (0.027)
Sub-Saharan Africa	0.211*** (0.023)
time	−0.005*** (0.001)
Constant	−0.126*** (0.043)
Observations	4,212
R^2	0.926
Adjusted R^2	0.925
Residual Std. Error	0.302 (df = 4202)
F Statistic	5,806.602*** (df = 9; 4202)
Note:	*p<0.1; **p<0.05; ***p<0.01

2.9 Estimation of the number of deaths in children aged 5-9, 10-14 and 5-14

To calculate the number of deaths in children aged 5-14, age-specific probabilities (${}_5q_5$ and ${}_5q_{10}$) were converted into death rates (${}_5m_5$ and ${}_5m_{10}$), and multiplied by the population estimates from the World Population Prospects (2017 Revision). A specific database of mortality measurement for children aged 5-9 was constructed, from the same data sources, but only in computing mortality rates from age 5 up to age 10. A penalized spline regression model similar to the B3 model was used to estimate country-specific trends in the logit transformation of r ($log(r/(1-r))$), where r is the ratio of the probability ${}_5q_5$ to the median B3 estimate of ${}_{10}q_5$ (in the corresponding country-year). That approach ensures that the probability ${}_5q_5$ is constrained to be lower than the probability ${}_{10}q_5$. Countries excluded from B3 when estimating ${}_{10}q_5$ because they had insufficient data were also excluded when estimating ${}_5q_5$, and we used region-specific coefficients to relate U5MR and the probability ${}_5q_5$.

Because our estimates of mortality rates differ from the WPP estimates, there could be some differences in population numbers, and ultimately in numbers of deaths, if our estimates were to be used in the WPP to reconstruct demographic trends, and then recalculate the population at risk of dying in children aged 5-14. However, these differences would be very small. This is because only a fraction of children die between ages 5 and 15 (7.5 per 1000 children aged 5 in 2016 globally), resulting in small changes in the number of person-years of exposure. To illustrate how small these differences would be, we retain Sub-Saharan Africa, where the difference with the WPP estimates is the largest, and where mortality rates are the highest. Our estimates of the number of deaths in this region is about 150 000 deaths lower than WPP estimates. In 2016, according to our study, the probabilities ${}_5q_5$ and ${}_5q_{10}$ were respectively at 11.9 per 1000 and 6.6 per 1000 in this region, corresponding to central death rates of 2.4 per 1000 and 1.3 per thousand. If, to the mid-year population aged 5-14 estimated by the WPP for 2016 in Sub-Saharan Africa, we add half the additional 150 000 children who died in 2016 (that is, the difference between our study and the WPP deaths), we can recalculate the number of deaths based on this larger population at risk. The revised number of deaths would be only 1.4% higher than the number of deaths we estimated for 2016. In other words, a 21% difference in the number of deaths corresponds to small differences in the population at risk, due to relatively low mortality in the age group 5-14. For this reason, and because no other source of population estimates is as complete as the WPP, we retain the WPP estimates of the population aged 5-14.

3 Regional classification

Countries included in this study are classified in geographical regions according to the UNICEF classification (Figure S6). A full list of countries in each region is available on http://data.unicef.org/regionalclassifications/.

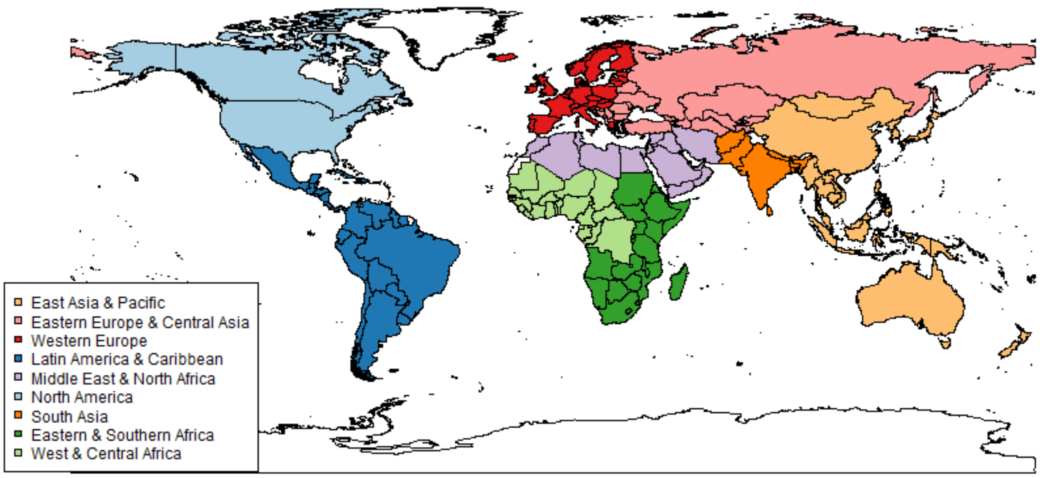

Figure S6 – *UNICEF Regional classification*

4 List of data series

Table S7 lists all countries included in the analysis, their region (according to UNICEF), the different data series included in the mortality database and indicates the type of data collection. This database is publicly available on CME Info (www.childmortality.org). Some data series were included in the database but excluded from the statistical model. They refer to incomplete vital registration data, or surveys for which an assessment by the UN IGME found evidence of substantial non-sampling errors or omission. The 'Inclusion' column identifies these data series with a 0. This column is also set to '0' for countries for which trends in ${}_{10}q_5$ were modelled based on trends in U5MR (because of insufficient data series) (see section 2.9).

Table S7 – *Data series included in the analysis*

Country	Region	Series	Years	Inclusion	Type
Afghanistan	South Asia	Census	1979	1	Household Deaths
		Afghanistan Mortality Survey (AMS) Excluding South Zone	2010	1	Household Deaths
		Afghanistan Mortality Survey (AMS) Excluding South Zone	2010	1	Full Birth Histories
		Demographic and Health Survey	2015	1	Full Birth Histories
Albania	Eastern Europe & Central Asia	Reproductive Health Survey	2002	0	Full Birth Histories
		Demographic and Health Survey	2008-2009	0	Full Birth Histories
Algeria	Middle East & North Africa	PAPCHILD Maternal and Child Health Survey	1992	1	Full Birth Histories
		PAPFAM Family Health Survey	2002	1	Full Birth Histories
		Multiple Indicator Cluster Survey	2012-2013	0	Full Birth Histories
		WHO VR (Recalculated)	1980-2015	1	Vital Registration
Andorra	Western Europe	WHO VR (Recalculated)	2002-2017	0	Vital Registration
Angola	Eastern & Southern Africa	Malaria Indicator Survey	2011	0	Full Birth Histories
		Demographic and Health Survey	2016	0	Full Birth Histories
Antigua and Barbuda	Latin America & Caribbean	WHO VR (Recalculated)	1984-2015	0	Vital Registration
Argentina	Latin America & Caribbean	WHO VR (Recalculated)	1970-2016	1	Vital Registration
Armenia	Eastern Europe & Central Asia	Demographic and Health Survey	2000	1	Full Birth Histories
		Demographic and Health Survey	2005	1	Full Birth Histories
		Demographic and Health Survey	2010	1	Full Birth Histories
		WHO VR (Recalculated)	1999-2016	1	Vital Registration
Australia	East Asia & Pacific	WHO VR (Recalculated)	1950-2016	1	Vital Registration
Austria	Western Europe	WHO VR (Recalculated)	1955-2016	1	Vital Registration
Azerbaijan	Eastern Europe & Central Asia	Demographic and Health Survey	2006	1	Full Birth Histories
		WHO VR (Recalculated)	1981-2017	1	Vital Registration
Bahamas	Latin America & Caribbean	WHO VR (Recalculated)	1975-2014	1	Vital Registration
Bahrain	Middle East & North Africa	WHO VR (Recalculated)	1992-2015	1	Vital Registration
Bangladesh	South Asia	World Fertility Survey	1975-1976	1	Full Birth Histories
		Demographic and Health Survey	1993-1994	1	Full Birth Histories
		Demographic and Health Survey	1996-1997	1	Full Birth Histories
		Demographic and Health Survey	1999-2000	1	Full Birth Histories
		Demographic and Health Survey	2004	1	Full Birth Histories
		Demographic and Health Survey	2007	1	Full Birth Histories
		Demographic and Health Survey	2011	1	Full Birth Histories
		Demographic and Health Survey	2014	1	Full Birth Histories
		SVR (from published reports)	1981-2016	1	Sample Vital Registration
Barbados	Latin America & Caribbean	WHO VR (Recalculated)	1956-2014	1	Vital Registration
Belarus	Eastern Europe & Central Asia	WHO VR (Recalculated)	1981-2017	1	Vital Registration
Belgium	Western Europe	WHO VR (Recalculated)	1954-2016	1	Vital Registration
Belize	Latin America & Caribbean	Family Health Survey	1991	1	Full Birth Histories
		WHO VR (Recalculated)	2001-2017	1	Vital Registration
Benin	West & Central Africa	Benin Fertility Survey	1981-1982	1	Full Birth Histories
		Demographic and Health Survey	1996	1	Full Birth Histories
		Demographic and Health Survey	2001	1	Full Birth Histories
		Demographic and Health Survey	2006	1	Full Birth Histories
		Demographic and Health Survey	2012	0	Full Birth Histories
Bhutan	South Asia	National Health Survey	1994	1	Household Deaths

Country	Region	Series	Years	Inclusion	Type
		National Health Survey	2000	1	Household Deaths
		Census	2005	1	Household Deaths
		National Health Survey	2012	1	Household Deaths
Bolivia (Plurinational State of)	Latin America & Caribbean	Demographic and Health Survey	1989	1	Full Birth Histories
		Demographic and Health Survey	1993-1994	1	Full Birth Histories
		Demographic and Health Survey	1998	1	Full Birth Histories
		Demographic and Health Survey	2003	1	Full Birth Histories
		Demographic and Health Survey	2008	1	Full Birth Histories
Bosnia and Herzegovina	Eastern Europe & Central Asia	WHO VR (Recalculated)	1985-2016	1	Vital Registration
Botswana	Eastern & Southern Africa	Family Health Survey	2008	0	Full Birth Histories
Brazil	Latin America & Caribbean	Demographic and Health Survey	1986	1	Full Birth Histories
		Demographic and Health Survey	1991	0	Full Birth Histories
		Demographic and Health Survey	1996	1	Full Birth Histories
		WHO VR (Recalculated)	2000-2016	1	Vital Registration
Brunei Darussalam	East Asia & Pacific	WHO VR (Recalculated)	1984-2017	1	Vital Registration
Bulgaria	Eastern Europe & Central Asia	WHO VR (Recalculated)	1964-2017	1	Vital Registration
Burkina Faso	West & Central Africa	Demographic and Health Survey	1993	1	Full Birth Histories
		Demographic and Health Survey	1998-1999	1	Full Birth Histories
		Demographic and Health Survey	2003	1	Full Birth Histories
		Demographic and Health Survey	2010	1	Full Birth Histories
Burundi	Eastern & Southern Africa	Demographic Survey	1970	1	Household Deaths
		Demographic and Health Survey	1987	1	Full Birth Histories
		Demographic and Health Survey	2010-2011	1	Full Birth Histories
Cabo Verde	West & Central Africa	Demographic and Reproductive Health Survey	1998	1	Full Birth Histories
		WHO VR (Recalculated)	1982-2014	1	Vital Registration
Cambodia	East Asia & Pacific	Demographic and Health Survey	2000	1	Full Birth Histories
		Demographic and Health Survey	2005	1	Full Birth Histories
		Demographic and Health Survey	2010	1	Full Birth Histories
		Demographic and Health Survey	2014	1	Full Birth Histories
Cameroon	West & Central Africa	World Fertility Survey	1978	1	Full Birth Histories
		Demographic and Health Survey	1991	1	Full Birth Histories
		Demographic and Health Survey	1998	1	Full Birth Histories
		Demographic and Health Survey	2004	1	Full Birth Histories
		Demographic and Health Survey	2011	1	Full Birth Histories
Canada	North America	WHO VR (Recalculated)	1950-2013	1	Vital Registration
Central African Republic	West & Central Africa	Demographic and Health Survey	1994-1995	0	Full Birth Histories
Chad	West & Central Africa	Demographic and Health Survey	1996-1997	1	Full Birth Histories
		Demographic and Health Survey	2004	1	Full Birth Histories
		Demographic and Health Survey	2014-2015	1	Full Birth Histories
Chile	Latin America & Caribbean	WHO VR (Recalculated)	1960-2016	1	Vital Registration
China	East Asia & Pacific	Adjusted Census Deaths	(1964-1982)	1	Household Deaths
		Adjusted Census Deaths	(1982-1990)	1	Household Deaths
		Adjusted Census Deaths	(1990-2000)	1	Household Deaths
		Adjusted Census Deaths	(1999-2000)	1	Household Deaths
		Adjusted Census Deaths	(2000-2010)	1	Household Deaths
		Population 1% Sample Survey	2005	1	Household Deaths

Country	Region	Series	Years	Inclusion	Type
		NHFPC, Death Registration Data	1990-2011	0	Sample Vital Registration
Colombia	Latin America & Caribbean	Demographic and Health Survey	1990	1	Full Birth Histories
		Demographic and Health Survey	1995	1	Full Birth Histories
		Demographic and Health Survey	2000	1	Full Birth Histories
		Demographic and Health Survey	2005	1	Full Birth Histories
		Demographic and Health Survey	2010	1	Full Birth Histories
		Demographic and Health Survey	2015	1	Full Birth Histories
Comoros	Eastern & Southern Africa	Demographic and Health Survey	1996	1	Full Birth Histories
		Census	2003	1	Household Deaths
Congo	West & Central Africa	Demographic and Health Survey	2005	1	Full Birth Histories
		Demographic and Health Survey	2011-2012	1	Full Birth Histories
Cook Islands	East Asia & Pacific	WHO VR (Recalculated)	1980-2010	0	Vital Registration
Costa Rica	Latin America & Caribbean	National Fertility Survey	1976	0	Full Birth Histories
		WHO VR (Recalculated)	1966-2015	1	Vital Registration
Côte d'Ivoire	West & Central Africa	World Fertility Survey	1980-1981	1	Full Birth Histories
		Demographic and Health Survey	1994	1	Full Birth Histories
		Demographic and Health Survey	1998-1999	1	Full Birth Histories
		AIDS Indicator Survey	2005	1	Full Birth Histories
		Demographic and Health Survey	2011-2012	1	Full Birth Histories
Croatia	Eastern Europe & Central Asia	WHO VR (Recalculated)	1982-2017	1	Vital Registration
Cuba	Latin America & Caribbean	WHO VR (Recalculated)	1964-2017	1	Vital Registration
Cyprus	Western Europe	WHO VR (Recalculated)	1980-2016	1	Vital Registration
Czechia	Western Europe	WHO VR (Recalculated)	1982-2017	1	Vital Registration
Democratic Republic of the Congo	West & Central Africa	Demographic and Health Survey	2007	1	Full Birth Histories
		Demographic and Health Survey	2013-2014	1	Full Birth Histories
Denmark	Western Europe	WHO VR (Recalculated)	1951-2015	1	Vital Registration
Djibouti	Eastern & Southern Africa	PAPFAM Family Health Survey	2002	1	Full Birth Histories
		PAPFAM Family Health Survey	2012	1	Full Birth Histories
Dominica	Latin America & Caribbean	WHO VR (Recalculated)	1968-2015	1	Vital Registration
Dominican Republic	Latin America & Caribbean	World Fertility Survey	1975	1	Full Birth Histories
		World Fertility Survey	1980	1	Full Birth Histories
		Demographic and Health Survey	1991	1	Full Birth Histories
		Demographic and Health Survey	1996	1	Full Birth Histories
		Demographic and Health Survey	1999	0	Full Birth Histories
		Demographic and Health Survey	2002	1	Full Birth Histories
		Demographic and Health Survey	2007	1	Full Birth Histories
		Demographic and Health Survey	2013	1	Full Birth Histories
		Multiple Indicator Cluster Survey	2014	1	Full Birth Histories
Ecuador	Latin America & Caribbean	National Fertility Survey	1979-1980	1	Full Birth Histories
		Demographic and Family Health Survey	1987	1	Full Birth Histories
		Demographic and Maternal and Child Health Survey	1994	1	Full Birth Histories
		Demographic and Maternal and Child Health Survey	1999	1	Full Birth Histories
		Demographic and Maternal and Child Health Survey	2004	1	Full Birth Histories
Egypt	Middle East & North Africa	World Fertility Survey	1980	1	Full Birth Histories
		Demographic and Health Survey	1988	1	Full Birth Histories
		PAPCHILD Maternal and Child Health Survey	1991	1	Full Birth Histories

Country	Region	Series	Years	Inclusion	Type
		Demographic and Health Survey	1992	1	Full Birth Histories
		Demographic and Health Survey	1995	1	Full Birth Histories
		Demographic and Health Survey	2000	1	Full Birth Histories
		Demographic and Health Survey	2003	1	Full Birth Histories
		Demographic and Health Survey	2005	1	Full Birth Histories
		Demographic and Health Survey	2008	1	Full Birth Histories
		Demographic and Health Survey	2014	1	Full Birth Histories
		WHO VR (Recalculated)	1970-2017	1	Vital Registration
El Salvador	Latin America & Caribbean	National Family Health Survey	1993	1	Full Birth Histories
		National Family Health Survey	1998	1	Full Birth Histories
		National Family Health Survey	2002-2003	1	Full Birth Histories
		National Family Health Survey	2008	1	Full Birth Histories
		National Health Survey	2014	1	Full Birth Histories
Equatorial Guinea	West & Central Africa	Census	1994	0	Household Deaths
Eritrea	Eastern & Southern Africa	Demographic and Health Survey	1995-1996	1	Full Birth Histories
		Demographic and Health Survey	2002	1	Full Birth Histories
Estonia	Western Europe	WHO VR (Recalculated)	1980-2017	1	Vital Registration
Ethiopia	Eastern & Southern Africa	Demographic and Health Survey	2000	1	Full Birth Histories
		Demographic and Health Survey	2005	1	Full Birth Histories
		Demographic and Health Survey	2011	1	Full Birth Histories
Fiji	East Asia & Pacific	World Fertility Survey	1974	1	Full Birth Histories
		WHO VR (Recalculated)	2002-2013	1	Vital Registration
Finland	Western Europe	WHO VR (Recalculated)	1952-2017	1	Vital Registration
France	Western Europe	WHO VR (Recalculated)	1950-2015	1	Vital Registration
Gabon	West & Central Africa	Demographic and Health Survey	2000	1	Full Birth Histories
		Demographic and Health Survey	2012	1	Full Birth Histories
Gambia	West & Central Africa	Demographic and Health Survey	2013	0	Full Birth Histories
Georgia	Eastern Europe & Central Asia	Reproductive Health Survey	1999-2000	1	Full Birth Histories
		Reproductive Health Survey	2005	1	Full Birth Histories
		Reproductive Health Survey	2010	1	Full Birth Histories
		WHO VR (Recalculated)	2010-2016	1	Vital Registration
Germany	Western Europe	WHO VR (Recalculated)	1969-2016	1	Vital Registration
Ghana	West & Central Africa	Ghana Fertility Survey	1979-1980	0	Full Birth Histories
		Demographic and Health Survey	1988	1	Full Birth Histories
		Demographic and Health Survey	1993-1994	1	Full Birth Histories
		Demographic and Health Survey	1998-1999	1	Full Birth Histories
		Demographic and Health Survey	2003	1	Full Birth Histories
		Demographic and Health Survey	2008	1	Full Birth Histories
		Multiple Indicator Cluster Survey	2011	1	Full Birth Histories
		Demographic and Health Survey	2014	1	Full Birth Histories
Greece	Western Europe	WHO VR (Recalculated)	1956-2014	1	Vital Registration
Grenada	Latin America & Caribbean	WHO VR (Recalculated)	1988-2016	0	Vital Registration
Guatemala	Latin America & Caribbean	Demographic and Health Survey	1987	1	Full Birth Histories
		Demographic and Health Survey	1995	1	Full Birth Histories
		Demographic and Health Survey	1998-1999	1	Full Birth Histories
		Encuesta Nacional de Salud Materno Infantil	2002	1	Full Birth Histories
		Encuesta Nacional de Salud Materno Infantil	2008-2009	1	Full Birth Histories

Country	Region	Series	Years	Inclusion	Type
		Demographic and Health Survey	2014-2015	1	Full Birth Histories
		WHO VR (Recalculated)	2000-2015	1	Vital Registration
Guinea	West & Central Africa	Census	1983	0	Household Deaths
		Demographic and Health Survey	1999	1	Full Birth Histories
		Demographic and Health Survey	2005	1	Full Birth Histories
		Demographic and Health Survey	2012	1	Full Birth Histories
		Census	2014	1	Household Deaths
Guinea-Bissau	West & Central Africa	Multiple Indicator Cluster Survey	2010	1	Full Birth Histories
		Multiple Indicator Cluster Survey	2014	1	Full Birth Histories
Guyana	Latin America & Caribbean	World Fertility Survey	1975	1	Full Birth Histories
		AIDS Indicator Survey	2005	1	Full Birth Histories
		Demographic and Health Survey	2009	1	Full Birth Histories
		Multiple Indicator Cluster Survey	2014	1	Full Birth Histories
Haiti	Latin America & Caribbean	World Fertility Survey	1977	1	Full Birth Histories
		Demographic and Health Survey	1994-1995	1	Full Birth Histories
		Demographic and Health Survey	2000	1	Full Birth Histories
		Demographic and Health Survey	2005-2006	1	Full Birth Histories
		Demographic and Health Survey	2012	1	Full Birth Histories
Honduras	Latin America & Caribbean	National Survey of Epidemiology and Family Health	1996	1	Full Birth Histories
		National Survey of Epidemiology and Family Health	2001	1	Full Birth Histories
		Demographic and Health Survey	2005-2006	1	Full Birth Histories
		Demographic and Health Survey	2011-2012	1	Full Birth Histories
Hungary	Western Europe	WHO VR (Recalculated)	1955-2015	1	Vital Registration
Iceland	Western Europe	WHO VR (Recalculated)	1952-2017	1	Vital Registration
India	South Asia	National Family Health Survey	1992-1993	1	Full Birth Histories
		National Family Health Survey	1998-1999	1	Full Birth Histories
		National Family Health Survey	2005-2006	1	Full Birth Histories
		Sample Registration System	1968-2015	1	Sample Vital Registration
Indonesia	East Asia & Pacific	World Fertility Survey	1976	1	Full Birth Histories
		Demographic and Health Survey	1987	1	Full Birth Histories
		Demographic and Health Survey	1991	1	Full Birth Histories
		Demographic and Health Survey	1994	1	Full Birth Histories
		Demographic and Health Survey	1997	1	Full Birth Histories
		Demographic and Health Survey	2002-2003	1	Full Birth Histories
		Demographic and Health Survey	2007	1	Full Birth Histories
		Demographic and Health Survey	2012	1	Full Birth Histories
Iran (Islamic Republic of)	Middle East & North Africa	WHO VR (Recalculated)	2008-2009	0	Vital Registration
Iraq	Middle East & North Africa	Multiple Indicator Cluster Survey	2006	1	Full Birth Histories
		Multiple Indicator Cluster Survey	2011	1	Full Birth Histories
Ireland	Western Europe	WHO VR (Recalculated)	1950-2015	1	Vital Registration
Israel	Middle East & North Africa	WHO VR (Recalculated)	1975-2015	1	Vital Registration
Italy	Western Europe	WHO VR (Recalculated)	1951-2015	1	Vital Registration
Jamaica	Latin America & Caribbean	Jamaica Fertility Survey	1975-1976	0	Full Birth Histories
Japan	East Asia & Pacific	WHO VR (Recalculated)	1950-2017	1	Vital Registration
Jordan	Middle East & North Africa	World Fertility Survey	1976	1	Full Birth Histories
		Population and Family Health Survey	1990	1	Full Birth Histories

Country	Region	Series	Years	Inclusion	Type
		Population and Family Health Survey	1997	1	Full Birth Histories
		Population and Family Health Survey	2002	1	Full Birth Histories
		Population and Family Health Survey	2007	1	Full Birth Histories
		Population and Family Health Survey	2012	1	Full Birth Histories
Kazakhstan	Eastern Europe & Central Asia	Demographic and Health Survey	1995	1	Full Birth Histories
		Demographic and Health Survey	1999	1	Full Birth Histories
		WHO VR (Recalculated)	2008-2017	1	Vital Registration
Kenya	Eastern & Southern Africa	World Fertility Survey	1977-1978	1	Full Birth Histories
		Demographic and Health Survey	1989	1	Full Birth Histories
		Demographic and Health Survey	1993	1	Full Birth Histories
		Demographic and Health Survey	1998	1	Full Birth Histories
		Demographic and Health Survey	2003	1	Full Birth Histories
		Demographic and Health Survey	2008-2009	1	Full Birth Histories
		Demographic and Health Survey	2014	1	Full Birth Histories
Kuwait	Middle East & North Africa	WHO VR (Recalculated)	1975-2015	1	Vital Registration
Kyrgyzstan	Eastern Europe & Central Asia	Demographic and Health Survey	1997	1	Full Birth Histories
		Demographic and Health Survey	2012	1	Full Birth Histories
		Multiple Indicator Cluster Survey	2014	1	Full Birth Histories
Lao People's Democratic Republic	East Asia & Pacific	Lao Social Indicator Survey (combined MICS4/DHS)	2012	0	Full Birth Histories
Latvia	Western Europe	WHO VR (Recalculated)	1980-2017	1	Vital Registration
Lebanon	Middle East & North Africa	PAPCHILD Maternal and Child Health Survey	1996	1	Full Birth Histories
		PAPFAM Family Health Survey	2004	1	Full Birth Histories
Lesotho	Eastern & Southern Africa	World Fertility Survey	1977	1	Full Birth Histories
		Demographic and Health Survey	2004	1	Full Birth Histories
		Demographic and Health Survey	2009	1	Full Birth Histories
		Demographic and Health Survey	2014	1	Full Birth Histories
Liberia	West & Central Africa	Demographic and Health Survey	1986	1	Full Birth Histories
		Demographic and Health Survey	2006-2007	1	Full Birth Histories
		Malaria Indicator Survey	2008-2009	1	Full Birth Histories
		Demographic and Health Survey	2013	1	Full Birth Histories
Libya	Middle East & North Africa	PAPCHILD Maternal and Child Health Survey	1995	0	Full Birth Histories
		PAPFAM Family Health Survey	2007	0	Full Birth Histories
		WHO VR (Recalculated)	2009-2011	0	Vital Registration
Lithuania	Western Europe	WHO VR (Recalculated)	1968-2017	1	Vital Registration
Luxembourg	Western Europe	WHO VR (Recalculated)	1963-2016	1	Vital Registration
Madagascar	Eastern & Southern Africa	Demographic and Health Survey	1992	1	Full Birth Histories
		Demographic and Health Survey	1997	1	Full Birth Histories
		Demographic and Health Survey	2003-2004	1	Full Birth Histories
		Demographic and Health Survey	2008-2009	1	Full Birth Histories
Malawi	Eastern & Southern Africa	Demographic and Health Survey	1992	1	Full Birth Histories
		Demographic and Health Survey	2000	1	Full Birth Histories
		Demographic and Health Survey	2004	1	Full Birth Histories
		MDG Endline Survey	2006	1	Full Birth Histories
		Demographic and Health Survey	2010	1	Full Birth Histories
		MDG Endline Survey	2013-2014	1	Full Birth Histories
		Demographic and Health Survey	2015-2016	1	Full Birth Histories
Malaysia	East Asia & Pacific	Fertility and Family Survey	1974	1	Full Birth Histories

Country	Region	Series	Years	Inclusion	Type
		WHO VR (Recalculated)	1990-2016	1	Vital Registration
Maldives	South Asia	Demographic and Health Survey	2009	1	Full Birth Histories
		WHO VR (Recalculated)	2008-2013	1	Vital Registration
Mali	West & Central Africa	Demographic and Health Survey	1987	1	Full Birth Histories
		Demographic and Health Survey	1995-1996	1	Full Birth Histories
		Demographic and Health Survey	2001	1	Full Birth Histories
		Demographic and Health Survey	2006	1	Full Birth Histories
		Demographic and Health Survey	2012-2013	0	Full Birth Histories
Malta	Western Europe	WHO VR (Recalculated)	1955-2017	1	Vital Registration
Mauritania	West & Central Africa	World Fertility Survey	1981-1982	1	Full Birth Histories
		PAPCHILD Maternal and Child Health Survey	1990	1	Full Birth Histories
		Demographic and Health Survey	2000-2001	1	Full Birth Histories
		Census	2013	0	Household Deaths
Mauritius	Eastern & Southern Africa	WHO VR (Recalculated)	1957-2017	1	Vital Registration
Mexico	Latin America & Caribbean	Demographic and Health Survey	1987	1	Full Birth Histories
		Encuesta Nacional de la Dinamica Demografica (ENADID)	2009	1	Full Birth Histories
		Encuesta Nacional de la Dinamica Demografica (ENADID)	2014	1	Full Birth Histories
		WHO VR (Recalculated)	2005-2016	1	Vital Registration
Mongolia	East Asia & Pacific	Reproductive Health Survey	2002	0	Full Birth Histories
		Social Indicator Sample Survey (SISS)	2013-2014	0	Full Birth Histories
		WHO VR (Recalculated)	2005-2017	0	Vital Registration
Montenegro	Eastern Europe & Central Asia	WHO VR (Recalculated)	1985-2017	1	Vital Registration
Morocco	Middle East & North Africa	World Fertility Survey	1980	1	Full Birth Histories
		Demographic and Health Survey	1987	1	Full Birth Histories
		Demographic and Health Survey	1992	1	Full Birth Histories
		Demographic and Health Survey	1995	1	Full Birth Histories
		PAPCHILD Maternal and Child Health Survey	1997	1	Full Birth Histories
		Demographic and Health Survey	2003-2004	1	Full Birth Histories
Mozambique	Eastern & Southern Africa	Demographic and Health Survey	1997	0	Full Birth Histories
		Demographic and Health Survey	2003-2004	1	Full Birth Histories
		Multiple Indicator Cluster Survey	2008	1	Full Birth Histories
		Demographic and Health Survey	2011	1	Full Birth Histories
Myanmar	East Asia & Pacific	Demographic and Health Survey	2015-2016	0	Full Birth Histories
Namibia	Eastern & Southern Africa	Demographic and Health Survey	1992	1	Full Birth Histories
		Demographic and Health Survey	2000	1	Full Birth Histories
		Census	2001	0	Household Deaths
		Demographic and Health Survey	2006-2007	1	Full Birth Histories
		Demographic and Health Survey	2013	1	Full Birth Histories
Nepal	South Asia	World Fertility Survey	1976	1	Full Birth Histories
		Fertility and Family Planning Survey	1991	1	Full Birth Histories
		Demographic and Health Survey	1996	1	Full Birth Histories
		Demographic and Health Survey	2001	1	Full Birth Histories
		Demographic and Health Survey	2006	1	Full Birth Histories
		Demographic and Health Survey	2011	1	Full Birth Histories
		Multiple Indicator Cluster Survey	2014	1	Full Birth Histories
Netherlands	Western Europe	WHO VR (Recalculated)	1950-2016	1	Vital Registration

Country	Region	Series	Years	Inclusion	Type
New Zealand	East Asia & Pacific	WHO VR (Recalculated)	1950-2014	1	Vital Registration
Nicaragua	Latin America & Caribbean	Demographic and Health Survey	1998	1	Full Birth Histories
		Demographic and Health Survey	2001	1	Full Birth Histories
		Encuesta Nicaraguense de Demografia y Salud	2006-2007	1	Full Birth Histories
		Encuesta Nicaraguense de Demografia y Salud	2012	1	Full Birth Histories
Niger	West & Central Africa	Demographic and Health Survey	1992	1	Full Birth Histories
		Demographic and Health Survey	1998	1	Full Birth Histories
		Demographic and Health Survey	2006	1	Full Birth Histories
		Demographic and Health Survey	2012	1	Full Birth Histories
Nigeria	West & Central Africa	World Fertility Survey	1981-1982	0	Full Birth Histories
		Demographic and Health Survey	1990	1	Full Birth Histories
		Demographic and Health Survey	1999	0	Full Birth Histories
		Demographic and Health Survey	2003	1	Full Birth Histories
		Demographic and Health Survey	2008	1	Full Birth Histories
		Malaria Indicator Survey	2010	1	Full Birth Histories
		Demographic and Health Survey	2013	1	Full Birth Histories
Niue	East Asia & Pacific	WHO VR (Recalculated)	1980-2010	0	Vital Registration
Norway	Western Europe	WHO VR (Recalculated)	1951-2016	1	Vital Registration
Oman	Middle East & North Africa	WHO VR (Recalculated)	2009-2017	0	Vital Registration
Pakistan	South Asia	World Fertility Survey	1975	1	Full Birth Histories
		Demographic and Health Survey	1990-1991	1	Full Birth Histories
		Demographic and Health Survey	2006-2007	1	Full Birth Histories
		Demographic and Health Survey	2012-2013	1	Full Birth Histories
		Pakistan Demographic Survey	1984-2004	1	Sample Vital Registration
		Pakistan Demographic Survey	2005-2008	1	Household Deaths
Palau	East Asia & Pacific	WHO VR (Recalculated)	1985-2007	0	Vital Registration
Panama	Latin America & Caribbean	World Fertility Survey	1975-1976	1	Full Birth Histories
		WHO VR (Recalculated)	2010-2016	1	Vital Registration
Paraguay	Latin America & Caribbean	National Fertility Survey	1979	1	Full Birth Histories
		Demographic and Health Survey	1990	1	Full Birth Histories
		National Survey of Demography and Reproductive Health	1995-1996	1	Full Birth Histories
		Reproductive Health Survey	2004	1	Full Birth Histories
		ENDSSR	2008	1	Full Birth Histories
Peru	Latin America & Caribbean	World Fertility Survey	1977-1978	1	Full Birth Histories
		Demographic and Health Survey	1991-1992	1	Full Birth Histories
		Demographic and Health Survey	1996	1	Full Birth Histories
		Demographic and Health Survey	2000	1	Full Birth Histories
		Demographic and Health Survey	2004-2008	1	Full Birth Histories
		Demographic and Health Survey	2009	1	Full Birth Histories
		Demographic and Health Survey	2010	1	Full Birth Histories
		Demographic and Health Survey	2011	1	Full Birth Histories
		Demographic and Health Survey	2012	1	Full Birth Histories
		Demographic and Health Survey	2013	1	Full Birth Histories
		Demographic and Health Survey	2014	1	Full Birth Histories
Philippines	East Asia & Pacific	World Fertility Survey	1978	1	Full Birth Histories
		National Demographic and Health Survey	1993	1	Full Birth Histories
		National Demographic and Health Survey	1998	1	Full Birth Histories

Country	Region	Series	Years	Inclusion	Type
		National Demographic and Health Survey	2003	1	Full Birth Histories
		National Demographic and Health Survey	2008	1	Full Birth Histories
		National Demographic and Health Survey	2013	1	Full Birth Histories
Poland	Western Europe	WHO VR (Recalculated)	1959-2017	1	Vital Registration
Portugal	Western Europe	World Fertility Survey	1979-1980	0	Full Birth Histories
		WHO VR (Recalculated)	1955-2016	1	Vital Registration
Qatar	Middle East & North Africa	WHO VR (Recalculated)	1989-2016	1	Vital Registration
Republic of Korea	East Asia & Pacific	Korea Fertility Survey	1974	1	Full Birth Histories
		WHO VR (Recalculated)	1980-2016	1	Vital Registration
Republic of Moldova	Eastern Europe & Central Asia	Demographic and Health Survey	2005	1	Full Birth Histories
		Multiple Indicator Cluster Survey	2012	1	Full Birth Histories
		WHO VR (Recalculated)	1990-2016	1	Vital Registration
Romania	Eastern Europe & Central Asia	WHO VR (Recalculated)	1963-2016	1	Vital Registration
Russian Federation	Eastern Europe & Central Asia	WHO VR (Recalculated)	1980-2015	1	Vital Registration
Rwanda	Eastern & Southern Africa	World Fertility Survey	1983	1	Full Birth Histories
		Demographic and Health Survey	1992	1	Full Birth Histories
		Demographic and Health Survey	2000	1	Full Birth Histories
		Demographic and Health Survey	2005	1	Full Birth Histories
		Demographic and Health Survey	2010	1	Full Birth Histories
		Demographic and Health Survey	2014-2015	1	Full Birth Histories
Saint Kitts and Nevis	Latin America & Caribbean	WHO VR (Recalculated)	1984-2013	0	Vital Registration
Saint Lucia	Latin America & Caribbean	WHO VR (Recalculated)	1972-2015	1	Vital Registration
Saint Vincent and the Grenadines	Latin America & Caribbean	WHO VR (Recalculated)	1972-2016	1	Vital Registration
Samoa	East Asia & Pacific	Demographic and Health Survey	1999	0	Household Deaths
		Population and Housing Census	2011	0	Household Deaths
San Marino	Western Europe	WHO VR (Recalculated)	1980-2008	0	Vital Registration
Sao Tome and Principe	West & Central Africa	Demographic and Health Survey	2008-2009	1	Full Birth Histories
		Multiple Indicator Cluster Survey	2014	1	Full Birth Histories
		WHO VR (Recalculated)	1985-1988	1	Vital Registration
Saudi Arabia	Middle East & North Africa	Census	2004	0	Household Deaths
Senegal	West & Central Africa	World Fertility Survey	1978	1	Full Birth Histories
		Demographic and Health Survey	1986	1	Full Birth Histories
		Demographic and Health Survey	1992-1993	1	Full Birth Histories
		Demographic and Health Survey	1997	1	Full Birth Histories
		Demographic and Health Survey	1999-2000	1	Full Birth Histories
		Demographic and Health Survey	2005	1	Full Birth Histories
		Malaria Indicator Survey	2008-2009	1	Full Birth Histories
		Demographic and Health Survey	2010-2011	1	Full Birth Histories
		Demographic and Health Survey	2012-2013	1	Full Birth Histories
		Demographic and Health Survey	2014	1	Full Birth Histories
		Demographic and Health Survey	2015	1	Full Birth Histories
Serbia	Eastern Europe & Central Asia	WHO VR (Recalculated)	1985-2016	1	Vital Registration
Seychelles	Eastern & Southern Africa	WHO VR (Recalculated)	1968-2011	0	Vital Registration
Sierra Leone	West & Central Africa	Census	2004	0	Household Deaths
		Demographic and Health Survey	2008	0	Full Birth Histories
		Demographic and Health Survey	2013	0	Full Birth Histories

Country	Region	Series	Years	Inclusion	Type
Singapore	East Asia & Pacific	WHO VR (Recalculated)	1955-2017	1	Vital Registration
Slovakia	Western Europe	WHO VR (Recalculated)	1982-2017	1	Vital Registration
Slovenia	Western Europe	WHO VR (Recalculated)	1982-2016	1	Vital Registration
Somalia	Eastern & Southern Africa	Multiple Indicator Cluster Survey	2006	0	Full Birth Histories
South Africa	Eastern & Southern Africa	Demographic and Health Survey	1998	1	Full Birth Histories
		Census	2001	1	Household Deaths
		Community Survey	2007	0	Household Deaths
		WHO VR (Recalculated)	2005-2016	1	Vital Registration
Spain	Western Europe	WHO VR (Recalculated)	1951-2015	1	Vital Registration
Sri Lanka	South Asia	World Fertility Survey	1975	1	Full Birth Histories
		Demographic and Health Survey	1987	1	Full Birth Histories
		WHO VR (Recalculated)	1983-2014	1	Vital Registration
State of Palestine	Middle East & North Africa	Multiple Indicator Cluster Survey	2010	1	Full Birth Histories
		Multiple Indicator Cluster Survey	2014	1	Full Birth Histories
Sudan	Eastern & Southern Africa	World Fertility Survey	1978-1979	1	Full Birth Histories
		PAPCHILD Maternal and Child Health Survey	1992	1	Full Birth Histories
		Census	1993	0	Household Deaths
		Multiple Indicator Cluster Survey	2010	1	Full Birth Histories
		Multiple Indicator Cluster Survey	2014	1	Full Birth Histories
Swaziland	Eastern & Southern Africa	Demographic and Health Survey	2006-2007	1	Full Birth Histories
		Multiple Indicator Cluster Survey	2010	1	Full Birth Histories
		Multiple Indicator Cluster Survey	2014	1	Full Birth Histories
Sweden	Western Europe	WHO VR (Recalculated)	1951-2017	1	Vital Registration
Switzerland	Western Europe	WHO VR (Recalculated)	1951-2017	1	Vital Registration
Syrian Arab Republic	Middle East & North Africa	World Fertility Survey	1978	1	Full Birth Histories
		PAPCHILD Maternal and Child Health Survey	1993	1	Full Birth Histories
		PAPFAM Family Health Survey	2001	1	Full Birth Histories
Tajikistan	Eastern Europe & Central Asia	Demographic and Health Survey	2012	1	Full Birth Histories
Thailand	East Asia & Pacific	World Fertility Survey	1975	1	Full Birth Histories
		Demographic and Health Survey	1987	1	Full Birth Histories
		Survey of Population Change	1989	1	Household Deaths
		Survey of Population Change	1996	1	Household Deaths
		Survey of Population Change	2006	1	Household Deaths
The former Yugoslav Republic of Macedonia	Eastern Europe & Central Asia	WHO VR (Recalculated)	1982-2014	1	Vital Registration
Timor-Leste	East Asia & Pacific	Demographic and Health Survey	2009-2010	0	Full Birth Histories
Togo	West & Central Africa	Demographic and Health Survey	1988	1	Full Birth Histories
		Demographic and Health Survey	1998	1	Full Birth Histories
		Demographic and Health Survey	2013-2014	1	Full Birth Histories
Trinidad and Tobago	Latin America & Caribbean	World Fertility Survey	1977	1	Full Birth Histories
		Demographic and Health Survey	1987	1	Full Birth Histories
		WHO VR (Recalculated)	1970-2011	1	Vital Registration
Tunisia	Middle East & North Africa	National Demographic Survey	1968-1969	1	Household Deaths
		World Fertility Survey	1978	0	Full Birth Histories
		Demographic and Health Survey	1988	1	Full Birth Histories
		PAPCHILD Maternal and Child Health Survey	1994	1	Full Birth Histories

Country	Region	Series	Years	Inclusion	Type
		PAPFAM Family Health Survey	2001	1	Full Birth Histories
		Multiple Indicator Cluster Survey	2011-2012	1	Full Birth Histories
Turkey	Eastern Europe & Central Asia	World Fertility Survey	1978	1	Full Birth Histories
		Demographic and Health Survey	1993	1	Full Birth Histories
		Demographic and Health Survey	1998	1	Full Birth Histories
		Demographic and Health Survey	2003-2004	1	Full Birth Histories
		Turkey Demographic and Health Survey	2008	1	Full Birth Histories
		Demographic and Health Survey	2013	1	Full Birth Histories
		WHO VR (Recalculated)	2013-2017	1	Vital Registration
Turkmenistan	Eastern Europe & Central Asia	Demographic and Health Survey	2000	1	Full Birth Histories
		Multiple Indicator Cluster Survey	2016	0	Full Birth Histories
		WHO VR (Recalculated)	1981-2016	1	Vital Registration
Tuvalu	East Asia & Pacific	WHO VR (Recalculated)	1991-2006	0	Vital Registration
Uganda	Eastern & Southern Africa	Demographic and Health Survey	1988-1989	1	Full Birth Histories
		Demographic and Health Survey	1995	1	Full Birth Histories
		Demographic and Health Survey	2000-2001	1	Full Birth Histories
		Demographic and Health Survey	2006	1	Full Birth Histories
		Demographic and Health Survey	2009-2010	1	Full Birth Histories
		Demographic and Health Survey	2011	1	Full Birth Histories
Ukraine	Eastern Europe & Central Asia	Reproductive Health Survey	1999	1	Full Birth Histories
		Demographic and Health Survey	2007	1	Full Birth Histories
		WHO VR (Recalculated)	1990-2017	1	Vital Registration
United Arab Emirates	Middle East & North Africa	WHO VR (Recalculated)	2003-2013	1	Vital Registration
United Kingdom	Western Europe	WHO VR (Recalculated)	1950-2016	1	Vital Registration
United Republic of Tanzania	Eastern & Southern Africa	Demographic and Health Survey	1991-1992	1	Full Birth Histories
		Demographic and Health Survey	1996	1	Full Birth Histories
		Demographic and Health Survey	1999	1	Full Birth Histories
		Demographic and Health Survey	2004-2005	1	Full Birth Histories
		AIDS Indicator Survey	2007-2008	1	Full Birth Histories
		Demographic and Health Survey	2010	1	Full Birth Histories
		Demographic and Health Survey	2015-2016	1	Full Birth Histories
United States	North America	WHO VR (Recalculated)	1950-2016	1	Vital Registration
Uruguay	Latin America & Caribbean	WHO VR (Recalculated)	1955-2017	1	Vital Registration
Uzbekistan	Eastern Europe & Central Asia	Demographic and Health Survey	1996	0	Full Birth Histories
		Demographic and Health Survey	2002	1	Full Birth Histories
		WHO VR (Recalculated)	1981-2017	1	Vital Registration
Venezuela (Bolivarian Republic of)	Latin America & Caribbean	World Fertility Survey	1977	1	Full Birth Histories
		WHO VR (Recalculated)	1990-2014	1	Vital Registration
Viet Nam	East Asia & Pacific	National Demographic and Health Survey	1988	1	Full Birth Histories
		Demographic and Health Survey	1997	1	Full Birth Histories
		Demographic and Health Survey	2002	1	Full Birth Histories
		Population Change and Family Planning Survey	2013	1	Household Deaths
		Multiple Indicator Cluster Survey	2013-2014	1	Full Birth Histories
Yemen	Middle East & North Africa	World Fertility Survey	1979	1	Full Birth Histories
		Demographic and Health Survey	1991-1992	1	Full Birth Histories
		Demographic and Health Survey	1997	1	Full Birth Histories
		PAPFAM Family Health Survey	2003	1	Full Birth Histories

Country	Region	Series	Years	Inclusion	Type
		Multiple Indicator Cluster Survey	2006	1	Full Birth Histories
		Demographic and Health Survey	2013	1	Full Birth Histories
Zambia	Eastern & Southern Africa	Demographic and Health Survey	1992	1	Full Birth Histories
		Demographic and Health Survey	1996-1997	1	Full Birth Histories
		Demographic and Health Survey	2001-2002	1	Full Birth Histories
		Demographic and Health Survey	2007	1	Full Birth Histories
		Demographic and Health Survey	2013-2014	1	Full Birth Histories
Zimbabwe	Eastern & Southern Africa	Demographic and Health Survey	1988-1989	1	Full Birth Histories
		Demographic and Health Survey	1994	1	Full Birth Histories
		Demographic and Health Survey	1999	1	Full Birth Histories
		Demographic and Health Survey	2005-2006	0	Full Birth Histories
		Multiple Indicator Cluster Survey	2009	1	Full Birth Histories
		Demographic and Health Survey	2010-2011	1	Full Birth Histories
		Multiple Indicator Cluster Survey	2014	1	Full Birth Histories
		Demographic and Health Survey	2015	1	Full Birth Histories

5 Mortality levels and trends

Table S8 presents the estimates of the probability of dying among children aged 5-14 (deaths per 1,000 children aged 5), and Table S9 presents the estimates of the probability of dying among children aged 5-9 (deaths per 1,000 children aged 5). Tables S10 and S11 present the numbers of deaths among children aged 5-14 and 5-9 in 1990 and 2016. Estimates can be downloaded from www.childmortality.org.

Table S8 – *Probability of dying among children aged 5-14 (deaths per 1,000 children aged 5)*

Country	1990			2016		
	$_{10}q_5$	Lower bound	Upper bound	$_{10}q_5$	Lower bound	Upper bound
Afghanistan	20.6	12.9	29.5	9.5	6.3	15.1
Albania	5.5	4.3	7.1	2.1	1.7	2.7
Algeria	8.9	8.4	9.5	4.1	3.8	4.4
Andorra	1.8	1.4	2.4	0.8	0.7	1.1
Angola	46.2	36.1	58.9	15.4	12.0	19.6
Antigua and Barbuda	4.5	3.6	5.8	1.8	1.4	2.3
Argentina	3.4	3.3	3.5	2.1	2.0	2.2
Armenia	4.2	3.0	6.0	2.2	1.9	2.5
Australia	1.9	1.8	2.0	0.9	0.8	1.0
Austria	1.8	1.7	1.9	0.9	0.8	1.0
Azerbaijan	5.3	5.2	5.4	2.9	2.7	3.1
Bahamas	3.9	3.6	4.3	2.8	2.1	3.6
Bahrain	3.6	2.7	4.5	1.8	1.4	2.1
Bangladesh	25.4	22.8	28.2	4.9	4.0	6.1
Barbados	2.9	2.5	3.3	2.0	1.4	2.8
Belarus	3.9	3.8	4.0	1.5	1.4	1.6
Belgium	2.1	2.0	2.1	0.9	0.8	0.9
Belize	5.3	3.7	7.5	2.9	2.4	3.4
Benin	45.5	40.0	51.7	22.4	14.4	36.3
Bhutan	18.1	13.4	24.9	7.1	5.2	9.8
Bolivia (Plurinational State of)	13.5	11.6	15.5	6.4	3.8	12.1
Bosnia and Herzegovina	2.7	2.5	2.8	1.2	1.0	1.4
Botswana	10.4	8.1	13.3	7.8	6.1	10.1
Brazil	4.7	3.9	5.6	2.5	2.4	2.6
Brunei Darussalam	3.8	3.5	4.2	2.3	1.8	2.8
Bulgaria	4.0	3.9	4.1	1.7	1.5	1.9
Burkina Faso	40.6	35.4	46.3	25.5	15.5	38.7
Burundi	57.4	40.3	80.6	20.2	9.7	38.5
Cabo Verde	6.0	5.5	6.6	2.4	1.9	3.0
Cambodia	36.4	29.7	44.2	5.3	3.3	7.9
Cameroon	37.4	32.7	42.7	29.6	20.5	42.6
Canada	2.1	2.0	2.1	0.8	0.7	1.0
Central African Republic	35.6	27.8	45.4	22.2	17.3	28.2
Chad	54.7	46.8	63.9	26.4	19.2	36.3

Country	1990			2016		
	$_{10}q_5$	Lower bound	Upper bound	$_{10}q_5$	Lower bound	Upper bound
Chile	3.1	3.1	3.2	1.5	1.4	1.7
China	6.7	5.6	7.9	2.8	1.9	4.3
Colombia	5.1	4.2	6.2	3.0	1.9	4.3
Comoros	17.2	12.5	23.3	6.2	3.6	10.7
Congo	37.0	21.3	63.6	11.2	6.6	18.9
Cook Islands	5.2	4.1	6.7	2.1	1.6	2.7
Costa Rica	2.9	2.8	3.1	1.8	1.5	2.0
Côte d'Ivoire	31.0	26.3	36.1	27.9	18.7	45.0
Croatia	2.9	2.8	3.1	1.1	1.0	1.3
Cuba	3.9	3.8	4.0	2.1	2.0	2.3
Cyprus	2.0	1.8	2.1	0.9	0.7	1.2
Czechia	2.4	2.3	2.5	0.9	0.8	1.0
Democratic People's Republic of Korea	8.2	6.4	10.5	3.9	3.0	5.0
Democratic Republic of the Congo	44.2	25.7	77.6	28.2	18.9	41.7
Denmark	1.9	1.8	2.0	0.5	0.4	0.6
Djibouti	16.7	7.8	38.1	2.0	0.6	5.9
Dominica	3.3	2.7	4.2	2.4	1.5	3.8
Dominican Republic	7.8	6.6	9.2	3.1	2.2	4.5
Ecuador	7.8	6.7	9.1	3.4	1.8	6.5
Egypt	10.9	10.7	11.1	4.9	4.7	5.0
El Salvador	7.0	5.7	8.6	3.7	2.2	7.1
Equatorial Guinea	39.7	31.0	50.9	16.8	13.2	21.6
Eritrea	48.3	40.6	57.9	12.5	6.6	23.5
Estonia	4.7	4.5	5.0	1.2	1.0	1.5
Ethiopia	81.4	68.2	96.8	17.4	9.6	26.8
Fiji	9.7	6.9	13.8	4.1	3.3	5.0
Finland	1.8	1.7	1.9	0.8	0.7	0.9
France	2.0	1.9	2.0	0.7	0.7	0.8
Gabon	21.5	15.4	30.1	15.7	9.2	27.8
Gambia	34.8	27.3	44.7	12.5	9.8	16.1
Georgia	7.2	4.3	12.0	2.8	2.5	3.1
Germany	1.9	1.9	2.0	0.8	0.7	0.8
Ghana	28.9	25.4	33.1	12.6	8.7	17.3
Greece	1.9	1.8	2.0	0.9	0.8	1.1
Grenada	4.0	3.1	5.2	2.8	2.1	3.5
Guatemala	12.2	10.6	14.0	3.7	3.5	4.0
Guinea	52.1	43.7	62.5	22.3	17.3	29.0
Guinea-Bissau	34.3	10.1	89.3	17.6	10.8	27.3
Guyana	11.4	7.8	18.4	6.1	3.1	11.0
Haiti	30.8	26.4	36.2	14.5	9.9	21.2
Honduras	8.8	7.1	11.0	3.9	2.3	6.2
Hungary	2.5	2.4	2.6	1.0	0.9	1.1

Country	1990 $_{10}q_5$	1990 Lower bound	1990 Upper bound	2016 $_{10}q_5$	2016 Lower bound	2016 Upper bound
Iceland	1.7	1.5	2.0	0.9	0.6	1.3
India	20.9	19.9	21.9	6.3	5.6	7.0
Indonesia	15.4	14.0	17.1	5.3	3.7	7.8
Iran (Islamic Republic of)	8.8	6.8	11.2	2.7	2.1	3.5
Iraq	8.1	4.6	16.4	5.6	3.5	9.4
Ireland	1.9	1.8	2.0	0.8	0.7	0.9
Israel	2.0	1.9	2.0	0.9	0.8	1.0
Italy	1.8	1.8	1.9	0.7	0.6	0.8
Jamaica	5.1	4.0	6.5	2.7	2.1	3.4
Japan	1.7	1.6	1.7	0.8	0.8	0.8
Jordan	5.0	4.0	6.1	4.4	2.5	8.2
Kazakhstan	5.7	4.1	8.1	3.0	2.8	3.1
Kenya	18.2	15.9	21.1	10.6	7.0	15.8
Kiribati	16.5	12.9	21.1	8.6	6.7	11.1
Kuwait	3.7	3.5	3.8	1.8	1.5	2.0
Kyrgyzstan	8.7	5.7	13.6	2.7	1.6	5.3
Lao People's Democratic Republic	27.1	21.3	34.8	9.9	7.8	12.7
Latvia	5.5	5.3	5.8	1.5	1.3	1.8
Lebanon	6.7	4.8	9.3	1.3	0.4	3.9
Lesotho	14.1	9.3	19.3	13.8	9.1	22.2
Liberia	33.9	26.2	44.1	17.3	10.2	26.4
Libya	8.5	6.9	10.6	4.9	4.0	6.1
Lithuania	4.3	4.1	4.5	1.7	1.5	1.9
Luxembourg	1.8	1.5	2.1	0.5	0.3	0.7
Madagascar	44.3	38.8	51.8	13.4	8.1	21.5
Malawi	41.6	36.7	46.9	13.6	10.6	17.5
Malaysia	5.0	4.8	5.1	2.6	2.5	2.8
Maldives	12.6	5.5	28.6	2.5	1.9	3.4
Mali	46.7	41.4	52.4	23.5	14.1	37.5
Malta	1.6	1.4	1.8	0.9	0.6	1.3
Marshall Islands	9.3	7.3	12.0	6.0	4.7	7.8
Mauritania	21.7	18.0	26.0	9.3	5.0	18.7
Mauritius	3.7	3.5	3.9	1.8	1.6	2.1
Mexico	5.5	4.3	6.9	2.5	2.4	2.6
Micronesia (Federated States of)	10.0	7.8	12.7	5.8	4.5	7.4
Monaco	1.7	1.3	2.2	0.9	0.7	1.2
Mongolia	18.5	14.4	23.7	3.6	2.8	4.6
Montenegro	2.4	2.2	2.6	1.2	0.9	1.5
Morocco	10.4	9.1	11.9	3.4	1.9	5.8
Mozambique	68.4	50.5	92.6	15.4	9.6	22.7
Myanmar	19.6	15.4	25.1	8.2	6.4	10.4
Namibia	16.9	13.9	20.4	10.6	6.8	16.5
Nauru	10.5	8.2	13.5	5.9	4.6	7.6

Country	1990 $_{10}q_5$	1990 Lower bound	1990 Upper bound	2016 $_{10}q_5$	2016 Lower bound	2016 Upper bound
Nepal	28.9	25.9	32.4	5.0	3.2	7.2
Netherlands	1.8	1.8	1.9	0.8	0.7	0.9
New Zealand	2.5	2.4	2.7	1.0	0.8	1.1
Nicaragua	8.1	6.6	10.0	3.8	2.1	6.7
Niger	70.8	62.8	80.3	40.3	27.8	57.9
Nigeria	42.3	35.7	49.4	20.6	14.3	28.7
Niue	3.4	2.7	4.4	4.2	3.3	5.4
Norway	1.8	1.7	1.9	0.6	0.5	0.8
Oman	6.4	5.0	8.1	2.1	1.7	2.7
Pakistan	13.6	12.6	14.6	11.3	6.7	21.3
Palau	7.1	5.5	9.1	3.3	2.6	4.3
Panama	6.2	3.8	10.2	3.0	2.7	3.3
Papua New Guinea	15.2	11.9	19.4	8.6	6.8	11.0
Paraguay	7.0	5.6	8.8	3.6	1.8	7.5
Peru	10.9	9.7	12.3	3.5	2.7	5.1
Philippines	14.2	12.6	16.2	6.5	4.4	9.5
Poland	2.8	2.7	2.8	1.0	1.0	1.1
Portugal	3.9	3.7	4.0	0.8	0.7	1.0
Qatar	3.8	3.4	4.3	1.8	1.5	2.1
Republic of Korea	5.3	5.2	5.4	0.8	0.8	0.9
Republic of Moldova	5.3	5.0	5.6	2.2	1.9	2.4
Romania	5.3	5.2	5.4	1.8	1.7	1.9
Russian Federation	5.1	5.0	5.3	2.4	2.2	2.7
Rwanda	67.0	56.5	78.8	12.0	7.3	19.5
Saint Kitts and Nevis	5.3	4.1	6.8	1.9	1.5	2.5
Saint Lucia	4.1	3.6	4.6	2.4	1.7	3.4
Saint Vincent and the Grenadines	3.8	3.4	4.4	3.7	2.8	5.0
Samoa	6.3	4.9	8.0	3.5	2.7	4.5
San Marino	2.1	1.7	2.7	0.9	0.7	1.1
Sao Tome and Principe	20.3	17.2	24.0	9.4	5.4	16.4
Saudi Arabia	7.1	5.6	9.1	2.4	1.9	3.1
Senegal	37.3	32.7	42.1	16.2	11.1	22.9
Serbia	3.1	3.0	3.2	1.3	1.1	1.5
Seychelles	4.9	3.8	6.2	3.8	3.0	4.8
Sierra Leone	55.2	43.1	70.5	20.8	16.2	26.5
Singapore	2.3	2.2	2.4	0.7	0.6	0.8
Slovakia	2.6	2.5	2.7	1.3	1.1	1.5
Slovenia	2.3	2.1	2.4	0.8	0.6	1.0
Solomon Islands	7.4	5.8	9.5	4.7	3.7	6.0
Somalia	37.7	29.4	48.4	24.1	18.8	30.9
South Africa	10.5	8.0	13.7	5.2	5.0	5.5
South Sudan	54.0	41.9	69.2	16.9	13.1	21.6
Spain	2.3	2.2	2.3	0.8	0.7	0.9

Country	1990 $_{10}q_5$	1990 Lower bound	1990 Upper bound	2016 $_{10}q_5$	2016 Lower bound	2016 Upper bound
Sri Lanka	6.3	6.2	6.4	2.3	2.0	2.5
State of Palestine	6.5	2.5	18.4	3.3	1.9	5.8
Sudan	29.7	24.4	36.8	9.1	6.1	13.1
Suriname	7.2	5.6	9.2	3.2	2.5	4.1
Swaziland	18.5	8.6	39.8	11.4	6.6	19.9
Sweden	1.4	1.4	1.5	0.8	0.7	0.9
Switzerland	2.0	1.9	2.0	0.7	0.6	0.8
Syrian Arab Republic	7.4	6.1	9.1	3.2	1.7	6.4
Tajikistan	17.6	2.7	126.8	3.4	1.5	7.7
Thailand	8.2	6.9	9.7	3.2	2.1	5.0
The former Yugoslav Republic of Macedonia	3.2	3.0	3.3	1.1	0.9	1.4
Timor-Leste	29.5	23.1	37.6	8.0	6.3	10.2
Togo	38.8	33.4	45.4	22.5	15.8	32.2
Tonga	4.8	3.7	6.1	3.4	2.7	4.3
Trinidad and Tobago	3.8	3.6	4.0	2.8	2.1	3.6
Tunisia	7.4	5.9	9.1	3.1	1.6	6.6
Turkey	9.1	7.6	11.0	2.3	2.2	2.4
Turkmenistan	6.5	6.4	6.7	3.7	3.5	4.0
Tuvalu	10.3	8.1	13.3	4.7	3.6	6.0
Uganda	32.8	29.0	37.4	16.1	10.7	23.0
Ukraine	4.5	4.4	4.6	1.8	1.7	1.9
United Arab Emirates	5.5	2.7	27.0	1.3	1.0	1.6
United Kingdom	1.9	1.8	1.9	0.8	0.8	0.9
United Republic of Tanzania	30.8	27.1	35.0	12.2	8.7	17.7
United States	2.4	2.4	2.5	1.3	1.2	1.4
Uruguay	3.3	3.1	3.4	1.7	1.5	1.9
Uzbekistan	6.0	5.9	6.1	3.4	3.3	3.5
Vanuatu	7.0	5.5	8.9	5.0	3.9	6.4
Venezuela (Bolivarian Republic of)	4.5	4.3	4.6	2.6	2.4	2.9
Viet Nam	13.2	10.8	16.1	2.8	2.0	3.7
Yemen	20.8	18.5	23.5	6.1	4.3	8.6
Zambia	30.2	26.6	34.3	13.9	9.0	20.6
Zimbabwe	12.7	10.1	15.6	15.3	10.2	21.3

Table S9 – *Probability of dying among children aged 5-9 (deaths per 1,000 children aged 5)*

Country	1990 $_5q_5$	1990 Lower bound	1990 Upper bound	2016 $_5q_5$	2016 Lower bound	2016 Upper bound
Afghanistan	10.3	3.9	17.5	5.9	3.9	8.4
Albania	2.8	2.2	3.6	1.1	0.8	1.3
Algeria	5.1	5.1	5.2	2.2	2.1	2.2
Andorra	0.9	0.7	1.2	0.4	0.3	0.5

Country	1990 $_5q_5$	1990 Lower bound	1990 Upper bound	2016 $_5q_5$	2016 Lower bound	2016 Upper bound
Angola	33.7	26.3	42.9	11.1	8.7	14.2
Antigua and Barbuda	2.0	1.6	2.6	0.8	0.6	1.0
Argentina	1.7	1.6	1.7	0.9	0.9	0.9
Armenia	2.3	1.8	2.7	1.1	1.0	1.2
Australia	0.9	0.9	0.9	0.4	0.4	0.5
Austria	0.9	0.9	0.9	0.4	0.4	0.4
Azerbaijan	3.2	3.1	3.2	1.6	1.6	1.7
Bahamas	2.1	1.9	2.3	1.5	1.3	1.8
Bahrain	2.1	1.8	2.4	1.0	0.8	1.1
Bangladesh	16.8	16.1	17.5	2.6	2.4	2.8
Barbados	1.4	1.2	1.5	0.8	0.5	1.0
Belarus	2.1	2.0	2.1	0.8	0.7	0.8
Belgium	1.0	1.0	1.1	0.4	0.4	0.4
Belize	2.8	1.9	3.9	1.3	1.1	1.6
Benin	32.3	28.6	36.7	14.4	10.5	18.1
Bhutan	13.9	6.7	17.7	2.3	1.3	4.0
Bolivia (Plurinational State of)	8.7	7.5	10.0	3.2	2.0	4.6
Bosnia and Herzegovina	1.4	1.3	1.5	0.5	0.5	0.6
Botswana	5.9	4.6	7.6	5.0	3.9	6.5
Brazil	2.4	2.3	2.5	1.0	1.0	1.1
Brunei Darussalam	1.9	1.7	2.1	1.2	1.0	1.4
Bulgaria	2.1	2.1	2.1	0.8	0.8	0.9
Burkina Faso	32.0	28.0	36.4	16.0	11.2	21.0
Burundi	40.9	33.7	50.9	9.9	6.1	14.6
Cabo Verde	3.7	3.4	4.0	1.0	0.9	1.2
Cambodia	24.7	19.9	30.1	3.7	2.6	4.7
Cameroon	24.6	21.5	27.9	19.9	14.9	24.4
Canada	1.0	1.0	1.0	0.3	0.3	0.4
Central African Republic	24.3	19.0	30.9	16.5	12.9	21.0
Chad	40.8	34.3	47.9	16.3	12.2	21.0
Chile	1.5	1.5	1.6	0.7	0.6	0.7
China	3.9	3.2	4.9	1.1	0.7	1.5
Colombia	2.7	2.2	3.4	1.5	1.1	2.1
Comoros	15.5	11.5	17.1	5.8	2.2	6.2
Congo	26.7	11.7	35.9	6.7	3.0	10.6
Cook Islands	3.2	2.5	4.1	1.2	1.0	1.6
Costa Rica	1.4	1.3	1.5	0.7	0.7	0.8
Côte d'Ivoire	19.9	17.0	22.9	16.8	12.3	22.1
Croatia	1.5	1.5	1.6	0.5	0.5	0.6
Cuba	1.8	1.8	1.9	1.0	0.9	1.0
Cyprus	1.1	1.0	1.2	0.5	0.4	0.6
Czechia	1.3	1.2	1.3	0.4	0.4	0.4
Democratic People's	5.4	4.3	7.0	2.6	2.1	3.4

Country	1990 $_5q_5$	1990 Lower bound	1990 Upper bound	2016 $_5q_5$	2016 Lower bound	2016 Upper bound
Republic of Korea						
Democratic Republic of the Congo	35.2	24.1	42.2	16.1	11.1	22.1
Denmark	0.9	0.9	1.0	0.2	0.2	0.2
Djibouti	9.0	2.8	16.5	1.8	0.3	2.0
Dominica	1.7	0.2	3.1	2.1	0.6	2.4
Dominican Republic	5.0	4.3	5.9	1.6	1.1	2.2
Ecuador	4.8	4.0	5.7	1.4	0.7	2.4
Egypt	6.0	6.0	6.1	2.5	2.4	2.5
El Salvador	5.2	4.1	6.2	0.6	0.3	1.4
Equatorial Guinea	27.5	21.5	35.3	12.0	9.4	15.4
Eritrea	34.7	28.6	42.4	9.0	5.2	12.0
Estonia	2.5	2.4	2.6	0.6	0.5	0.7
Ethiopia	59.6	48.7	71.9	11.9	7.5	15.6
Fiji	5.7	4.7	7.0	1.7	1.4	2.0
Finland	0.9	0.9	1.0	0.4	0.3	0.4
France	1.0	1.0	1.0	0.3	0.3	0.4
Gabon	15.8	10.4	20.5	4.8	2.2	9.2
Gambia	23.7	18.6	30.5	8.5	6.7	10.9
Georgia	4.1	2.4	5.8	1.3	1.1	1.4
Germany	1.0	1.0	1.0	0.4	0.4	0.4
Ghana	19.3	17.1	21.8	7.6	5.7	9.6
Greece	0.9	0.9	1.0	0.4	0.4	0.5
Grenada	1.8	1.4	2.3	1.3	1.0	1.7
Guatemala	7.4	7.1	7.7	1.6	1.5	1.6
Guinea	38.8	32.1	46.0	14.3	9.8	18.9
Guinea-Bissau	32.4	9.6	84.3	11.6	7.2	18.1
Guyana	6.8	5.0	9.6	2.5	1.2	4.4
Haiti	20.0	17.4	23.0	9.4	7.1	11.8
Honduras	6.9	5.4	8.3	1.4	0.6	2.5
Hungary	1.2	1.2	1.2	0.4	0.4	0.5
Iceland	0.9	0.8	1.1	0.4	0.3	0.6
India	13.6	13.2	14.2	3.4	3.0	3.6
Indonesia	10.6	9.7	11.7	3.3	2.5	4.2
Iran (Islamic Republic of)	5.5	4.3	7.1	1.6	1.3	2.1
Iraq	5.6	3.2	7.5	2.9	1.6	4.4
Ireland	0.9	0.9	1.0	0.3	0.3	0.4
Israel	1.0	1.0	1.1	0.4	0.4	0.5
Italy	0.8	0.8	0.9	0.3	0.3	0.3
Jamaica	2.3	1.8	3.0	1.3	1.0	1.6
Japan	0.9	0.9	1.0	0.4	0.4	0.4
Jordan	3.5	2.9	4.2	2.2	1.3	3.4
Kazakhstan	3.4	2.8	4.0	1.4	1.4	1.5
Kenya	12.8	11.4	14.3	6.2	4.7	7.9

Country	1990 $_5q_5$	1990 Lower bound	1990 Upper bound	2016 $_5q_5$	2016 Lower bound	2016 Upper bound
Kiribati	12.3	9.6	15.7	6.8	5.3	8.7
Kuwait	2.0	1.9	2.0	0.8	0.8	0.9
Kyrgyzstan	6.0	3.1	8.6	1.4	0.4	2.7
Lao People's Democratic Republic	21.9	17.2	28.1	8.0	6.3	10.3
Latvia	3.0	2.9	3.1	0.8	0.7	0.9
Lebanon	4.9	3.3	6.4	0.9	0.1	1.3
Lesotho	8.9	5.1	11.3	9.4	6.7	12.7
Liberia	22.3	18.4	27.3	9.0	6.3	12.0
Libya	5.6	4.2	7.7	2.8	2.3	3.1
Lithuania	2.3	2.2	2.4	0.8	0.7	0.9
Luxembourg	1.0	0.9	1.1	0.2	0.2	0.3
Madagascar	28.4	24.8	32.7	8.7	5.9	11.3
Malawi	29.0	25.7	32.6	9.4	7.9	11.0
Malaysia	2.4	2.4	2.5	1.1	1.1	1.2
Maldives	11.6	6.8	12.6	1.0	0.5	1.4
Mali	32.9	29.3	37.1	16.0	11.3	20.1
Malta	0.8	0.7	0.9	0.4	0.3	0.6
Marshall Islands	6.3	4.9	8.1	4.5	3.5	5.7
Mauritania	15.5	12.8	18.9	6.9	4.1	8.8
Mauritius	1.8	1.8	1.9	0.7	0.7	0.8
Mexico	2.7	2.5	2.9	1.1	1.1	1.1
Micronesia (Federated States of)	6.8	5.3	8.7	4.2	3.3	5.4
Monaco	0.8	0.6	1.0	0.5	0.4	0.6
Mongolia	14.0	10.9	18.0	2.4	1.9	3.1
Montenegro	1.2	1.0	1.4	0.6	0.5	0.7
Morocco	6.6	5.7	7.6	2.1	1.3	2.8
Mozambique	49.1	35.8	60.9	8.4	4.9	11.8
Myanmar	15.0	11.8	19.3	6.3	5.0	8.1
Namibia	10.8	8.8	13.4	6.2	3.8	8.7
Nauru	7.3	5.7	9.4	4.4	3.4	5.6
Nepal	20.5	18.2	23.2	3.6	2.6	4.4
Netherlands	0.9	0.9	0.9	0.3	0.3	0.4
New Zealand	1.2	1.1	1.2	0.4	0.3	0.4
Nicaragua	5.6	4.2	7.2	1.5	0.5	2.9
Niger	56.0	49.4	62.8	22.7	16.5	29.9
Nigeria	31.4	27.2	36.1	14.0	10.9	17.2
Niue	1.9	1.5	2.5	2.9	2.3	3.7
Norway	0.9	0.9	1.0	0.3	0.2	0.3
Oman	3.8	3.0	4.9	1.2	1.0	1.5
Pakistan	8.1	7.8	8.5	6.1	5.2	7.3
Palau	4.5	3.5	5.8	2.2	1.7	2.8
Panama	2.6	2.1	3.3	1.4	1.3	1.5
Papua New Guinea	11.2	8.8	14.3	6.8	5.3	8.7

Country	1990 $_5q_5$	1990 Lower bound	1990 Upper bound	2016 $_5q_5$	2016 Lower bound	2016 Upper bound
Paraguay	4.4	3.5	5.6	1.4	0.6	2.7
Peru	6.4	5.7	7.1	1.9	1.5	2.3
Philippines	10.6	9.3	12.0	3.2	2.1	4.6
Poland	1.4	1.4	1.4	0.4	0.4	0.4
Portugal	2.0	1.9	2.0	0.4	0.3	0.4
Qatar	1.6	1.4	1.9	0.9	0.8	1.0
Republic of Korea	3.1	3.0	3.1	0.4	0.4	0.4
Republic of Moldova	3.0	2.9	3.2	1.0	0.9	1.1
Romania	3.0	3.0	3.1	0.8	0.8	0.9
Russian Federation	2.7	2.7	2.8	1.1	1.1	1.1
Rwanda	49.2	43.7	55.3	6.4	4.8	8.1
Saint Kitts and Nevis	2.4	1.9	3.2	0.8	0.7	1.1
Saint Lucia	2.4	2.2	2.7	1.1	0.8	1.4
Saint Vincent and the Grenadines	2.1	1.9	2.3	1.7	1.2	2.2
Samoa	3.9	3.1	5.0	2.3	1.8	3.0
San Marino	1.1	0.8	1.4	0.4	0.3	0.5
Sao Tome and Principe	15.0	13.2	17.4	7.5	4.5	9.4
Saudi Arabia	4.4	3.4	5.6	1.4	1.1	1.8
Senegal	26.5	24.2	29.1	9.1	7.0	11.1
Serbia	1.7	1.6	1.7	0.6	0.5	0.6
Seychelles	2.4	1.9	3.1	2.1	1.7	2.7
Sierra Leone	40.1	31.3	51.2	15.3	12.0	19.5
Singapore	1.0	1.0	1.1	0.3	0.2	0.3
Slovakia	1.4	1.3	1.4	0.7	0.6	0.7
Slovenia	1.2	1.1	1.3	0.4	0.3	0.4
Solomon Islands	4.8	3.8	6.1	3.3	2.6	4.2
Somalia	26.6	20.8	34.2	18.7	14.6	24.0
South Africa	7.6	7.2	8.0	2.4	2.4	2.5
South Sudan	40.1	31.2	51.4	12.3	9.6	15.8
Spain	1.1	1.1	1.2	0.4	0.3	0.4
Sri Lanka	3.4	3.4	3.5	1.0	1.0	1.1
State of Palestine	4.4	1.7	12.3	2.0	1.2	3.5
Sudan	23.2	20.1	27.1	6.4	4.5	8.1
Suriname	3.5	2.8	4.5	1.6	1.3	2.1
Swaziland	8.6	4.0	18.5	8.3	4.8	14.5
Sweden	0.7	0.7	0.7	0.3	0.3	0.4
Switzerland	1.0	1.0	1.0	0.3	0.3	0.3
Syrian Arab Republic	4.3	3.4	5.5	1.6	0.8	2.6
Tajikistan	7.7	1.2	55.9	3.0	1.3	6.8
Thailand	5.2	4.1	6.3	1.5	0.9	2.4
The former Yugoslav Republic of Macedonia	1.7	1.7	1.8	0.5	0.4	0.6
Timor-Leste	24.0	18.8	30.7	6.2	4.9	7.9

Country	1990 $_5q_5$	1990 Lower bound	1990 Upper bound	2016 $_5q_5$	2016 Lower bound	2016 Upper bound
Togo	29.5	25.2	34.5	12.9	9.0	17.7
Tonga	2.9	2.2	3.7	2.2	1.7	2.8
Trinidad and Tobago	1.9	1.8	2.0	1.2	1.0	1.4
Tunisia	5.7	4.5	7.2	1.8	0.4	3.1
Turkey	6.7	5.6	8.1	1.1	1.0	1.1
Turkmenistan	3.7	3.6	3.7	1.9	1.8	1.9
Tuvalu	7.1	5.6	9.1	3.3	2.6	4.2
Uganda	23.0	20.3	26.2	10.0	7.0	12.9
Ukraine	2.4	2.4	2.5	0.9	0.8	0.9
United Arab Emirates	2.9	1.4	4.3	0.7	0.6	0.9
United Kingdom	0.9	0.9	0.9	0.4	0.4	0.4
United Republic of Tanzania	20.8	18.4	23.7	8.9	6.8	10.7
United States	1.1	1.1	1.1	0.6	0.6	0.6
Uruguay	1.6	1.5	1.6	0.8	0.7	0.8
Uzbekistan	3.3	3.3	3.3	1.7	1.6	1.7
Vanuatu	4.5	3.5	5.7	3.5	2.8	4.5
Venezuela (Bolivarian Republic of)	2.2	2.1	2.2	1.1	1.1	1.2
Viet Nam	10.1	8.2	12.2	1.3	0.6	2.5
Yemen	13.3	11.9	14.9	4.0	3.0	5.0
Zambia	19.7	17.4	22.4	9.7	7.3	12.0
Zimbabwe	8.3	7.1	9.7	9.3	7.1	11.5

Table S10 – *Number of deaths among children aged 5-14 (thousands) in 1990 and 2016*

Country	1990 5-14	1990 Lower bound	1990 Upper bound	2016 5-14	2016 Lower bound	2016 Upper bound
Afghanistan	7.3	4.8	10.2	9.6	6.6	15.0
Albania	0.4	0.3	0.5	0.1	0.1	0.1
Algeria	6.5	6.2	7.0	2.9	2.8	3.1
Andorra	0.0	0.0	0.0	0.0	0.0	0.0
Angola	16.9	13.1	21.7	13.4	10.4	17.1
Antigua and Barbuda	0.0	0.0	0.0	0.0	0.0	0.0
Argentina	2.2	2.2	2.3	1.5	1.4	1.6
Armenia	0.3	0.2	0.4	0.1	0.1	0.1
Australia	0.5	0.5	0.5	0.3	0.3	0.3
Austria	0.2	0.2	0.2	0.1	0.1	0.1
Azerbaijan	0.8	0.8	0.8	0.4	0.4	0.4
Bahamas	0.0	0.0	0.0	0.0	0.0	0.0
Bahrain	0.0	0.0	0.0	0.0	0.0	0.0
Bangladesh	74.7	67.6	82.3	15.7	12.6	19.6
Barbados	0.0	0.0	0.0	0.0	0.0	0.0
Belarus	0.6	0.6	0.6	0.1	0.1	0.2
Belgium	0.2	0.2	0.3	0.1	0.1	0.1

Country	1990 5-14	1990 Lower bound	1990 Upper bound	2016 5-14	2016 Lower bound	2016 Upper bound
Belize	0.0	0.0	0.0	0.0	0.0	0.0
Benin	6.6	5.9	7.4	6.7	4.4	10.5
Bhutan	0.3	0.2	0.4	0.1	0.1	0.1
Bolivia (Plurinational State of)	2.5	2.1	2.8	1.5	0.9	2.8
Bosnia and Herzegovina	0.2	0.2	0.2	0.0	0.0	0.0
Botswana	0.4	0.3	0.5	0.4	0.3	0.5
Brazil	16.5	14.0	19.6	7.7	7.4	8.1
Brunei Darussalam	0.0	0.0	0.0	0.0	0.0	0.0
Bulgaria	0.5	0.5	0.5	0.1	0.1	0.1
Burkina Faso	10.9	9.6	12.2	13.8	8.8	20.4
Burundi	9.2	6.9	12.5	5.7	3.1	10.5
Cabo Verde	0.1	0.1	0.1	0.0	0.0	0.0
Cambodia	9.2	7.9	10.6	1.7	1.1	2.5
Cameroon	12.8	11.3	14.4	19.2	13.7	27.2
Canada	0.8	0.8	0.8	0.3	0.3	0.4
Central African Republic	2.9	2.2	3.7	2.9	2.3	3.7
Chad	9.7	8.5	11.2	11.4	8.5	15.3
Chile	0.8	0.8	0.8	0.4	0.4	0.4
China	138.5	115.4	162.5	45.3	30.5	68.6
Colombia	4.2	3.5	5.0	2.4	1.5	3.5
Comoros	0.2	0.2	0.3	0.1	0.1	0.2
Congo	2.6	1.5	4.3	1.5	1.0	2.5
Cook Islands	0.0	0.0	0.0	0.0	0.0	0.0
Costa Rica	0.2	0.2	0.2	0.1	0.1	0.1
Côte d'Ivoire	10.8	9.3	12.4	17.9	12.3	28.3
Croatia	0.2	0.2	0.2	0.0	0.0	0.1
Cuba	0.6	0.6	0.6	0.3	0.2	0.3
Cyprus	0.0	0.0	0.0	0.0	0.0	0.0
Czechia	0.4	0.4	0.4	0.1	0.1	0.1
Democratic People's Republic of Korea	3.0	2.3	3.8	1.4	1.1	1.8
Democratic Republic of the Congo	44.2	27.7	73.9	63.7	44.6	91.7
Denmark	0.1	0.1	0.1	0.0	0.0	0.0
Djibouti	0.3	0.1	0.6	0.0	0.0	0.1
Dominica	0.0	0.0	0.0	0.0	0.0	0.0
Dominican Republic	1.4	1.2	1.7	0.6	0.5	0.9
Ecuador	2.0	1.7	2.3	1.1	0.6	2.0
Egypt	16.0	15.7	16.3	9.3	9.1	9.6
El Salvador	1.0	0.8	1.2	0.4	0.3	0.9
Equatorial Guinea	0.5	0.4	0.6	0.5	0.4	0.6
Eritrea	4.4	3.7	5.1	1.7	1.0	3.1
Estonia	0.1	0.1	0.1	0.0	0.0	0.0
Ethiopia	116.7	99.6	137.0	47.5	26.9	72.8

Country	1990 5-14	1990 Lower bound	1990 Upper bound	2016 5-14	2016 Lower bound	2016 Upper bound
Fiji	0.2	0.1	0.2	0.1	0.1	0.1
Finland	0.1	0.1	0.1	0.0	0.0	0.1
France	1.5	1.5	1.5	0.6	0.5	0.7
Gabon	0.5	0.4	0.7	0.7	0.4	1.2
Gambia	0.9	0.7	1.2	0.7	0.6	0.9
Georgia	0.6	0.4	1.1	0.1	0.1	0.1
Germany	1.6	1.6	1.6	0.5	0.5	0.6
Ghana	11.8	10.5	13.5	8.8	6.3	11.9
Greece	0.3	0.3	0.3	0.1	0.1	0.1
Grenada	0.0	0.0	0.0	0.0	0.0	0.0
Guatemala	3.3	2.9	3.8	1.5	1.4	1.6
Guinea	8.8	7.5	10.3	7.5	6.0	9.6
Guinea-Bissau	1.1	0.3	2.9	0.8	0.5	1.3
Guyana	0.2	0.1	0.3	0.1	0.0	0.2
Haiti	6.1	5.4	7.1	3.5	2.4	5.1
Honduras	1.3	1.1	1.6	0.8	0.5	1.2
Hungary	0.4	0.4	0.4	0.1	0.1	0.1
Iceland	0.0	0.0	0.0	0.0	0.0	0.0
India	448.8	429.2	468.5	160.0	142.5	178.2
Indonesia	68.7	62.6	75.9	25.0	17.6	37.1
Iran (Islamic Republic of)	14.6	11.3	18.6	3.4	2.6	4.3
Iraq	4.2	2.5	8.1	5.3	3.4	8.5
Ireland	0.1	0.1	0.1	0.1	0.0	0.1
Israel	0.2	0.2	0.2	0.1	0.1	0.1
Italy	1.2	1.2	1.3	0.4	0.4	0.5
Jamaica	0.3	0.2	0.4	0.1	0.1	0.2
Japan	2.7	2.6	2.7	0.9	0.9	0.9
Jordan	0.5	0.4	0.6	0.9	0.6	1.7
Kazakhstan	1.9	1.4	2.7	0.9	0.8	0.9
Kenya	13.4	11.9	15.2	13.8	9.3	20.1
Kiribati	0.0	0.0	0.0	0.0	0.0	0.0
Kuwait	0.2	0.2	0.2	0.1	0.1	0.1
Kyrgyzstan	0.9	0.6	1.4	0.3	0.2	0.6
Lao People's Democratic Republic	3.3	2.6	4.2	1.5	1.2	1.9
Latvia	0.2	0.2	0.2	0.0	0.0	0.0
Lebanon	0.4	0.3	0.6	0.1	0.0	0.4
Lesotho	0.6	0.4	0.9	0.7	0.5	1.1
Liberia	2.0	1.6	2.6	2.1	1.3	3.2
Libya	1.0	0.8	1.3	0.6	0.5	0.7
Lithuania	0.2	0.2	0.2	0.0	0.0	0.1
Luxembourg	0.0	0.0	0.0	0.0	0.0	0.0
Madagascar	14.8	13.1	17.1	8.9	5.5	14.1
Malawi	11.1	9.9	12.4	7.2	5.8	9.1

Country	1990 5-14	1990 Lower bound	1990 Upper bound	2016 5-14	2016 Lower bound	2016 Upper bound
Malaysia	2.1	2.0	2.2	1.3	1.3	1.4
Maldives	0.1	0.0	0.2	0.0	0.0	0.0
Mali	11.7	10.5	13.0	13.0	8.3	20.0
Malta	0.0	0.0	0.0	0.0	0.0	0.0
Marshall Islands	0.0	0.0	0.0	0.0	0.0	0.0
Mauritania	1.3	1.1	1.5	1.0	0.6	2.0
Mauritius	0.1	0.1	0.1	0.0	0.0	0.0
Mexico	11.9	9.4	15.1	5.7	5.5	6.0
Micronesia (Federated States of)	0.0	0.0	0.0	0.0	0.0	0.0
Monaco	0.0	0.0	0.0	0.0	0.0	0.0
Mongolia	1.1	0.8	1.4	0.2	0.2	0.3
Montenegro	0.0	0.0	0.0	0.0	0.0	0.0
Morocco	6.9	6.1	7.8	2.1	1.2	3.6
Mozambique	28.1	21.2	37.6	12.5	8.1	18.1
Myanmar	20.4	16.0	26.3	8.0	6.2	10.2
Namibia	0.7	0.6	0.8	0.6	0.4	0.9
Nauru	0.0	0.0	0.0	0.0	0.0	0.0
Nepal	14.8	13.4	16.5	3.2	2.0	4.7
Netherlands	0.3	0.3	0.3	0.2	0.1	0.2
New Zealand	0.1	0.1	0.1	0.1	0.0	0.1
Nicaragua	1.0	0.8	1.2	0.5	0.3	0.8
Niger	17.5	15.7	19.7	25.7	18.3	36.1
Nigeria	116.7	100.4	134.8	107.5	77.5	146.0
Niue	0.0	0.0	0.0	0.0	0.0	0.0
Norway	0.1	0.1	0.1	0.0	0.0	0.0
Oman	0.3	0.3	0.4	0.1	0.1	0.2
Pakistan	38.8	36.3	41.3	48.3	30.0	89.0
Palau	0.0	0.0	0.0	0.0	0.0	0.0
Panama	0.4	0.2	0.6	0.2	0.2	0.2
Papua New Guinea	1.8	1.4	2.3	1.7	1.3	2.1
Paraguay	0.8	0.6	1.0	0.5	0.2	1.0
Peru	5.9	5.3	6.7	2.0	1.6	2.9
Philippines	23.4	21.0	26.5	13.9	9.6	20.4
Poland	1.8	1.8	1.9	0.4	0.4	0.4
Portugal	0.6	0.6	0.6	0.1	0.1	0.1
Qatar	0.0	0.0	0.0	0.0	0.0	0.0
Republic of Korea	4.1	4.1	4.2	0.4	0.4	0.4
Republic of Moldova	0.4	0.4	0.4	0.1	0.1	0.1
Romania	2.0	2.0	2.0	0.4	0.3	0.4
Russian Federation	11.5	11.3	11.7	3.7	3.4	4.1
Rwanda	15.7	13.5	18.1	3.7	2.4	5.9
Saint Kitts and Nevis	0.0	0.0	0.0	0.0	0.0	0.0
Saint Lucia	0.0	0.0	0.0	0.0	0.0	0.0

Country	1990 5-14	1990 Lower bound	1990 Upper bound	2016 5-14	2016 Lower bound	2016 Upper bound
Saint Vincent and the Grenadines	0.0	0.0	0.0	0.0	0.0	0.0
Samoa	0.0	0.0	0.0	0.0	0.0	0.0
San Marino	0.0	0.0	0.0	0.0	0.0	0.0
Sao Tome and Principe	0.1	0.1	0.1	0.1	0.0	0.1
Saudi Arabia	3.0	2.4	3.9	1.3	1.0	1.6
Senegal	8.5	7.6	9.5	6.7	4.8	9.3
Serbia	0.5	0.5	0.5	0.1	0.1	0.1
Seychelles	0.0	0.0	0.0	0.0	0.0	0.0
Sierra Leone	6.9	5.3	8.8	4.3	3.4	5.5
Singapore	0.1	0.1	0.1	0.0	0.0	0.0
Slovakia	0.2	0.2	0.2	0.1	0.1	0.1
Slovenia	0.1	0.1	0.1	0.0	0.0	0.0
Solomon Islands	0.1	0.1	0.1	0.1	0.1	0.1
Somalia	7.5	5.9	9.7	10.3	8.1	13.3
South Africa	10.2	8.0	13.2	5.5	5.3	5.8
South Sudan	8.7	6.7	11.2	5.6	4.3	7.2
Spain	1.3	1.3	1.4	0.4	0.3	0.4
Sri Lanka	2.4	2.3	2.4	0.8	0.7	0.9
State of Palestine	0.4	0.2	1.2	0.4	0.2	0.7
Sudan	17.7	14.8	21.5	9.6	6.6	13.6
Suriname	0.1	0.0	0.1	0.0	0.0	0.0
Swaziland	0.5	0.2	1.0	0.4	0.2	0.7
Sweden	0.1	0.1	0.1	0.1	0.1	0.1
Switzerland	0.1	0.1	0.2	0.1	0.0	0.1
Syrian Arab Republic	2.8	2.3	3.4	1.5	0.8	3.1
Tajikistan	2.4	0.4	18.4	0.7	0.3	1.6
Thailand	9.6	8.0	11.4	2.7	1.7	4.3
The former Yugoslav Republic of Macedonia	0.1	0.1	0.1	0.0	0.0	0.0
Timor-Leste	0.6	0.4	0.7	0.3	0.2	0.4
Togo	4.4	3.9	5.1	4.6	3.3	6.5
Tonga	0.0	0.0	0.0	0.0	0.0	0.0
Trinidad and Tobago	0.1	0.1	0.1	0.1	0.0	0.1
Tunisia	1.5	1.2	1.9	0.5	0.3	1.1
Turkey	11.8	9.9	14.2	3.0	2.9	3.1
Turkmenistan	0.6	0.6	0.6	0.4	0.4	0.4
Tuvalu	0.0	0.0	0.0	0.0	0.0	0.0
Uganda	17.1	15.3	19.2	20.2	14.0	28.2
Ukraine	3.4	3.3	3.5	0.8	0.8	0.8
United Arab Emirates	0.2	0.1	0.8	0.1	0.1	0.1
United Kingdom	1.3	1.3	1.3	0.6	0.6	0.7
United Republic of Tanzania	22.8	20.2	25.6	19.6	14.5	27.7
United States	8.6	8.5	8.8	5.4	5.1	5.7

Country	1990 5-14	1990 Lower bound	1990 Upper bound	2016 5-14	2016 Lower bound	2016 Upper bound
Uruguay	0.2	0.2	0.2	0.1	0.1	0.1
Uzbekistan	3.1	3.1	3.2	1.9	1.9	2.0
Vanuatu	0.0	0.0	0.0	0.0	0.0	0.0
Venezuela (Bolivarian Republic of)	2.1	2.1	2.2	1.5	1.4	1.7
Viet Nam	22.1	18.5	26.7	3.9	2.9	5.2
Yemen	7.9	7.2	8.8	4.4	3.1	6.1
Zambia	7.2	6.4	8.1	6.7	4.5	9.7
Zimbabwe	3.9	3.2	4.7	6.5	4.5	8.8

Table S11 – *Number of deaths among children aged 5-9 (thousands) in 1990 and 2016*

Country	1990 5-9	1990 Lower bound	1990 Upper bound	2016 5-9	2016 Lower bound	2016 Upper bound
Afghanistan	4.0	1.5	6.8	6.2	4.0	8.8
Albania	0.2	0.2	0.3	0.0	0.0	0.0
Algeria	3.9	3.9	4.0	1.8	1.7	1.8
Andorra	0.0	0.0	0.0	0.0	0.0	0.0
Angola	12.8	10.0	16.4	10.1	7.9	12.9
Antigua and Barbuda	0.0	0.0	0.0	0.0	0.0	0.0
Argentina	1.1	1.1	1.1	0.7	0.6	0.7
Armenia	0.2	0.1	0.2	0.0	0.0	0.0
Australia	0.2	0.2	0.2	0.1	0.1	0.1
Austria	0.1	0.1	0.1	0.0	0.0	0.0
Azerbaijan	0.5	0.5	0.5	0.2	0.2	0.2
Bahamas	0.0	0.0	0.0	0.0	0.0	0.0
Bahrain	0.0	0.0	0.0	0.0	0.0	0.0
Bangladesh	51.0	48.9	53.2	8.1	7.5	8.8
Barbados	0.0	0.0	0.0	0.0	0.0	0.0
Belarus	0.3	0.3	0.3	0.1	0.1	0.1
Belgium	0.1	0.1	0.1	0.1	0.1	0.1
Belize	0.0	0.0	0.0	0.0	0.0	0.0
Benin	4.9	4.3	5.5	4.5	3.3	5.7
Bhutan	0.2	0.1	0.3	0.0	0.0	0.1
Bolivia (Plurinational State of)	1.6	1.4	1.9	0.7	0.5	1.1
Bosnia and Herzegovina	0.1	0.1	0.1	0.0	0.0	0.0
Botswana	0.3	0.2	0.3	0.2	0.2	0.3
Brazil	8.8	8.3	9.1	3.0	3.0	3.2
Brunei Darussalam	0.0	0.0	0.0	0.0	0.0	0.0
Bulgaria	0.2	0.2	0.3	0.1	0.1	0.1
Burkina Faso	8.8	7.7	10.1	9.1	6.4	12.0
Burundi	7.0	5.7	8.7	3.1	1.9	4.6
Cabo Verde	0.0	0.0	0.0	0.0	0.0	0.0
Cambodia	7.2	5.8	8.8	1.3	0.9	1.6

Country	1990 5-9	1990 Lower bound	1990 Upper bound	2016 5-9	2016 Lower bound	2016 Upper bound
Cameroon	8.9	7.8	10.1	13.5	10.1	16.6
Canada	0.4	0.4	0.4	0.1	0.1	0.1
Central African Republic	2.1	1.6	2.6	2.2	1.7	2.8
Chad	7.6	6.4	8.9	7.4	5.6	9.6
Chile	0.4	0.4	0.4	0.2	0.2	0.2
China	82.0	67.9	102.5	18.3	11.6	25.5
Colombia	2.3	1.9	2.8	1.2	0.8	1.6
Comoros	0.2	0.1	0.2	0.1	0.0	0.1
Congo	1.9	0.8	2.6	1.0	0.4	1.6
Cook Islands	0.0	0.0	0.0	0.0	0.0	0.0
Costa Rica	0.1	0.1	0.1	0.1	0.0	0.1
Côte d'Ivoire	7.3	6.3	8.4	11.2	8.2	14.8
Croatia	0.1	0.1	0.1	0.0	0.0	0.0
Cuba	0.3	0.3	0.3	0.1	0.1	0.1
Cyprus	0.0	0.0	0.0	0.0	0.0	0.0
Czechia	0.2	0.2	0.2	0.0	0.0	0.0
Democratic People's Republic of Korea	2.0	1.6	2.6	0.9	0.7	1.2
Democratic Republic of the Congo	36.2	24.6	43.6	39.2	27.1	53.9
Denmark	0.0	0.0	0.1	0.0	0.0	0.0
Djibouti	0.2	0.0	0.3	0.0	0.0	0.0
Dominica	0.0	0.0	0.0	0.0	0.0	0.0
Dominican Republic	0.9	0.8	1.1	0.3	0.2	0.5
Ecuador	1.2	1.0	1.5	0.4	0.2	0.7
Egypt	9.5	9.4	9.6	5.1	5.0	5.1
El Salvador	0.7	0.6	0.9	0.1	0.0	0.2
Equatorial Guinea	0.4	0.3	0.5	0.4	0.3	0.5
Eritrea	3.3	2.7	4.0	1.3	0.7	1.7
Estonia	0.1	0.1	0.1	0.0	0.0	0.0
Ethiopia	89.0	72.3	108.1	33.0	20.8	43.5
Fiji	0.1	0.1	0.1	0.0	0.0	0.0
Finland	0.1	0.1	0.1	0.0	0.0	0.0
France	0.8	0.7	0.8	0.2	0.2	0.3
Gabon	0.4	0.3	0.5	0.2	0.1	0.4
Gambia	0.7	0.5	0.8	0.5	0.4	0.7
Georgia	0.4	0.2	0.5	0.1	0.1	0.1
Germany	0.9	0.8	0.9	0.3	0.2	0.3
Ghana	8.2	7.3	9.3	5.5	4.2	7.0
Greece	0.1	0.1	0.1	0.0	0.0	0.1
Grenada	0.0	0.0	0.0	0.0	0.0	0.0
Guatemala	2.1	2.0	2.2	0.6	0.6	0.6
Guinea	6.8	5.6	8.1	5.0	3.4	6.7
Guinea-Bissau	1.0	0.3	2.7	0.6	0.4	0.9

Country	1990 5-9	1990 Lower bound	1990 Upper bound	2016 5-9	2016 Lower bound	2016 Upper bound
Guyana	0.1	0.1	0.2	0.0	0.0	0.1
Haiti	4.3	3.7	4.9	2.3	1.7	2.9
Honduras	1.1	0.8	1.3	0.3	0.1	0.5
Hungary	0.2	0.2	0.2	0.0	0.0	0.0
Iceland	0.0	0.0	0.0	0.0	0.0	0.0
India	303.5	294.3	316.7	86.0	77.1	91.5
Indonesia	47.9	43.8	52.7	15.7	11.9	20.0
Iran (Islamic Republic of)	9.8	7.7	12.6	2.1	1.7	2.7
Iraq	3.0	1.7	4.0	2.9	1.6	4.4
Ireland	0.1	0.1	0.1	0.0	0.0	0.0
Israel	0.1	0.1	0.1	0.1	0.1	0.1
Italy	0.5	0.5	0.5	0.2	0.2	0.2
Jamaica	0.1	0.1	0.2	0.1	0.0	0.1
Japan	1.4	1.4	1.5	0.4	0.4	0.5
Jordan	0.4	0.3	0.5	0.5	0.3	0.8
Kazakhstan	1.2	1.0	1.4	0.5	0.5	0.5
Kenya	9.9	8.8	11.1	8.4	6.3	10.6
Kiribati	0.0	0.0	0.0	0.0	0.0	0.0
Kuwait	0.1	0.1	0.1	0.0	0.0	0.1
Kyrgyzstan	0.7	0.3	0.9	0.2	0.0	0.3
Lao People's Democratic Republic	2.7	2.1	3.5	1.2	1.0	1.6
Latvia	0.1	0.1	0.1	0.0	0.0	0.0
Lebanon	0.3	0.2	0.4	0.1	0.0	0.1
Lesotho	0.4	0.2	0.5	0.5	0.3	0.6
Liberia	1.4	1.2	1.7	1.2	0.8	1.6
Libya	0.7	0.5	1.0	0.3	0.3	0.4
Lithuania	0.1	0.1	0.1	0.0	0.0	0.0
Luxembourg	0.0	0.0	0.0	0.0	0.0	0.0
Madagascar	9.9	8.6	11.4	6.0	4.1	7.7
Malawi	8.0	7.1	9.1	5.2	4.3	6.1
Malaysia	1.1	1.1	1.1	0.5	0.5	0.6
Maldives	0.1	0.0	0.1	0.0	0.0	0.0
Mali	8.5	7.6	9.6	9.3	6.6	11.8
Malta	0.0	0.0	0.0	0.0	0.0	0.0
Marshall Islands	0.0	0.0	0.0	0.0	0.0	0.0
Mauritania	0.9	0.8	1.1	0.8	0.5	1.0
Mauritius	0.0	0.0	0.0	0.0	0.0	0.0
Mexico	5.9	5.5	6.4	2.5	2.4	2.5
Micronesia (Federated States of)	0.0	0.0	0.0	0.0	0.0	0.0
Monaco	0.0	0.0	0.0	0.0	0.0	0.0
Mongolia	0.8	0.6	1.1	0.1	0.1	0.2
Montenegro	0.0	0.0	0.0	0.0	0.0	0.0
Morocco	4.5	3.9	5.2	1.3	0.8	1.8

Country	1990 5-9	1990 Lower bound	1990 Upper bound	2016 5-9	2016 Lower bound	2016 Upper bound
Mozambique	20.7	15.0	25.8	7.3	4.3	10.2
Myanmar	15.9	12.5	20.4	6.0	4.8	7.8
Namibia	0.4	0.4	0.5	0.4	0.2	0.5
Nauru	0.0	0.0	0.0	0.0	0.0	0.0
Nepal	10.9	9.6	12.4	2.2	1.6	2.7
Netherlands	0.2	0.2	0.2	0.1	0.1	0.1
New Zealand	0.1	0.1	0.1	0.0	0.0	0.0
Nicaragua	0.7	0.6	0.9	0.2	0.1	0.4
Niger	14.3	12.6	16.1	15.6	11.3	20.6
Nigeria	89.7	77.6	103.5	76.5	59.5	93.9
Niue	0.0	0.0	0.0	0.0	0.0	0.0
Norway	0.0	0.0	0.0	0.0	0.0	0.0
Oman	0.2	0.2	0.3	0.1	0.1	0.1
Pakistan	24.6	23.6	25.8	27.7	23.4	33.0
Palau	0.0	0.0	0.0	0.0	0.0	0.0
Panama	0.2	0.1	0.2	0.1	0.1	0.1
Papua New Guinea	1.4	1.1	1.7	1.3	1.0	1.7
Paraguay	0.5	0.4	0.7	0.2	0.1	0.4
Peru	3.5	3.2	3.9	1.1	0.9	1.4
Philippines	17.9	15.8	20.3	7.0	4.7	10.2
Poland	0.9	0.9	1.0	0.2	0.2	0.2
Portugal	0.3	0.3	0.3	0.0	0.0	0.0
Qatar	0.0	0.0	0.0	0.0	0.0	0.0
Republic of Korea	2.4	2.3	2.4	0.2	0.2	0.2
Republic of Moldova	0.2	0.2	0.3	0.0	0.0	0.0
Romania	1.1	1.1	1.1	0.2	0.2	0.2
Russian Federation	6.3	6.3	6.4	1.8	1.8	1.8
Rwanda	12.1	10.7	13.6	2.1	1.6	2.7
Saint Kitts and Nevis	0.0	0.0	0.0	0.0	0.0	0.0
Saint Lucia	0.0	0.0	0.0	0.0	0.0	0.0
Saint Vincent and the Grenadines	0.0	0.0	0.0	0.0	0.0	0.0
Samoa	0.0	0.0	0.0	0.0	0.0	0.0
San Marino	0.0	0.0	0.0	0.0	0.0	0.0
Sao Tome and Principe	0.1	0.0	0.1	0.0	0.0	0.1
Saudi Arabia	2.1	1.6	2.6	0.8	0.6	1.0
Senegal	6.3	5.8	7.0	4.0	3.1	4.9
Serbia	0.3	0.2	0.3	0.1	0.1	0.1
Seychelles	0.0	0.0	0.0	0.0	0.0	0.0
Sierra Leone	5.2	4.0	6.6	3.3	2.5	4.2
Singapore	0.0	0.0	0.0	0.0	0.0	0.0
Slovakia	0.1	0.1	0.1	0.0	0.0	0.0
Slovenia	0.0	0.0	0.0	0.0	0.0	0.0
Solomon Islands	0.0	0.0	0.1	0.1	0.0	0.1

Country	1990			2016		
	5-9	Lower bound	Upper bound	5-9	Lower bound	Upper bound
Somalia	5.5	4.3	7.1	8.3	6.5	10.7
South Africa	7.6	7.2	8.0	2.6	2.6	2.7
South Sudan	6.6	5.1	8.5	4.2	3.3	5.4
Spain	0.6	0.6	0.6	0.2	0.2	0.2
Sri Lanka	1.3	1.3	1.3	0.3	0.3	0.4
State of Palestine	0.3	0.1	0.8	0.3	0.1	0.4
Sudan	14.2	12.2	16.6	7.0	4.9	8.8
Suriname	0.0	0.0	0.0	0.0	0.0	0.0
Swaziland	0.2	0.1	0.5	0.3	0.2	0.5
Sweden	0.1	0.1	0.1	0.0	0.0	0.0
Switzerland	0.1	0.1	0.1	0.0	0.0	0.0
Syrian Arab Republic	1.7	1.3	2.2	0.8	0.4	1.3
Tajikistan	1.2	0.2	8.8	0.6	0.3	1.4
Thailand	5.9	4.7	7.2	1.2	0.7	1.9
The former Yugoslav Republic of Macedonia	0.1	0.1	0.1	0.0	0.0	0.0
Timor-Leste	0.5	0.4	0.6	0.2	0.2	0.3
Togo	3.5	2.9	4.1	2.8	1.9	3.8
Tonga	0.0	0.0	0.0	0.0	0.0	0.0
Trinidad and Tobago	0.1	0.1	0.1	0.0	0.0	0.0
Tunisia	1.2	0.9	1.5	0.3	0.1	0.5
Turkey	8.8	7.3	10.7	1.5	1.4	1.5
Turkmenistan	0.4	0.3	0.4	0.2	0.2	0.2
Tuvalu	0.0	0.0	0.0	0.0	0.0	0.0
Uganda	12.6	11.1	14.4	13.3	9.3	17.2
Ukraine	1.8	1.8	1.9	0.4	0.4	0.4
United Arab Emirates	0.1	0.1	0.2	0.1	0.1	0.1
United Kingdom	0.7	0.6	0.7	0.3	0.3	0.3
United Republic of Tanzania	16.1	14.2	18.3	14.9	11.4	17.9
United States	4.0	3.9	4.0	2.5	2.4	2.5
Uruguay	0.1	0.1	0.1	0.0	0.0	0.0
Uzbekistan	1.8	1.8	1.8	1.0	1.0	1.0
Vanuatu	0.0	0.0	0.0	0.0	0.0	0.0
Venezuela (Bolivarian Republic of)	1.1	1.1	1.1	0.6	0.6	0.7
Viet Nam	17.3	14.0	20.8	1.9	0.8	3.7
Yemen	5.5	4.9	6.1	3.0	2.2	3.7
Zambia	4.9	4.3	5.6	4.8	3.6	5.9
Zimbabwe	2.7	2.3	3.1	4.2	3.2	5.2

6 Country-specific plots

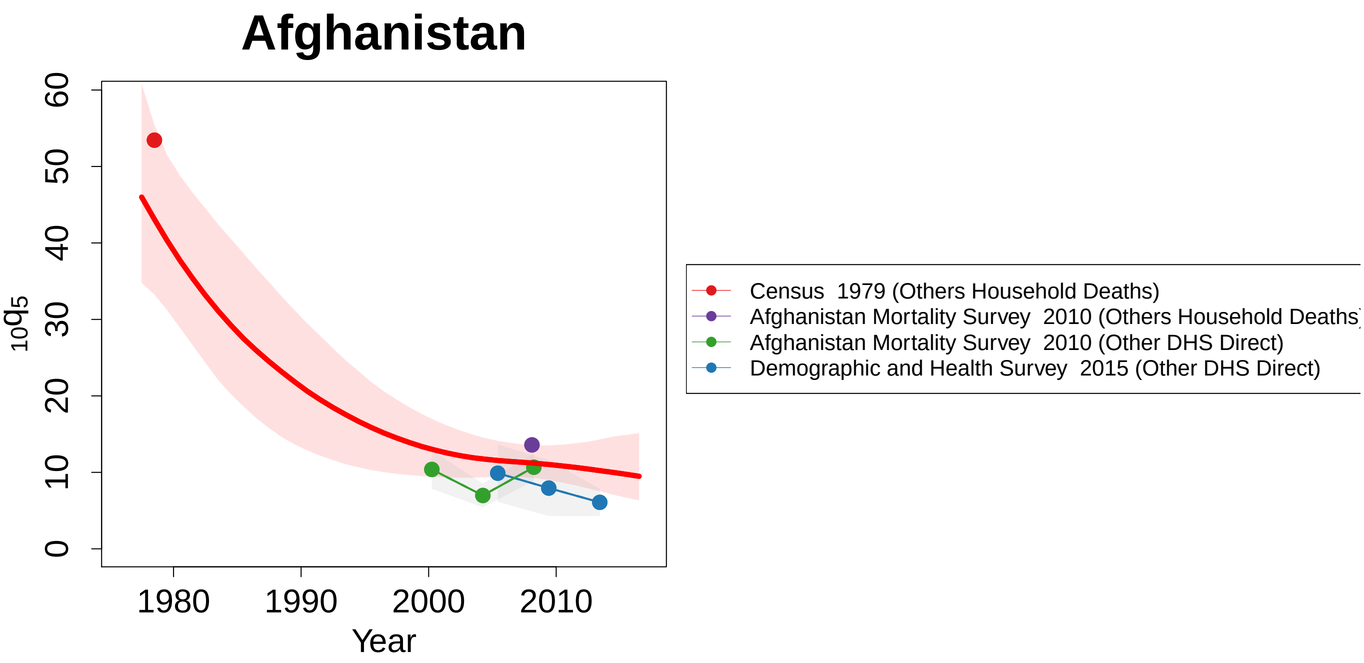

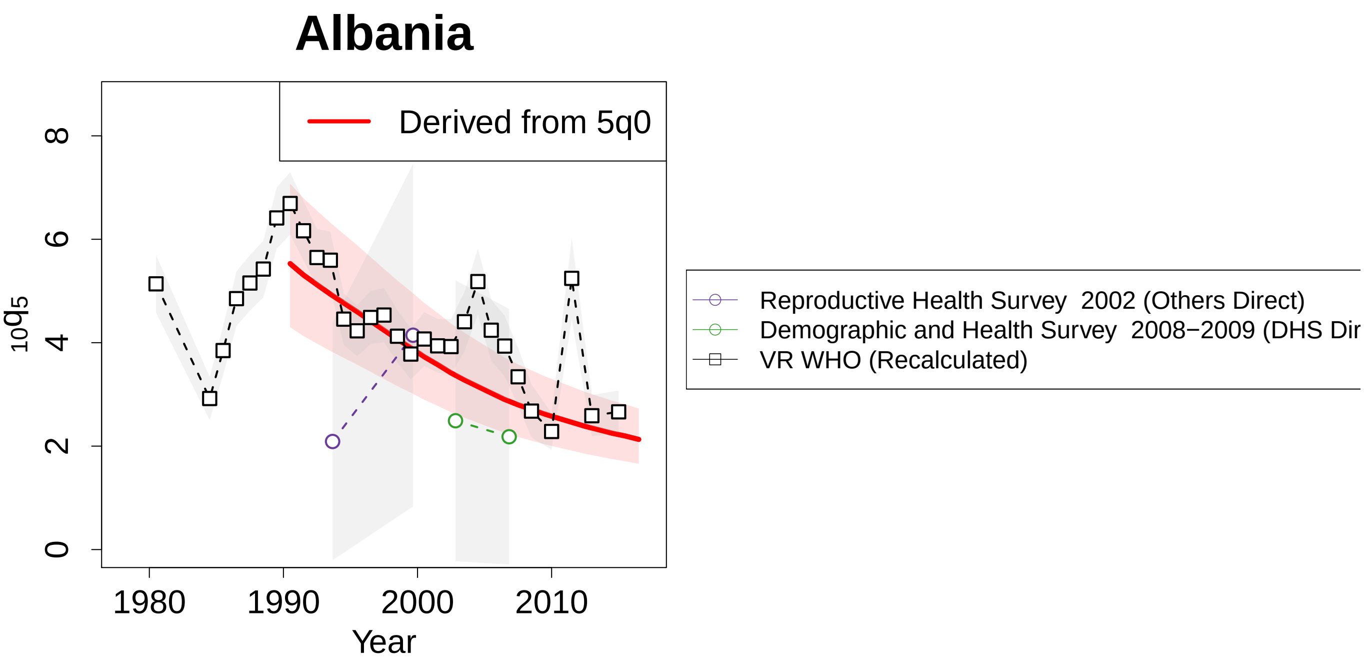

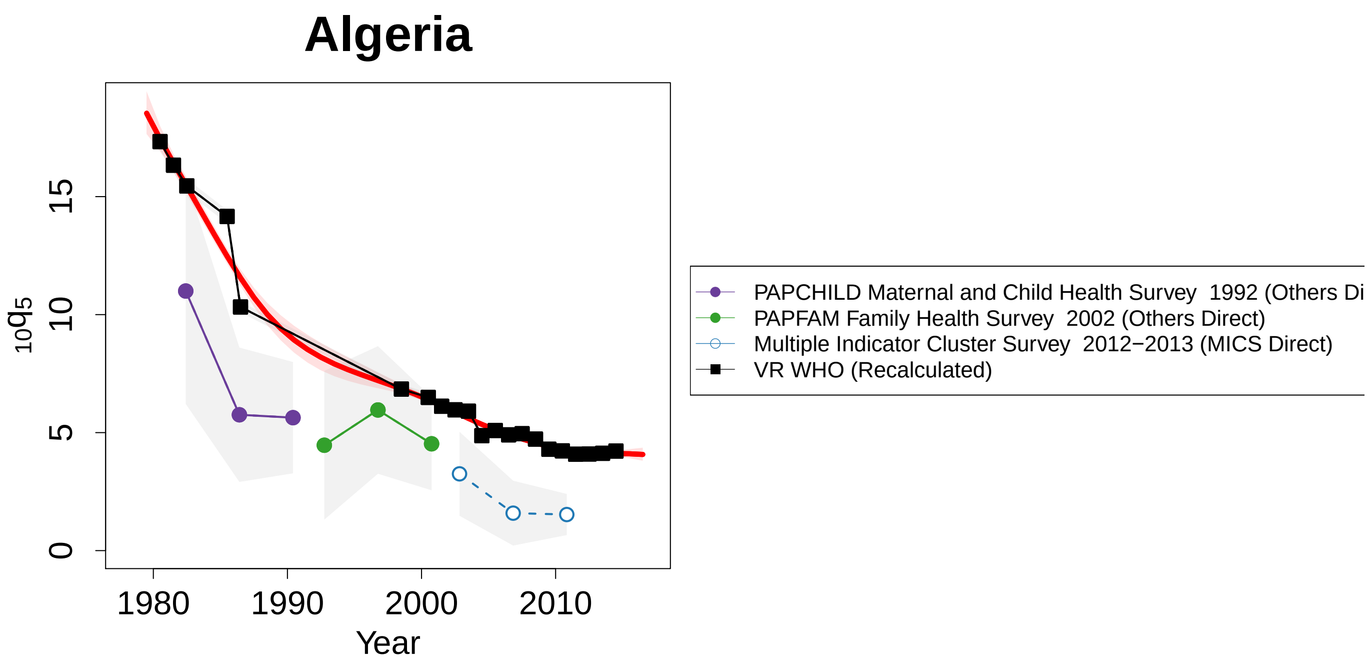
Algeria
15
10
5
0
$_{10}q_5$
1980
1990
2000
2010
Year
PAPCHILD Maternal and Child Health Survey 1992 (Others Di
PAPFAM Family Health Survey 2002 (Others Direct)
Multiple Indicator Cluster Survey 2012−2013 (MICS Direct)
VR WHO (Recalculated)

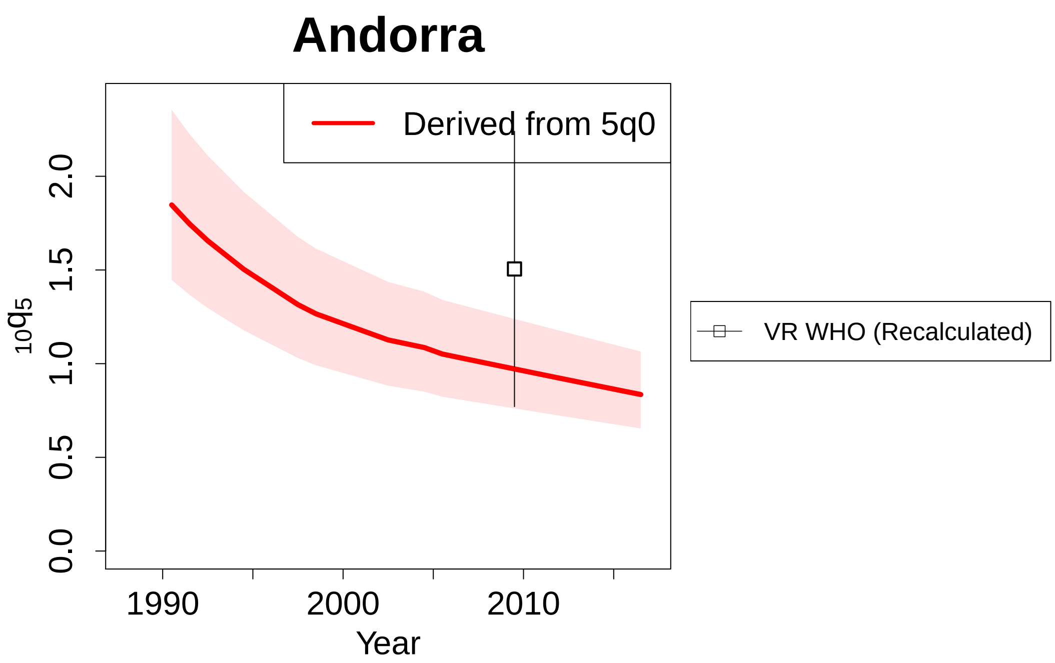
Andorra
Derived from 5q0
2.0
1.5
1.0
0.5
0.0
$_{10}q_5$
1990
2000
2010
Year
VR WHO (Recalculated)

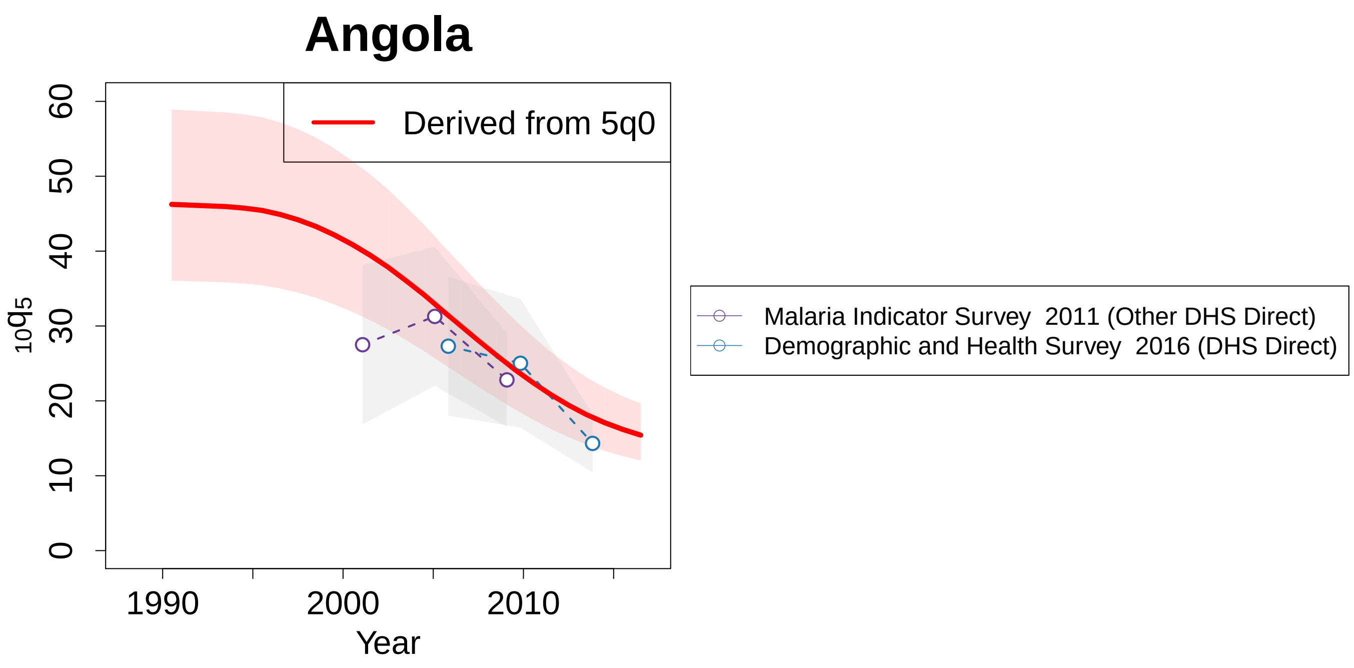
Angola
Derived from 5q0
Malaria Indicator Survey 2011 (Other DHS Direct)
Demographic and Health Survey 2016 (DHS Direct)
$_{10}q_5$
0
10
20
30
40
50
60
1990
2000
2010
Year

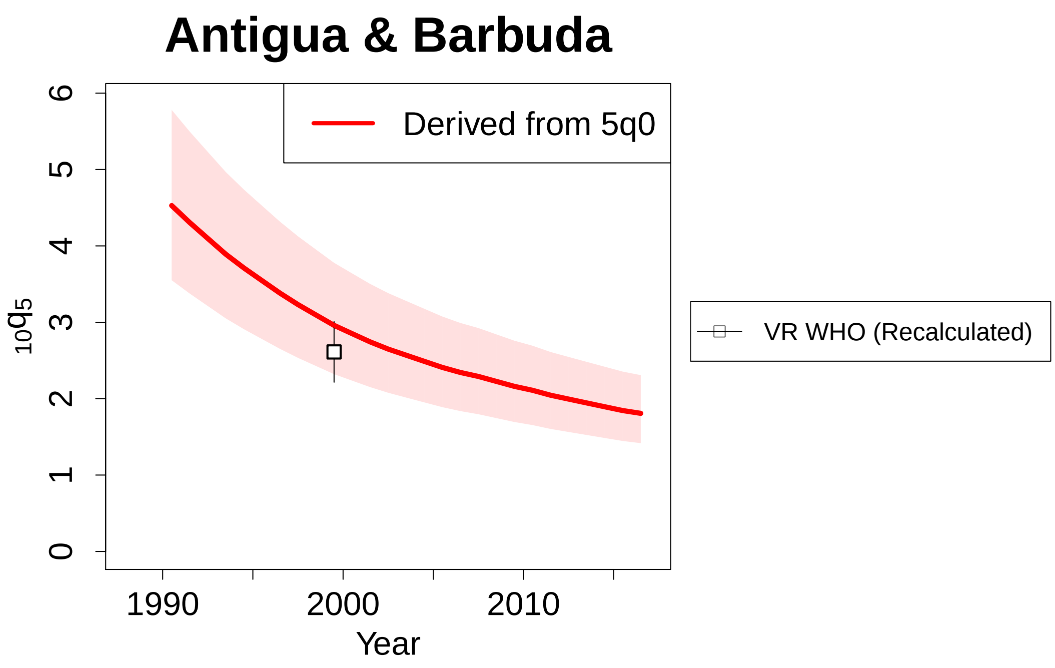
Antigua & Barbuda
Derived from 5q0
VR WHO (Recalculated)
$_{10}q_5$
0
1
2
3
4
5
6
1990
2000
2010
Year

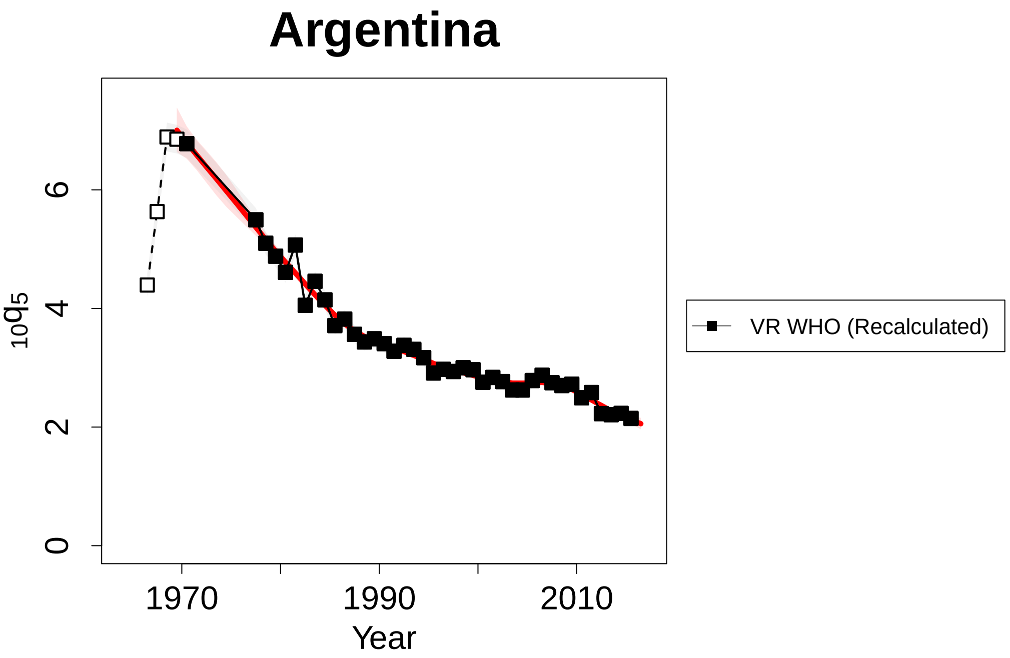
Argentina
0
2
4
6
$_{10}q_5$
1970
1990
2010
Year
VR WHO (Recalculated)

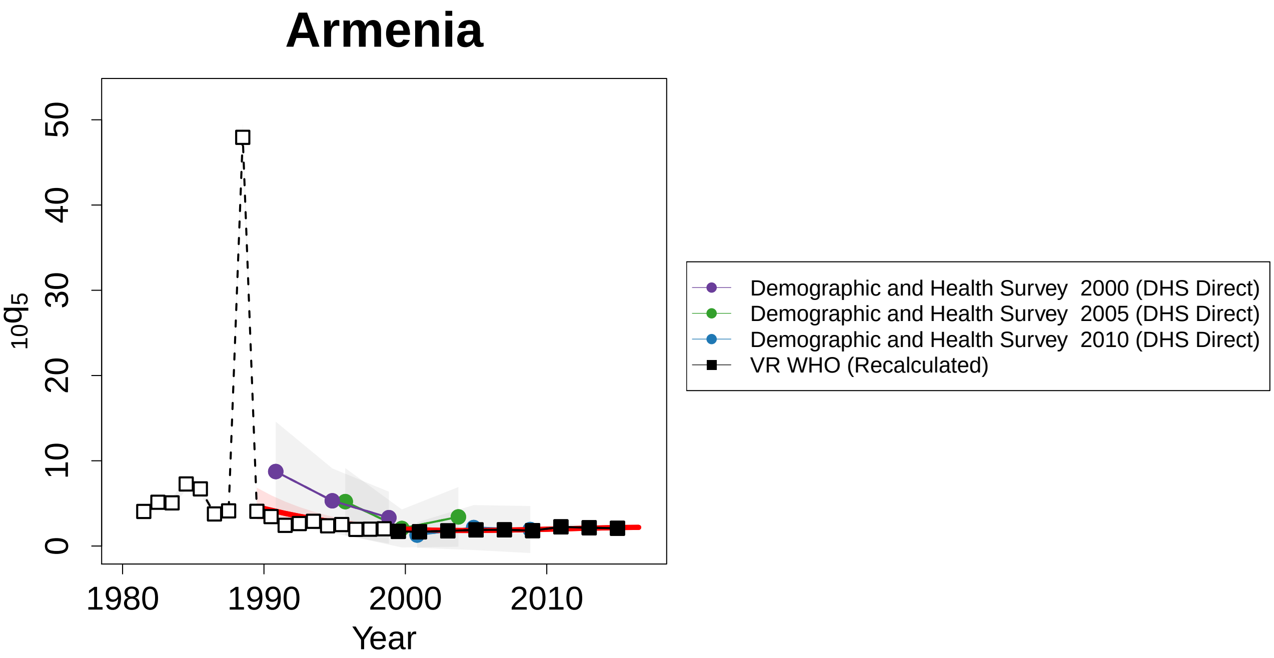
Armenia
0
10
20
30
40
50
$_{10}q_5$
1980
1990
2000
2010
Year
Demographic and Health Survey 2000 (DHS Direct)
Demographic and Health Survey 2005 (DHS Direct)
Demographic and Health Survey 2010 (DHS Direct)
VR WHO (Recalculated)

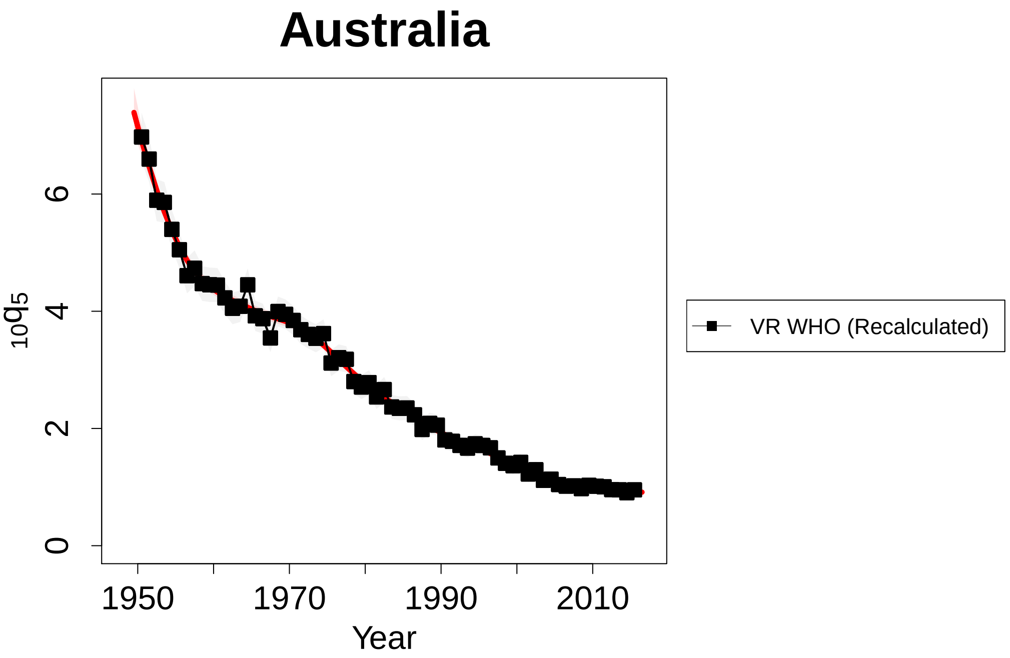
Australia
$_{10}q_5$
0
2
4
6
1950
1970
1990
2010
Year
VR WHO (Recalculated)

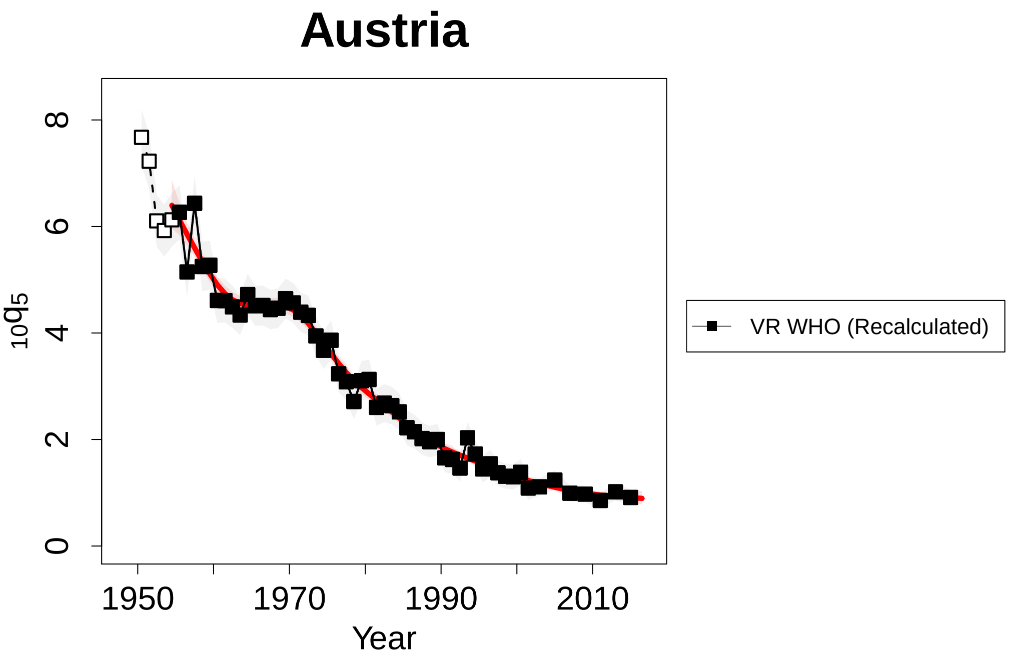
Austria
$_{10}q_5$
0
2
4
6
8
1950
1970
1990
2010
Year
VR WHO (Recalculated)

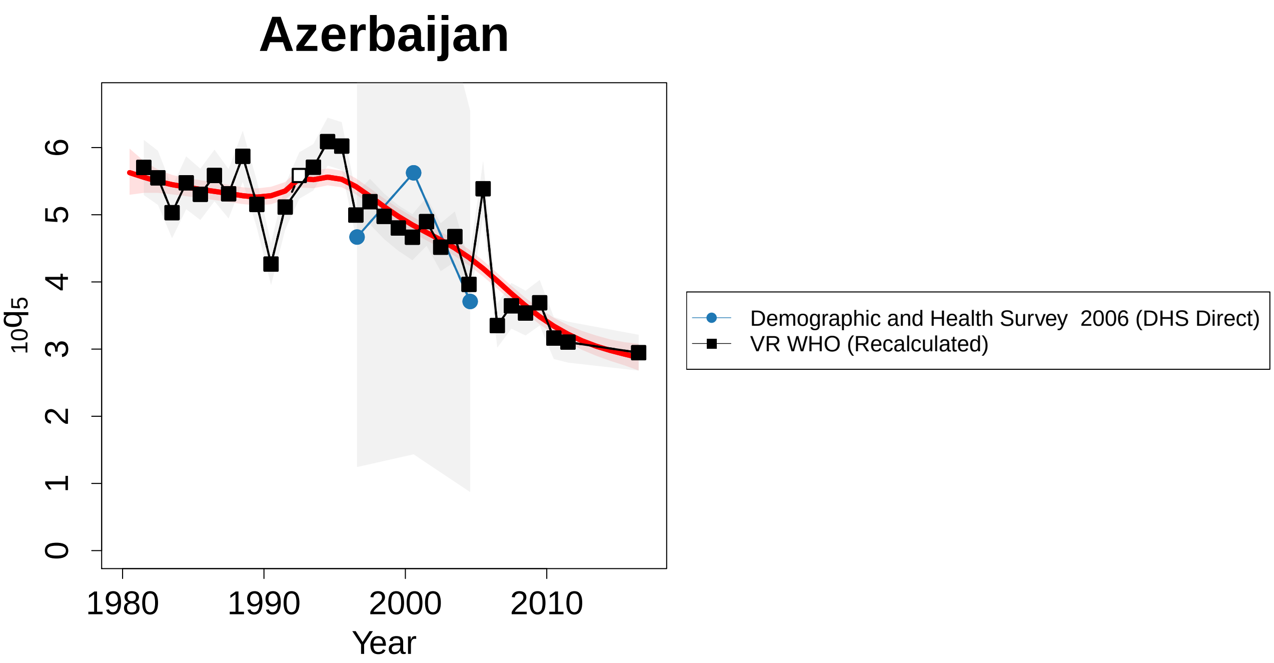
Azerbaijan
$_{10}q_5$
0
1
2
3
4
5
6
1980
1990
2000
2010
Year
Demographic and Health Survey 2006 (DHS Direct)
VR WHO (Recalculated)

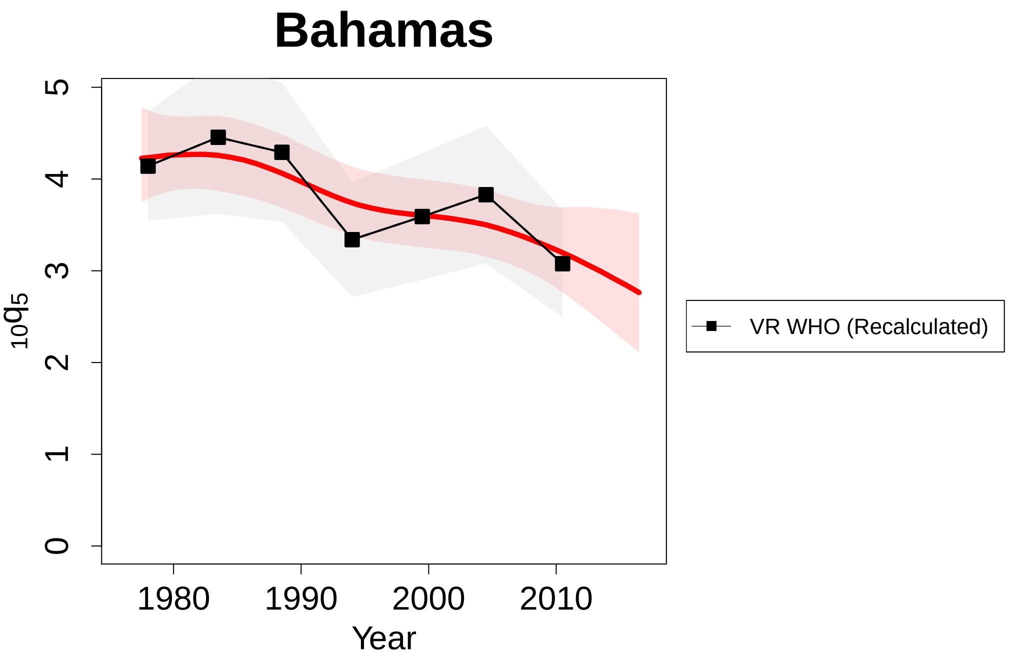
Bahamas
$_{10}q_5$
0
1
2
3
4
5
1980
1990
2000
2010
Year
VR WHO (Recalculated)

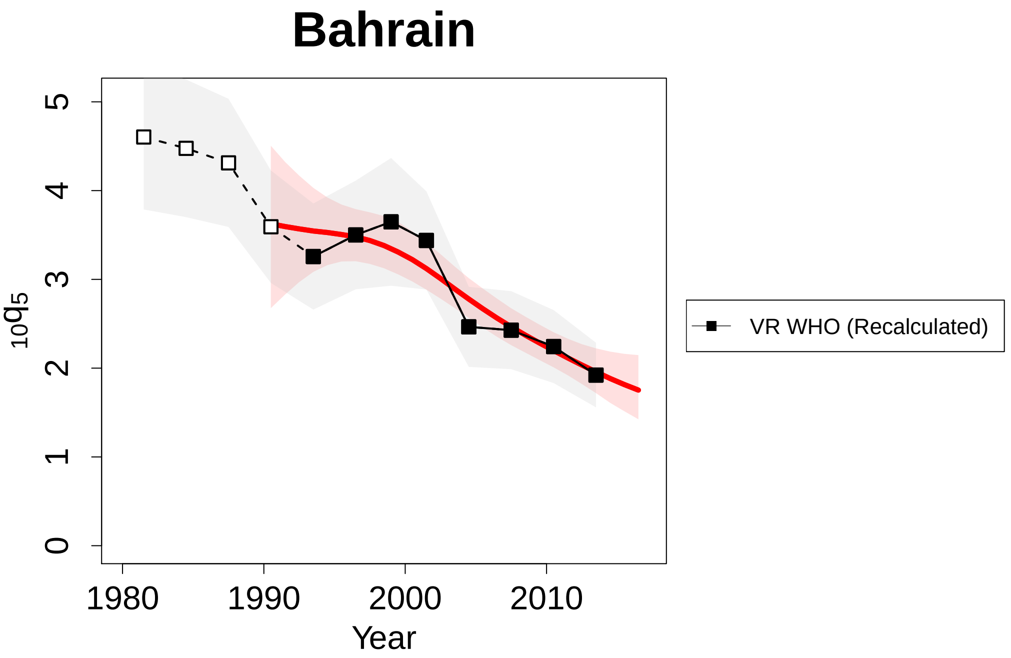
Bahrain
$_{10}q_5$
0
1
2
3
4
5
1980
1990
2000
2010
Year
VR WHO (Recalculated)

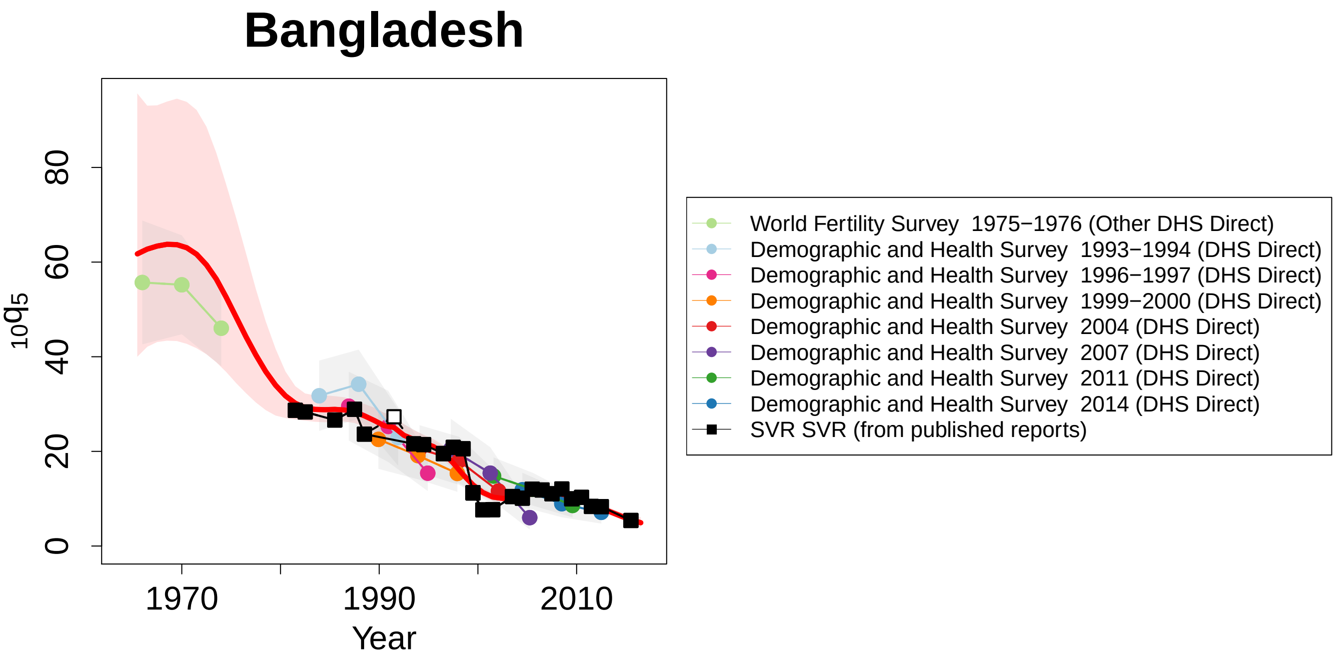
Bangladesh
$_{10}q_5$
0
20
40
60
80
1970
1990
2010
Year
World Fertility Survey 1975−1976 (Other DHS Direct)
Demographic and Health Survey 1993−1994 (DHS Direct)
Demographic and Health Survey 1996−1997 (DHS Direct)
Demographic and Health Survey 1999−2000 (DHS Direct)
Demographic and Health Survey 2004 (DHS Direct)
Demographic and Health Survey 2007 (DHS Direct)
Demographic and Health Survey 2011 (DHS Direct)
Demographic and Health Survey 2014 (DHS Direct)
SVR SVR (from published reports)

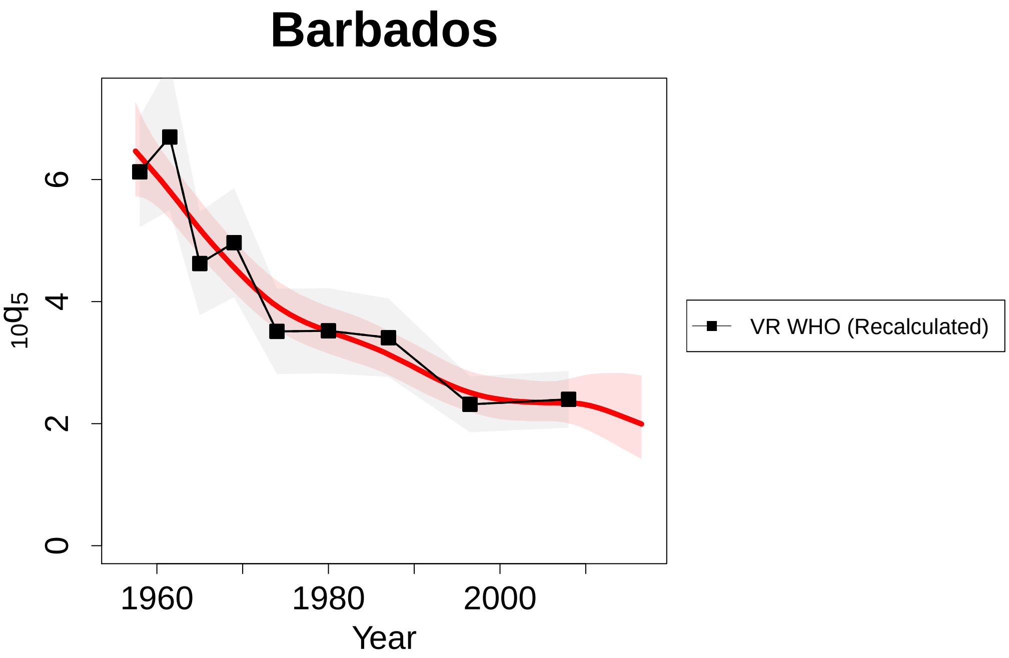
Barbados
0
2
4
6
$_{10}q_5$
1960
1980
2000
Year
VR WHO (Recalculated)

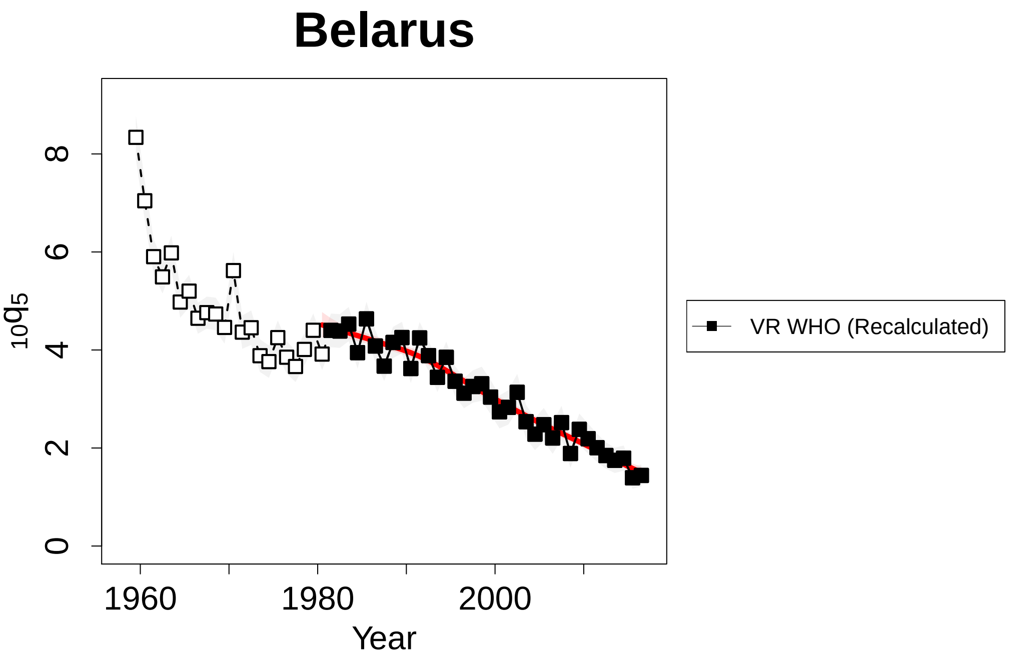
Belarus
0
2
4
6
8
$_{10}q_5$
1960
1980
2000
Year
VR WHO (Recalculated)

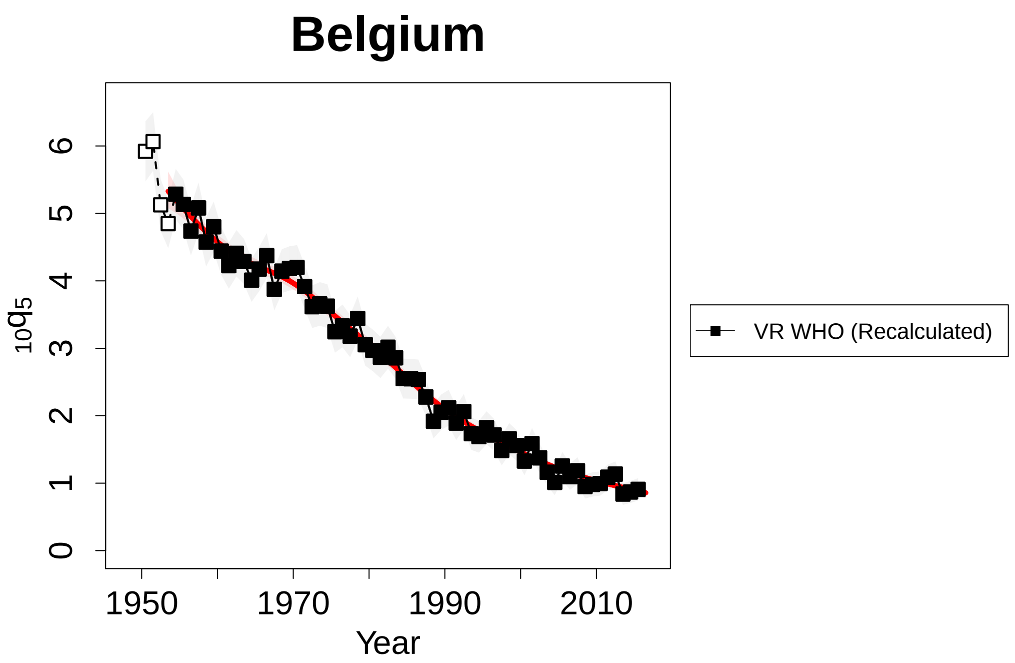
Belgium
$_{10}q_5$
0
1
2
3
4
5
6
1950
1970
1990
2010
Year
VR WHO (Recalculated)

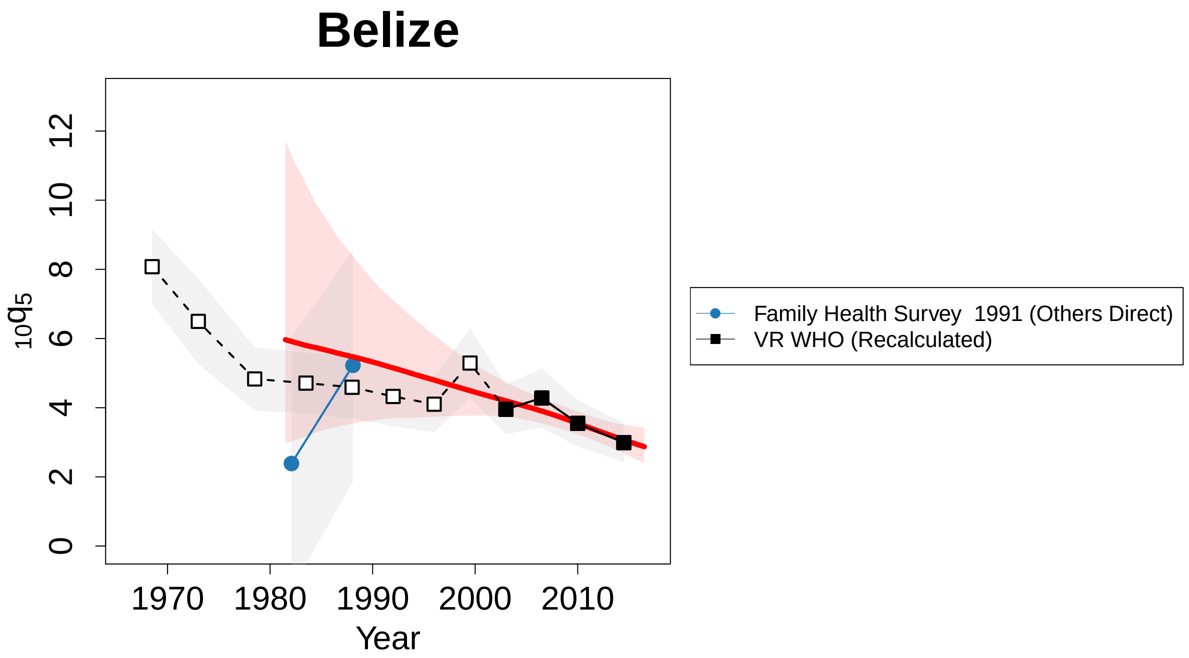
Belize
$_{10}q_5$
0
2
4
6
8
10
12
1970
1980
1990
2000
2010
Year
Family Health Survey 1991 (Others Direct)
VR WHO (Recalculated)

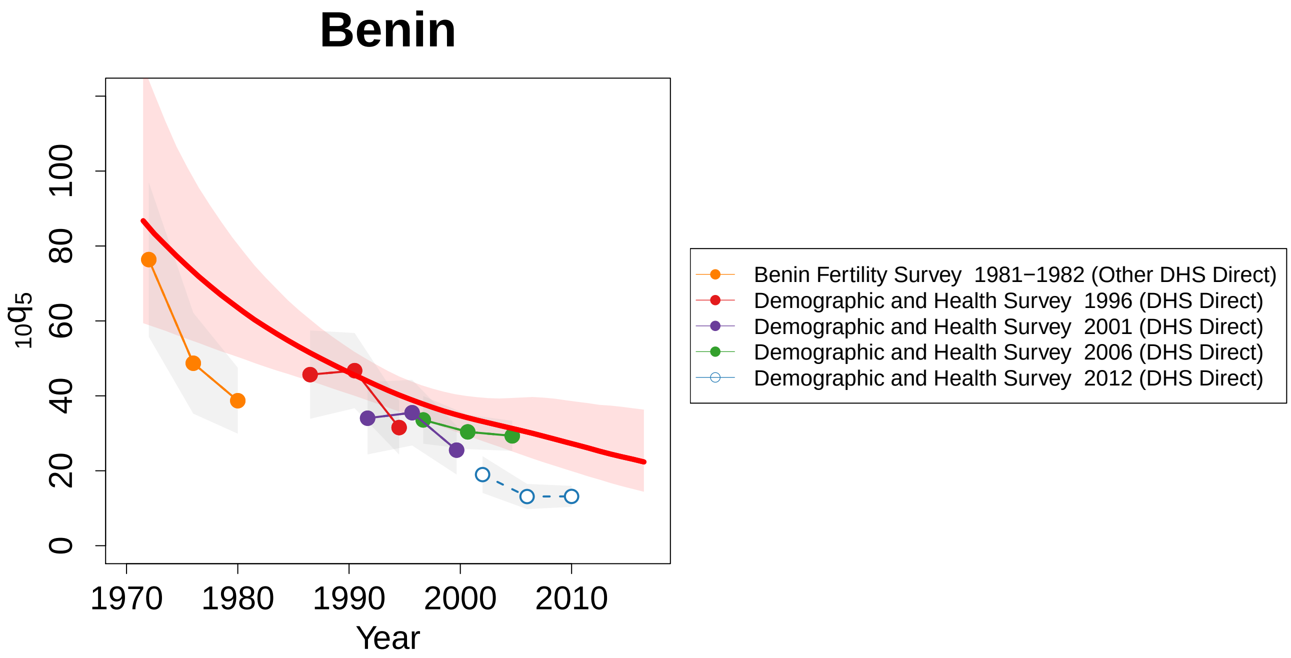
Benin
$_{10}q_5$
0
20
40
60
80
100
1970
1980
1990
2000
2010
Year
Benin Fertility Survey 1981−1982 (Other DHS Direct)
Demographic and Health Survey 1996 (DHS Direct)
Demographic and Health Survey 2001 (DHS Direct)
Demographic and Health Survey 2006 (DHS Direct)
Demographic and Health Survey 2012 (DHS Direct)

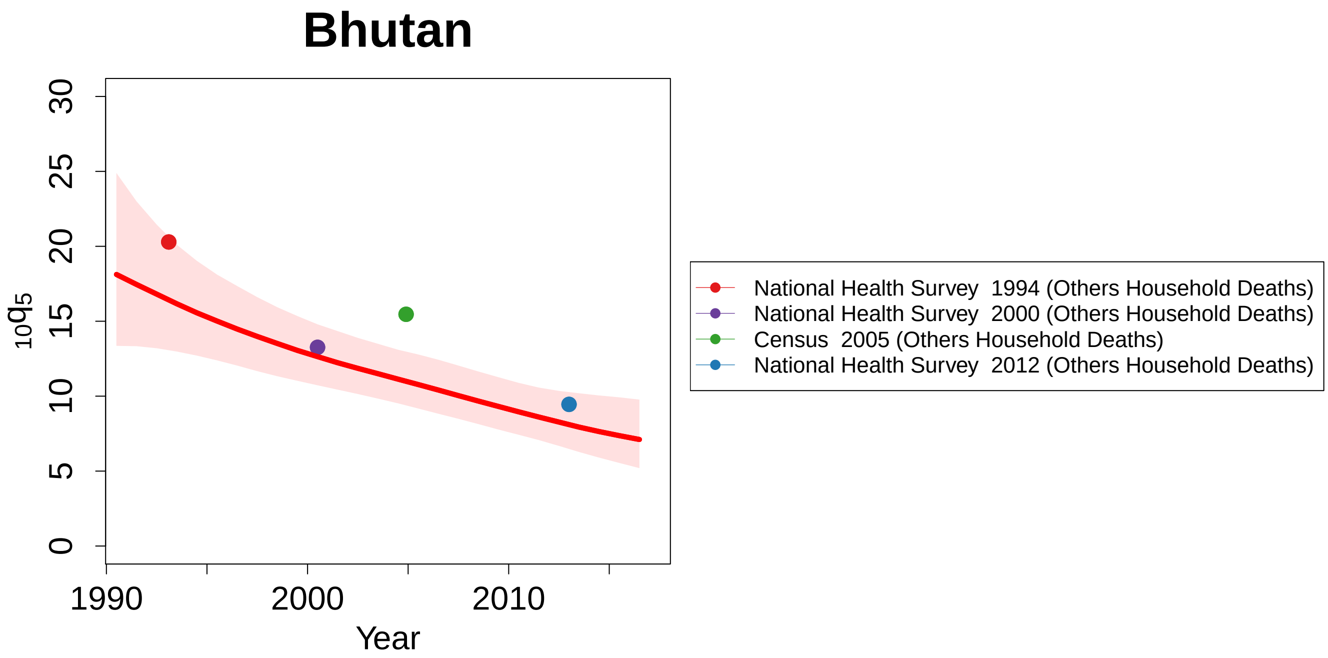
Bhutan
$_{10}q_5$
0
5
10
15
20
25
30
1990
2000
2010
Year
National Health Survey 1994 (Others Household Deaths)
National Health Survey 2000 (Others Household Deaths)
Census 2005 (Others Household Deaths)
National Health Survey 2012 (Others Household Deaths)

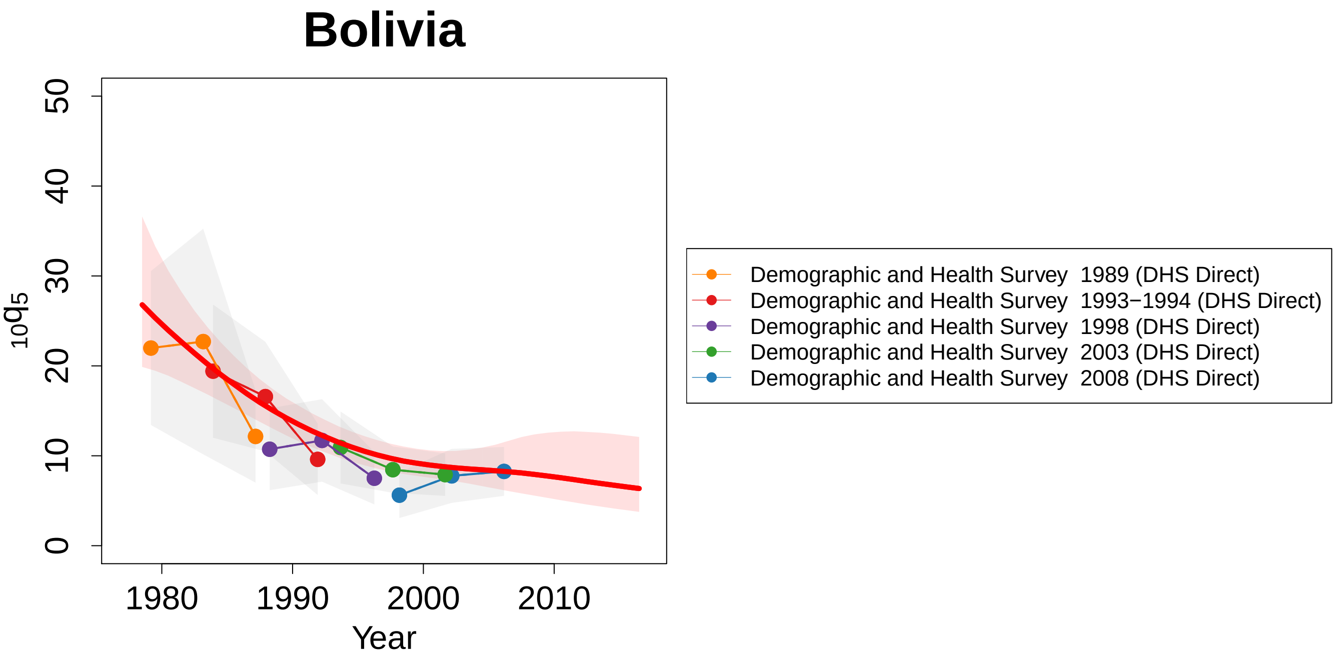
Bolivia
$_{10}q_5$
0
10
20
30
40
50
1980
1990
2000
2010
Year
Demographic and Health Survey 1989 (DHS Direct)
Demographic and Health Survey 1993−1994 (DHS Direct)
Demographic and Health Survey 1998 (DHS Direct)
Demographic and Health Survey 2003 (DHS Direct)
Demographic and Health Survey 2008 (DHS Direct)

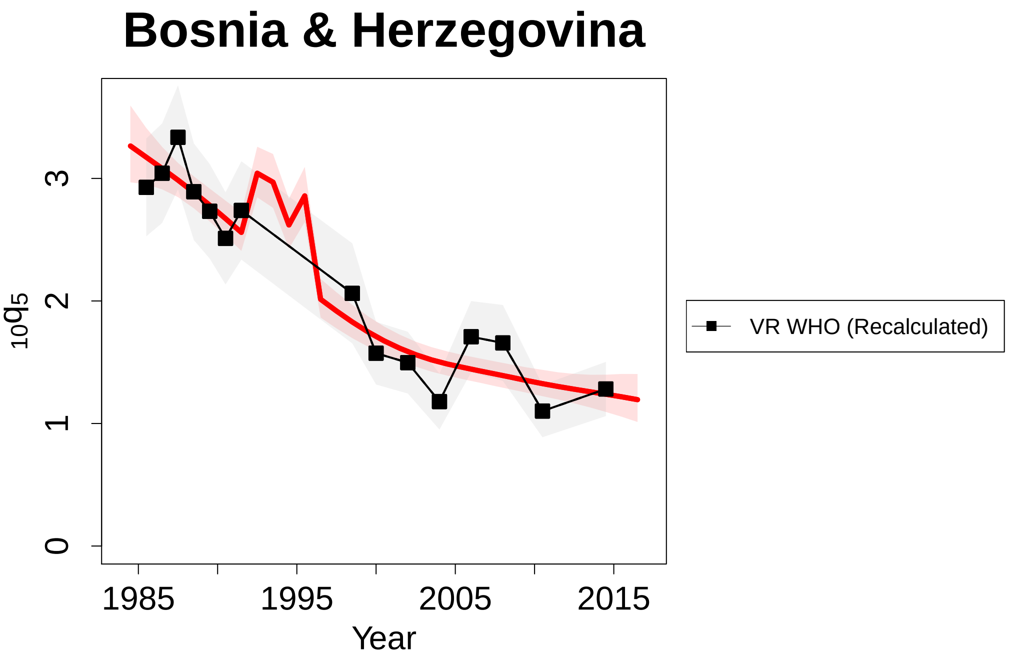
Bosnia & Herzegovina
$_{10}q_5$
0
1
2
3
1985
1995
2005
2015
Year
VR WHO (Recalculated)

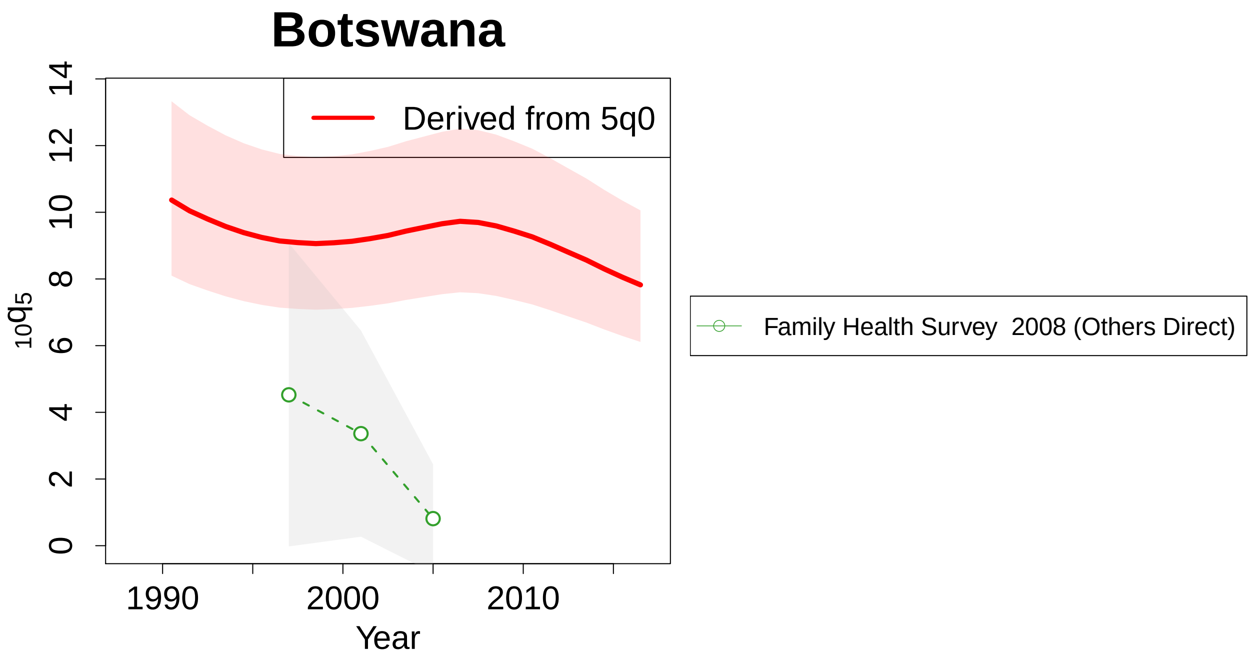
Botswana
Derived from 5q0
Family Health Survey 2008 (Others Direct)
$_{10}q_5$
0
2
4
6
8
10
12
14
1990
2000
2010
Year

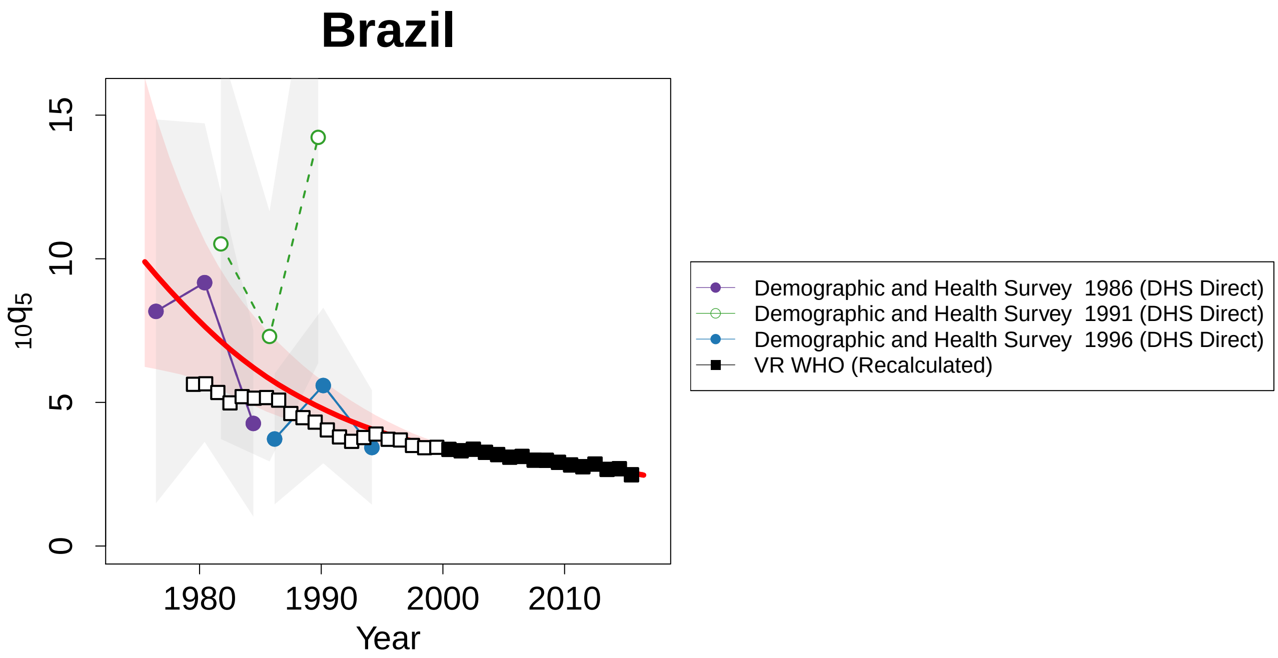
Brazil
Demographic and Health Survey 1986 (DHS Direct)
Demographic and Health Survey 1991 (DHS Direct)
Demographic and Health Survey 1996 (DHS Direct)
VR WHO (Recalculated)
$_{10}q_5$
0
5
10
15
1980
1990
2000
2010
Year

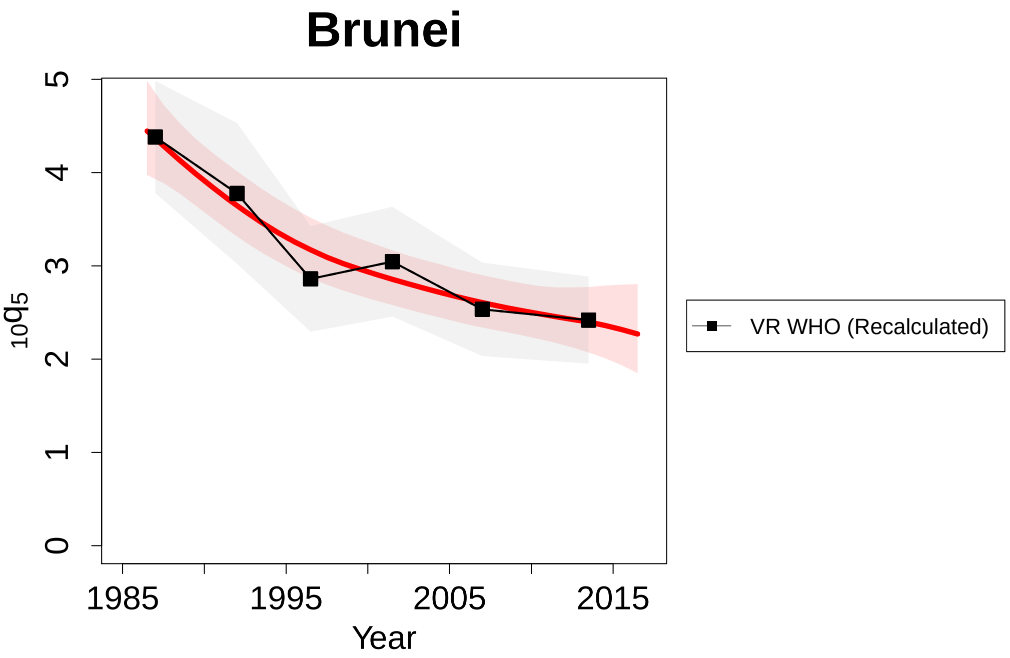
Brunei
5
4
3
2
1
0
$_{10}q_5$
1985
1995
2005
2015
Year
VR WHO (Recalculated)

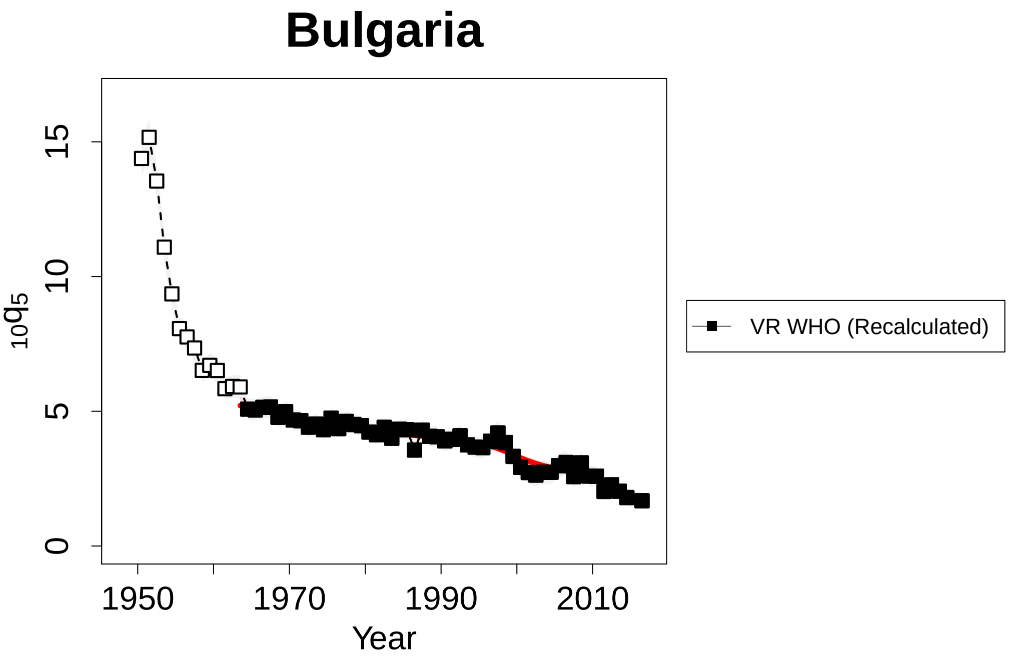
Bulgaria
15
10
5
0
$_{10}q_5$
1950
1970
1990
2010
Year
VR WHO (Recalculated)

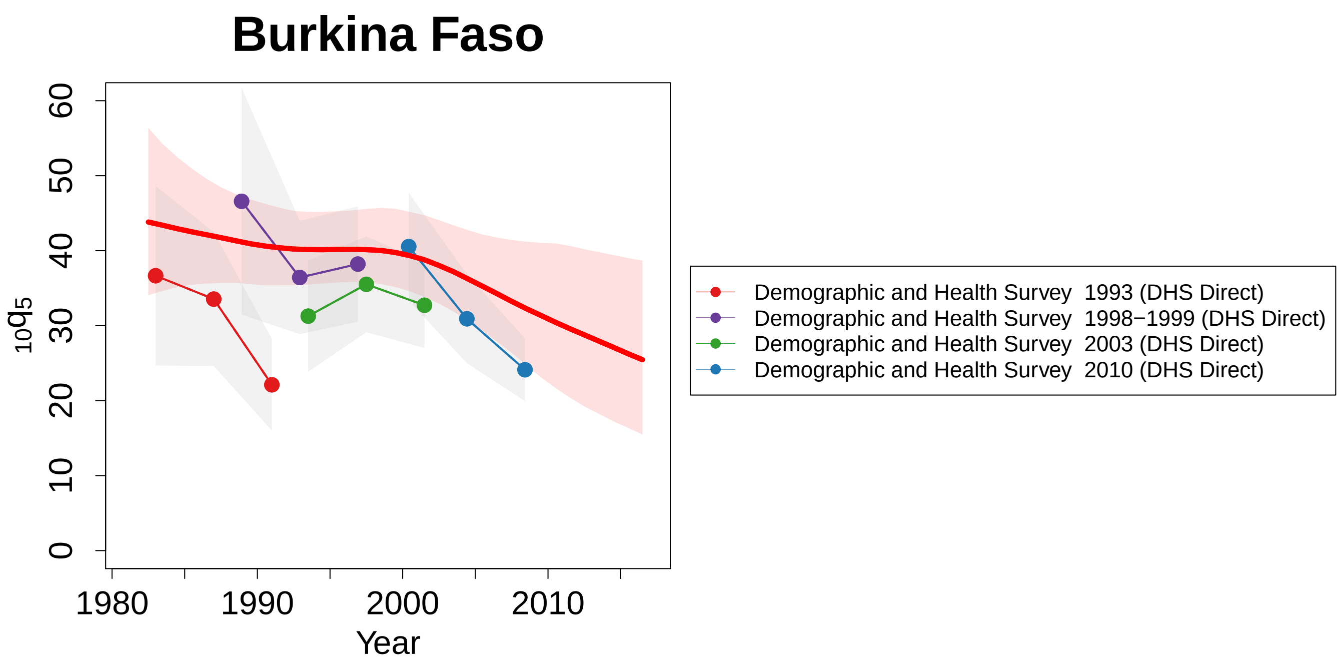
Burkina Faso
60
50
40
30
20
10
0
$_{10}q_5$
1980
1990
2000
2010
Year
Demographic and Health Survey 1993 (DHS Direct)
Demographic and Health Survey 1998−1999 (DHS Direct)
Demographic and Health Survey 2003 (DHS Direct)
Demographic and Health Survey 2010 (DHS Direct)

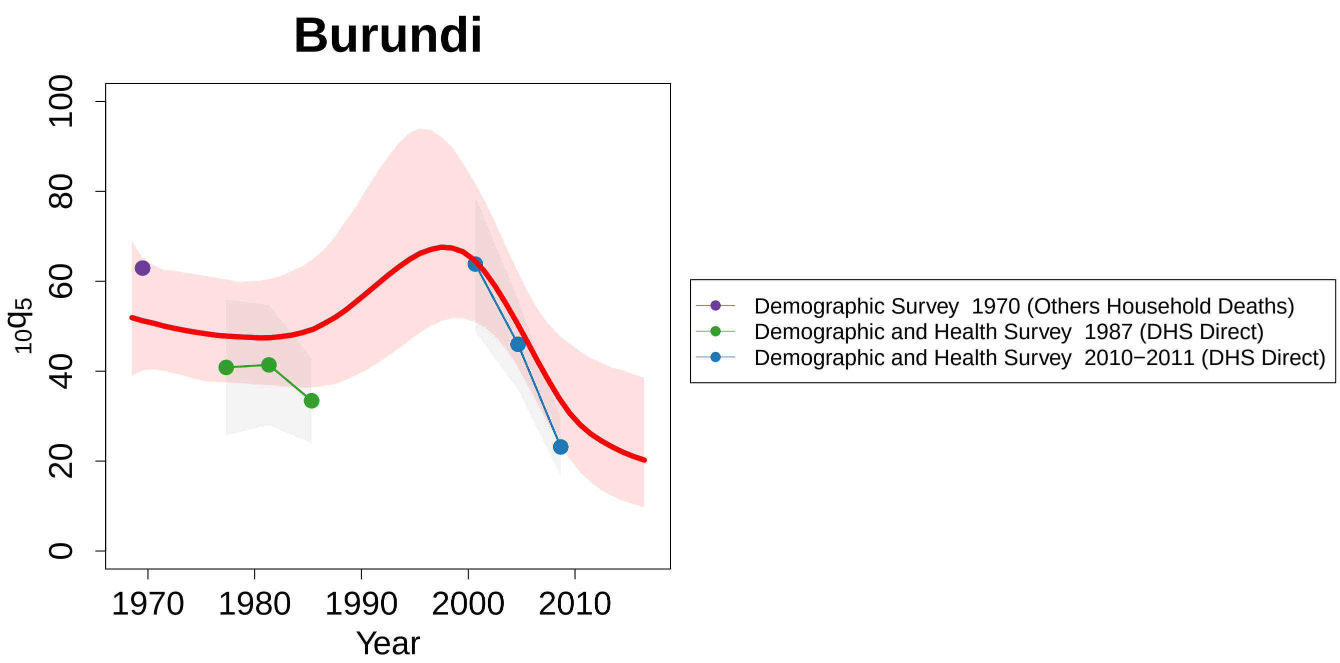
Burundi
100
80
60
40
20
0
$_{10}q_5$
1970
1980
1990
2000
2010
Year
Demographic Survey 1970 (Others Household Deaths)
Demographic and Health Survey 1987 (DHS Direct)
Demographic and Health Survey 2010−2011 (DHS Direct)

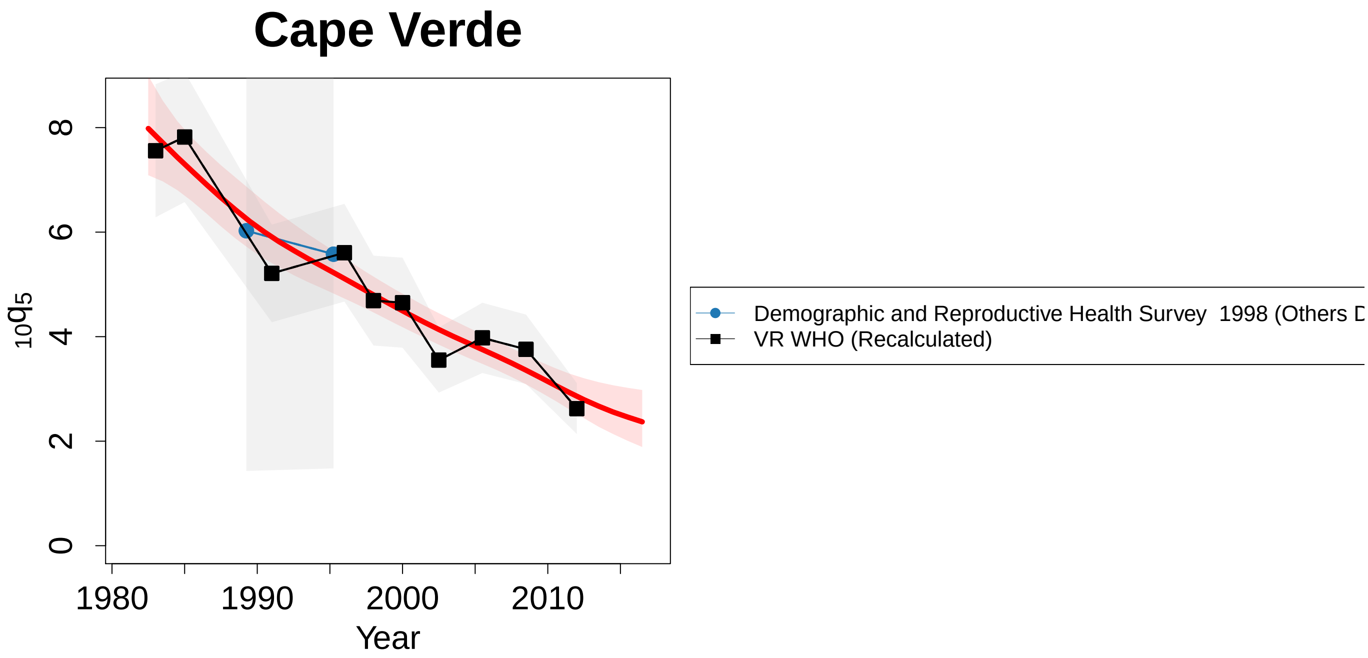
Cape Verde
8
6
4
2
0
$_{10}q_5$
1980
1990
2000
2010
Year
Demographic and Reproductive Health Survey 1998 (Others D
VR WHO (Recalculated)

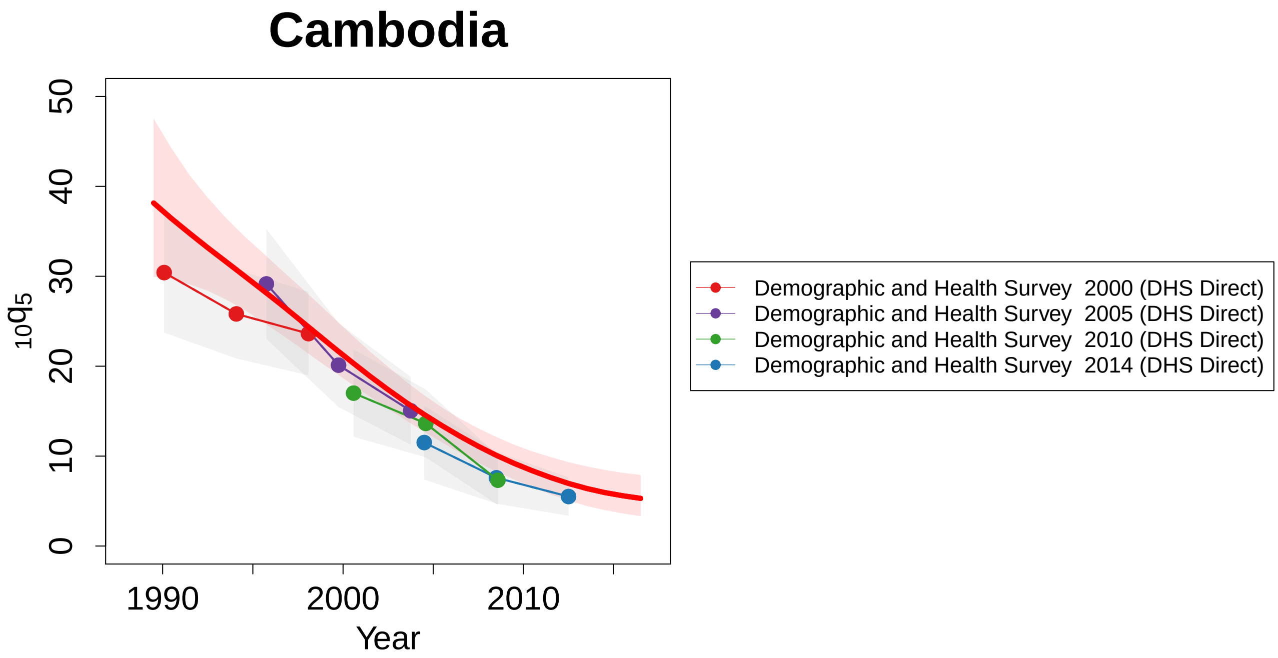
Cambodia
50
40
30
20
10
0
$_{10}q_5$
1990
2000
2010
Year
Demographic and Health Survey 2000 (DHS Direct)
Demographic and Health Survey 2005 (DHS Direct)
Demographic and Health Survey 2010 (DHS Direct)
Demographic and Health Survey 2014 (DHS Direct)

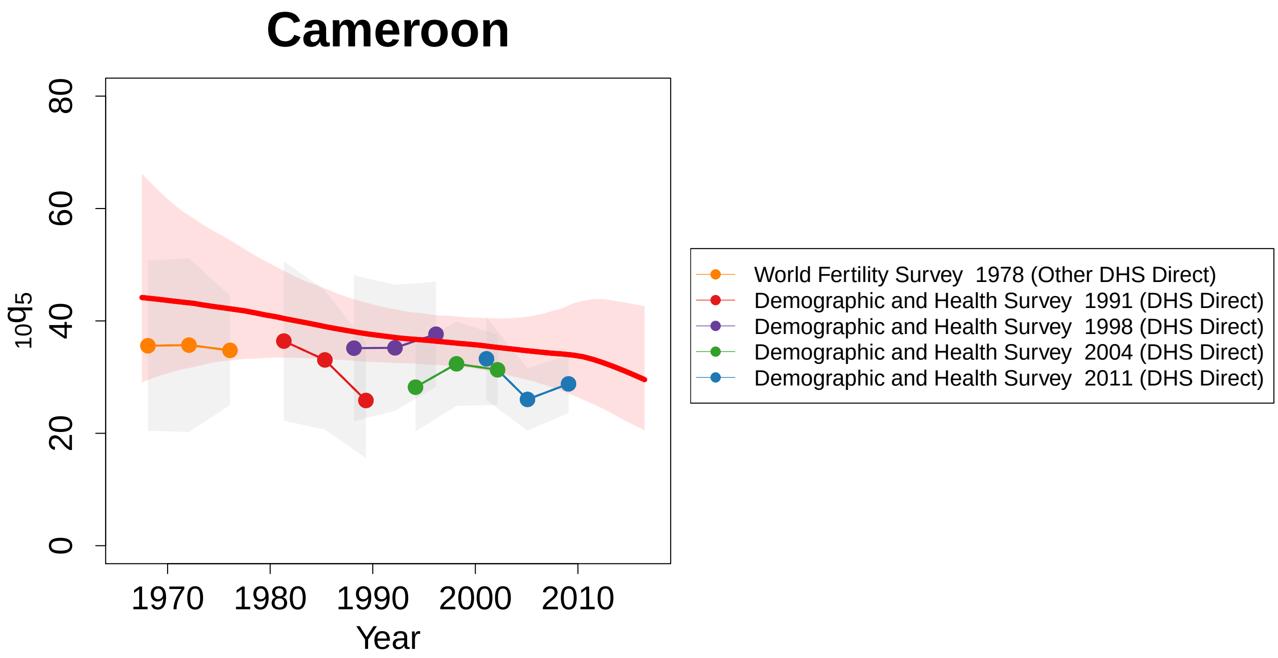
Cameroon
$_{10}q_5$
0
20
40
60
80
1970
1980
1990
2000
2010
Year
World Fertility Survey 1978 (Other DHS Direct)
Demographic and Health Survey 1991 (DHS Direct)
Demographic and Health Survey 1998 (DHS Direct)
Demographic and Health Survey 2004 (DHS Direct)
Demographic and Health Survey 2011 (DHS Direct)

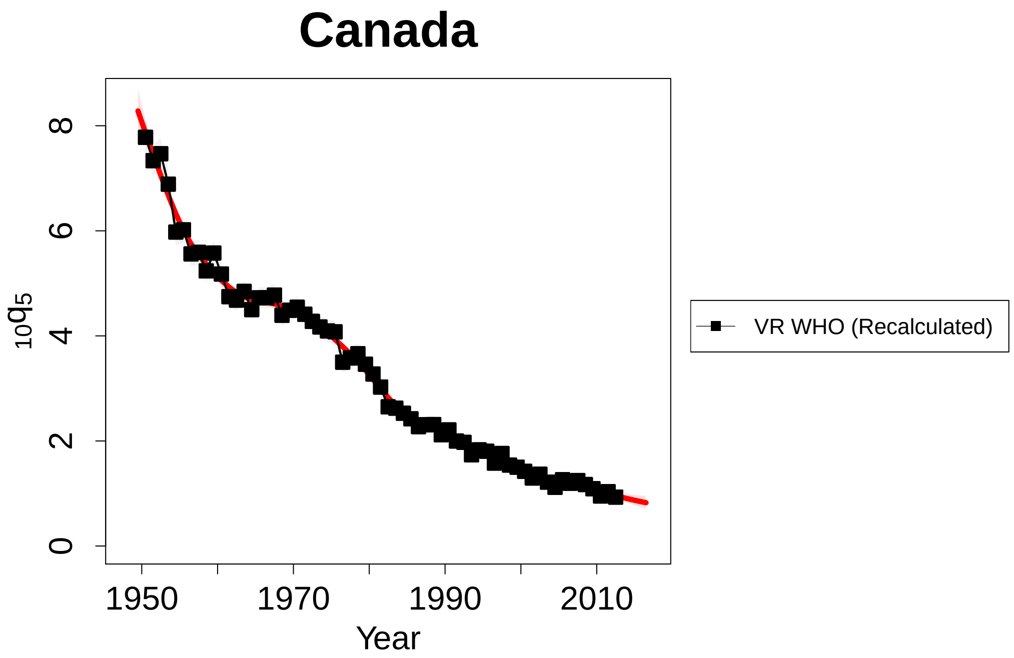
Canada
$_{10}q_5$
0
2
4
6
8
1950
1970
1990
2010
Year
VR WHO (Recalculated)

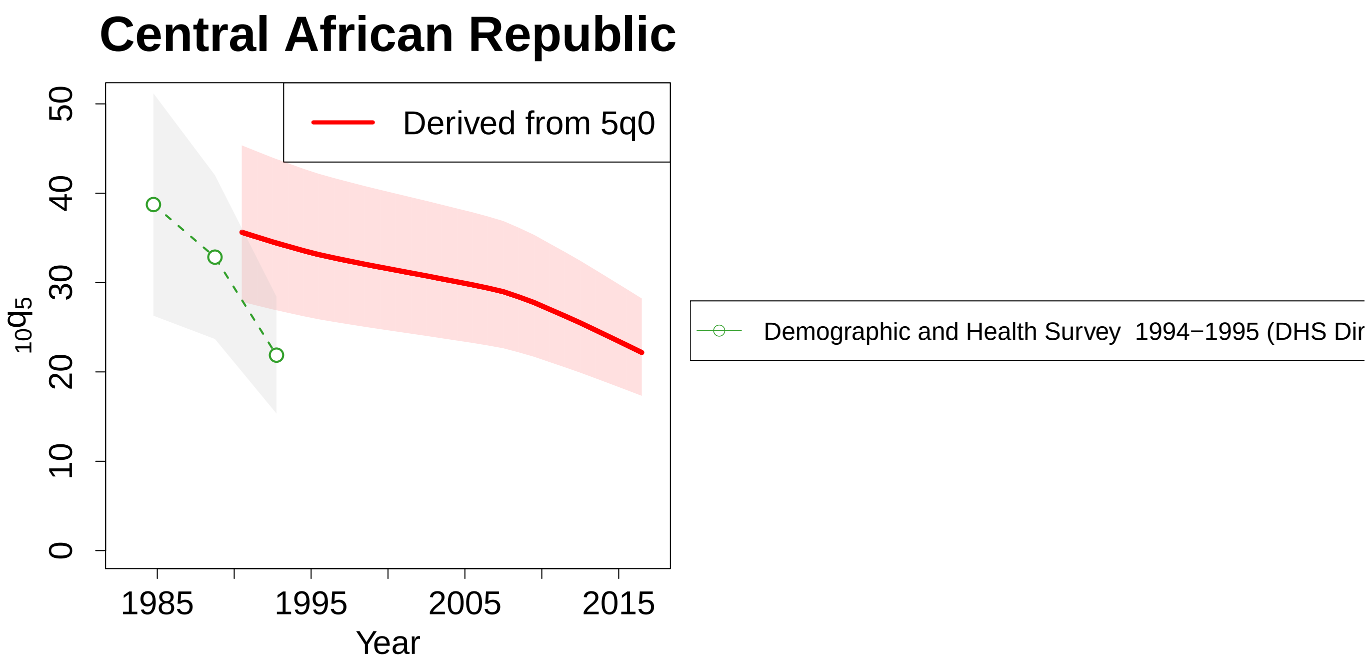
Central African Republic
Derived from 5q0
Demographic and Health Survey 1994−1995 (DHS Dir
$_{10}q_5$
0
10
20
30
40
50
1985
1995
2005
2015
Year

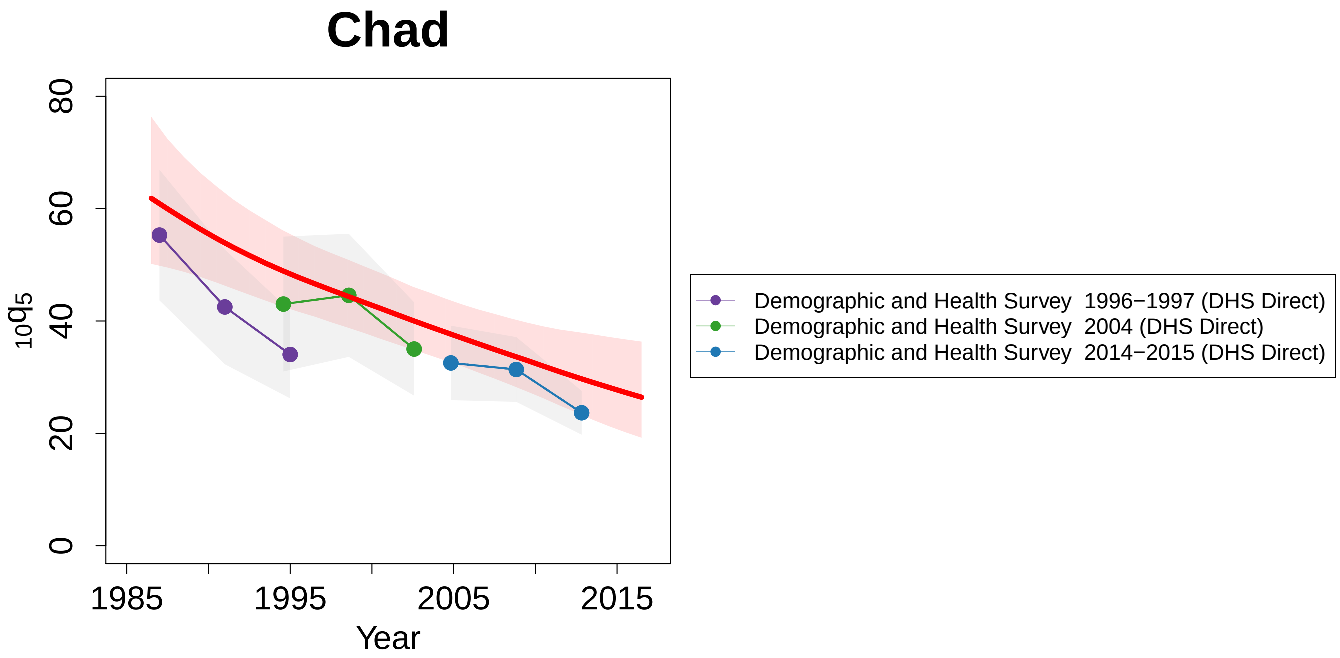
Chad
Demographic and Health Survey 1996−1997 (DHS Direct)
Demographic and Health Survey 2004 (DHS Direct)
Demographic and Health Survey 2014−2015 (DHS Direct)
$_{10}q_5$
0
20
40
60
80
1985
1995
2005
2015
Year

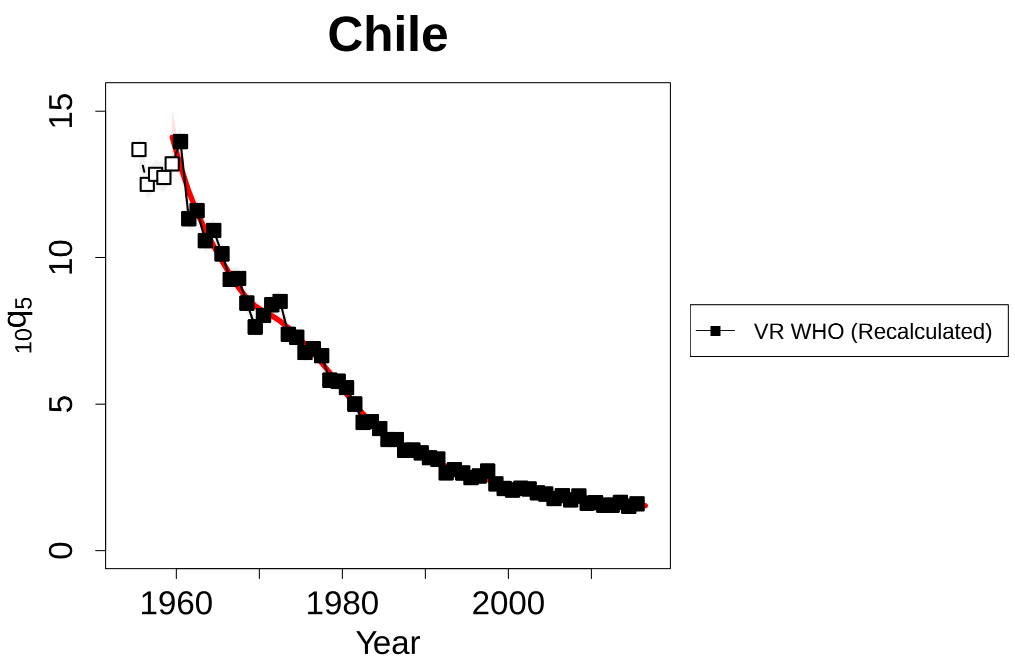
Chile
$_{10}q_5$
0
5
10
15
1960
1980
2000
Year
VR WHO (Recalculated)

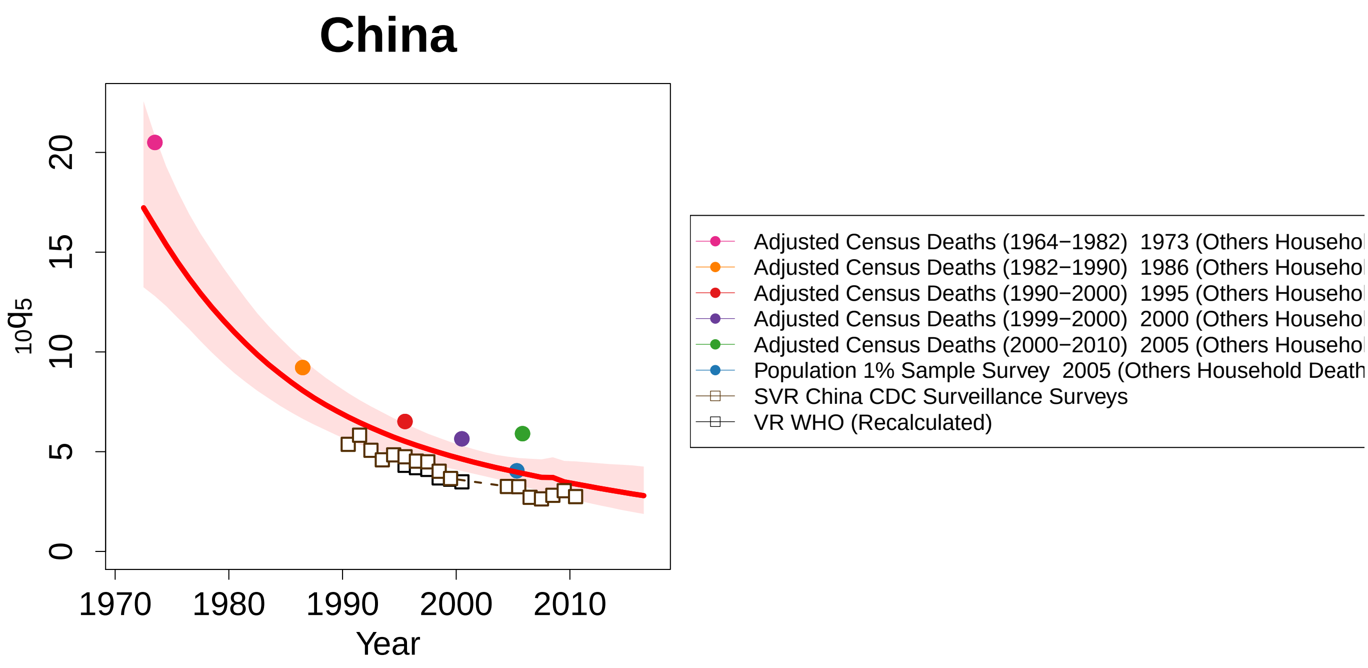
China
$_{10}q_5$
0
5
10
15
20
1970
1980
1990
2000
2010
Year
Adjusted Census Deaths (1964−1982) 1973 (Others Househol
Adjusted Census Deaths (1982−1990) 1986 (Others Househol
Adjusted Census Deaths (1990−2000) 1995 (Others Househol
Adjusted Census Deaths (1999−2000) 2000 (Others Househol
Adjusted Census Deaths (2000−2010) 2005 (Others Househol
Population 1% Sample Survey 2005 (Others Household Death
SVR China CDC Surveillance Surveys
VR WHO (Recalculated)

Colombia

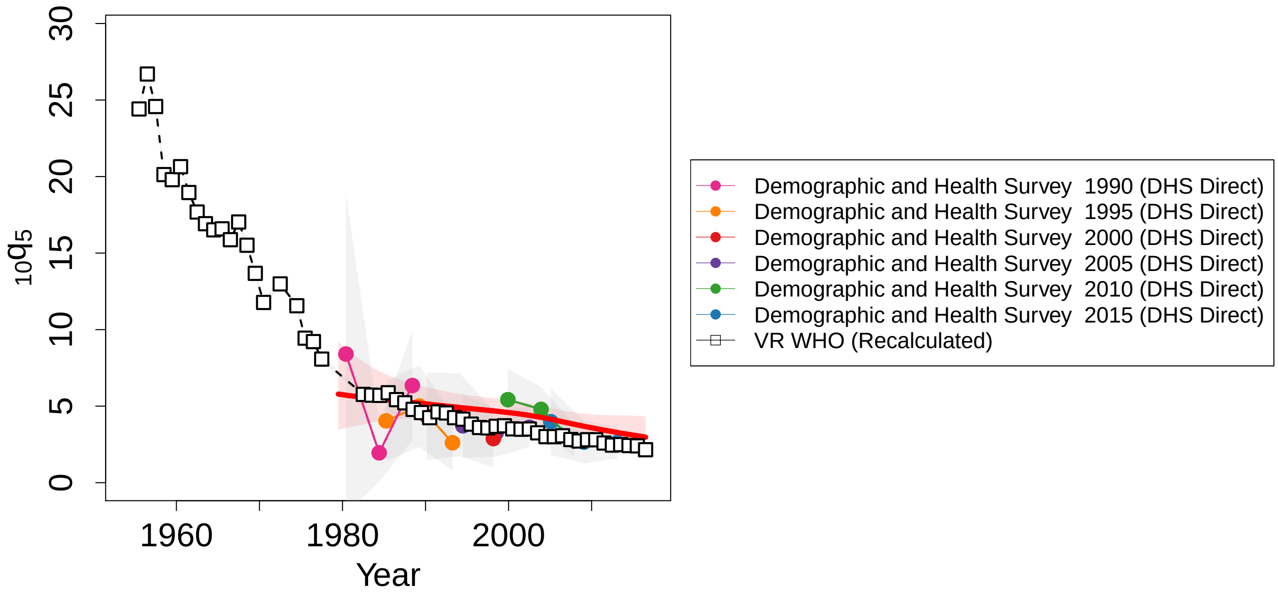

Comoros

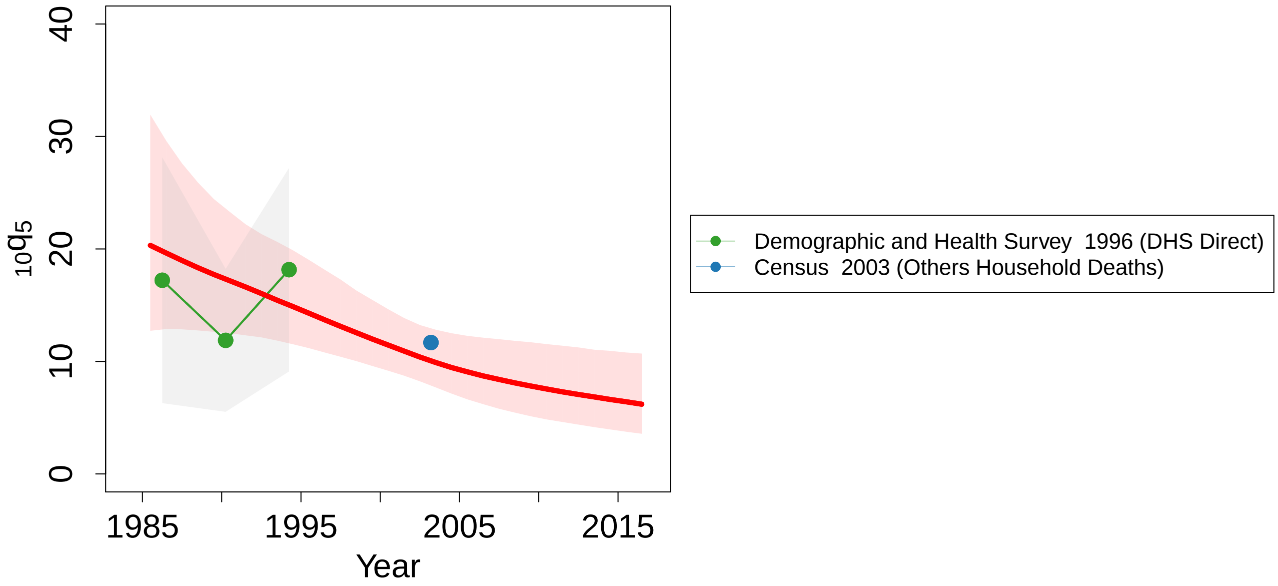

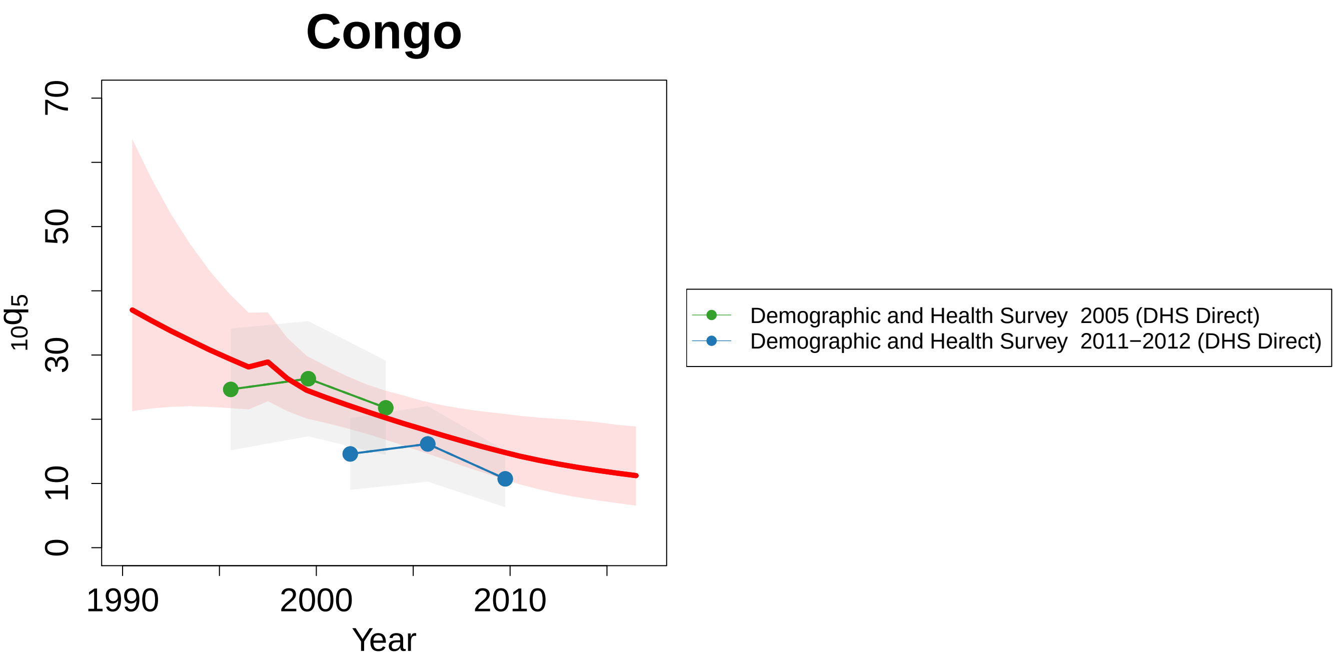
Congo
70
50
30
10
0
$_{10}q_5$
1990
2000
2010
Year
Demographic and Health Survey 2005 (DHS Direct)
Demographic and Health Survey 2011−2012 (DHS Direct)

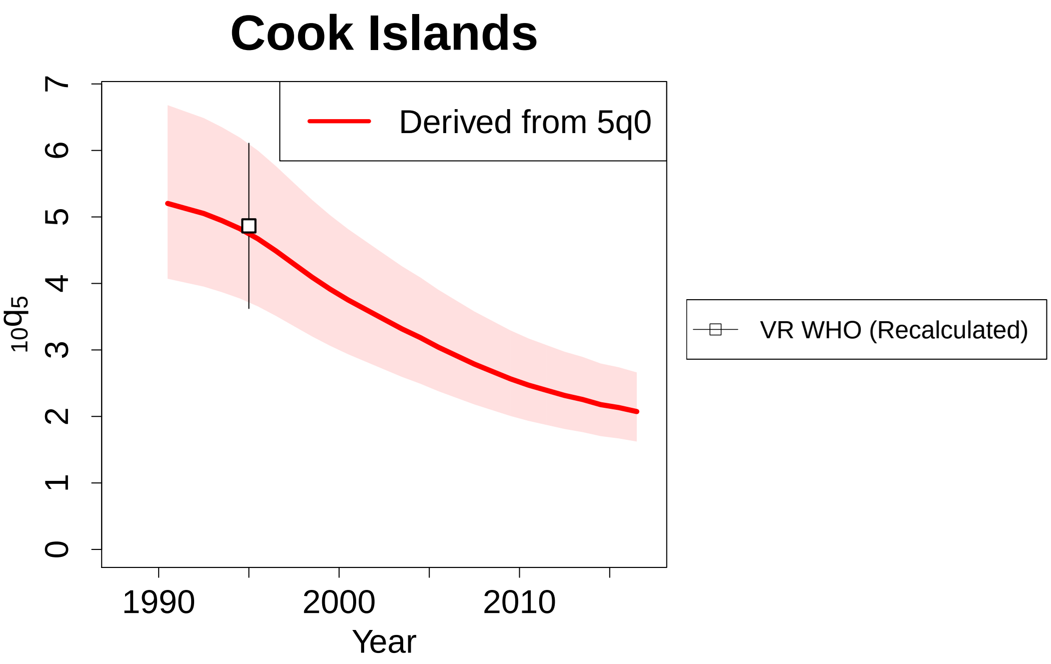
Cook Islands
7
6
5
4
3
2
1
0
$_{10}q_5$
1990
2000
2010
Year
Derived from 5q0
VR WHO (Recalculated)

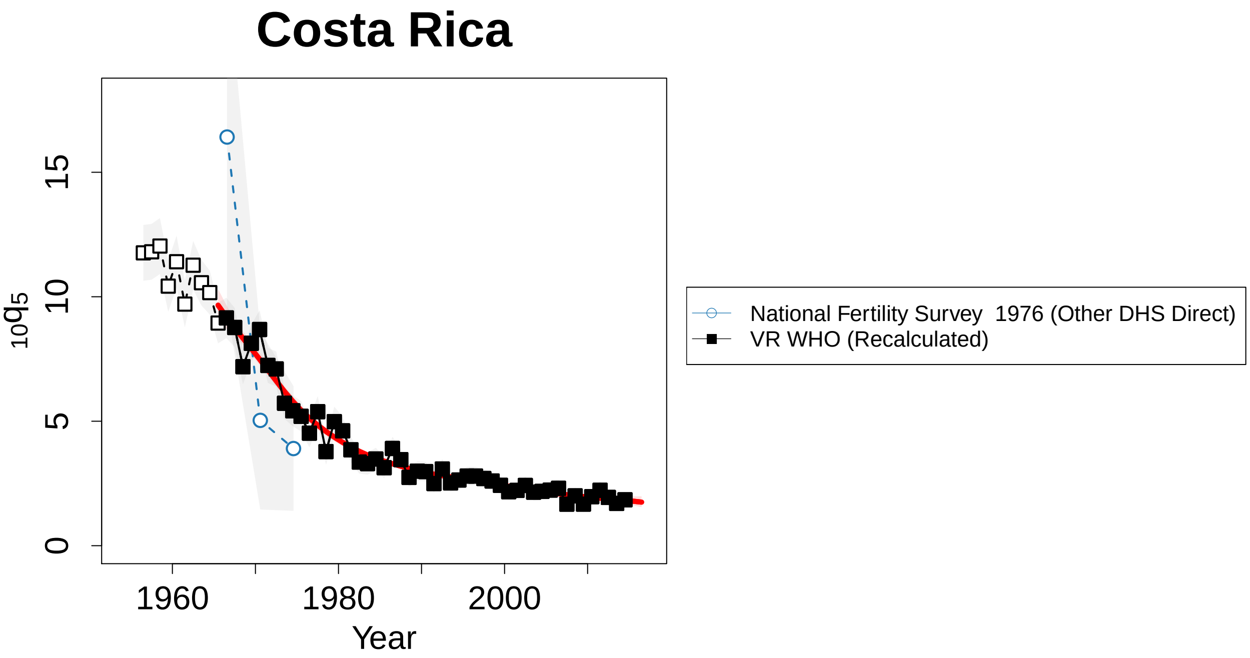
Costa Rica
$_{10}q_5$
0
5
10
15
1960
1980
2000
Year
National Fertility Survey 1976 (Other DHS Direct)
VR WHO (Recalculated)

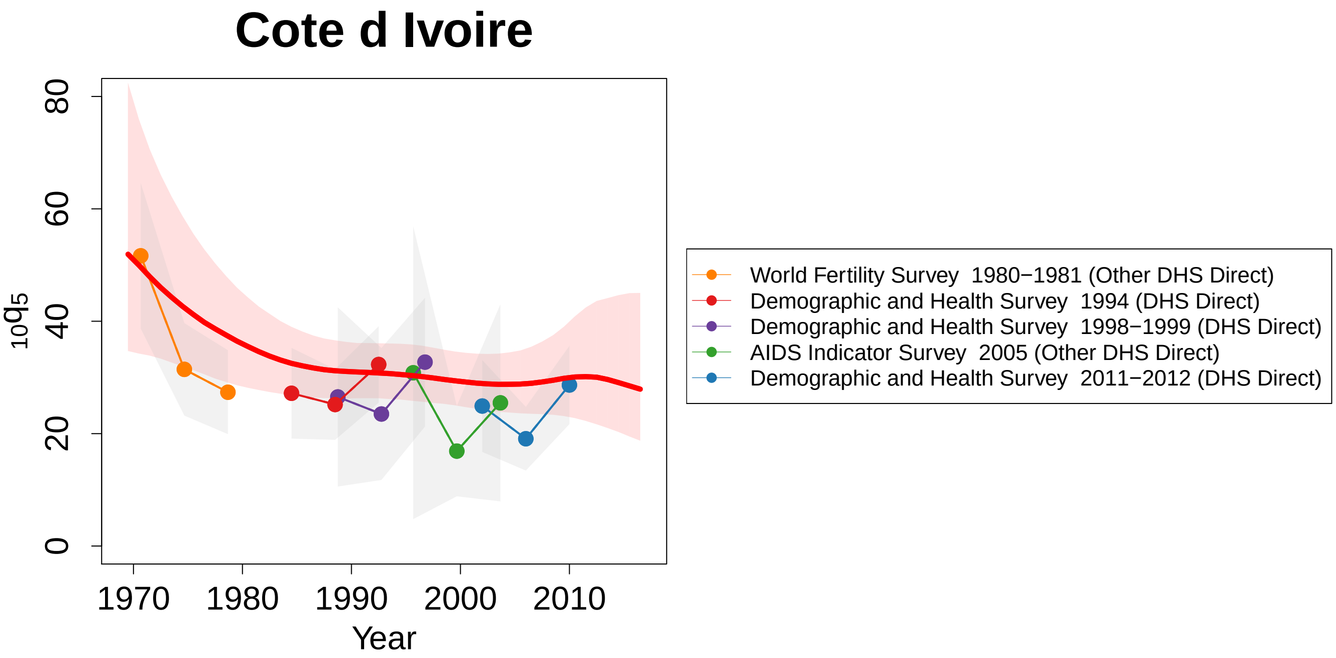
Cote d Ivoire
$_{10}q_5$
0
20
40
60
80
1970
1980
1990
2000
2010
Year
World Fertility Survey 1980−1981 (Other DHS Direct)
Demographic and Health Survey 1994 (DHS Direct)
Demographic and Health Survey 1998−1999 (DHS Direct)
AIDS Indicator Survey 2005 (Other DHS Direct)
Demographic and Health Survey 2011−2012 (DHS Direct)

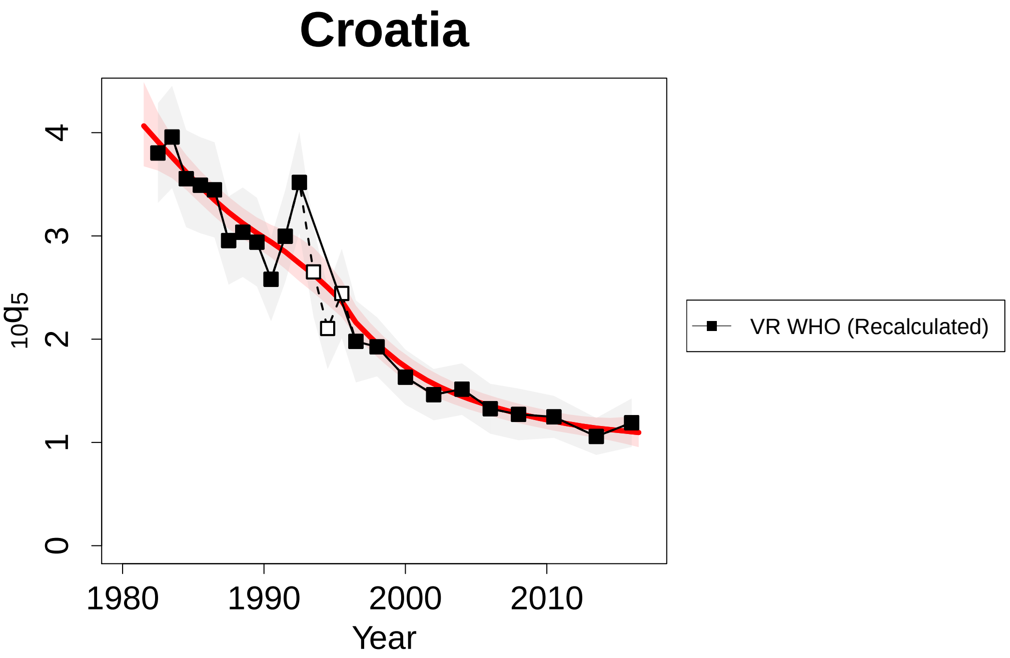
Croatia
4
3
2
1
0
$_{10}q_5$
1980
1990
2000
2010
Year
VR WHO (Recalculated)

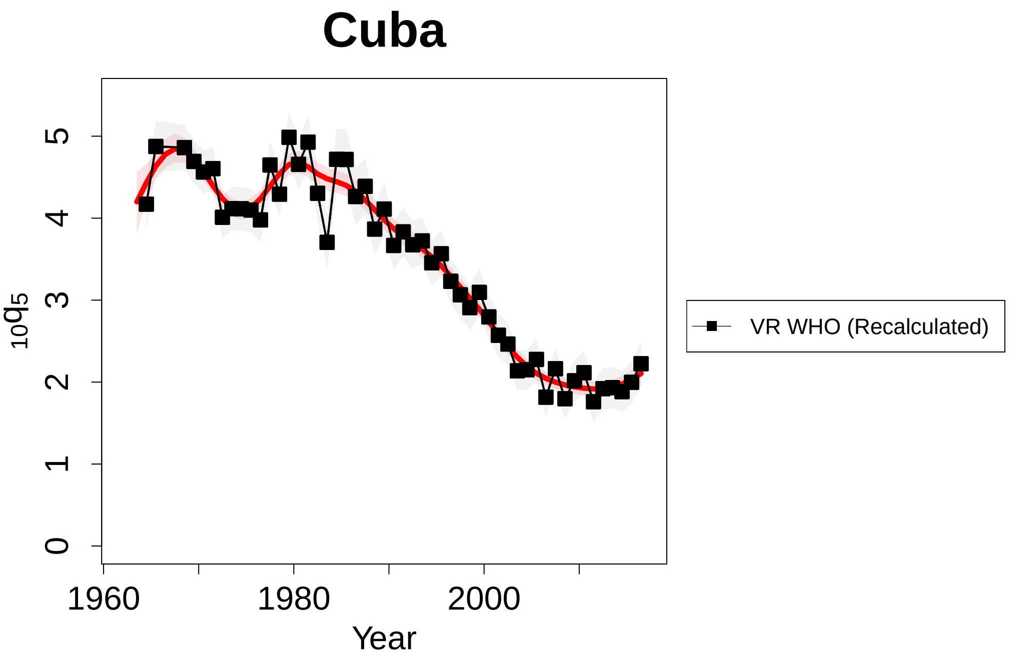
Cuba
5
4
3
2
1
0
$_{10}q_5$
1960
1980
2000
Year
VR WHO (Recalculated)

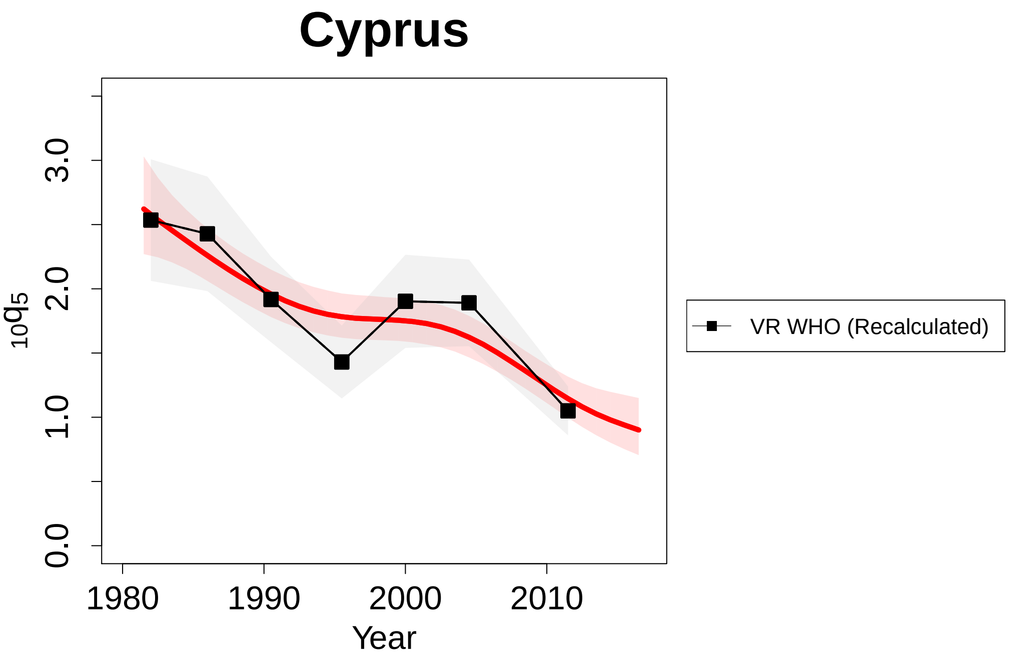
Cyprus
3.0
2.0
1.0
0.0
${}_{10}q_5$
1980
1990
2000
2010
Year
VR WHO (Recalculated)

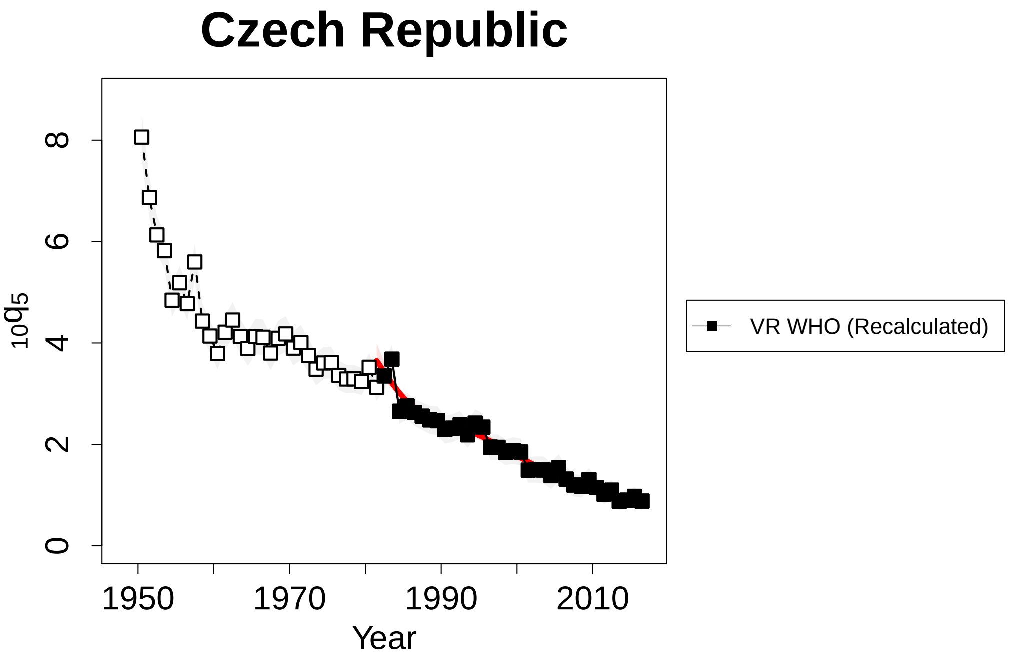
Czech Republic
8
6
4
2
0
${}_{10}q_5$
1950
1970
1990
2010
Year
VR WHO (Recalculated)

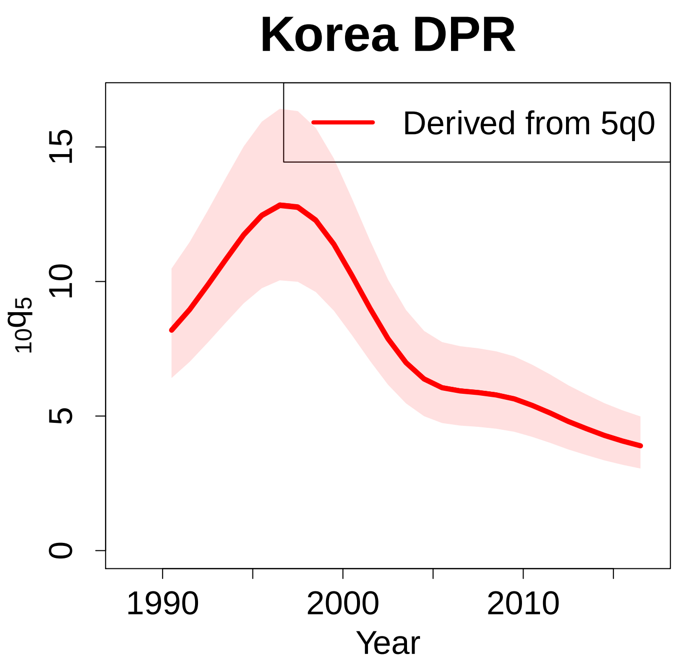
Korea DPR
Derived from 5q0
$_{10}q_5$
0
5
10
15
1990
2000
2010
Year

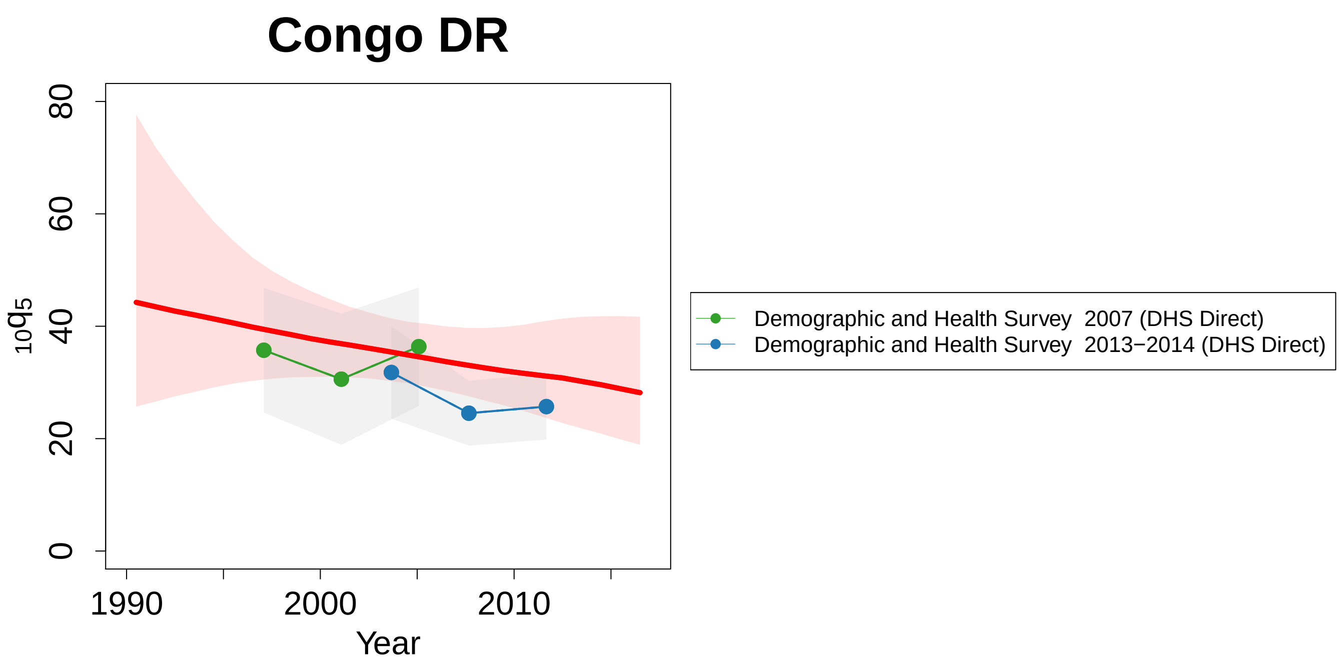
Congo DR
$_{10}q_5$
0
20
40
60
80
1990
2000
2010
Year
Demographic and Health Survey 2007 (DHS Direct)
Demographic and Health Survey 2013−2014 (DHS Direct)

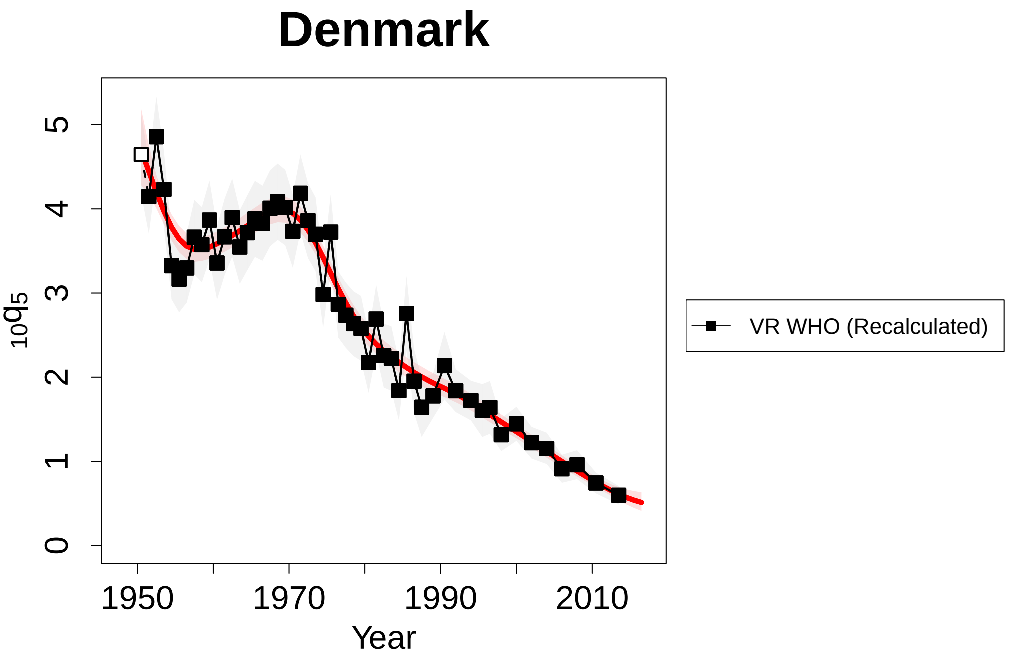
Denmark
5
4
3
2
1
0
10q5
1950
1970
1990
2010
Year
VR WHO (Recalculated)

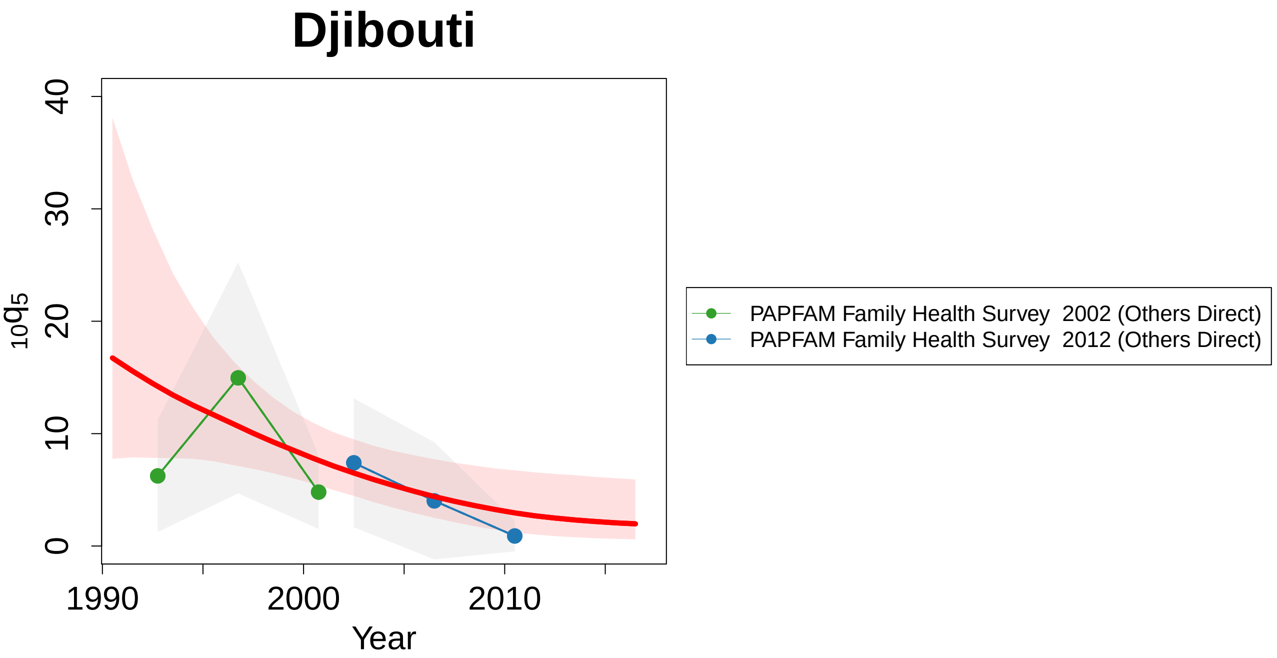
Djibouti
40
30
20
10
0
10q5
1990
2000
2010
Year
PAPFAM Family Health Survey 2002 (Others Direct)
PAPFAM Family Health Survey 2012 (Others Direct)

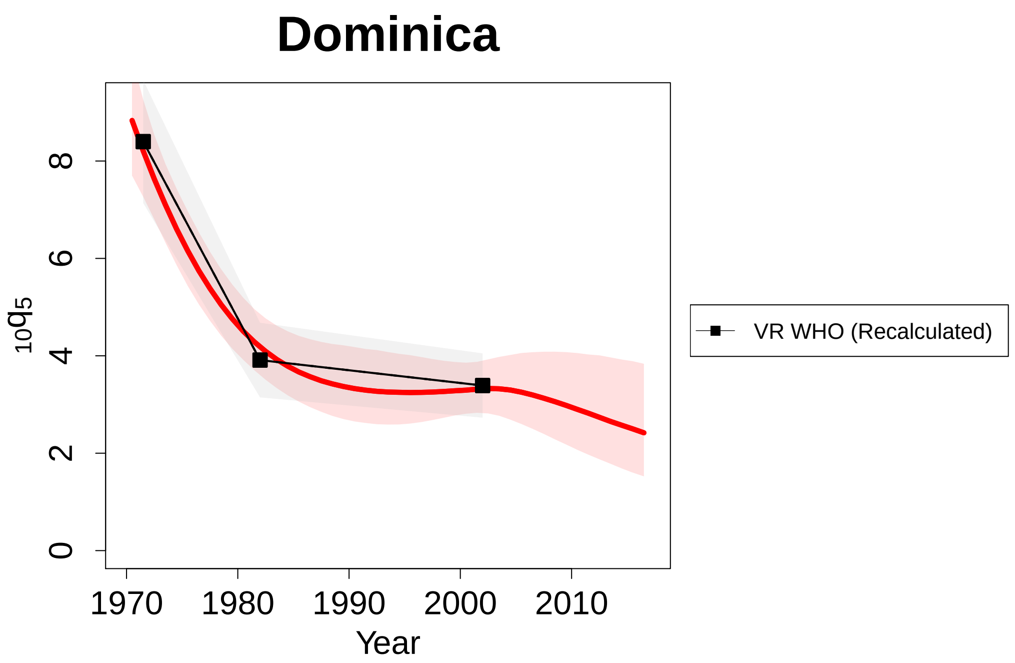
Dominica
8
6
4
2
0
${}_{10}q_5$
1970
1980
1990
2000
2010
Year
VR WHO (Recalculated)

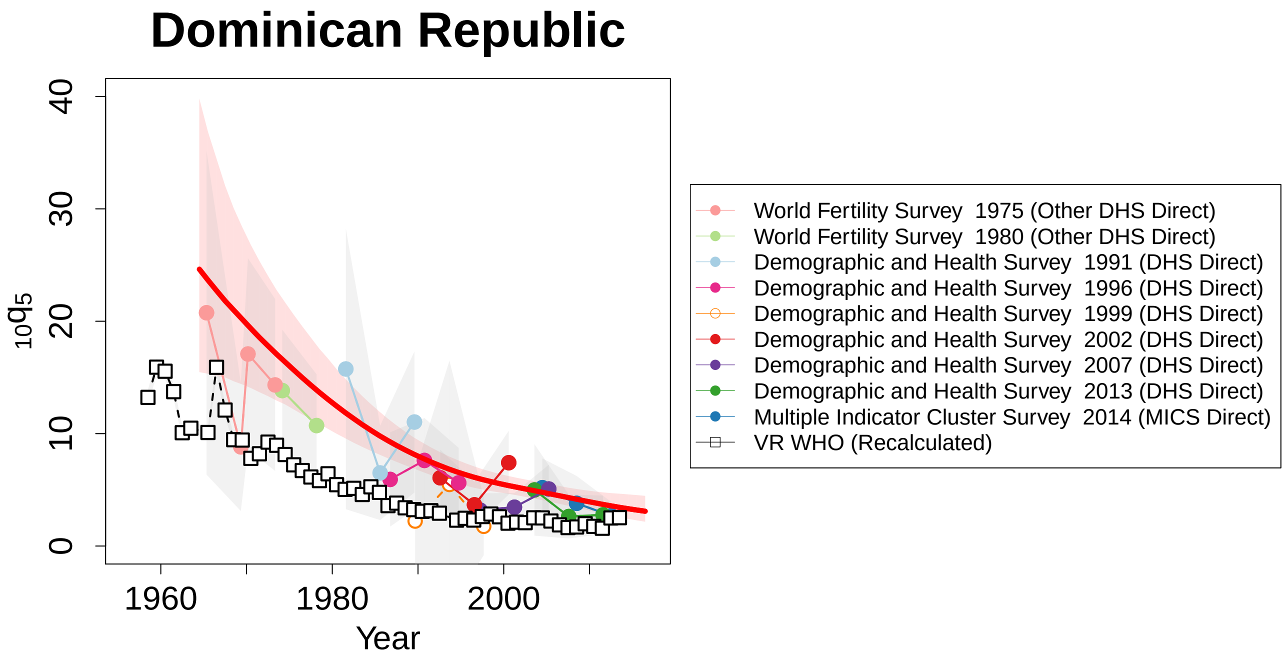
Dominican Republic
40
30
20
10
0
${}_{10}q_5$
1960
1980
2000
Year
World Fertility Survey 1975 (Other DHS Direct)
World Fertility Survey 1980 (Other DHS Direct)
Demographic and Health Survey 1991 (DHS Direct)
Demographic and Health Survey 1996 (DHS Direct)
Demographic and Health Survey 1999 (DHS Direct)
Demographic and Health Survey 2002 (DHS Direct)
Demographic and Health Survey 2007 (DHS Direct)
Demographic and Health Survey 2013 (DHS Direct)
Multiple Indicator Cluster Survey 2014 (MICS Direct)
VR WHO (Recalculated)

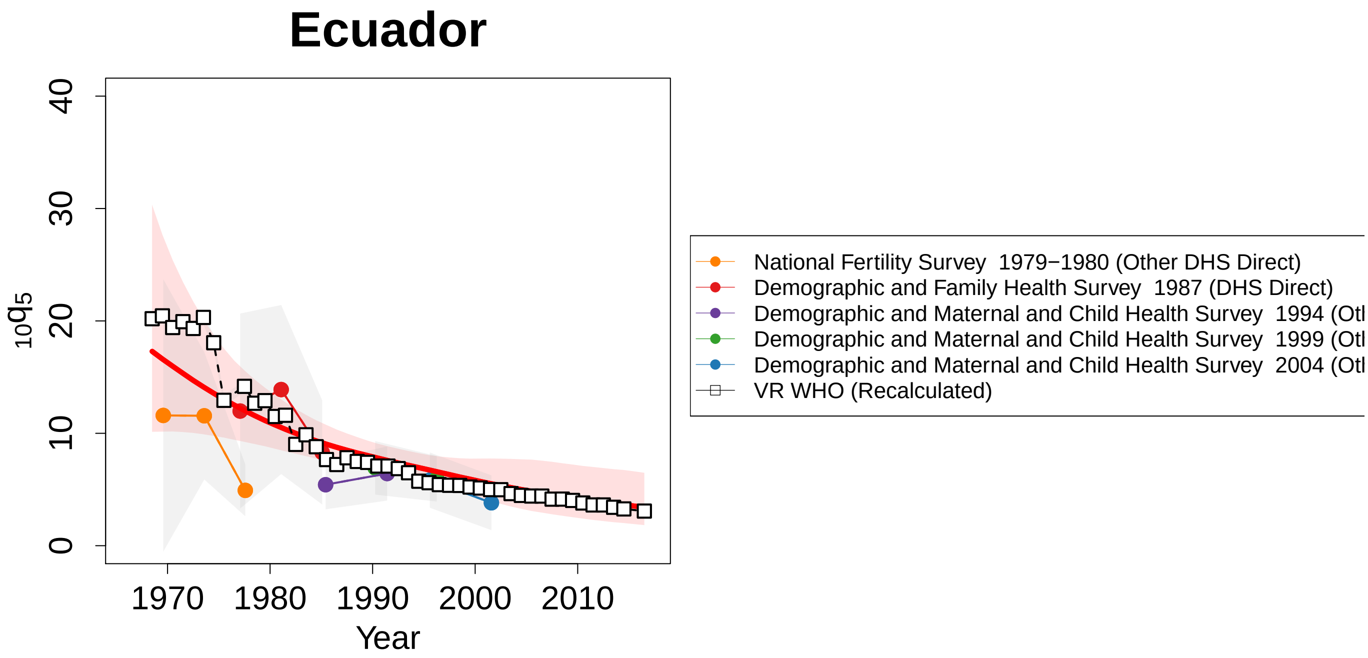
Ecuador
$_{10}q_5$
0
10
20
30
40
1970
1980
1990
2000
2010
Year
National Fertility Survey 1979−1980 (Other DHS Direct)
Demographic and Family Health Survey 1987 (DHS Direct)
Demographic and Maternal and Child Health Survey 1994 (Otl
Demographic and Maternal and Child Health Survey 1999 (Otl
Demographic and Maternal and Child Health Survey 2004 (Otl
VR WHO (Recalculated)

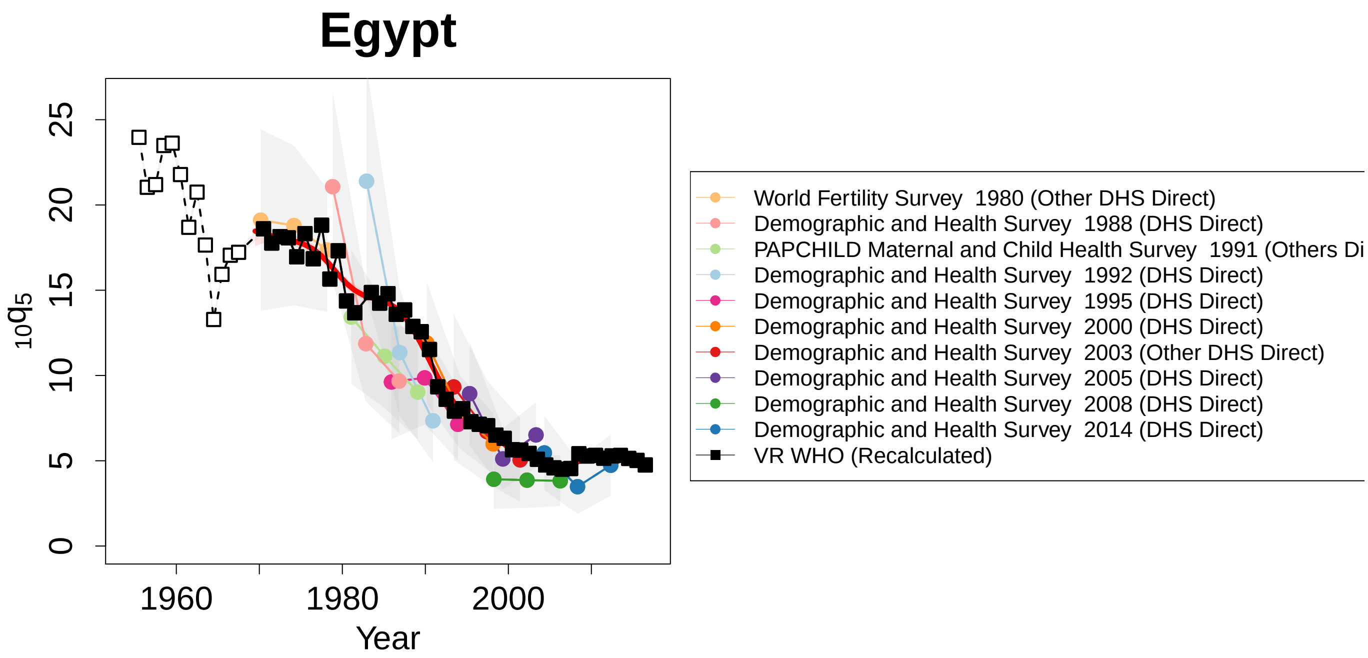
Egypt
$_{10}q_5$
0
5
10
15
20
25
1960
1980
2000
Year
World Fertility Survey 1980 (Other DHS Direct)
Demographic and Health Survey 1988 (DHS Direct)
PAPCHILD Maternal and Child Health Survey 1991 (Others Di
Demographic and Health Survey 1992 (DHS Direct)
Demographic and Health Survey 1995 (DHS Direct)
Demographic and Health Survey 2000 (DHS Direct)
Demographic and Health Survey 2003 (Other DHS Direct)
Demographic and Health Survey 2005 (DHS Direct)
Demographic and Health Survey 2008 (DHS Direct)
Demographic and Health Survey 2014 (DHS Direct)
VR WHO (Recalculated)

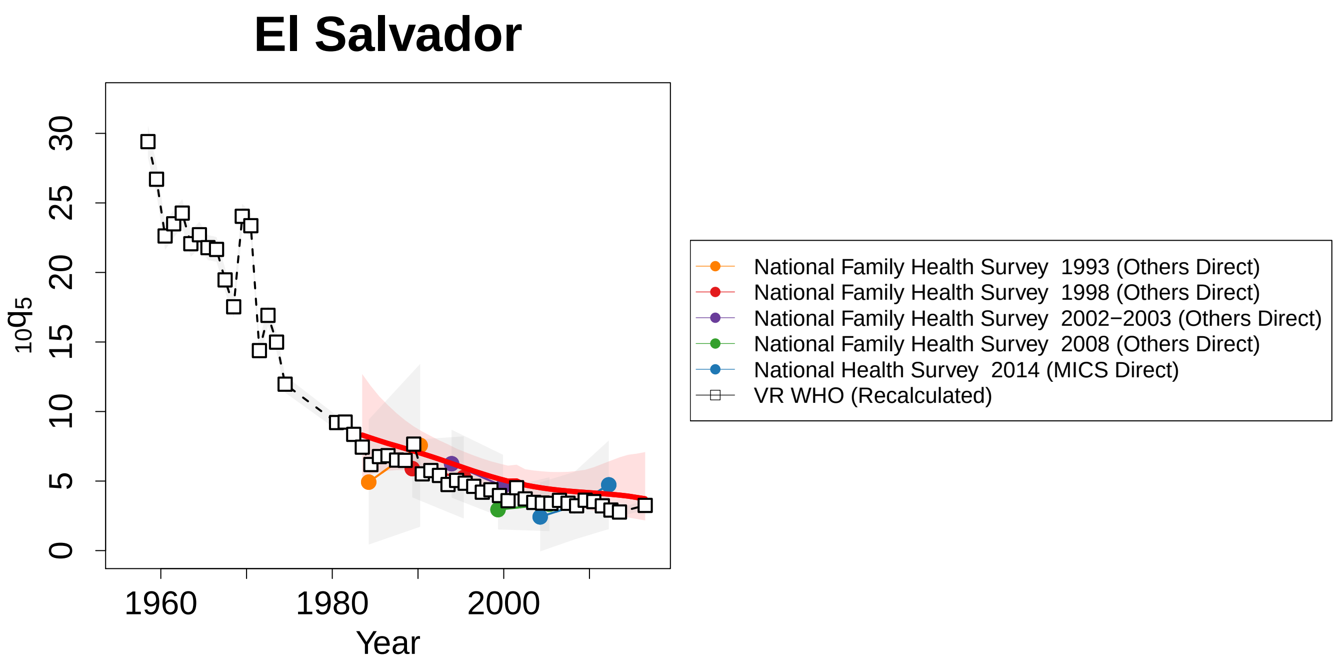
El Salvador
$_{10}q_5$
0
5
10
15
20
25
30
1960
1980
2000
Year
National Family Health Survey 1993 (Others Direct)
National Family Health Survey 1998 (Others Direct)
National Family Health Survey 2002−2003 (Others Direct)
National Family Health Survey 2008 (Others Direct)
National Health Survey 2014 (MICS Direct)
VR WHO (Recalculated)

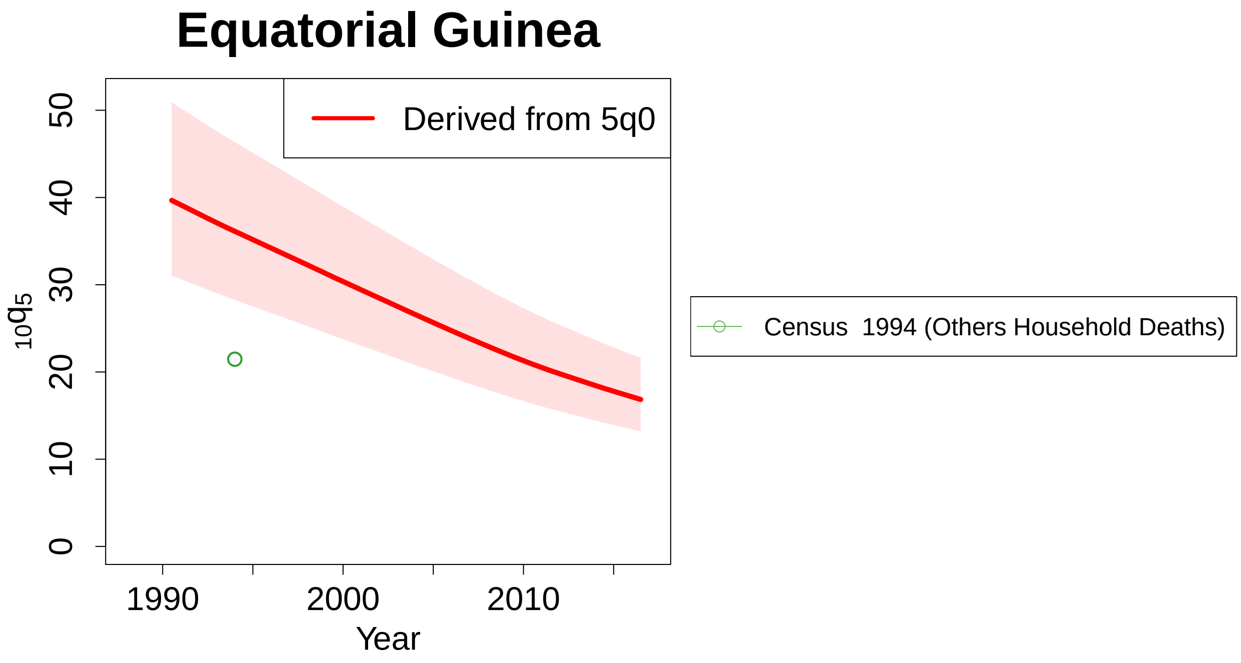
Equatorial Guinea
$_{10}q_5$
0
10
20
30
40
50
1990
2000
2010
Year
Derived from 5q0
Census 1994 (Others Household Deaths)

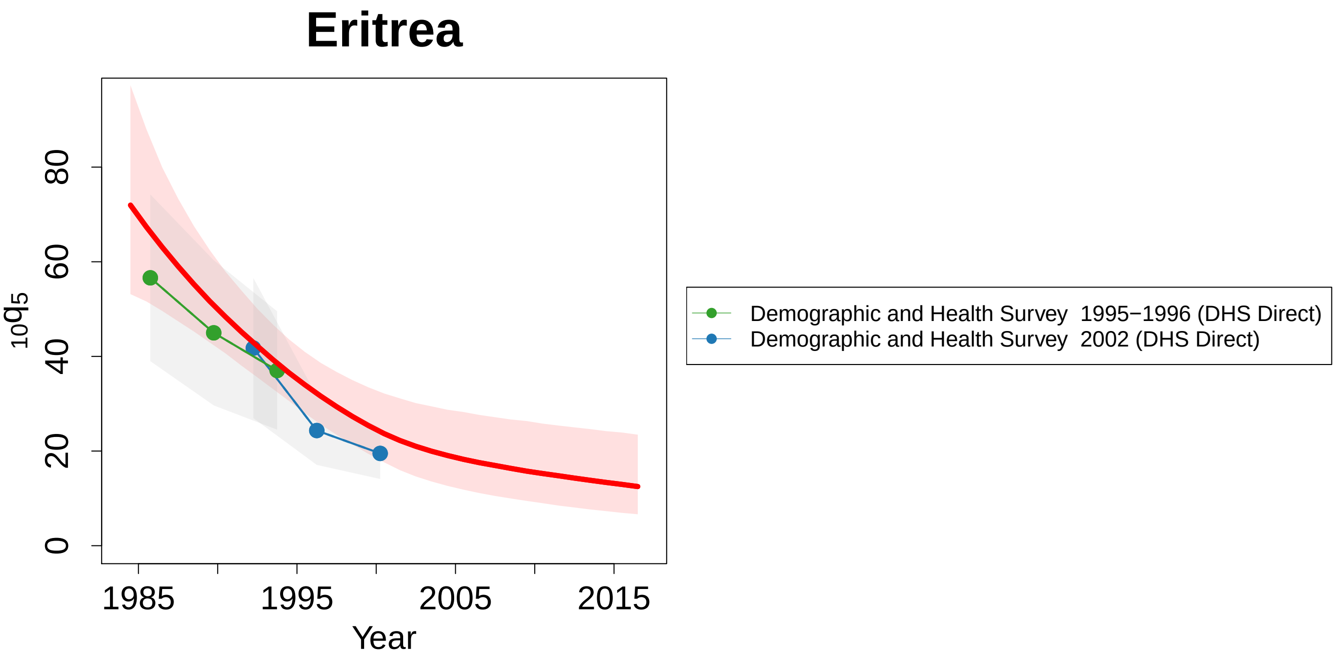
Eritrea
$_{10}q_5$
0
20
40
60
80
1985
1995
2005
2015
Year
Demographic and Health Survey 1995−1996 (DHS Direct)
Demographic and Health Survey 2002 (DHS Direct)

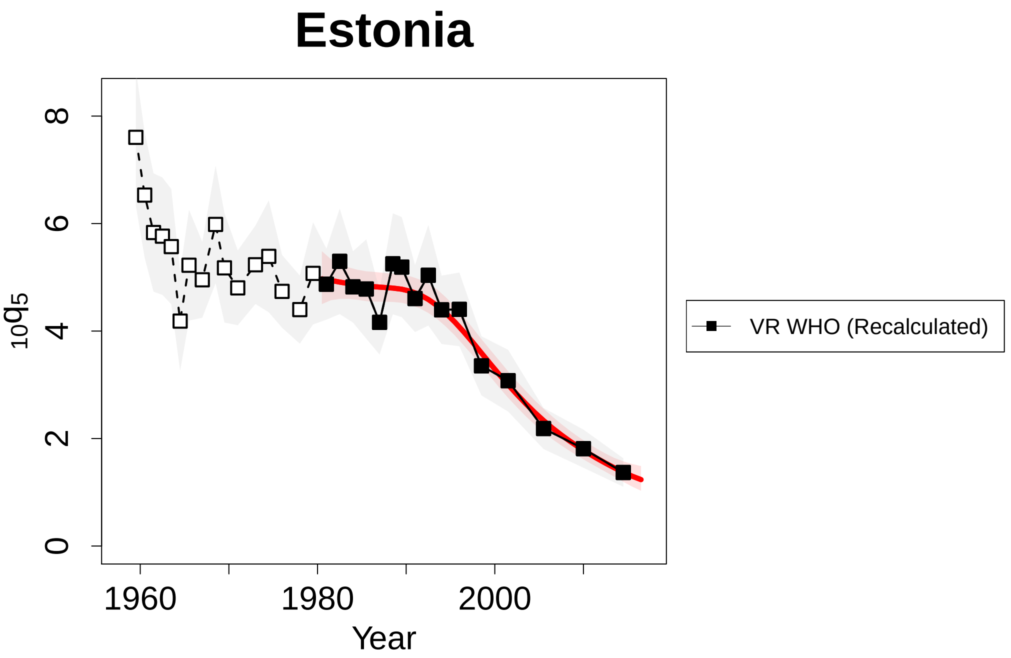
Estonia
$_{10}q_5$
0
2
4
6
8
1960
1980
2000
Year
VR WHO (Recalculated)

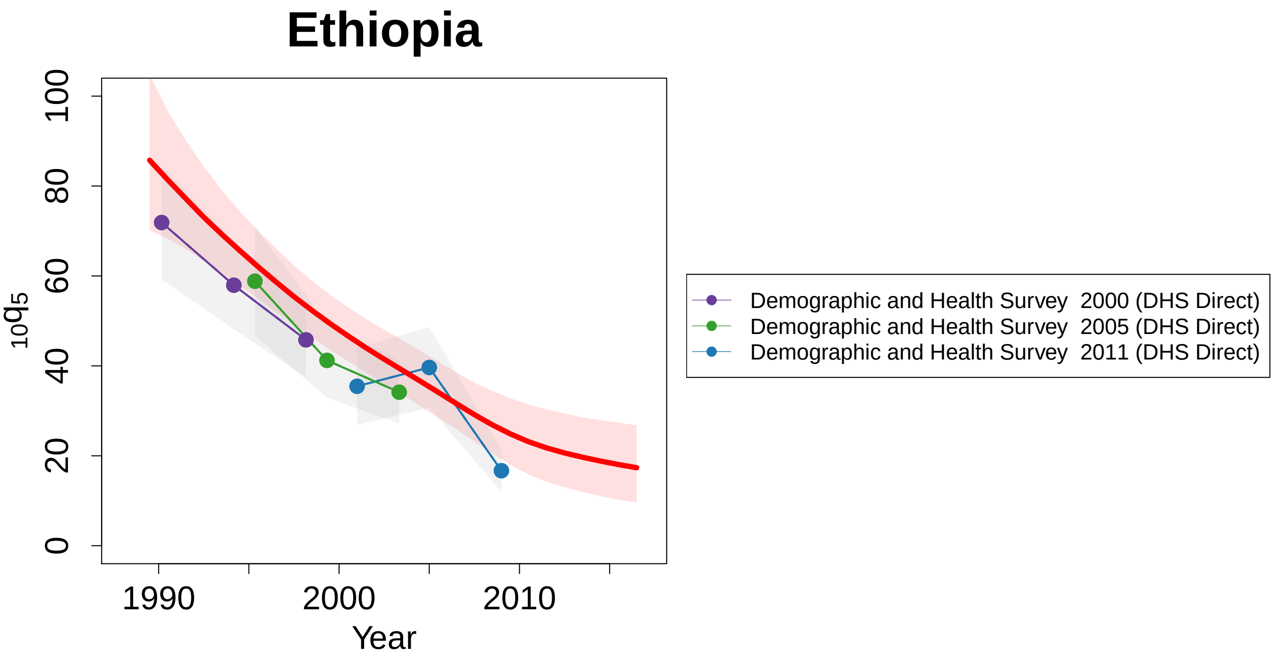
Ethiopia
$_{10}q_5$
0
20
40
60
80
100
1990
2000
2010
Year
Demographic and Health Survey 2000 (DHS Direct)
Demographic and Health Survey 2005 (DHS Direct)
Demographic and Health Survey 2011 (DHS Direct)

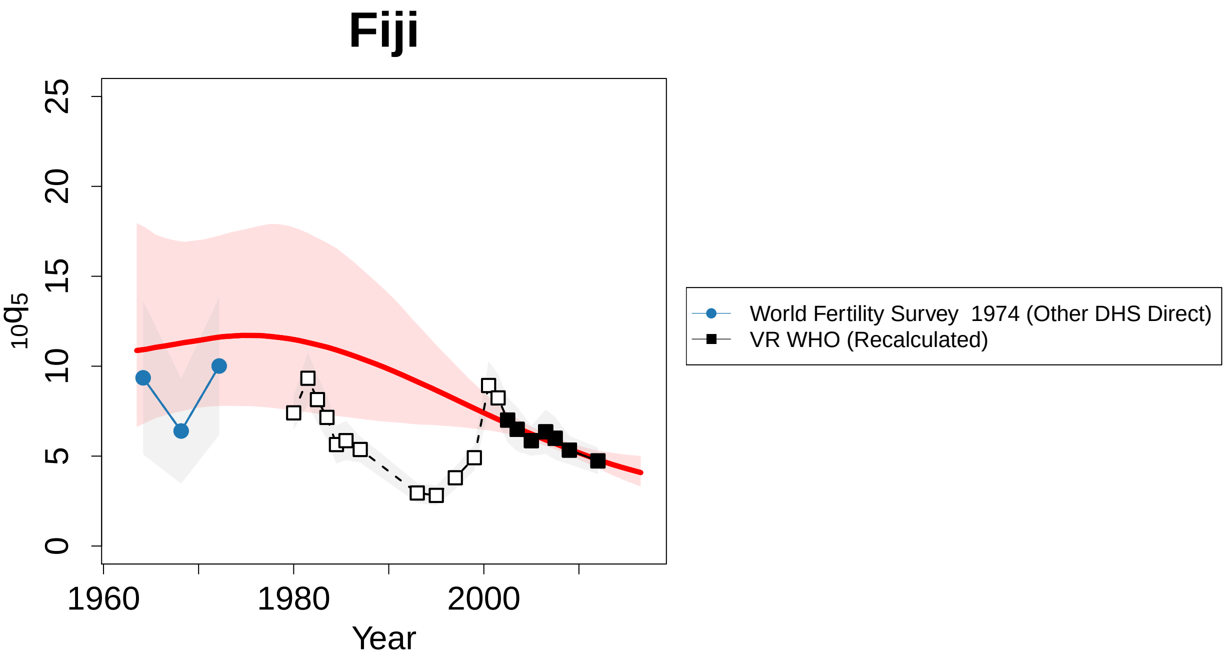
Fiji
$_{10}q_5$
0
5
10
15
20
25
1960
1980
2000
Year
World Fertility Survey 1974 (Other DHS Direct)
VR WHO (Recalculated)

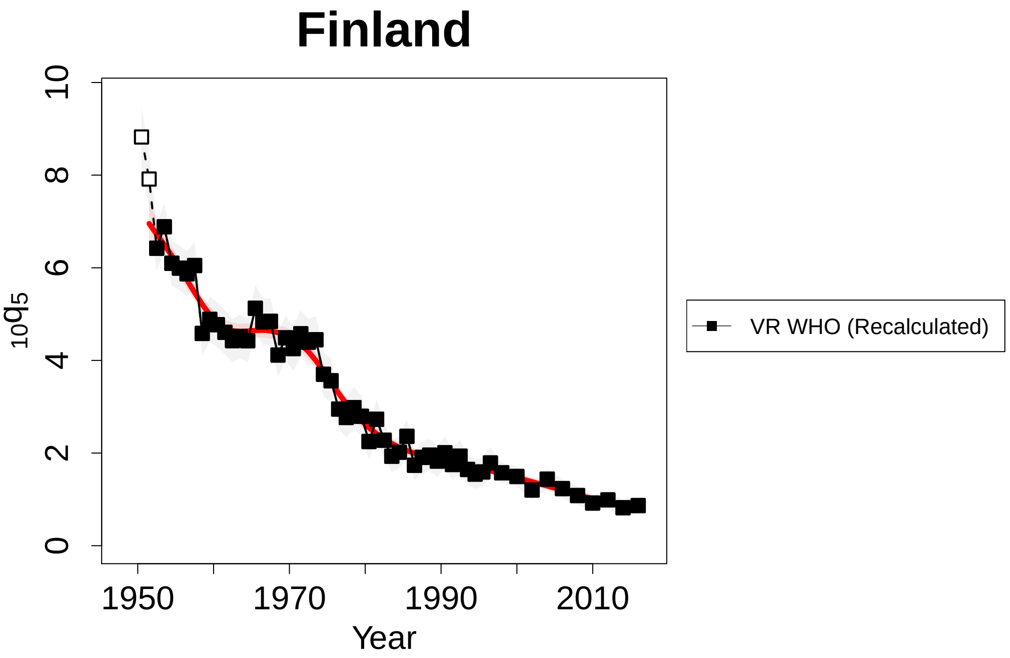
Finland
$_{10}q_5$
0
2
4
6
8
10
1950
1970
1990
2010
Year
VR WHO (Recalculated)

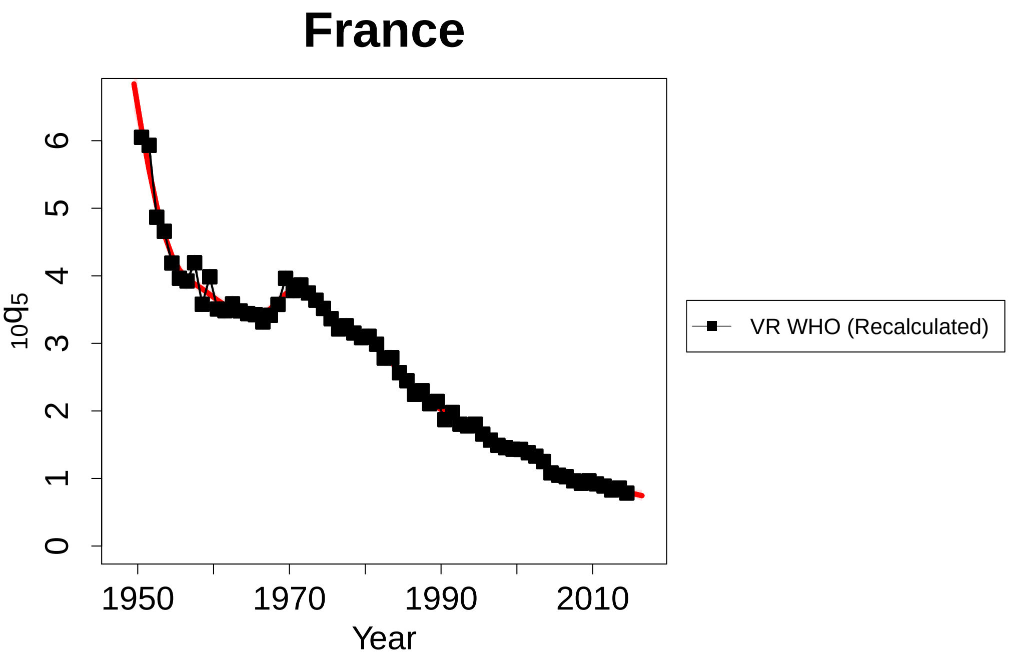
France
$_{10}q_5$
0
1
2
3
4
5
6
1950
1970
1990
2010
Year
VR WHO (Recalculated)

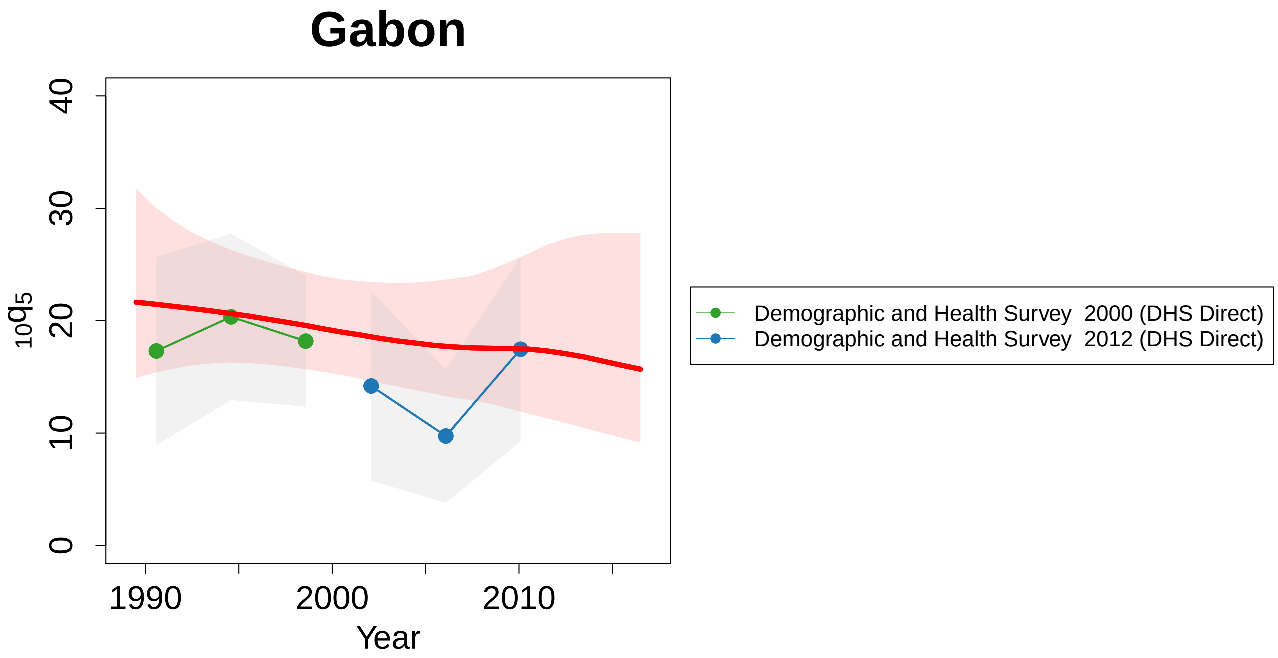
Gabon
40
30
20
10
0
$_{10}q_5$
1990
2000
2010
Year
Demographic and Health Survey 2000 (DHS Direct)
Demographic and Health Survey 2012 (DHS Direct)

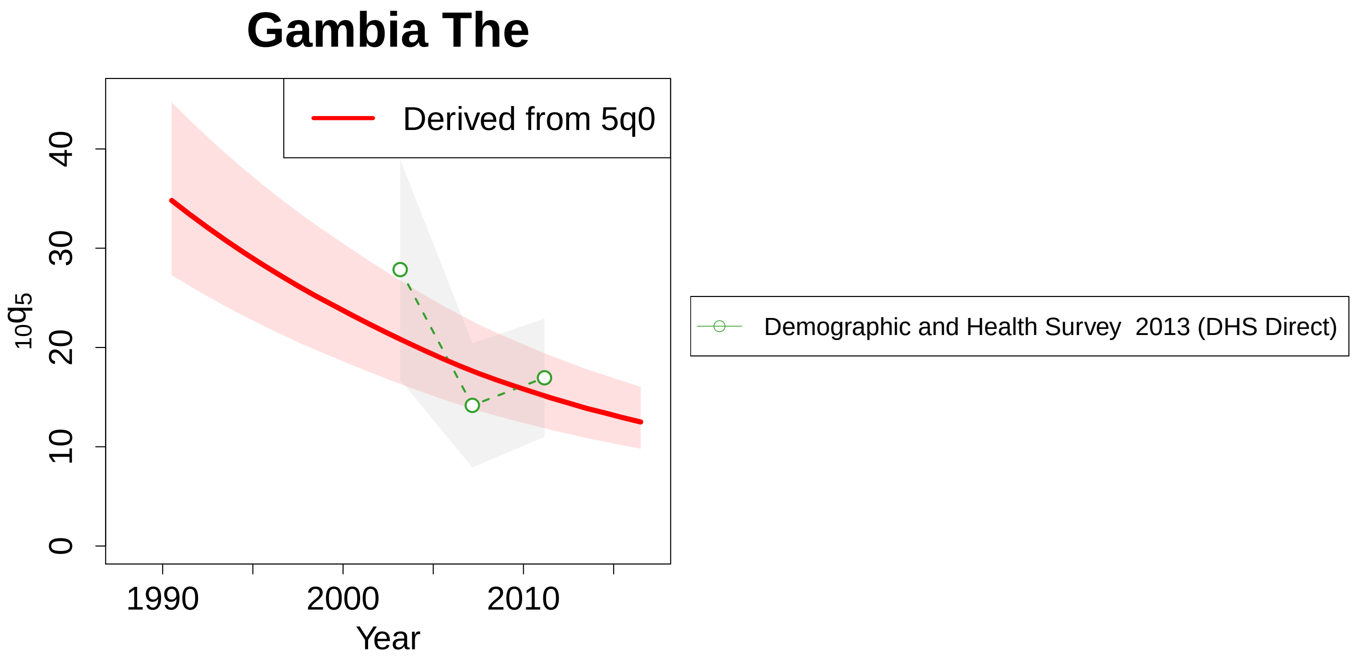
Gambia The
Derived from 5q0
40
30
20
10
0
$_{10}q_5$
1990
2000
2010
Year
Demographic and Health Survey 2013 (DHS Direct)

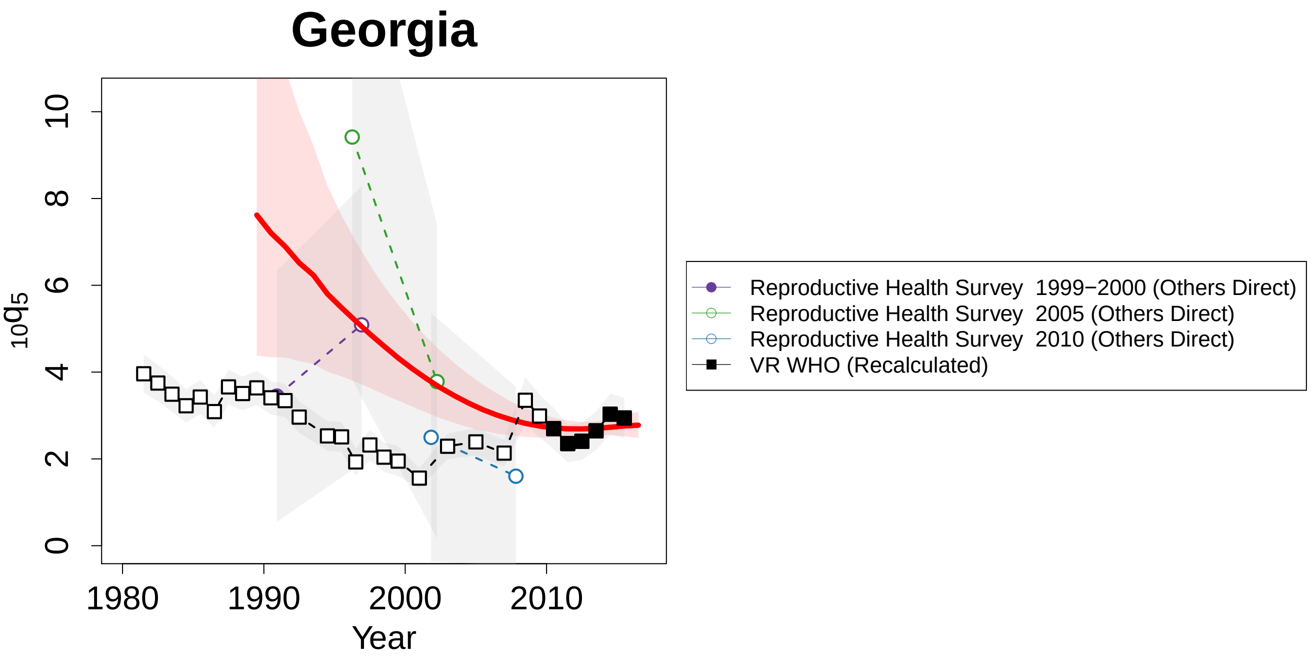
Georgia
10q5
0
2
4
6
8
10
1980
1990
2000
2010
Year
Reproductive Health Survey 1999−2000 (Others Direct)
Reproductive Health Survey 2005 (Others Direct)
Reproductive Health Survey 2010 (Others Direct)
VR WHO (Recalculated)

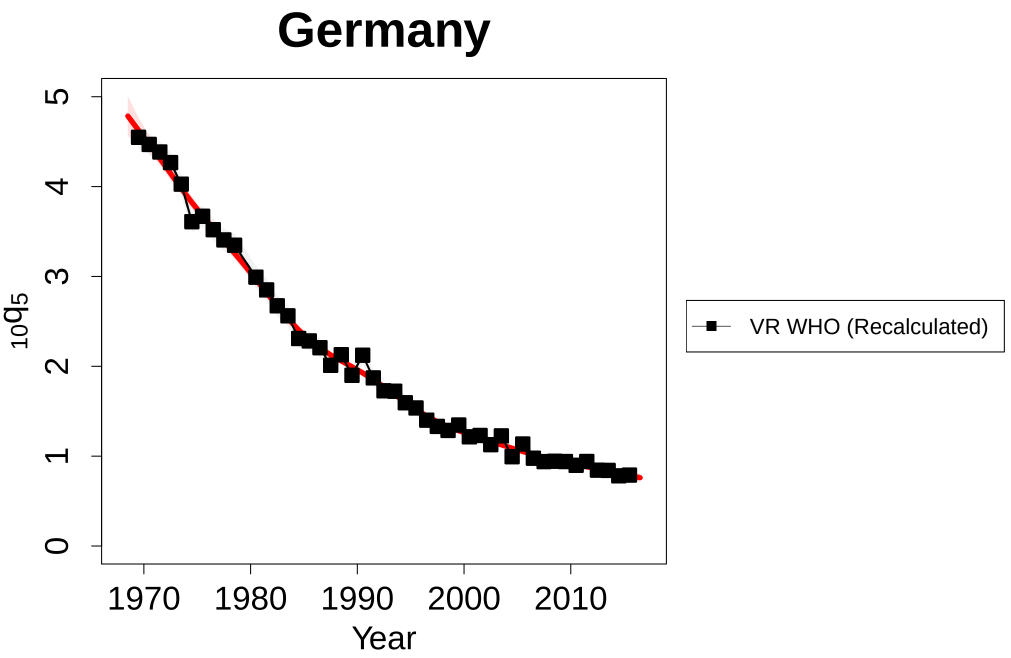
Germany
10q5
0
1
2
3
4
5
1970
1980
1990
2000
2010
Year
VR WHO (Recalculated)

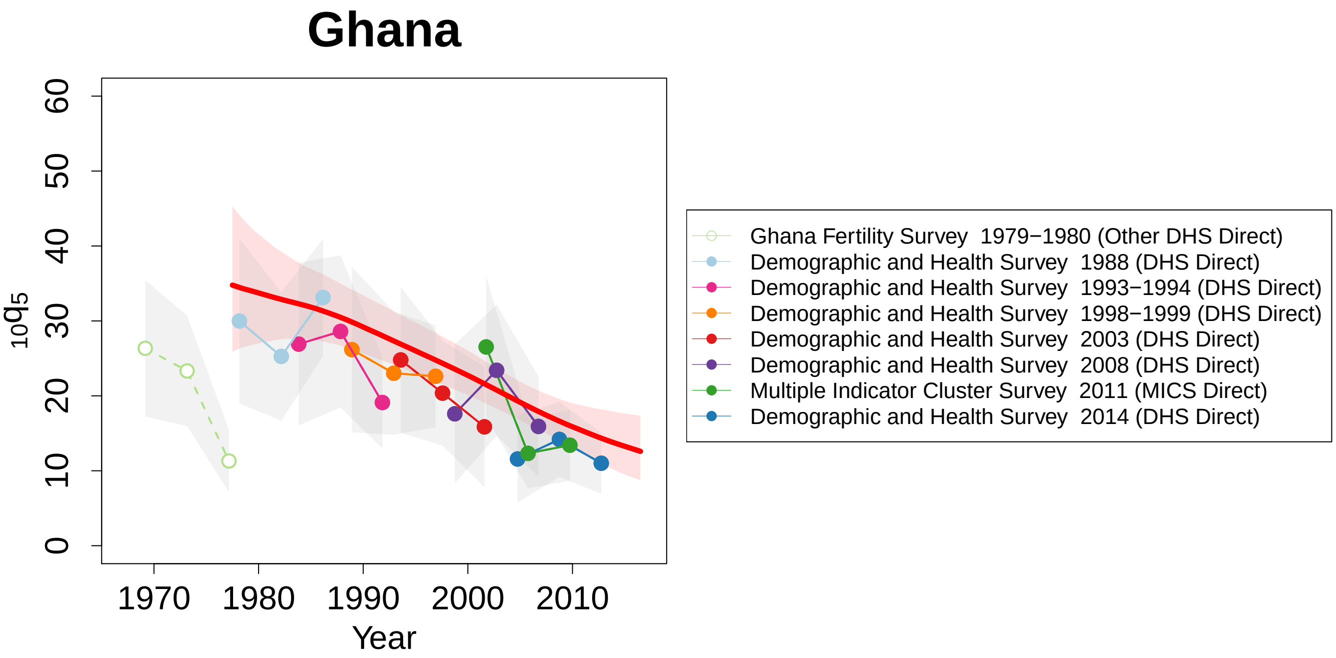
Ghana
$_{10}q_5$
Year
Ghana Fertility Survey 1979−1980 (Other DHS Direct)
Demographic and Health Survey 1988 (DHS Direct)
Demographic and Health Survey 1993−1994 (DHS Direct)
Demographic and Health Survey 1998−1999 (DHS Direct)
Demographic and Health Survey 2003 (DHS Direct)
Demographic and Health Survey 2008 (DHS Direct)
Multiple Indicator Cluster Survey 2011 (MICS Direct)
Demographic and Health Survey 2014 (DHS Direct)

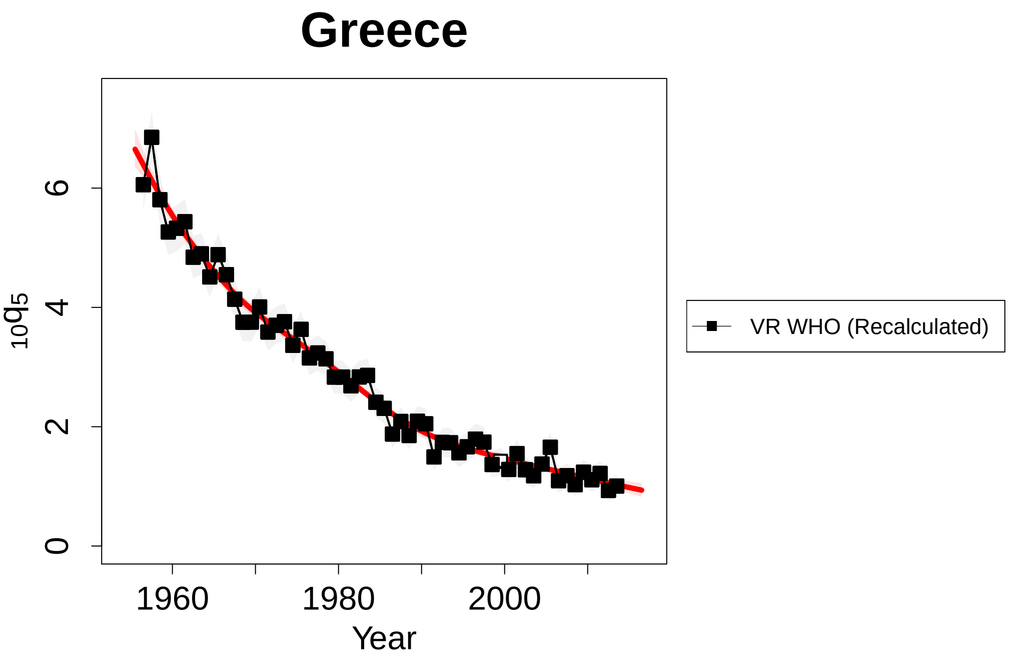
Greece
$_{10}q_5$
Year
VR WHO (Recalculated)

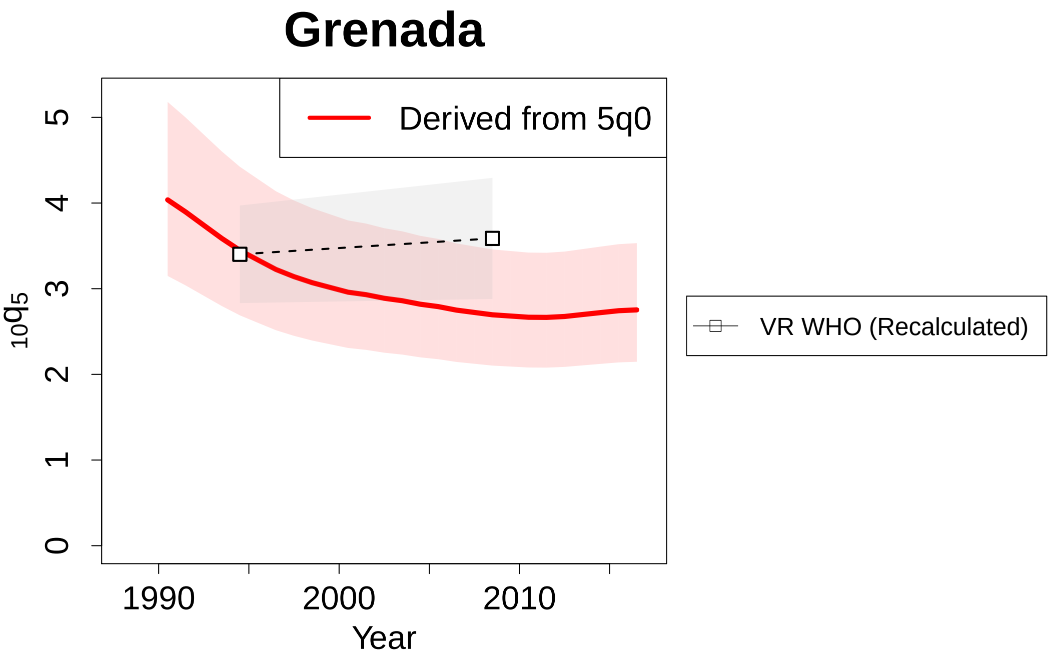
Grenada
Derived from 5q0
VR WHO (Recalculated)
$_{10}q_5$
0
1
2
3
4
5
1990
2000
2010
Year

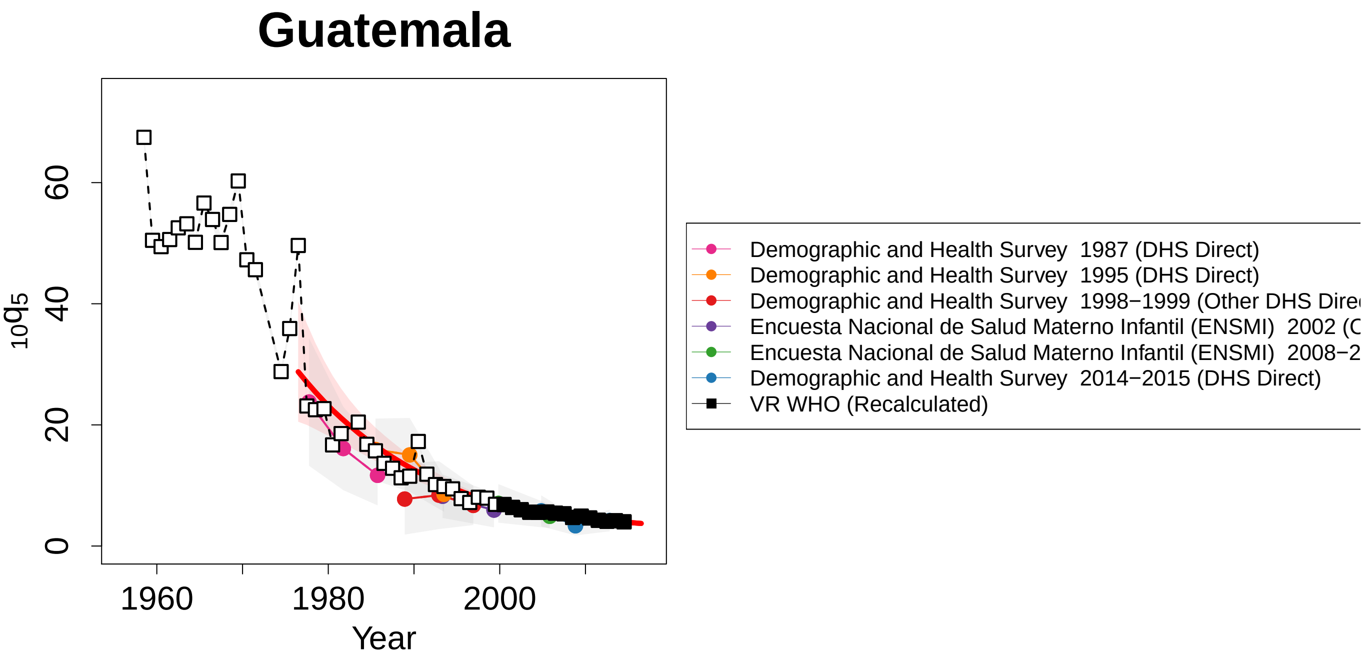
Guatemala
Demographic and Health Survey 1987 (DHS Direct)
Demographic and Health Survey 1995 (DHS Direct)
Demographic and Health Survey 1998−1999 (Other DHS Dire
Encuesta Nacional de Salud Materno Infantil (ENSMI) 2002 (C
Encuesta Nacional de Salud Materno Infantil (ENSMI) 2008−2
Demographic and Health Survey 2014−2015 (DHS Direct)
VR WHO (Recalculated)
$_{10}q_5$
0
20
40
60
1960
1980
2000
Year

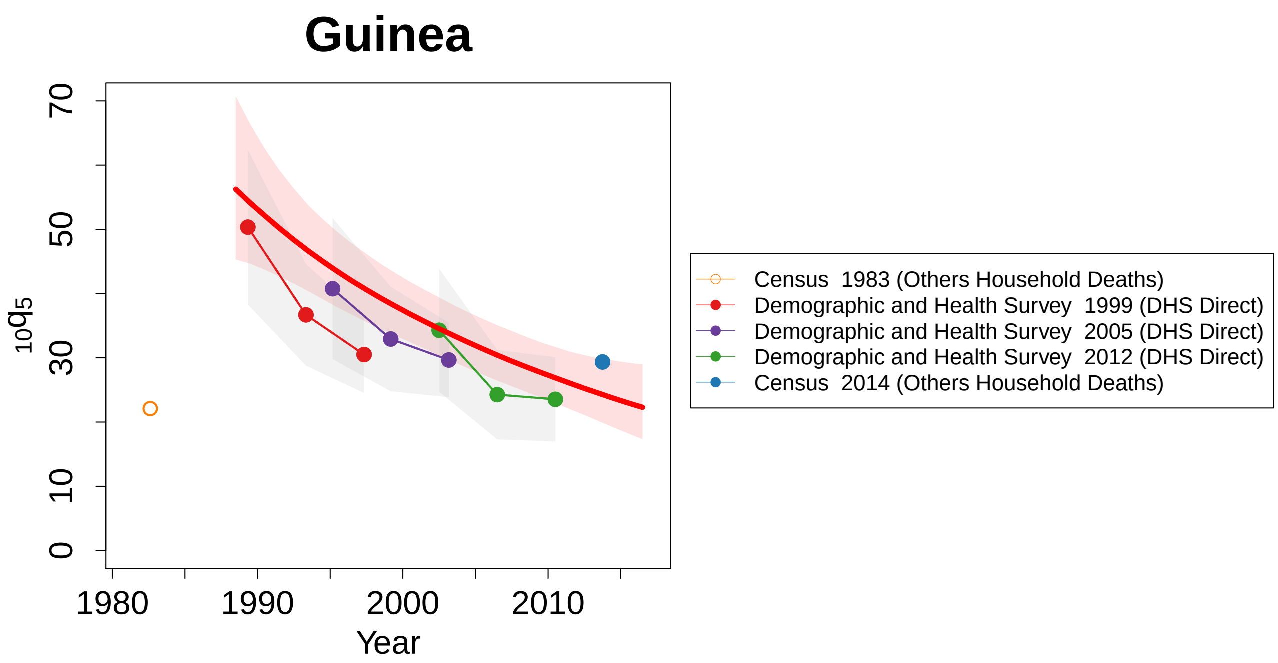
Guinea
70
50
30
10
0
$_{10}q_5$
1980
1990
2000
2010
Year
Census 1983 (Others Household Deaths)
Demographic and Health Survey 1999 (DHS Direct)
Demographic and Health Survey 2005 (DHS Direct)
Demographic and Health Survey 2012 (DHS Direct)
Census 2014 (Others Household Deaths)

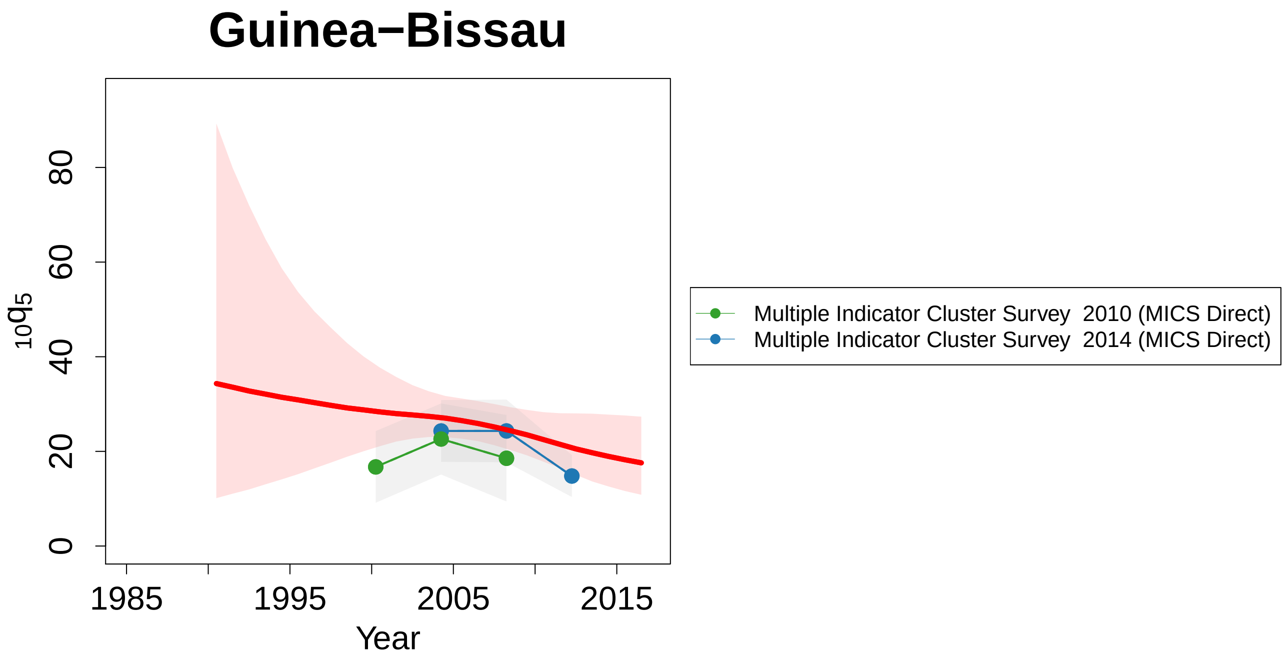
Guinea−Bissau
80
60
40
20
0
$_{10}q_5$
1985
1995
2005
2015
Year
Multiple Indicator Cluster Survey 2010 (MICS Direct)
Multiple Indicator Cluster Survey 2014 (MICS Direct)

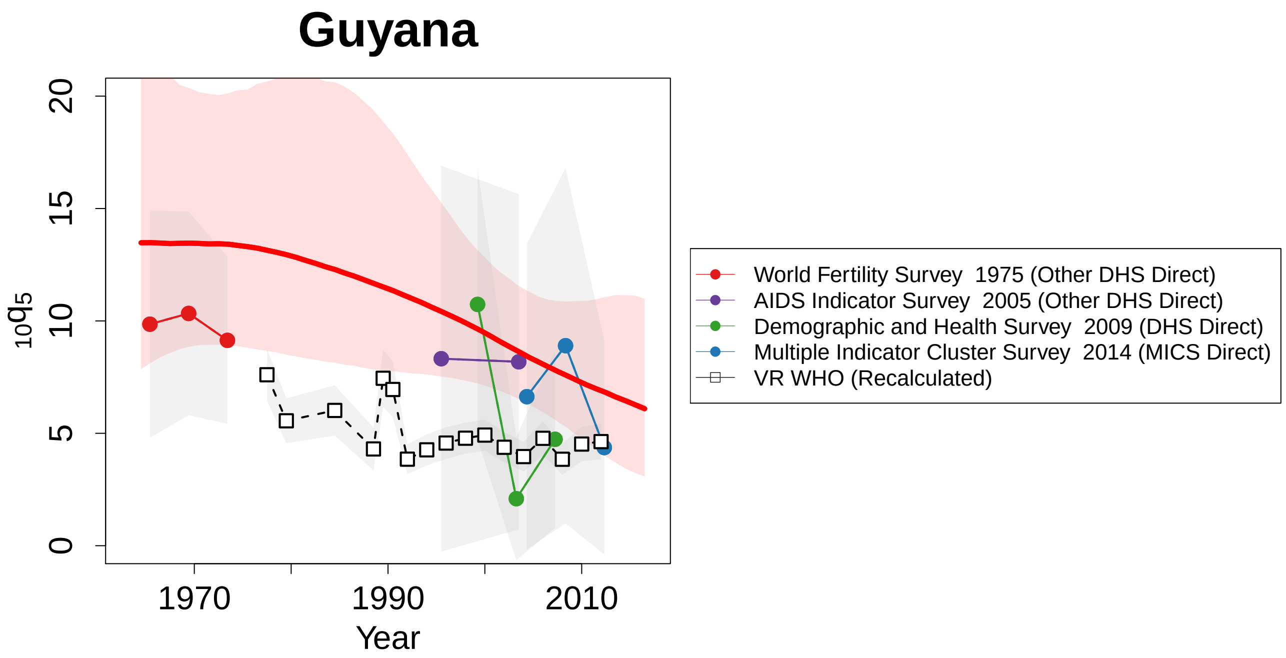
Guyana
$_{10}q_5$
0
5
10
15
20
1970
1990
2010
Year
World Fertility Survey 1975 (Other DHS Direct)
AIDS Indicator Survey 2005 (Other DHS Direct)
Demographic and Health Survey 2009 (DHS Direct)
Multiple Indicator Cluster Survey 2014 (MICS Direct)
VR WHO (Recalculated)

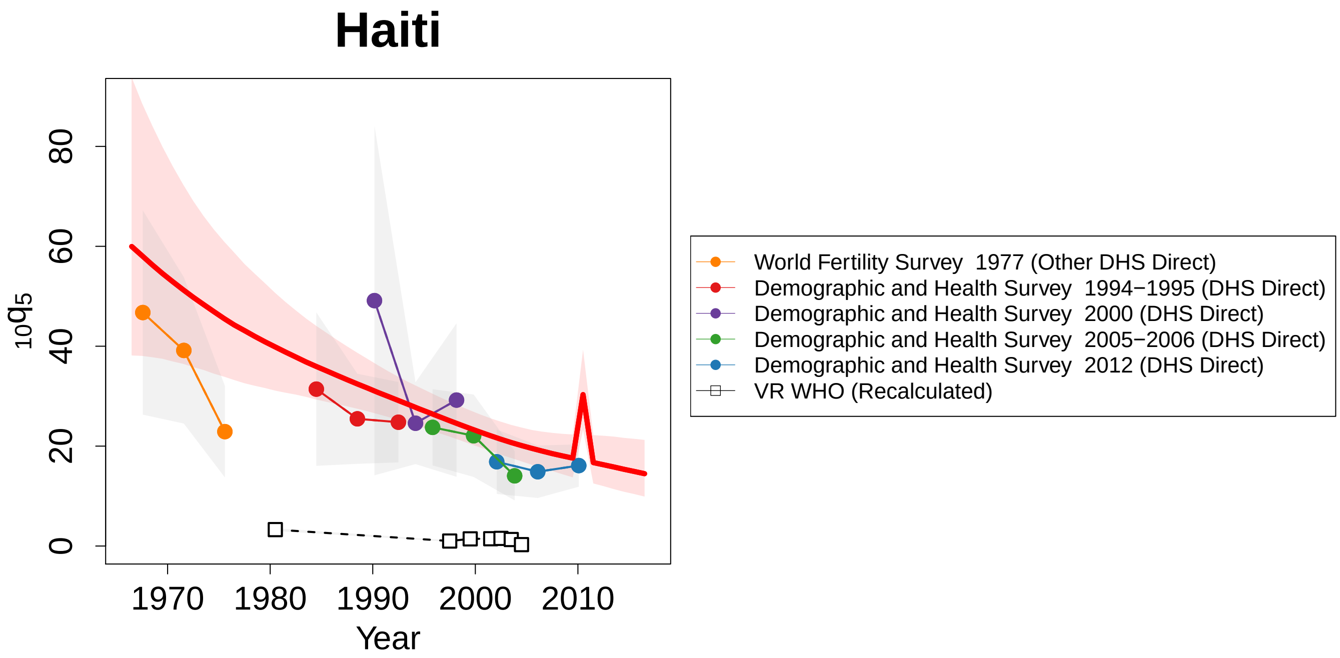
Haiti
$_{10}q_5$
0
20
40
60
80
1970
1980
1990
2000
2010
Year
World Fertility Survey 1977 (Other DHS Direct)
Demographic and Health Survey 1994−1995 (DHS Direct)
Demographic and Health Survey 2000 (DHS Direct)
Demographic and Health Survey 2005−2006 (DHS Direct)
Demographic and Health Survey 2012 (DHS Direct)
VR WHO (Recalculated)

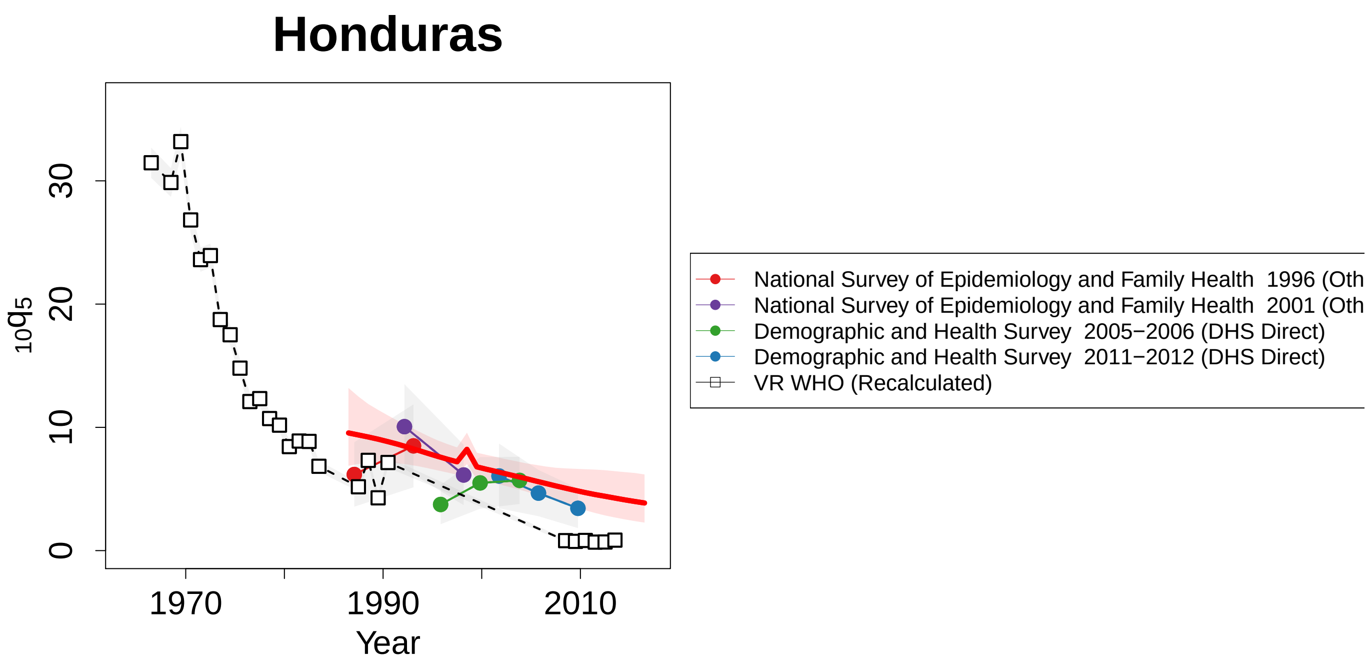
Honduras
30
20
10
0
$_{10}q_5$
1970
1990
2010
Year
National Survey of Epidemiology and Family Health 1996 (Oth
National Survey of Epidemiology and Family Health 2001 (Oth
Demographic and Health Survey 2005−2006 (DHS Direct)
Demographic and Health Survey 2011−2012 (DHS Direct)
VR WHO (Recalculated)

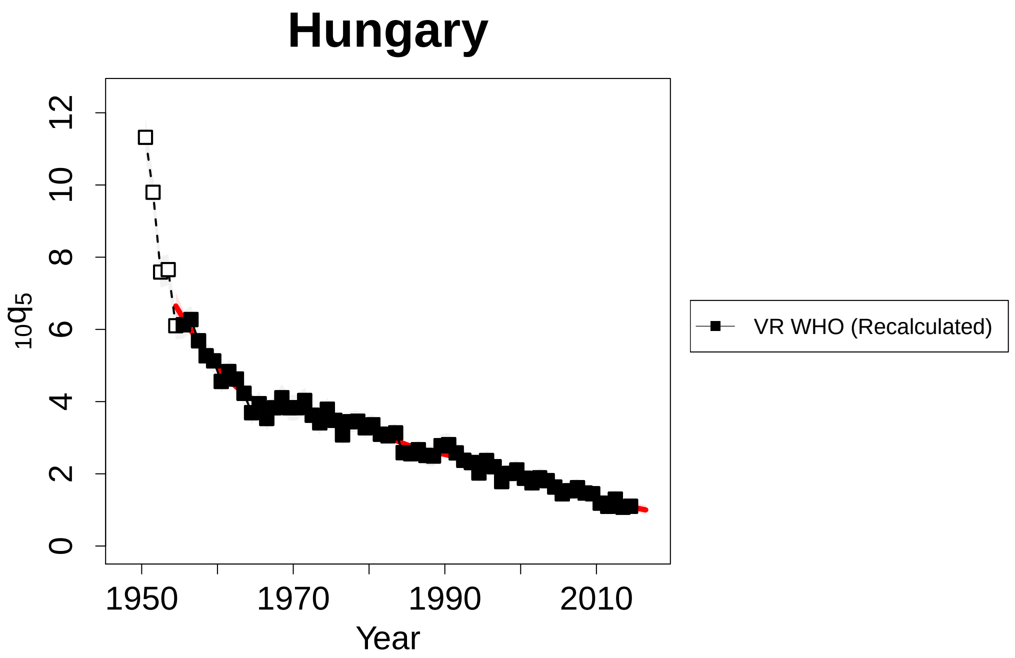
Hungary
12
10
8
6
4
2
0
$_{10}q_5$
1950
1970
1990
2010
Year
VR WHO (Recalculated)

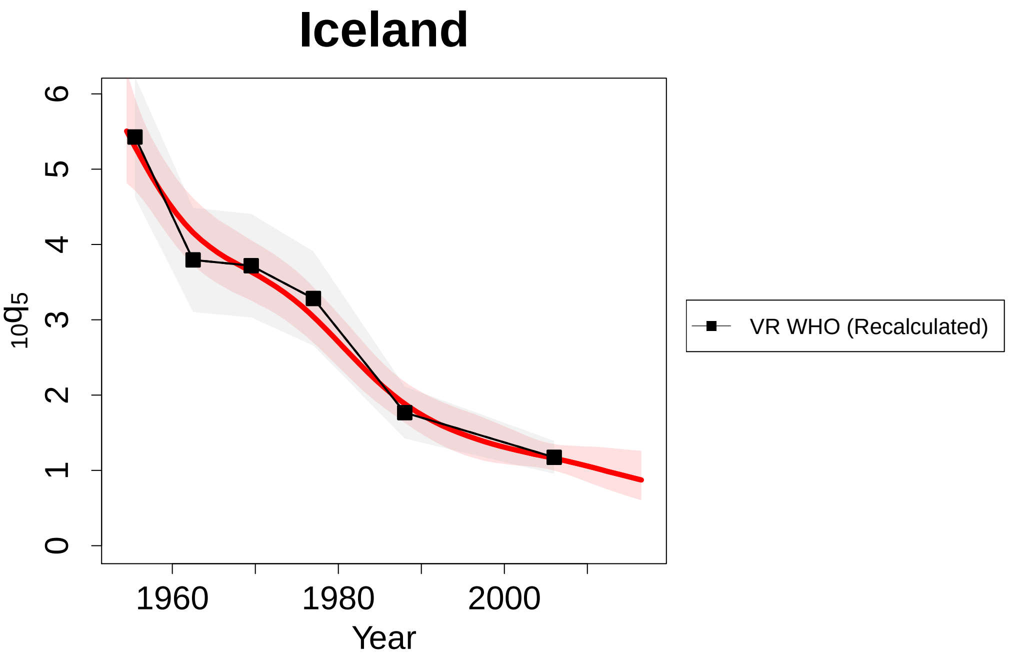
Iceland
6
5
4
3
2
1
0
10q5
1960
1980
2000
Year
VR WHO (Recalculated)

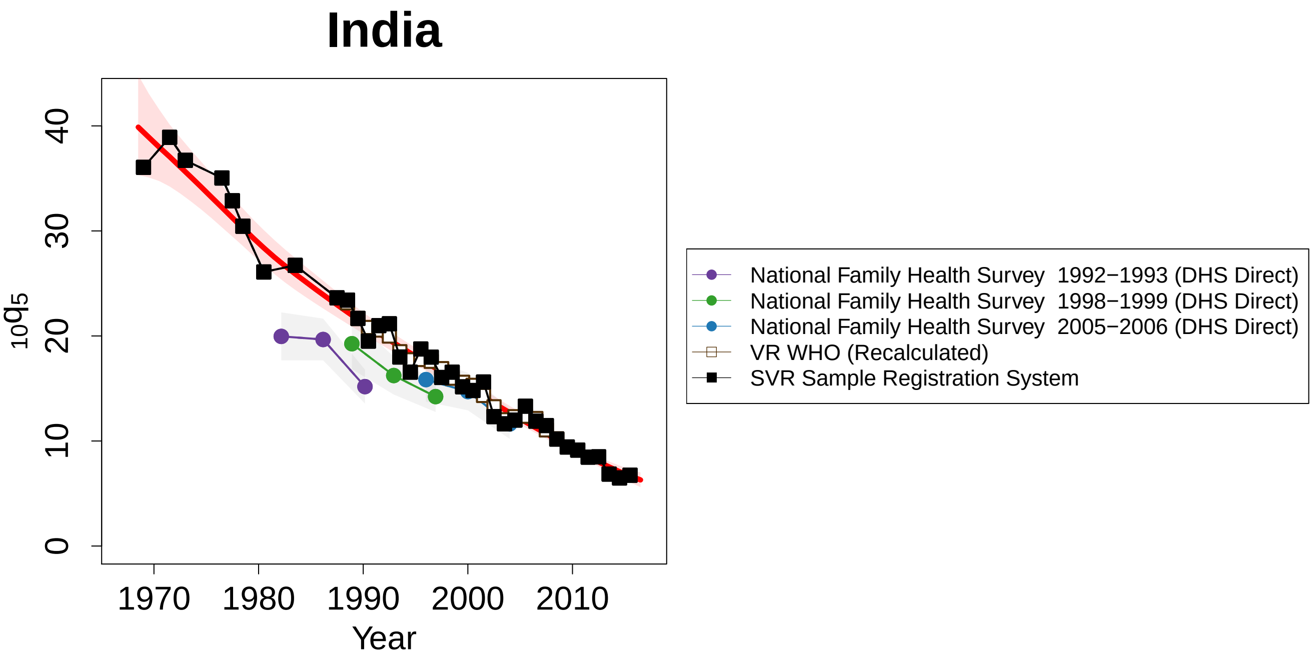
India
40
30
20
10
0
10q5
1970
1980
1990
2000
2010
Year
National Family Health Survey 1992−1993 (DHS Direct)
National Family Health Survey 1998−1999 (DHS Direct)
National Family Health Survey 2005−2006 (DHS Direct)
VR WHO (Recalculated)
SVR Sample Registration System

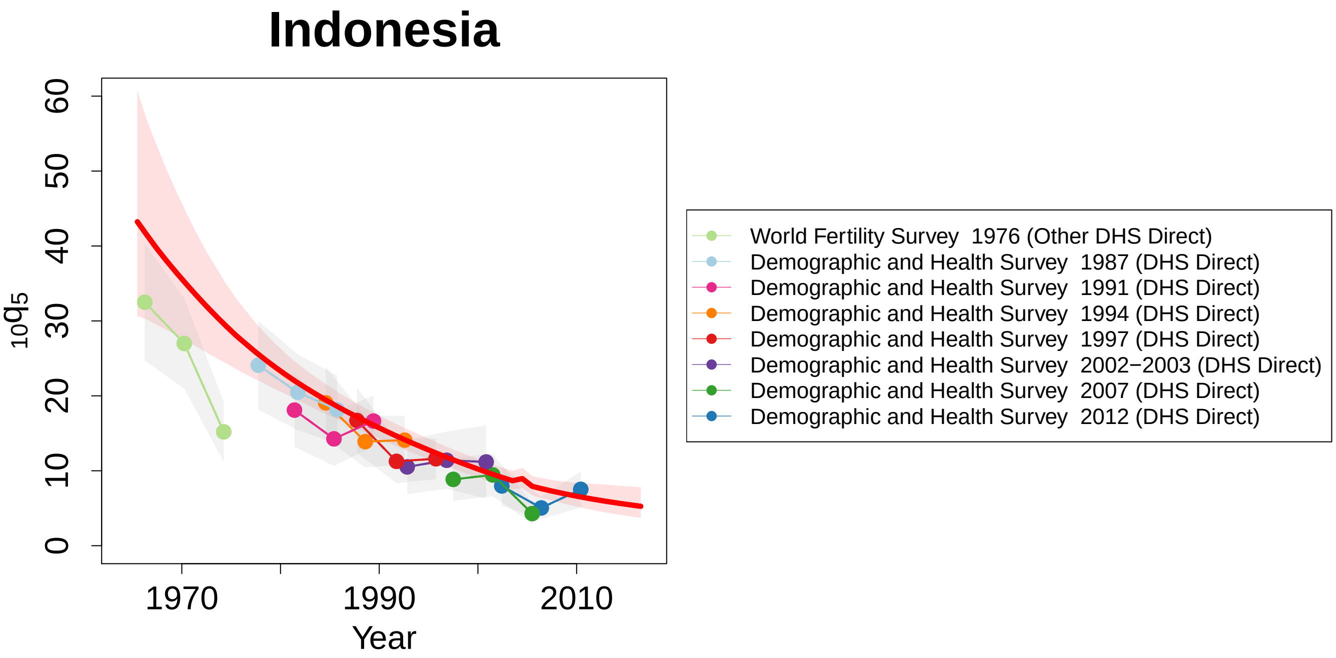
Indonesia
60
50
40
30
20
10
0
$_{10}q_5$
1970
1990
2010
Year
World Fertility Survey 1976 (Other DHS Direct)
Demographic and Health Survey 1987 (DHS Direct)
Demographic and Health Survey 1991 (DHS Direct)
Demographic and Health Survey 1994 (DHS Direct)
Demographic and Health Survey 1997 (DHS Direct)
Demographic and Health Survey 2002−2003 (DHS Direct)
Demographic and Health Survey 2007 (DHS Direct)
Demographic and Health Survey 2012 (DHS Direct)

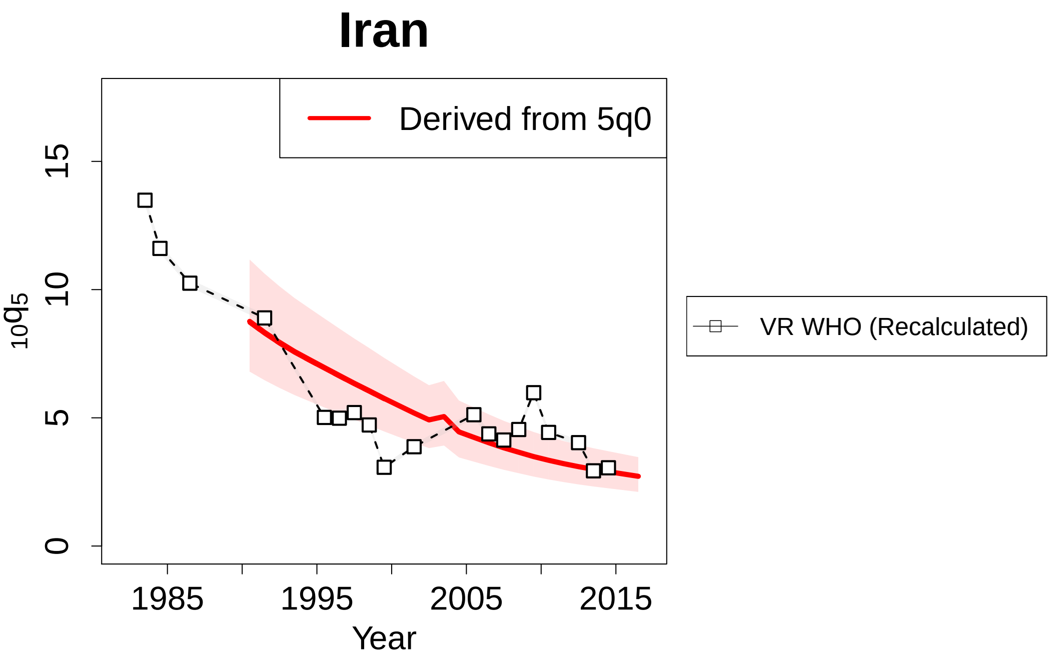
Iran
Derived from 5q0
15
10
5
0
$_{10}q_5$
1985
1995
2005
2015
Year
VR WHO (Recalculated)

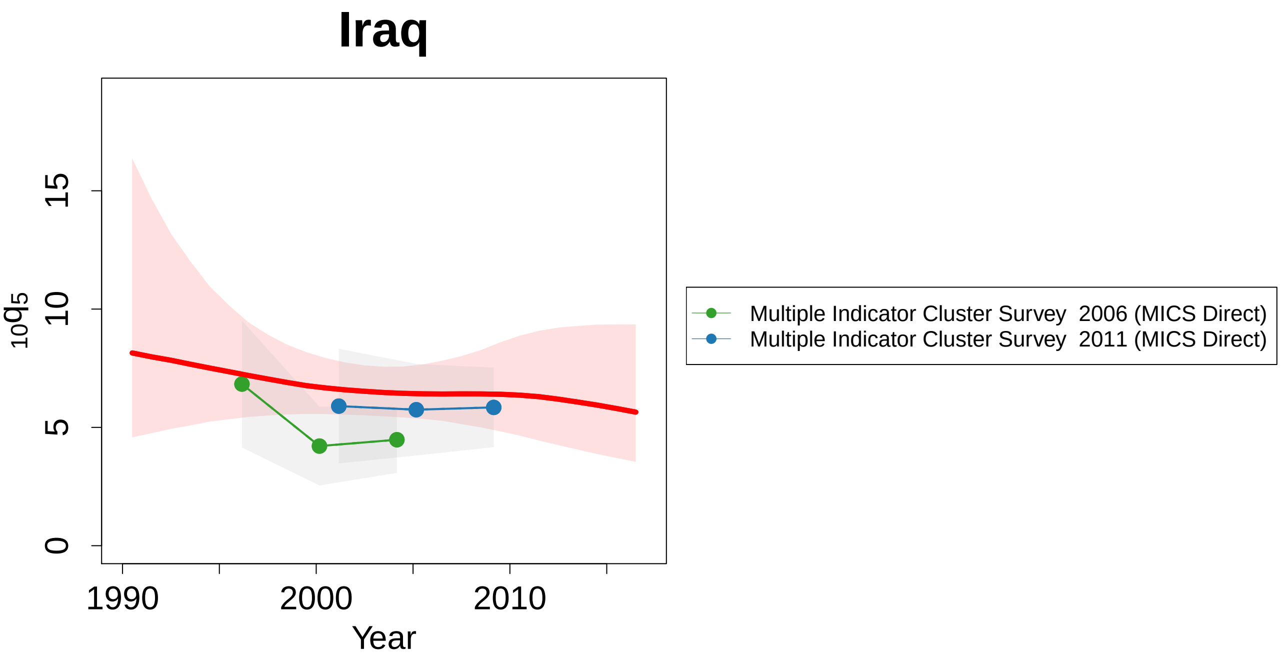
Iraq
0
5
10
15
$_{10}q_5$
1990
2000
2010
Year
Multiple Indicator Cluster Survey 2006 (MICS Direct)
Multiple Indicator Cluster Survey 2011 (MICS Direct)

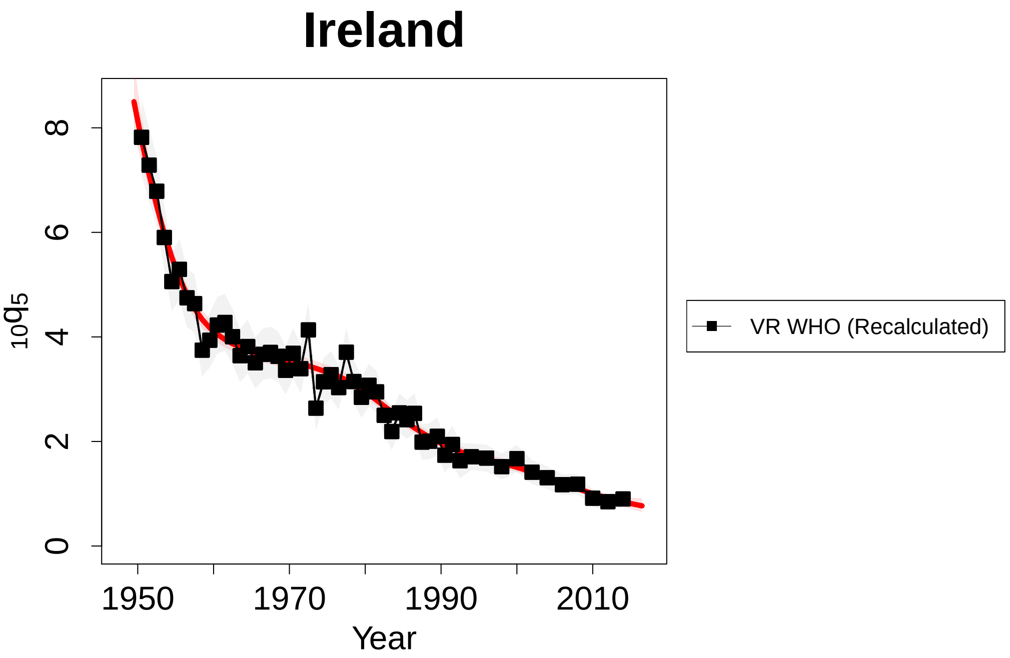
Ireland
0
2
4
6
8
$_{10}q_5$
1950
1970
1990
2010
Year
VR WHO (Recalculated)

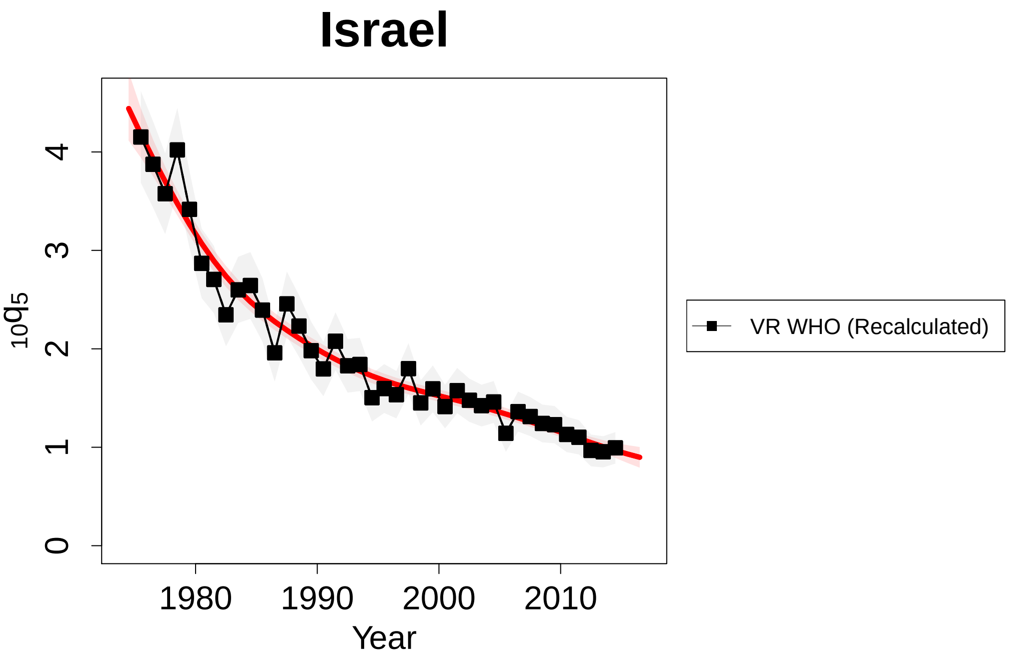
Israel
$_{10}q_5$
0
1
2
3
4
1980
1990
2000
2010
Year
VR WHO (Recalculated)

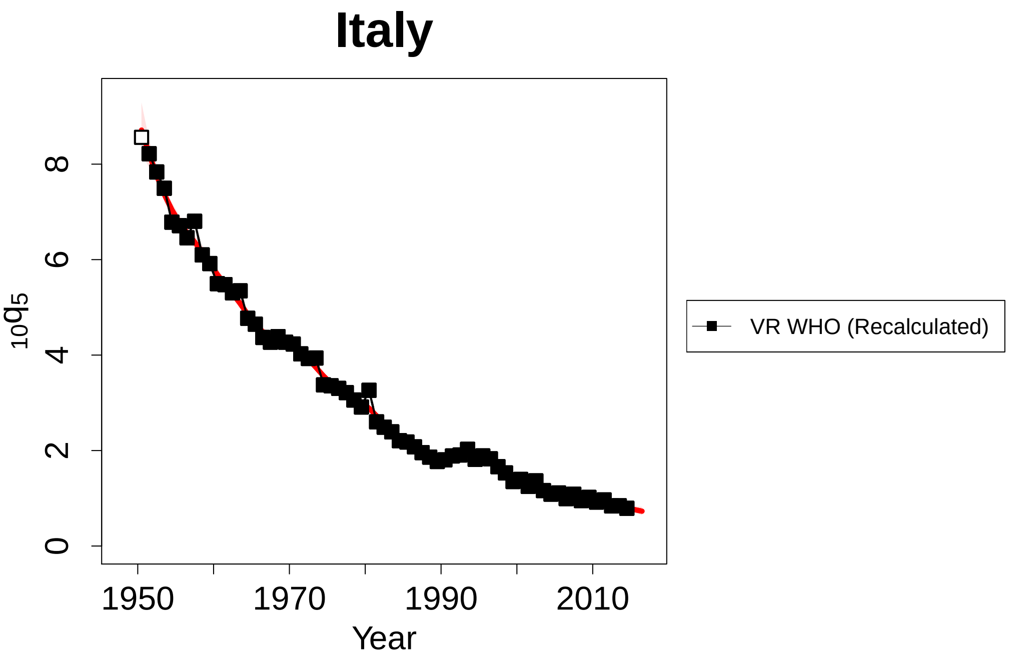
Italy
$_{10}q_5$
0
2
4
6
8
1950
1970
1990
2010
Year
VR WHO (Recalculated)

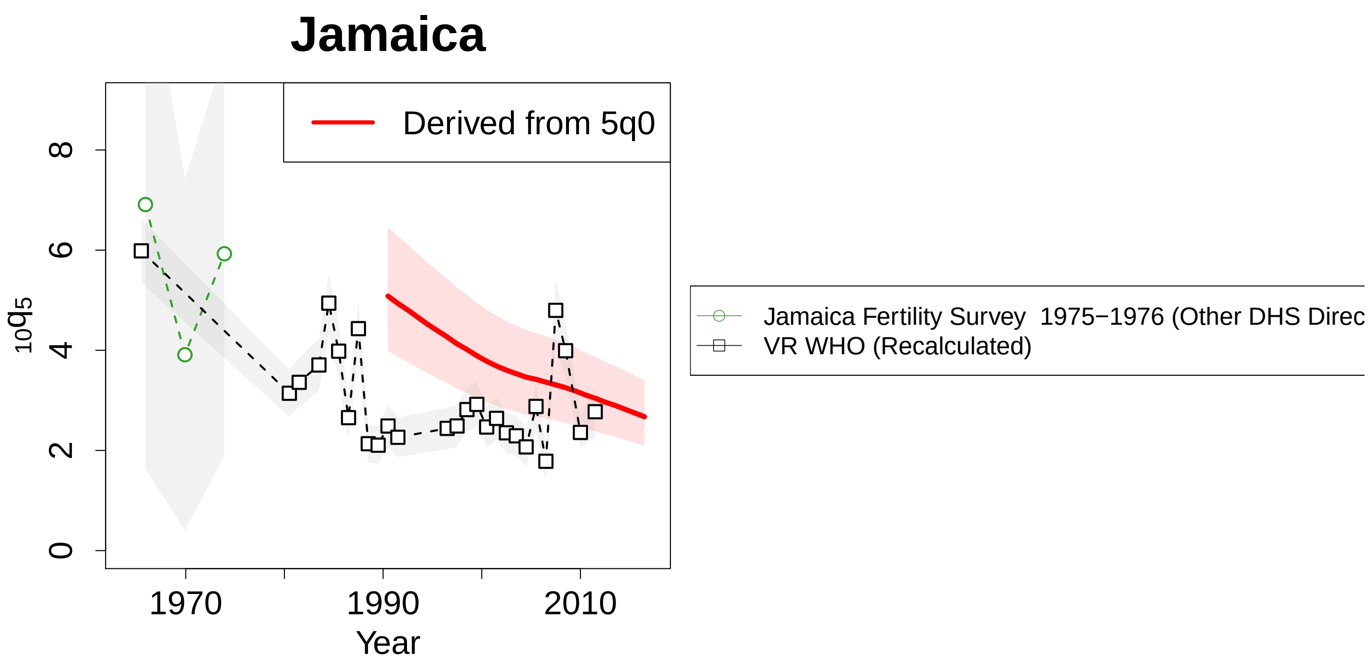
Jamaica
Derived from 5q0
Jamaica Fertility Survey 1975−1976 (Other DHS Direc
VR WHO (Recalculated)
$_{10}q_5$
0
2
4
6
8
1970
1990
2010
Year

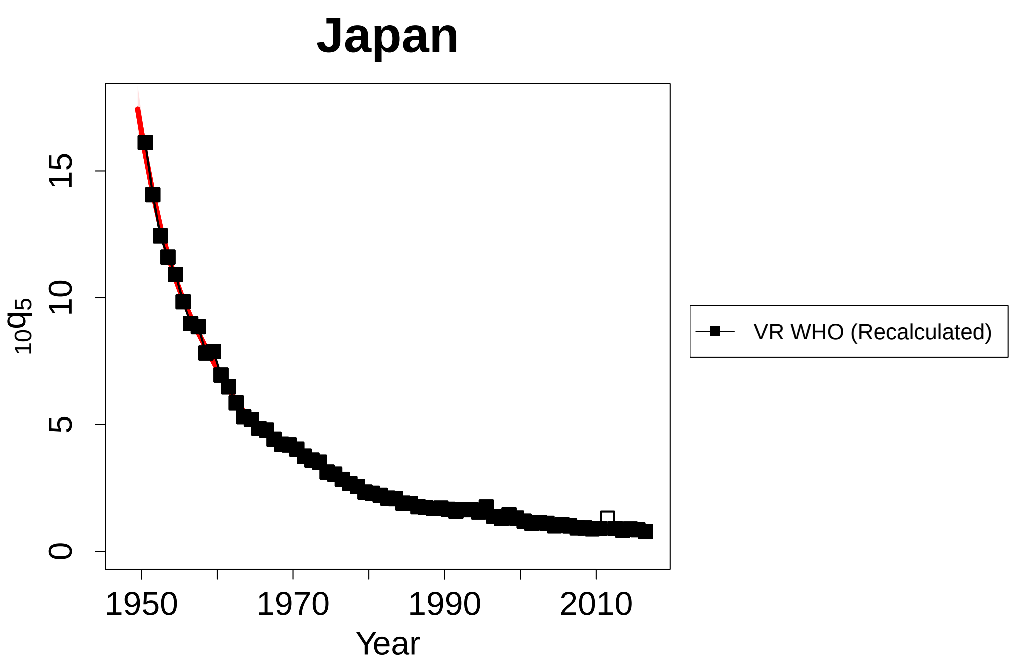
Japan
VR WHO (Recalculated)
$_{10}q_5$
0
5
10
15
1950
1970
1990
2010
Year

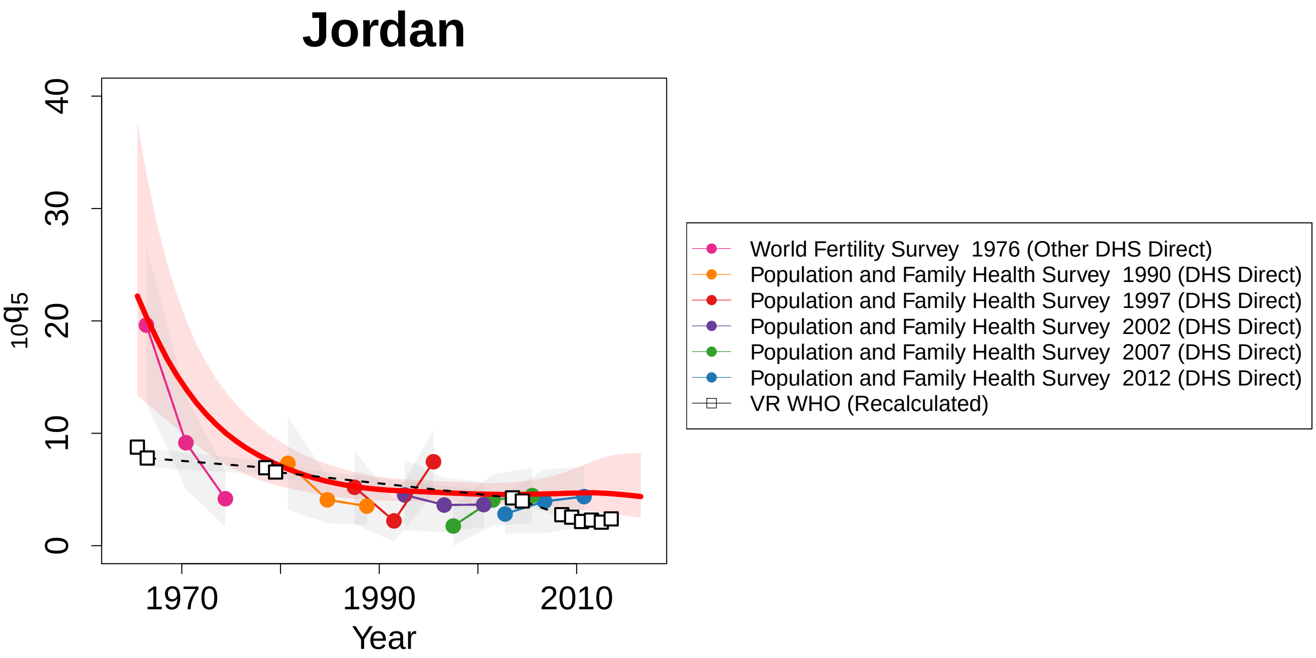
Jordan
40
30
20
10
0
$_{10}q_5$
1970
1990
2010
Year
World Fertility Survey 1976 (Other DHS Direct)
Population and Family Health Survey 1990 (DHS Direct)
Population and Family Health Survey 1997 (DHS Direct)
Population and Family Health Survey 2002 (DHS Direct)
Population and Family Health Survey 2007 (DHS Direct)
Population and Family Health Survey 2012 (DHS Direct)
VR WHO (Recalculated)

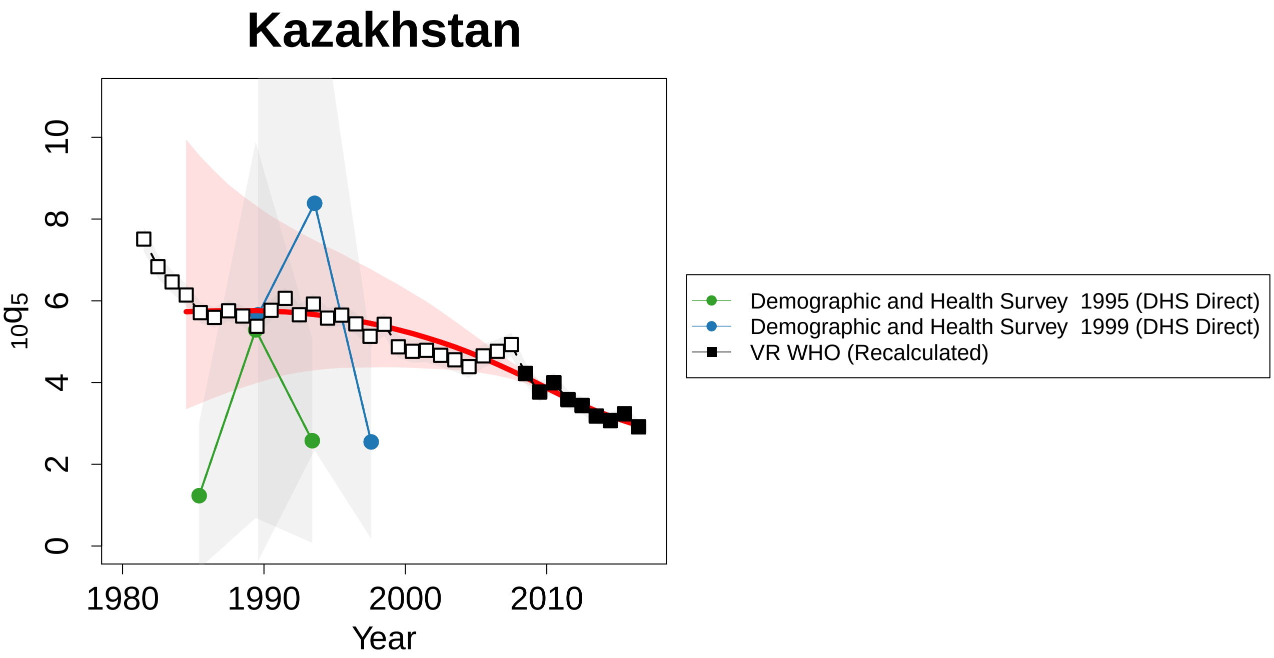
Kazakhstan
10
8
6
4
2
0
$_{10}q_5$
1980
1990
2000
2010
Year
Demographic and Health Survey 1995 (DHS Direct)
Demographic and Health Survey 1999 (DHS Direct)
VR WHO (Recalculated)

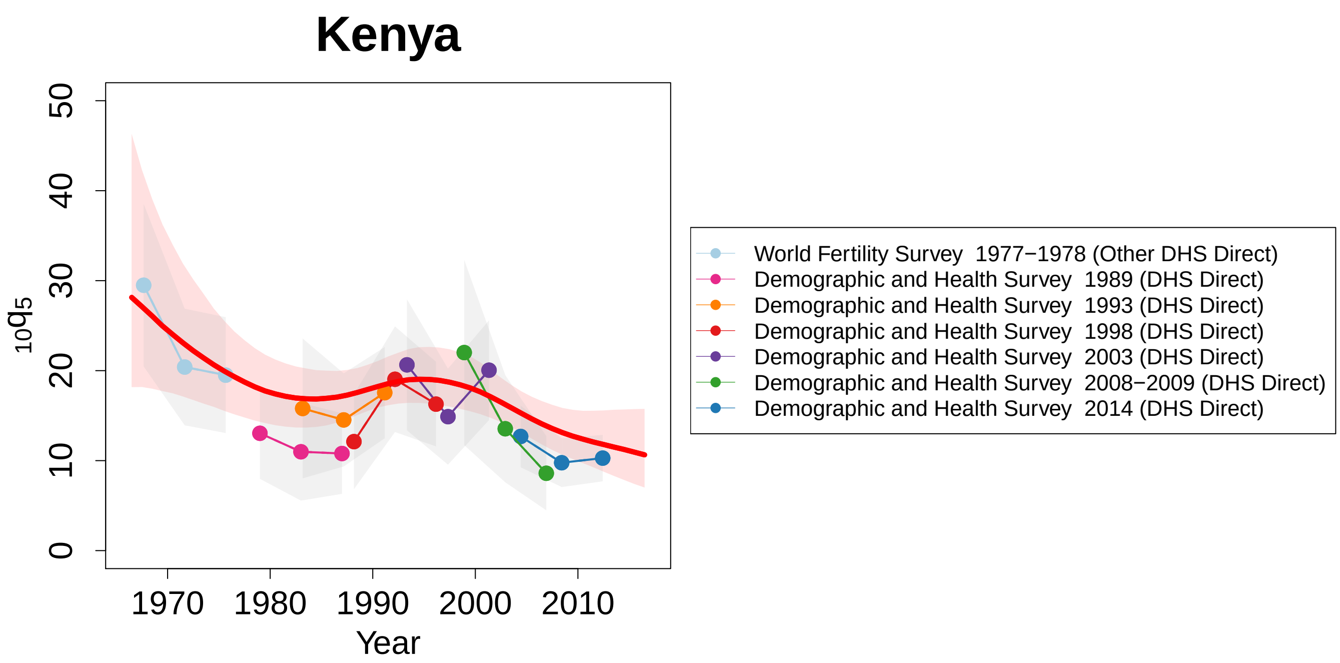
Kenya
$_{10}q_5$
0
10
20
30
40
50
1970
1980
1990
2000
2010
Year
World Fertility Survey 1977−1978 (Other DHS Direct)
Demographic and Health Survey 1989 (DHS Direct)
Demographic and Health Survey 1993 (DHS Direct)
Demographic and Health Survey 1998 (DHS Direct)
Demographic and Health Survey 2003 (DHS Direct)
Demographic and Health Survey 2008−2009 (DHS Direct)
Demographic and Health Survey 2014 (DHS Direct)

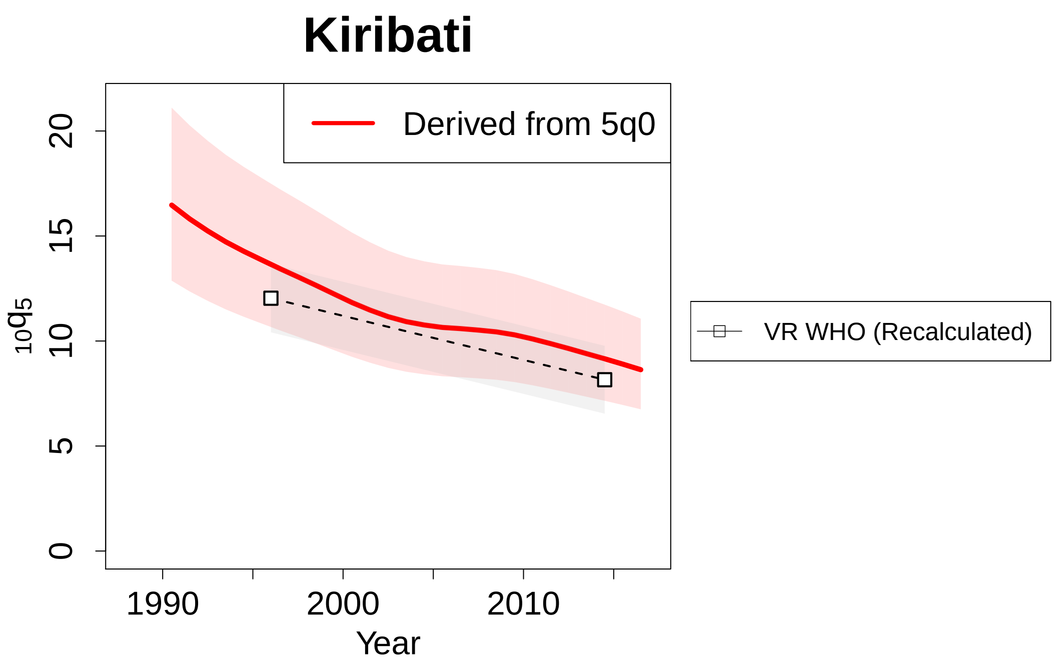
Kiribati
Derived from 5q0
$_{10}q_5$
0
5
10
15
20
1990
2000
2010
Year
VR WHO (Recalculated)

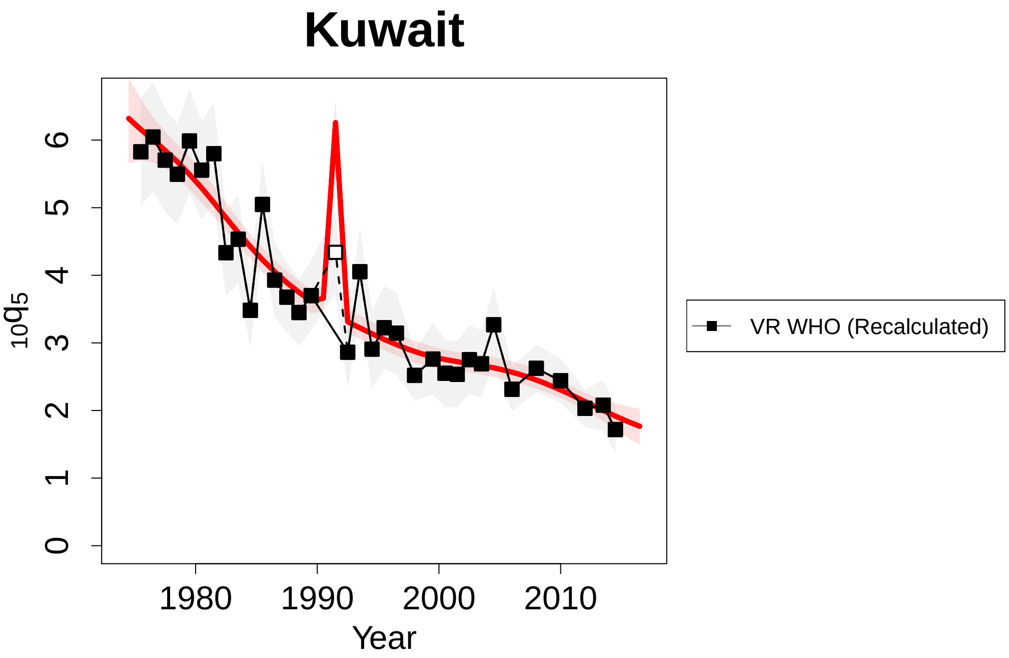
Kuwait
6
5
4
3
2
1
0
$_{10}q_5$
1980
1990
2000
2010
Year
VR WHO (Recalculated)

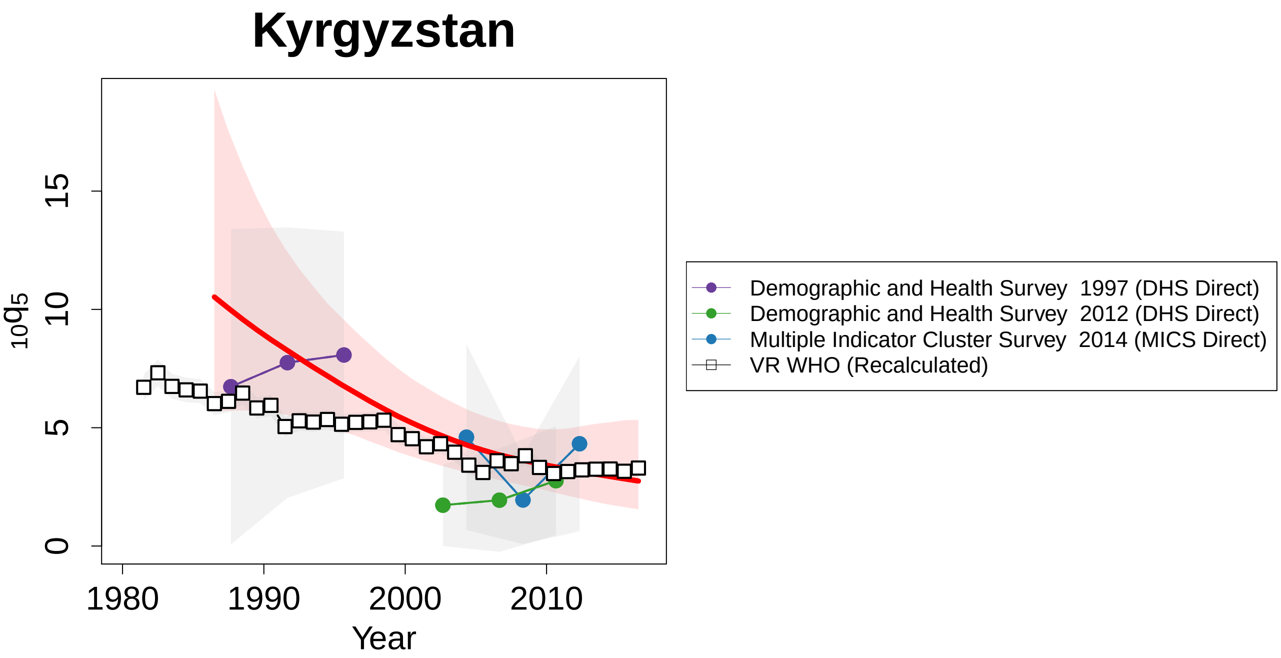
Kyrgyzstan
15
10
5
0
$_{10}q_5$
1980
1990
2000
2010
Year
Demographic and Health Survey 1997 (DHS Direct)
Demographic and Health Survey 2012 (DHS Direct)
Multiple Indicator Cluster Survey 2014 (MICS Direct)
VR WHO (Recalculated)

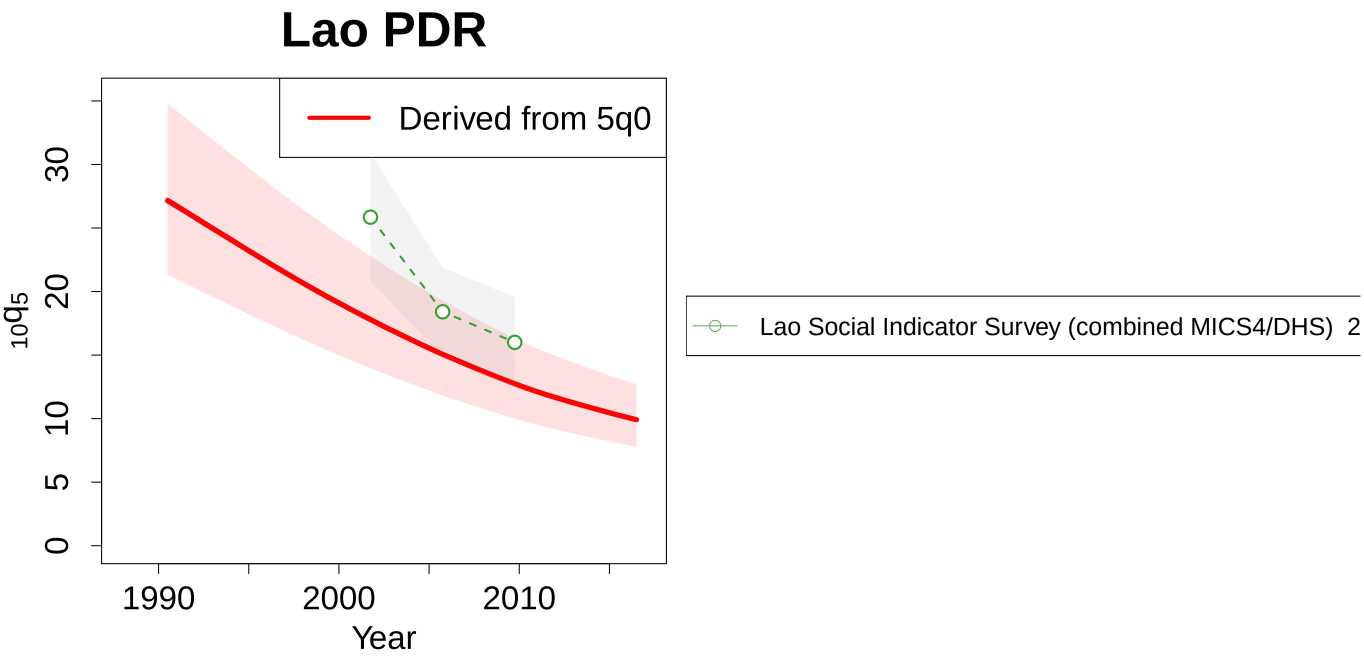
Lao PDR
Derived from 5q0
Lao Social Indicator Survey (combined MICS4/DHS) 2
$_{10}q_5$
0
5
10
20
30
1990
2000
2010
Year

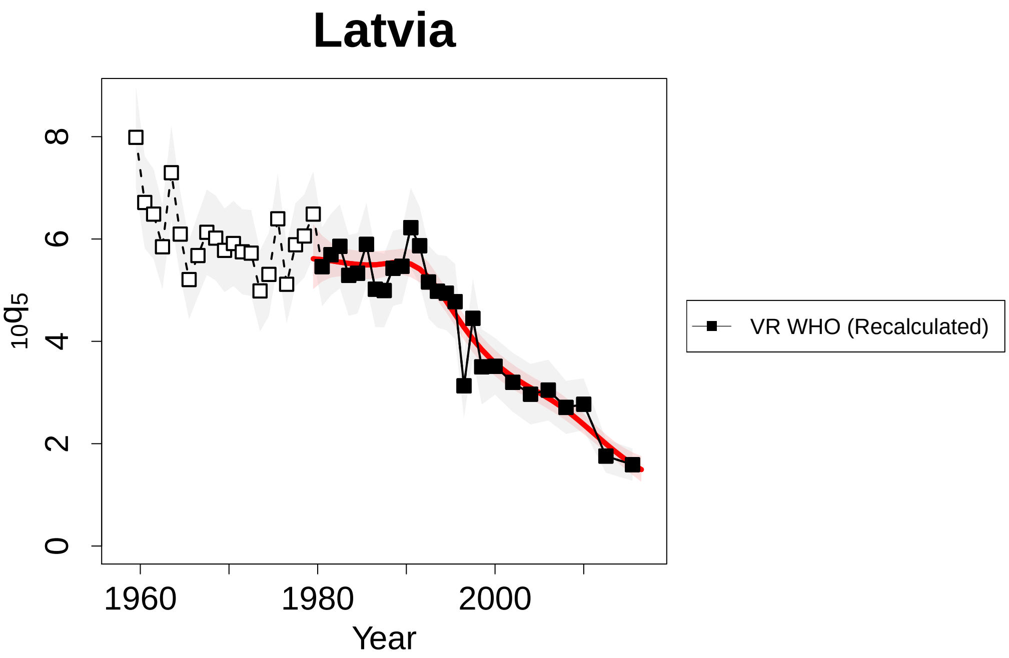
Latvia
VR WHO (Recalculated)
$_{10}q_5$
0
2
4
6
8
1960
1980
2000
Year

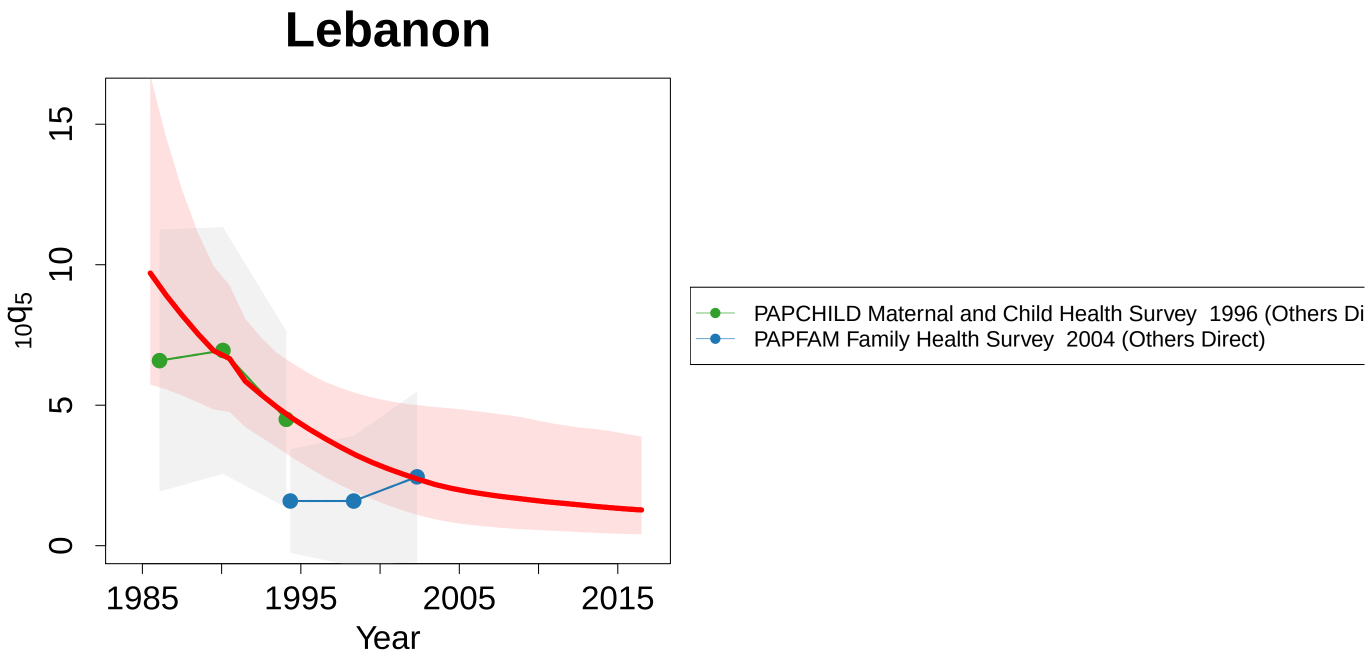
Lebanon
15
10
5
0
$_{10}q_5$
1985
1995
2005
2015
Year
PAPCHILD Maternal and Child Health Survey 1996 (Others Di
PAPFAM Family Health Survey 2004 (Others Direct)

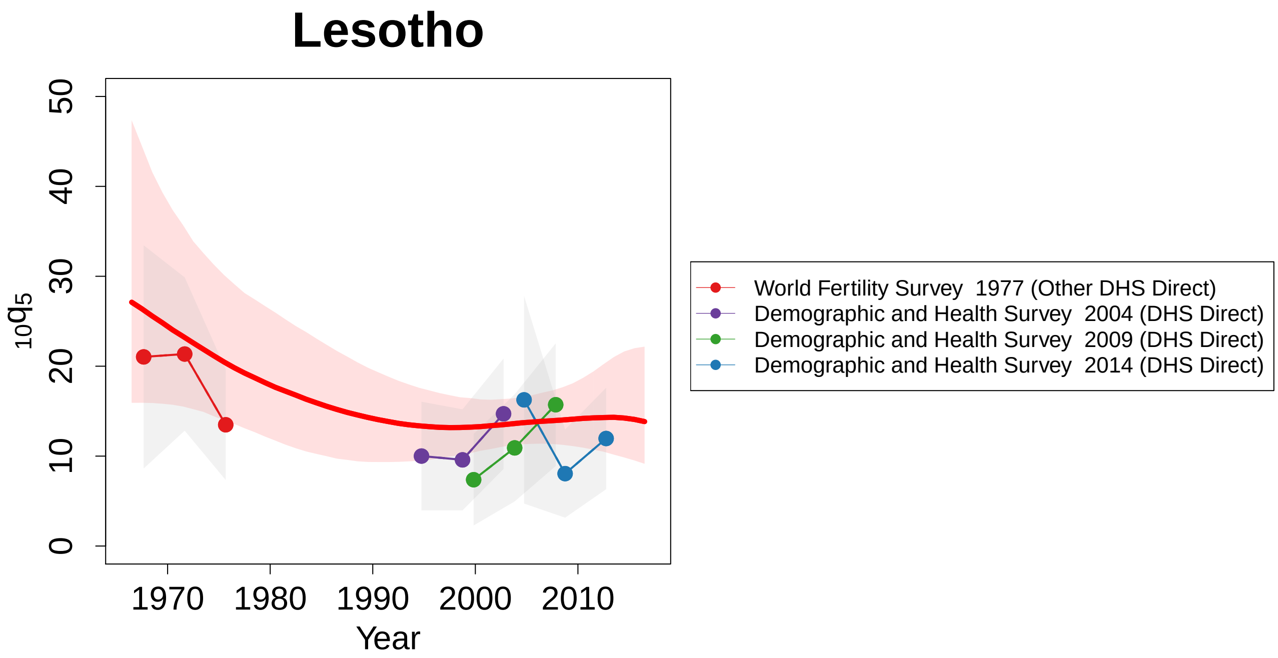
Lesotho
50
40
30
20
10
0
$_{10}q_5$
1970
1980
1990
2000
2010
Year
World Fertility Survey 1977 (Other DHS Direct)
Demographic and Health Survey 2004 (DHS Direct)
Demographic and Health Survey 2009 (DHS Direct)
Demographic and Health Survey 2014 (DHS Direct)

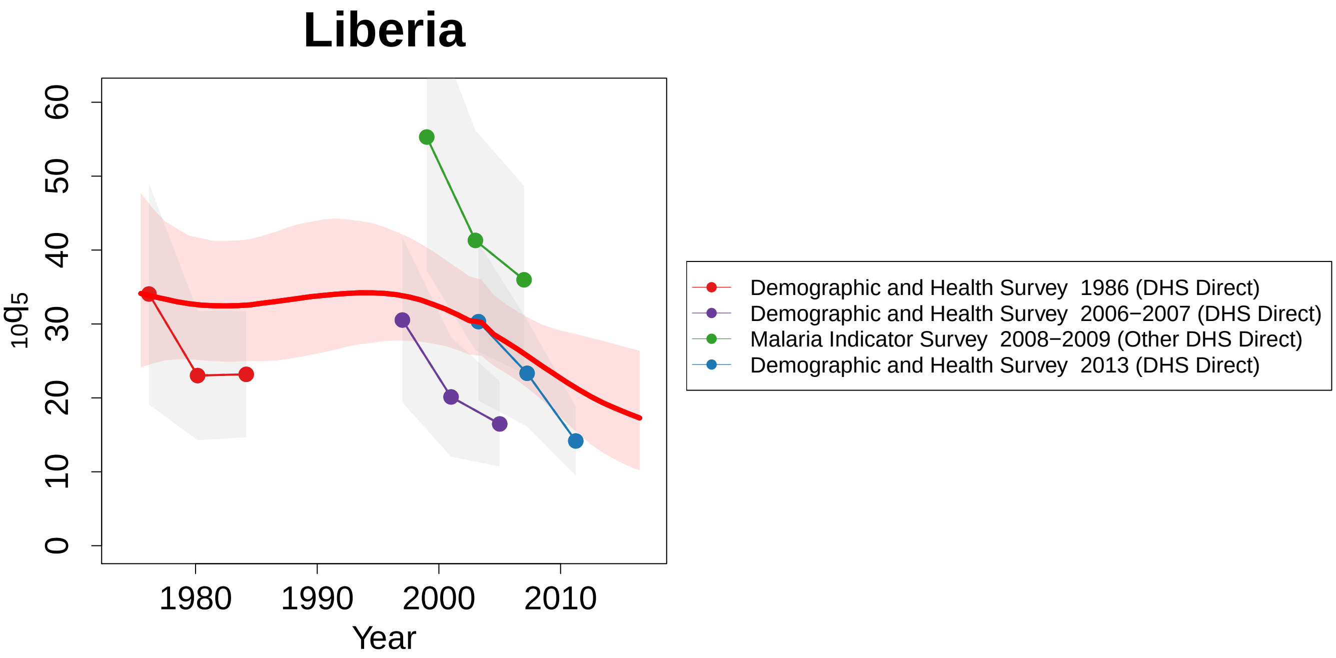
Liberia
60
50
40
30
20
10
0
$_{10}q_5$
1980
1990
2000
2010
Year
Demographic and Health Survey 1986 (DHS Direct)
Demographic and Health Survey 2006−2007 (DHS Direct)
Malaria Indicator Survey 2008−2009 (Other DHS Direct)
Demographic and Health Survey 2013 (DHS Direct)

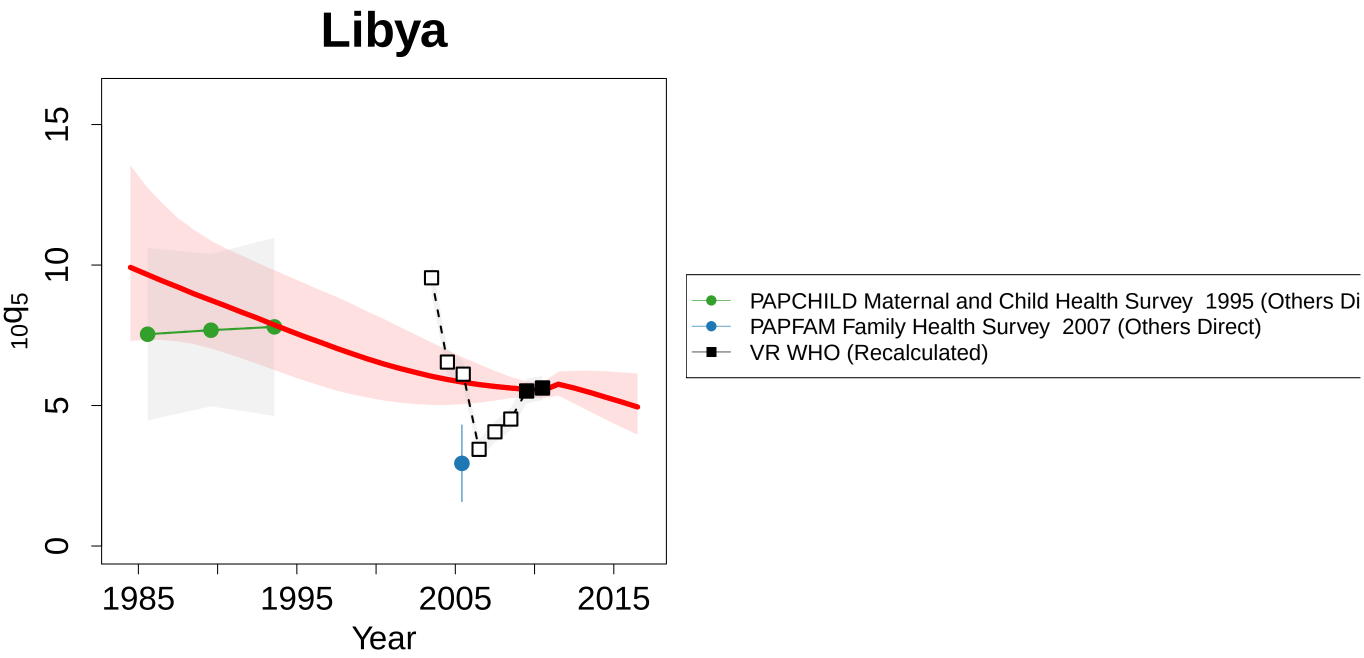
Libya
15
10
5
0
$_{10}q_5$
1985
1995
2005
2015
Year
PAPCHILD Maternal and Child Health Survey 1995 (Others Di
PAPFAM Family Health Survey 2007 (Others Direct)
VR WHO (Recalculated)

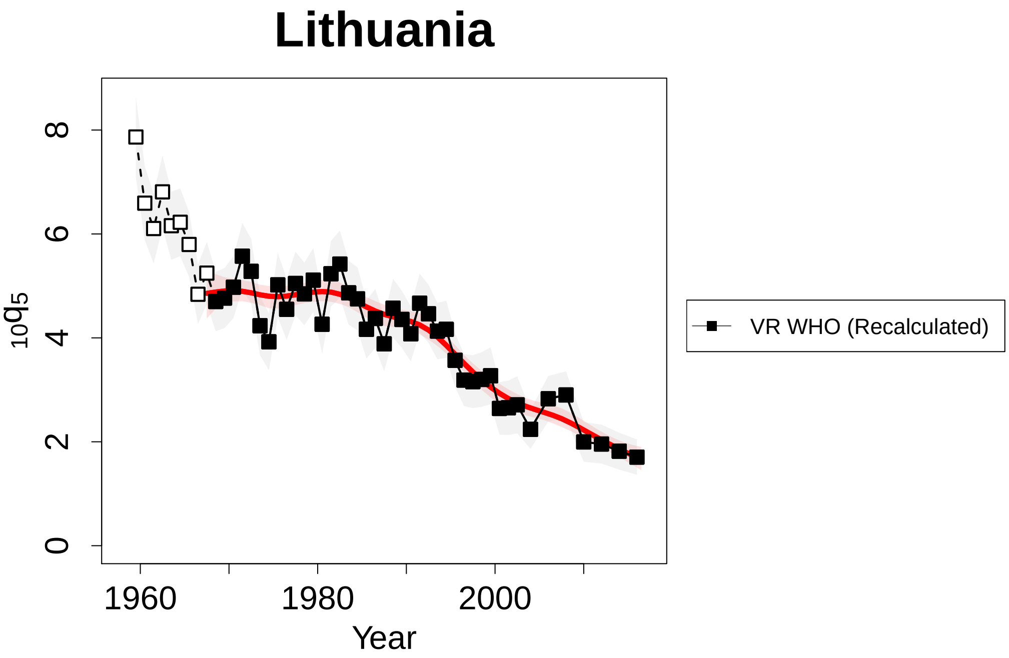
Lithuania
8
6
4
2
0
$_{10}q_5$
1960
1980
2000
Year
VR WHO (Recalculated)

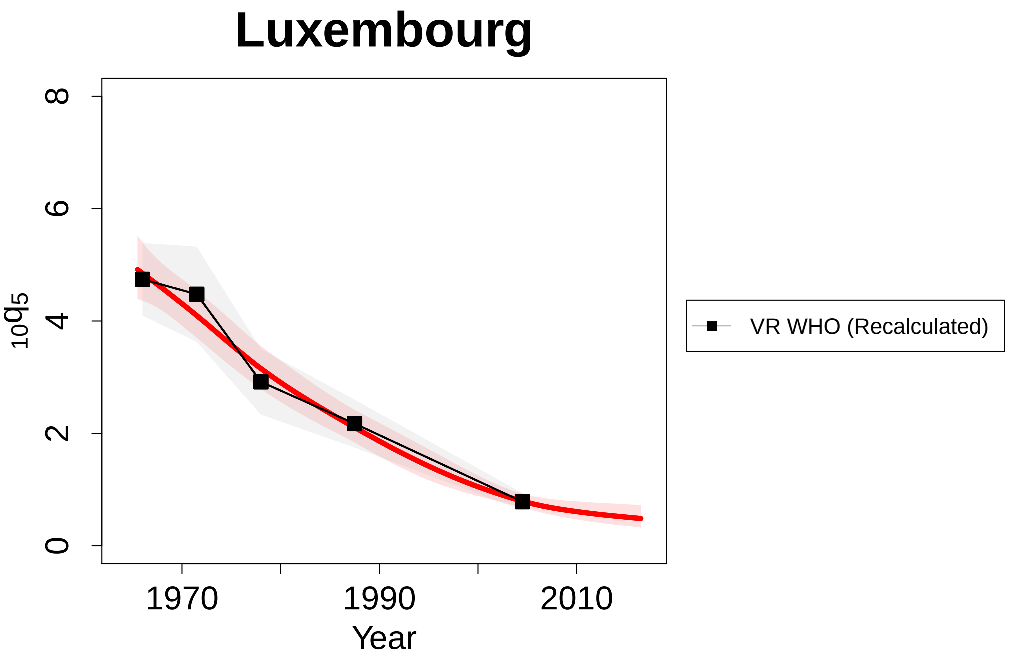
Luxembourg
8
6
4
2
0
$_{10}q_5$
1970
1990
2010
Year
VR WHO (Recalculated)

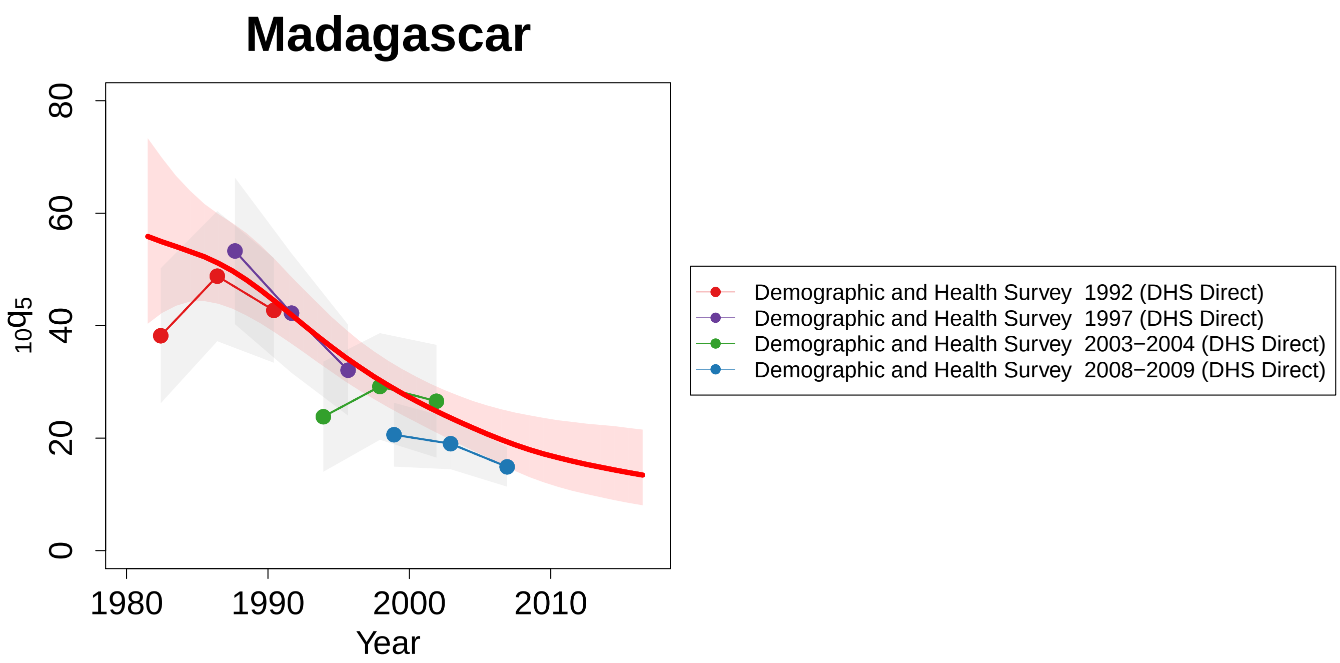
Madagascar
80
60
40
20
0
$_{10}q_5$
1980
1990
2000
2010
Year
Demographic and Health Survey 1992 (DHS Direct)
Demographic and Health Survey 1997 (DHS Direct)
Demographic and Health Survey 2003−2004 (DHS Direct)
Demographic and Health Survey 2008−2009 (DHS Direct)

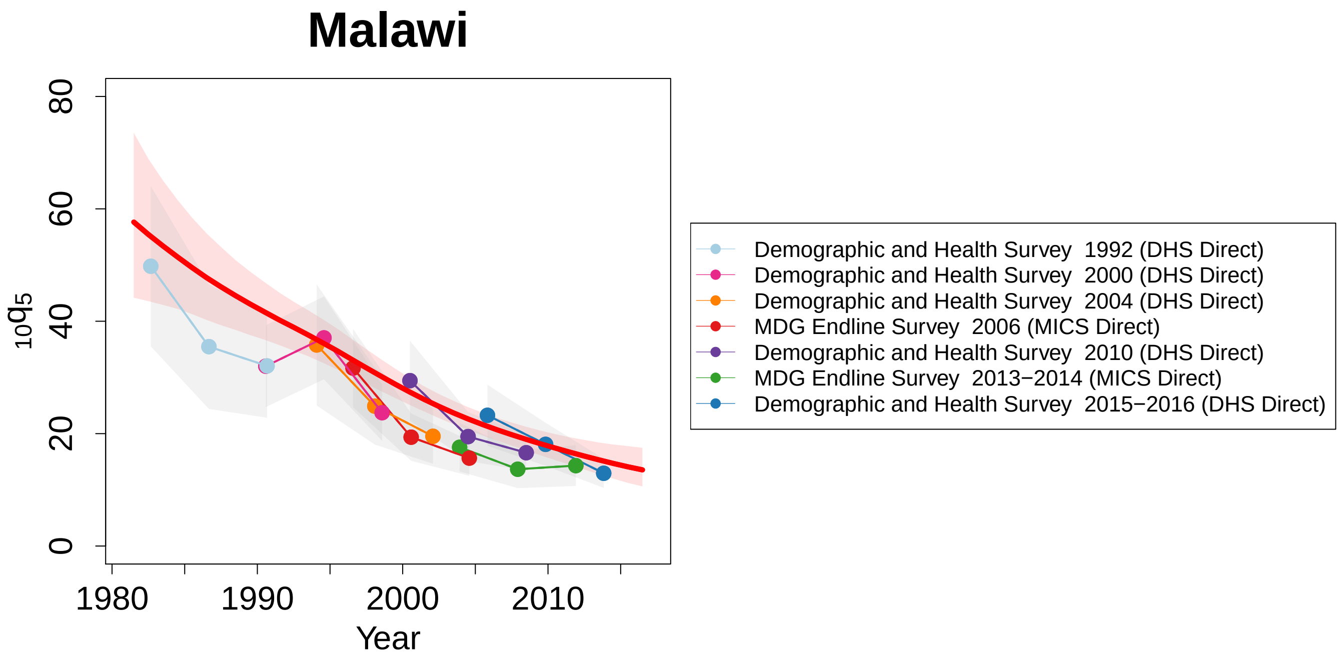
Malawi
80
60
40
20
0
$_{10}q_5$
1980
1990
2000
2010
Year
Demographic and Health Survey 1992 (DHS Direct)
Demographic and Health Survey 2000 (DHS Direct)
Demographic and Health Survey 2004 (DHS Direct)
MDG Endline Survey 2006 (MICS Direct)
Demographic and Health Survey 2010 (DHS Direct)
MDG Endline Survey 2013−2014 (MICS Direct)
Demographic and Health Survey 2015−2016 (DHS Direct)

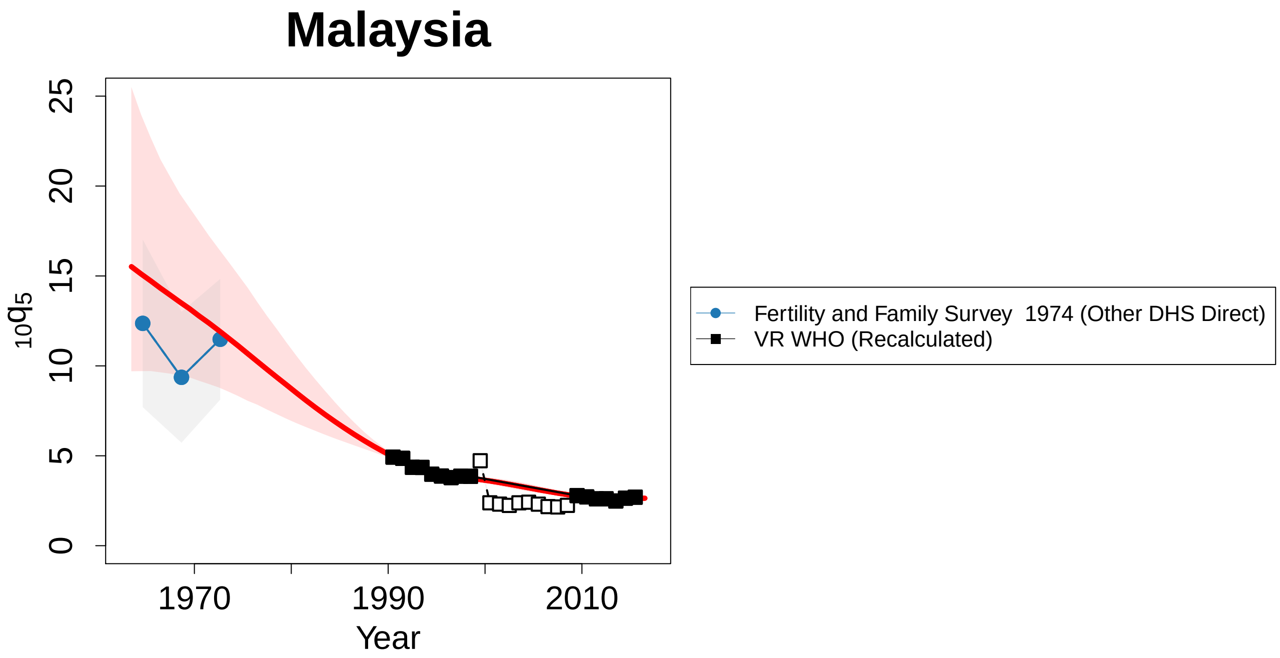
Malaysia
25
20
15
10
5
0
$_{10}q_5$
1970
1990
2010
Year
Fertility and Family Survey 1974 (Other DHS Direct)
VR WHO (Recalculated)

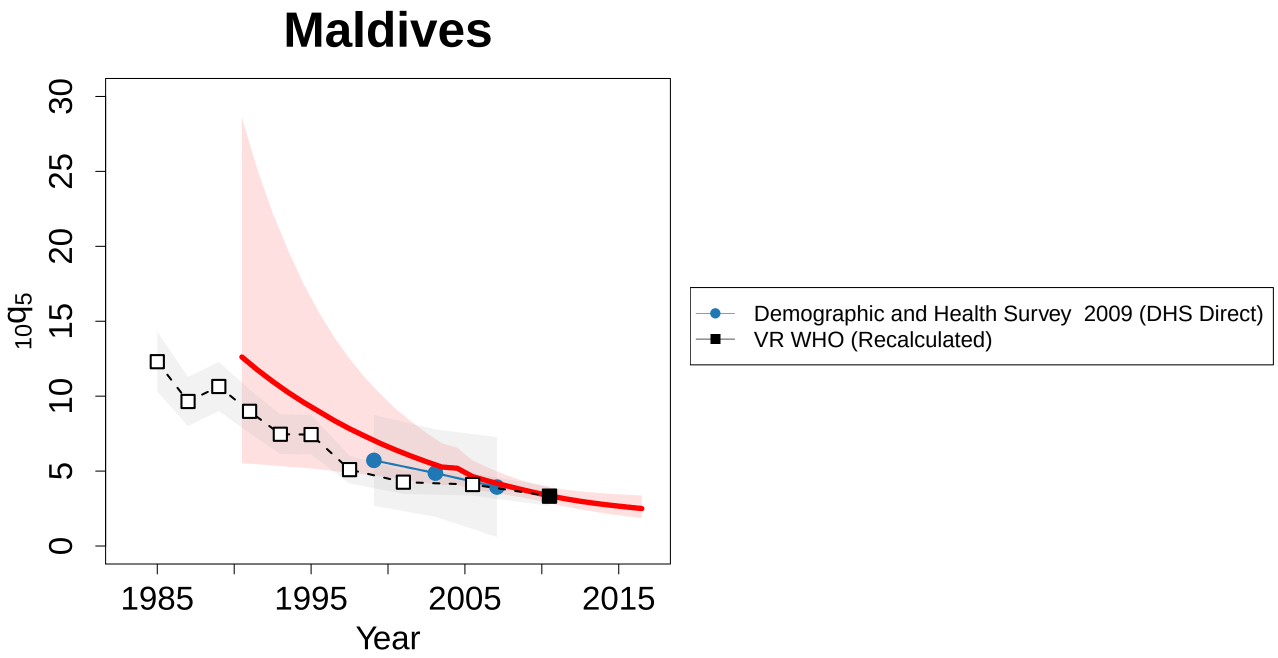
Maldives
30
25
20
15
10
5
0
$_{10}q_5$
1985
1995
2005
2015
Year
Demographic and Health Survey 2009 (DHS Direct)
VR WHO (Recalculated)

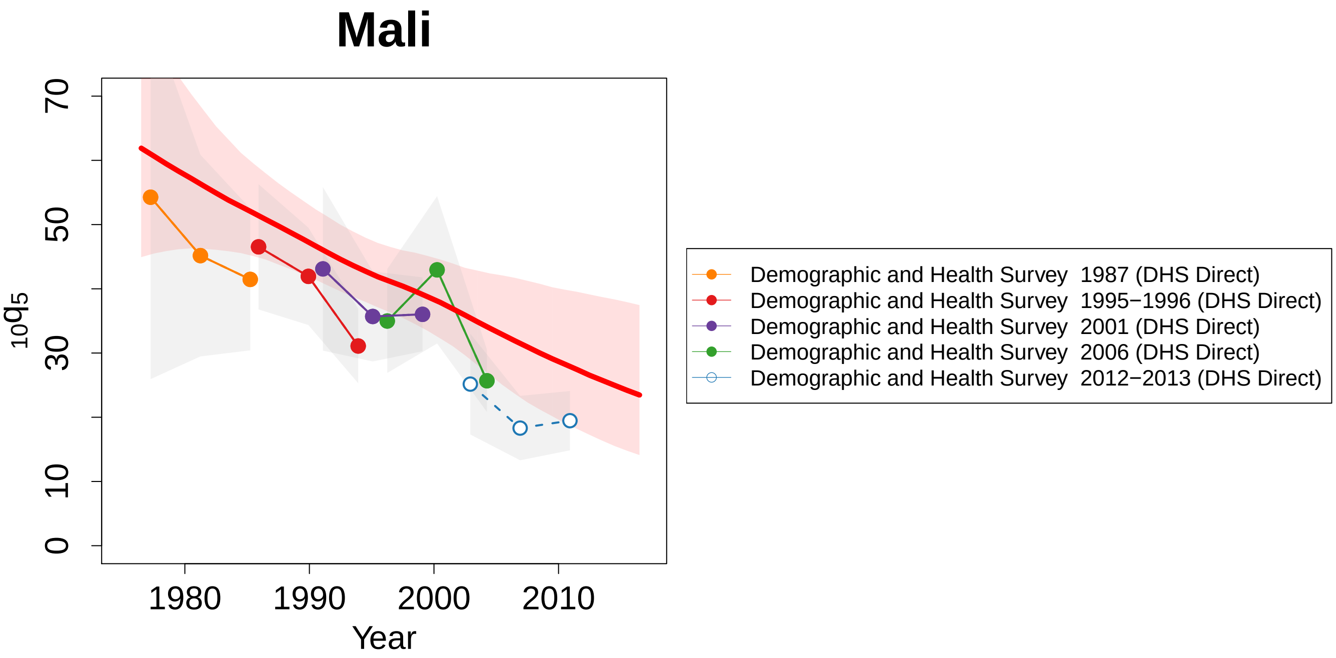
Mali
70
50
30
10
0
$_{10}q_5$
1980
1990
2000
2010
Year
Demographic and Health Survey 1987 (DHS Direct)
Demographic and Health Survey 1995−1996 (DHS Direct)
Demographic and Health Survey 2001 (DHS Direct)
Demographic and Health Survey 2006 (DHS Direct)
Demographic and Health Survey 2012−2013 (DHS Direct)

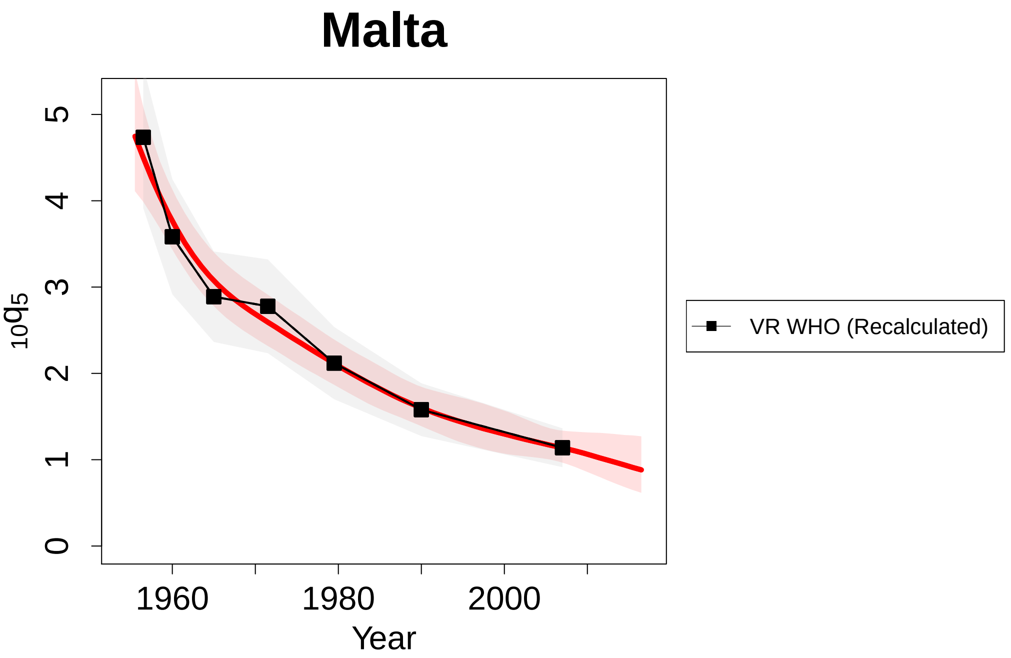
Malta
5
4
3
2
1
0
$_{10}q_5$
1960
1980
2000
Year
VR WHO (Recalculated)

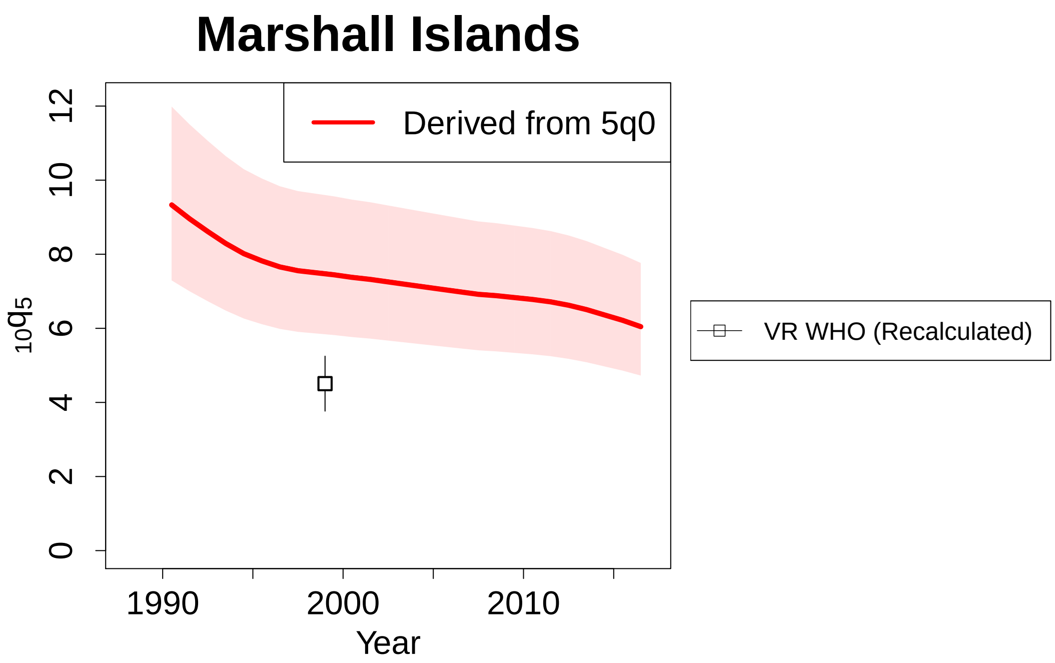
Marshall Islands
Derived from 5q0
VR WHO (Recalculated)
$_{10}q_5$
0
2
4
6
8
10
12
1990
2000
2010
Year

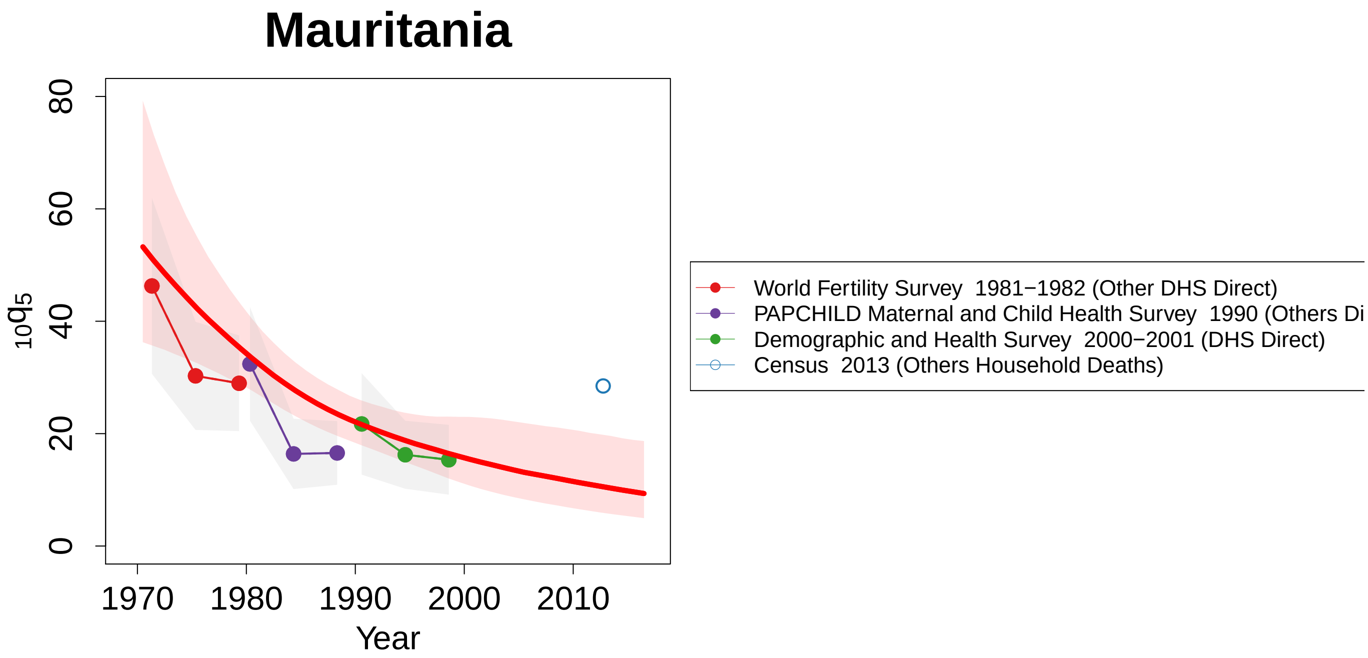
Mauritania
World Fertility Survey 1981−1982 (Other DHS Direct)
PAPCHILD Maternal and Child Health Survey 1990 (Others Di
Demographic and Health Survey 2000−2001 (DHS Direct)
Census 2013 (Others Household Deaths)
$_{10}q_5$
0
20
40
60
80
1970
1980
1990
2000
2010
Year

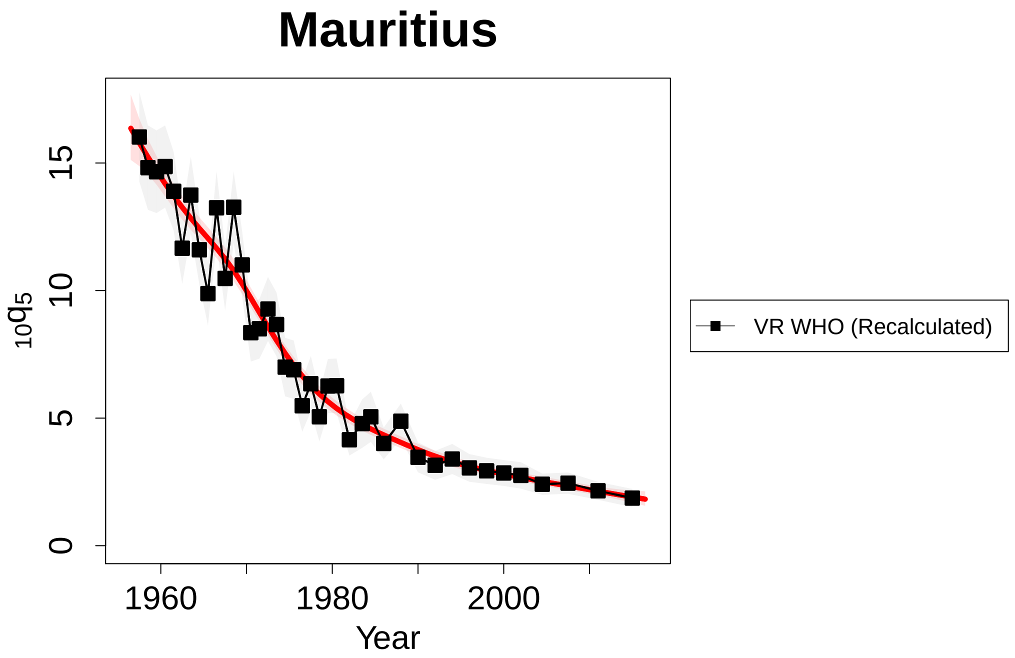
Mauritius
$_{10}q_5$
0
5
10
15
1960
1980
2000
Year
VR WHO (Recalculated)

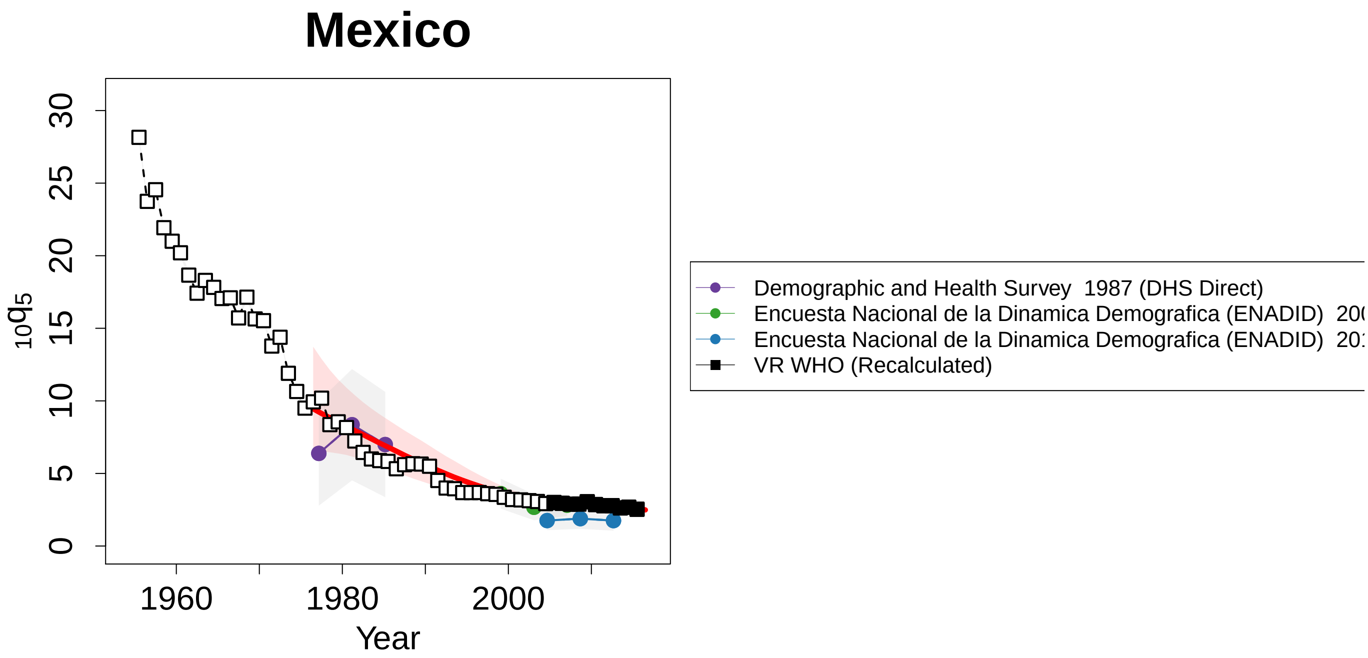
Mexico
$_{10}q_5$
0
5
10
15
20
25
30
1960
1980
2000
Year
Demographic and Health Survey 1987 (DHS Direct)
Encuesta Nacional de la Dinamica Demografica (ENADID) 20
Encuesta Nacional de la Dinamica Demografica (ENADID) 20
VR WHO (Recalculated)

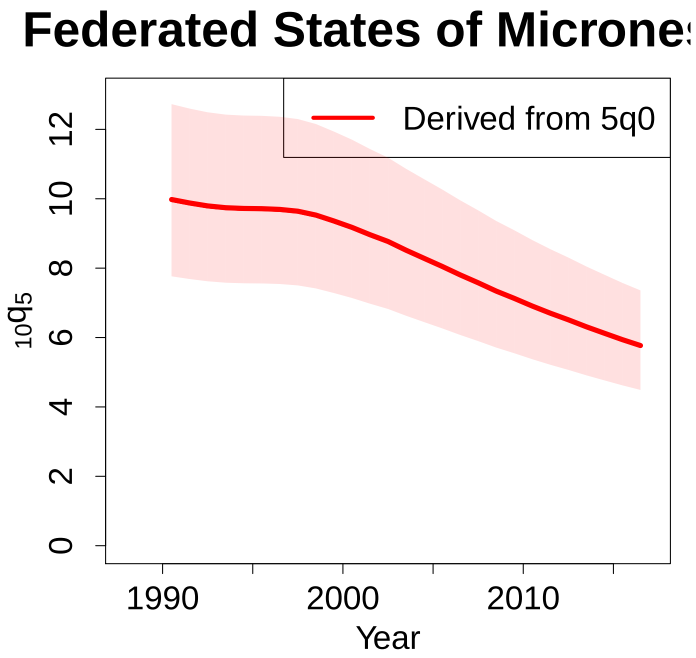
Federated States of Micrones
Derived from 5q0
$_{10}q_5$
0
2
4
6
8
10
12
1990
2000
2010
Year

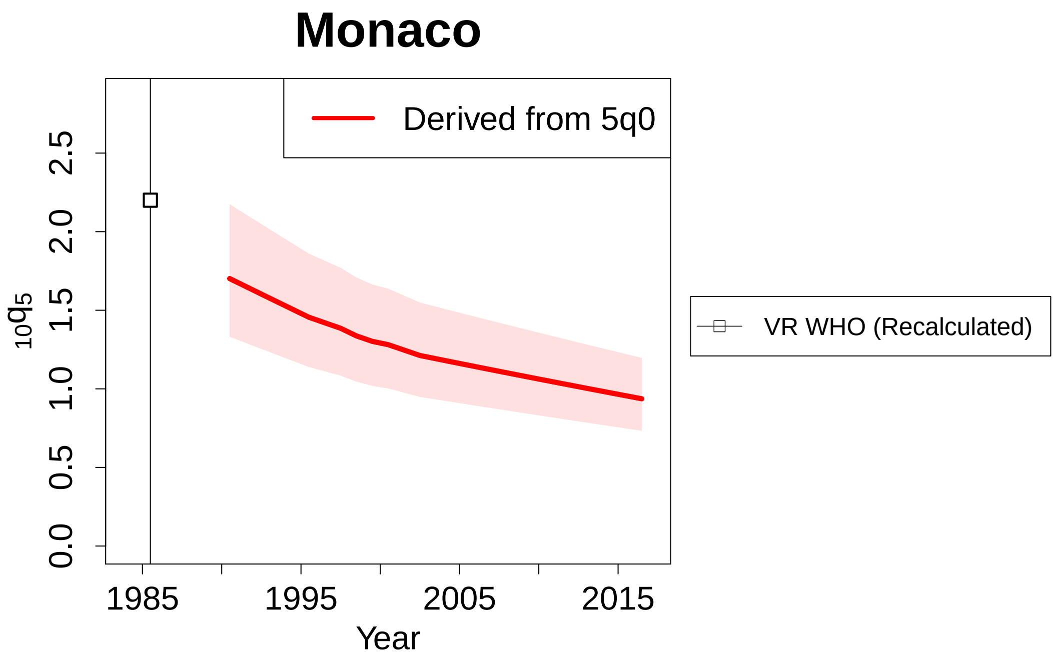
Monaco
Derived from 5q0
VR WHO (Recalculated)
$_{10}q_5$
0.0
0.5
1.0
1.5
2.0
2.5
1985
1995
2005
2015
Year

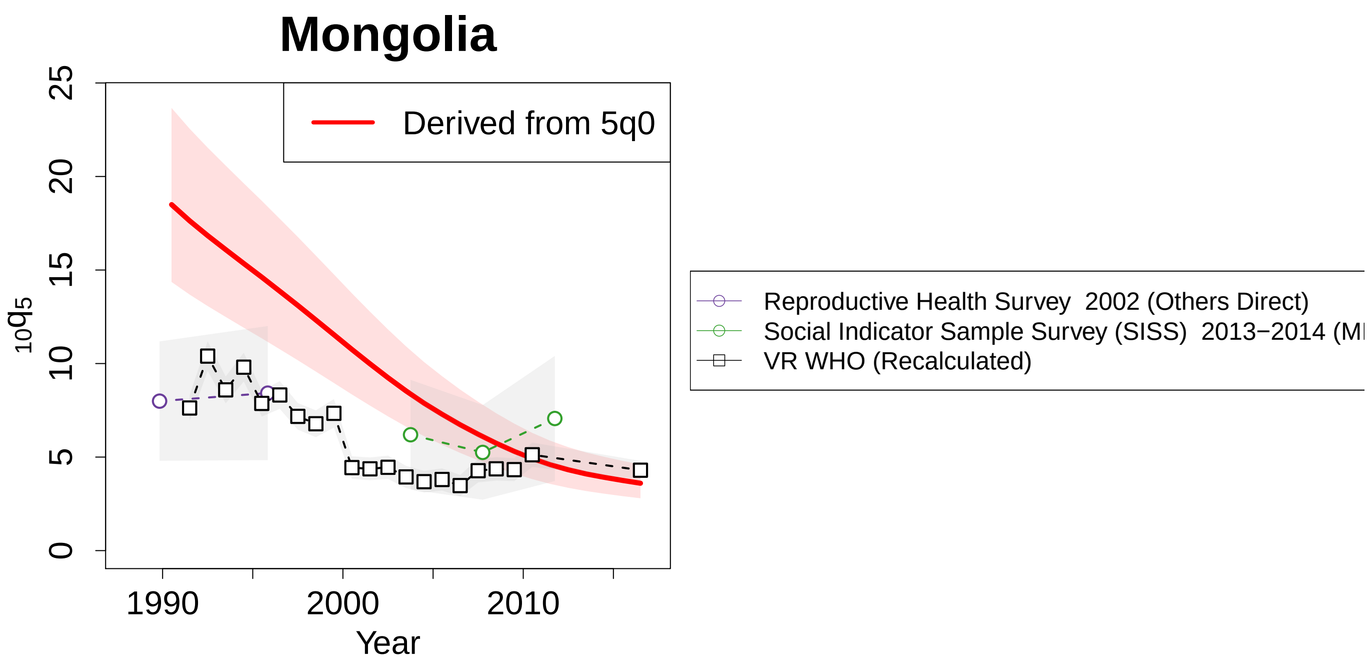
Mongolia
Derived from 5q0
Reproductive Health Survey 2002 (Others Direct)
Social Indicator Sample Survey (SISS) 2013−2014 (M
VR WHO (Recalculated)
$_{10}q_5$
0
5
10
15
20
25
1990
2000
2010
Year

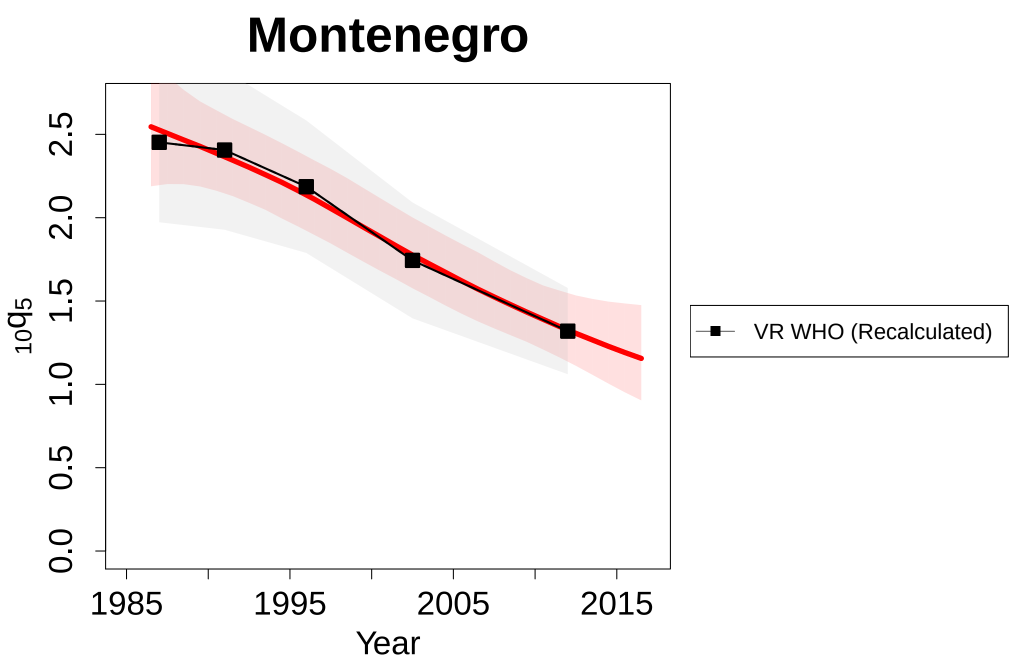
Montenegro
VR WHO (Recalculated)
$_{10}q_5$
0.0
0.5
1.0
1.5
2.0
2.5
1985
1995
2005
2015
Year

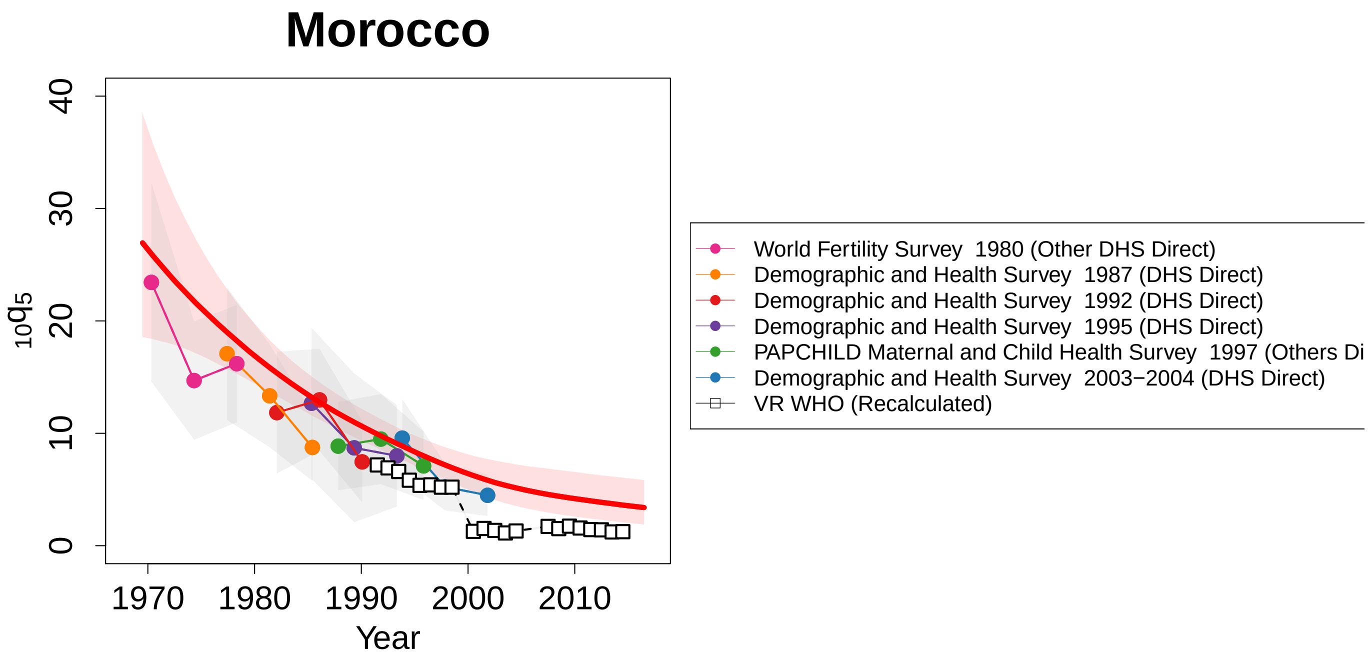
Morocco
40
30
20
10
0
$_{10}q_5$
1970
1980
1990
2000
2010
Year
World Fertility Survey 1980 (Other DHS Direct)
Demographic and Health Survey 1987 (DHS Direct)
Demographic and Health Survey 1992 (DHS Direct)
Demographic and Health Survey 1995 (DHS Direct)
PAPCHILD Maternal and Child Health Survey 1997 (Others Di
Demographic and Health Survey 2003−2004 (DHS Direct)
VR WHO (Recalculated)

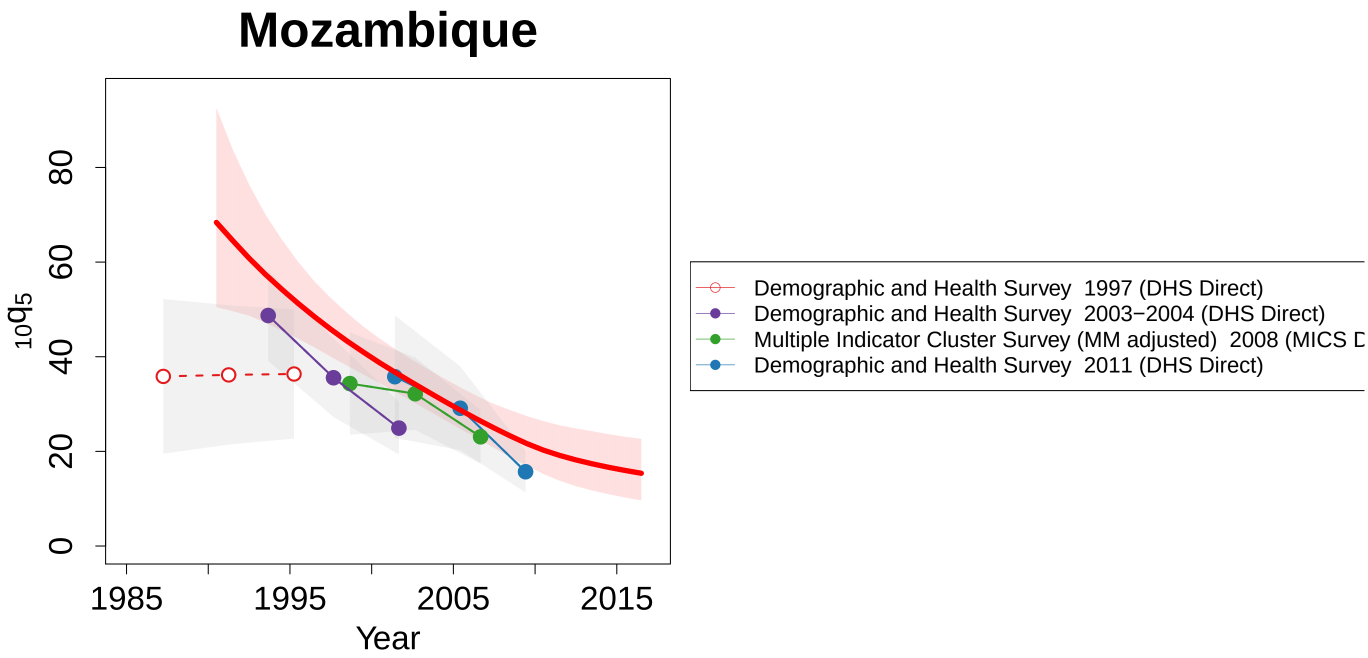
Mozambique
80
60
40
20
0
$_{10}q_5$
1985
1995
2005
2015
Year
Demographic and Health Survey 1997 (DHS Direct)
Demographic and Health Survey 2003−2004 (DHS Direct)
Multiple Indicator Cluster Survey (MM adjusted) 2008 (MICS D
Demographic and Health Survey 2011 (DHS Direct)

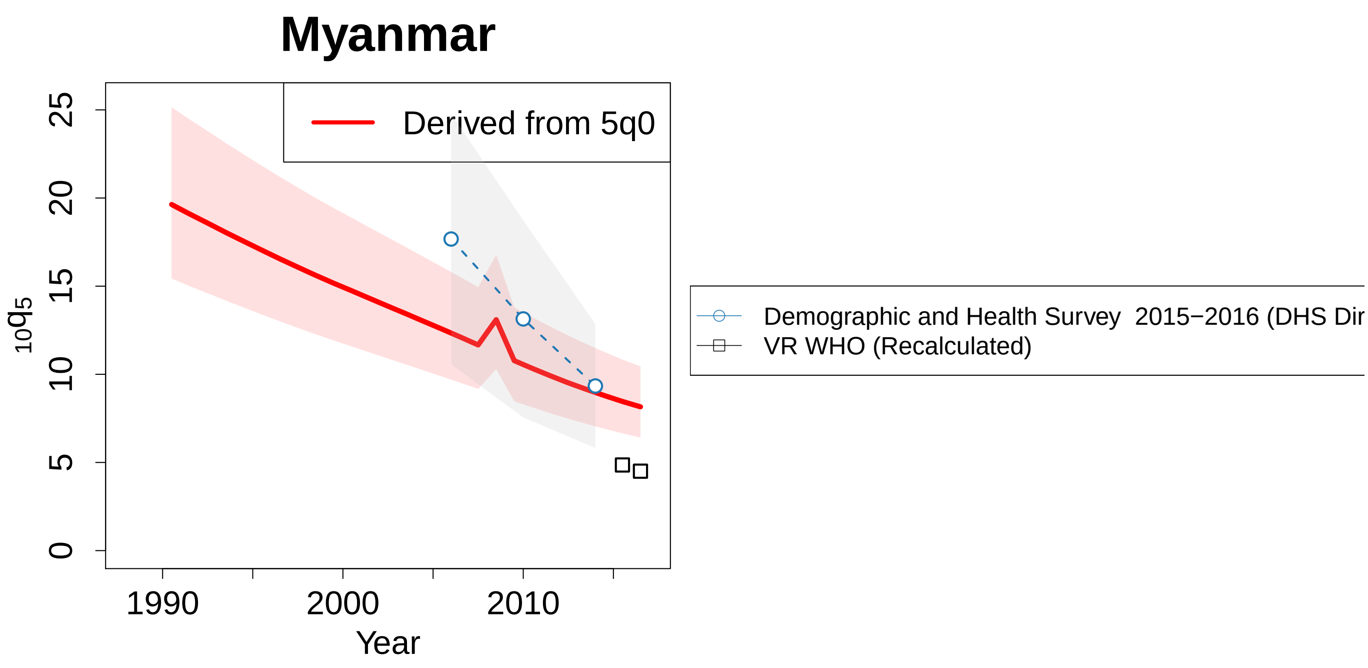
Myanmar
Derived from 5q0
Demographic and Health Survey 2015−2016 (DHS Dir
VR WHO (Recalculated)
$_{10}q_5$
0
5
10
15
20
25
1990
2000
2010
Year

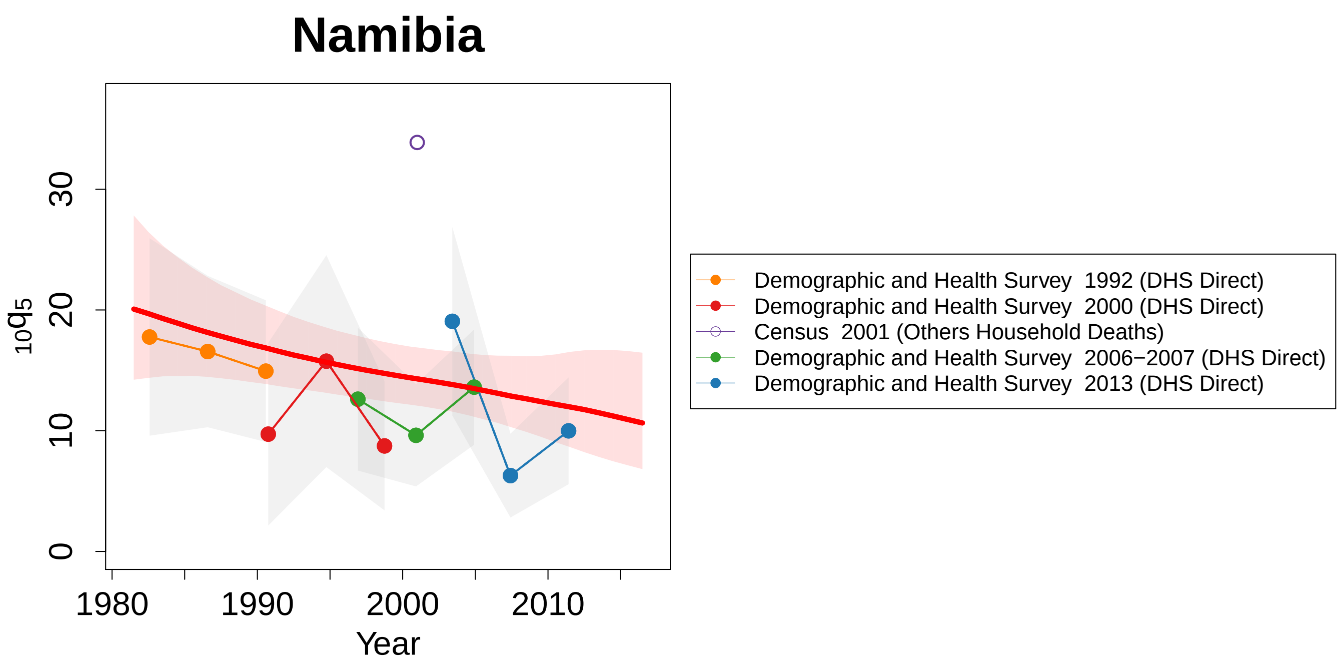
Namibia
Demographic and Health Survey 1992 (DHS Direct)
Demographic and Health Survey 2000 (DHS Direct)
Census 2001 (Others Household Deaths)
Demographic and Health Survey 2006−2007 (DHS Direct)
Demographic and Health Survey 2013 (DHS Direct)
$_{10}q_5$
0
10
20
30
1980
1990
2000
2010
Year

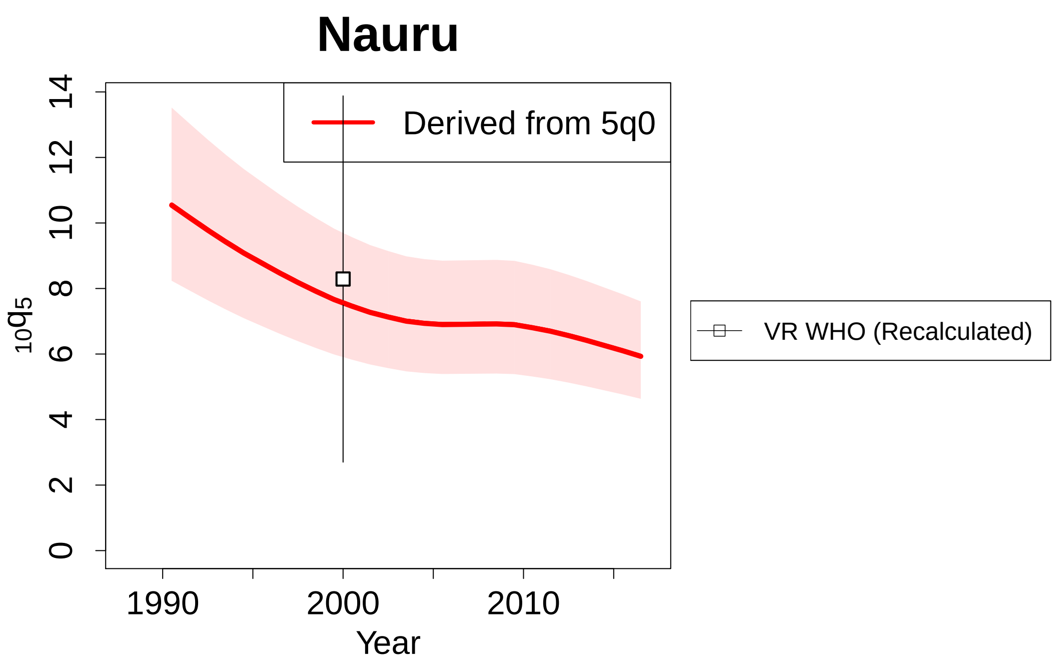
Nauru
Derived from 5q0
VR WHO (Recalculated)
$_{10}q_5$
0
2
4
6
8
10
12
14
1990
2000
2010
Year

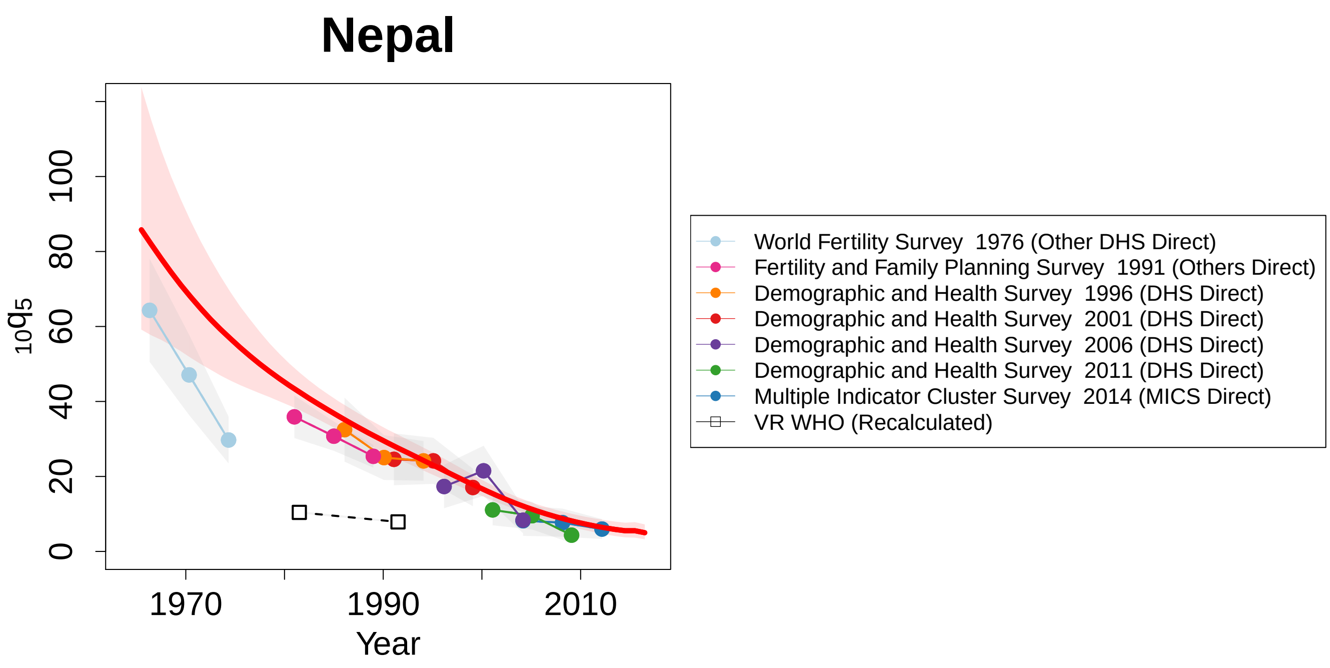
Nepal
World Fertility Survey 1976 (Other DHS Direct)
Fertility and Family Planning Survey 1991 (Others Direct)
Demographic and Health Survey 1996 (DHS Direct)
Demographic and Health Survey 2001 (DHS Direct)
Demographic and Health Survey 2006 (DHS Direct)
Demographic and Health Survey 2011 (DHS Direct)
Multiple Indicator Cluster Survey 2014 (MICS Direct)
VR WHO (Recalculated)
$_{10}q_5$
0
20
40
60
80
100
1970
1990
2010
Year

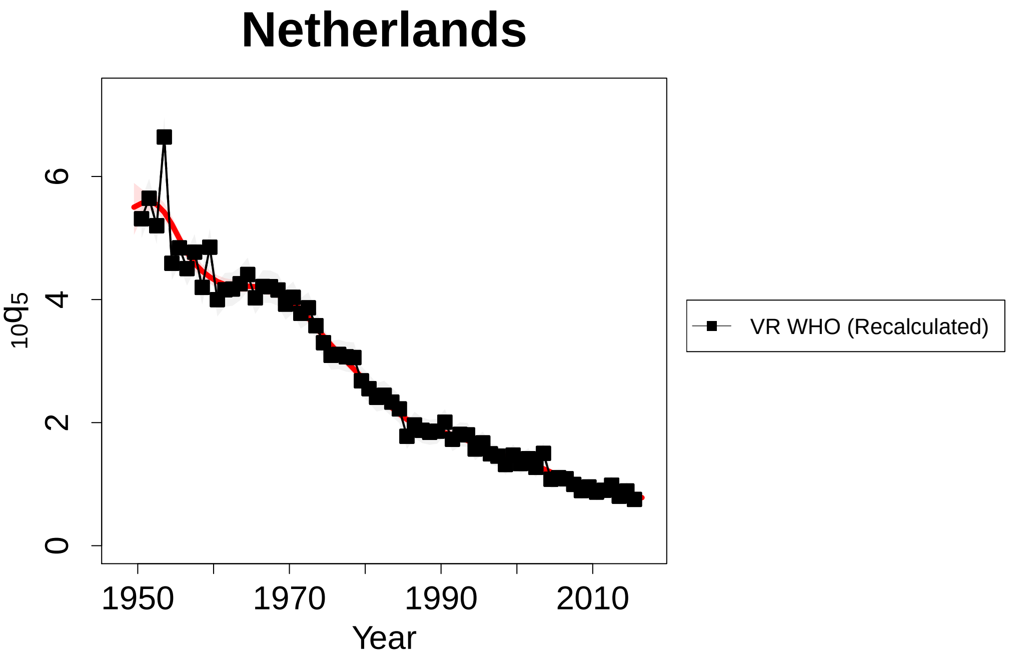
Netherlands
0
2
4
6
$_{10}q_5$
1950
1970
1990
2010
Year
VR WHO (Recalculated)

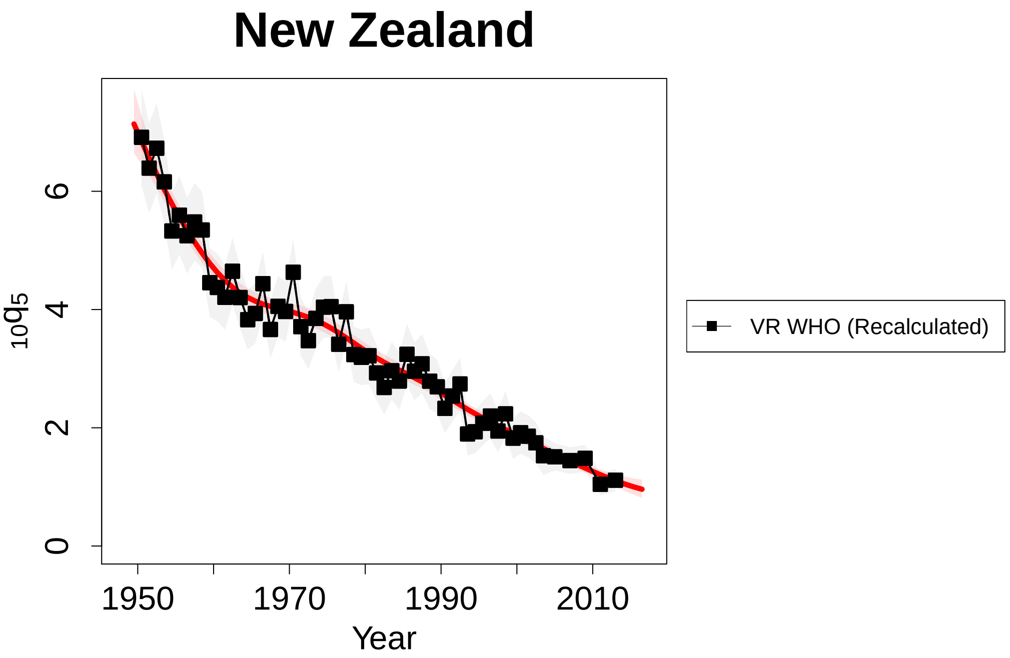
New Zealand
0
2
4
6
$_{10}q_5$
1950
1970
1990
2010
Year
VR WHO (Recalculated)

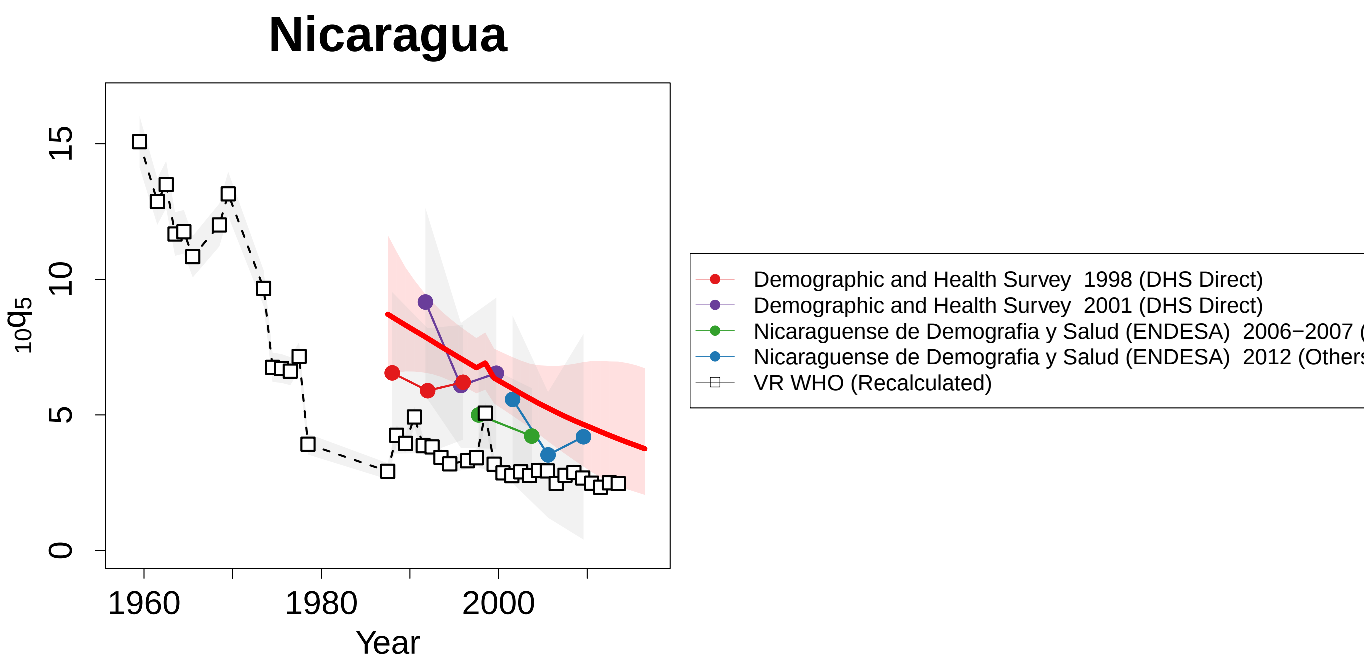
Nicaragua
15
10
5
0
$_{10}q_5$
1960
1980
2000
Year
Demographic and Health Survey 1998 (DHS Direct)
Demographic and Health Survey 2001 (DHS Direct)
Nicaraguense de Demografia y Salud (ENDESA) 2006−2007 (
Nicaraguense de Demografia y Salud (ENDESA) 2012 (Others
VR WHO (Recalculated)

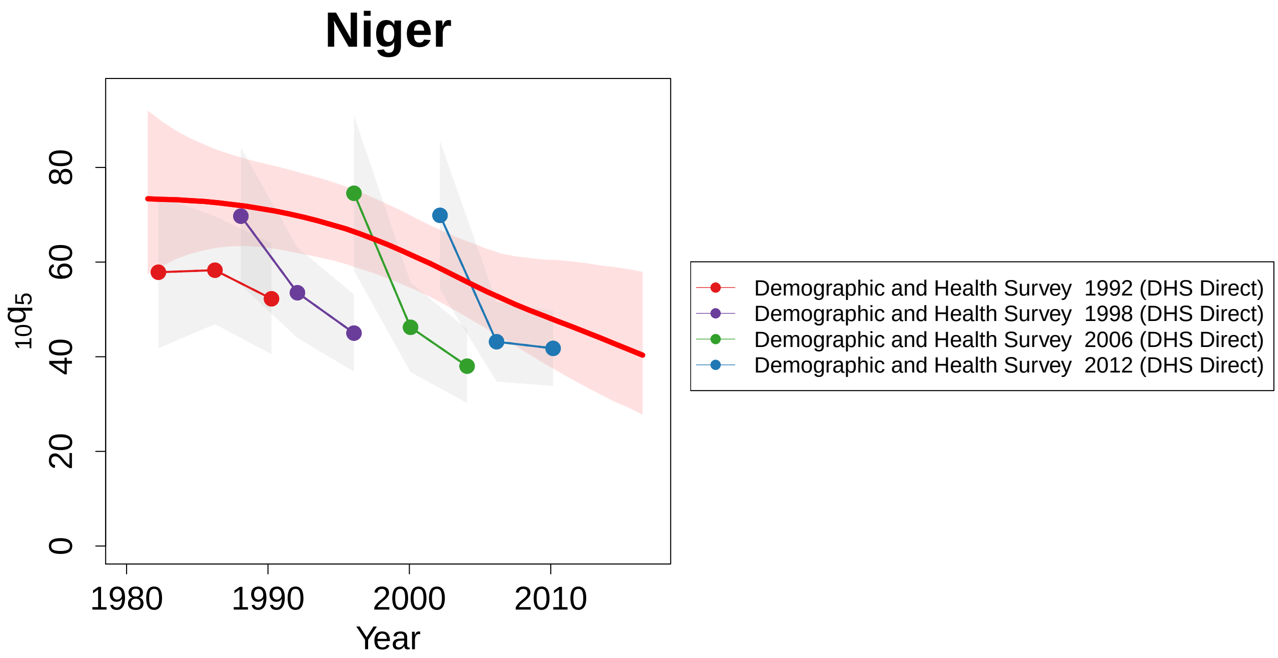
Niger
80
60
40
20
0
$_{10}q_5$
1980
1990
2000
2010
Year
Demographic and Health Survey 1992 (DHS Direct)
Demographic and Health Survey 1998 (DHS Direct)
Demographic and Health Survey 2006 (DHS Direct)
Demographic and Health Survey 2012 (DHS Direct)

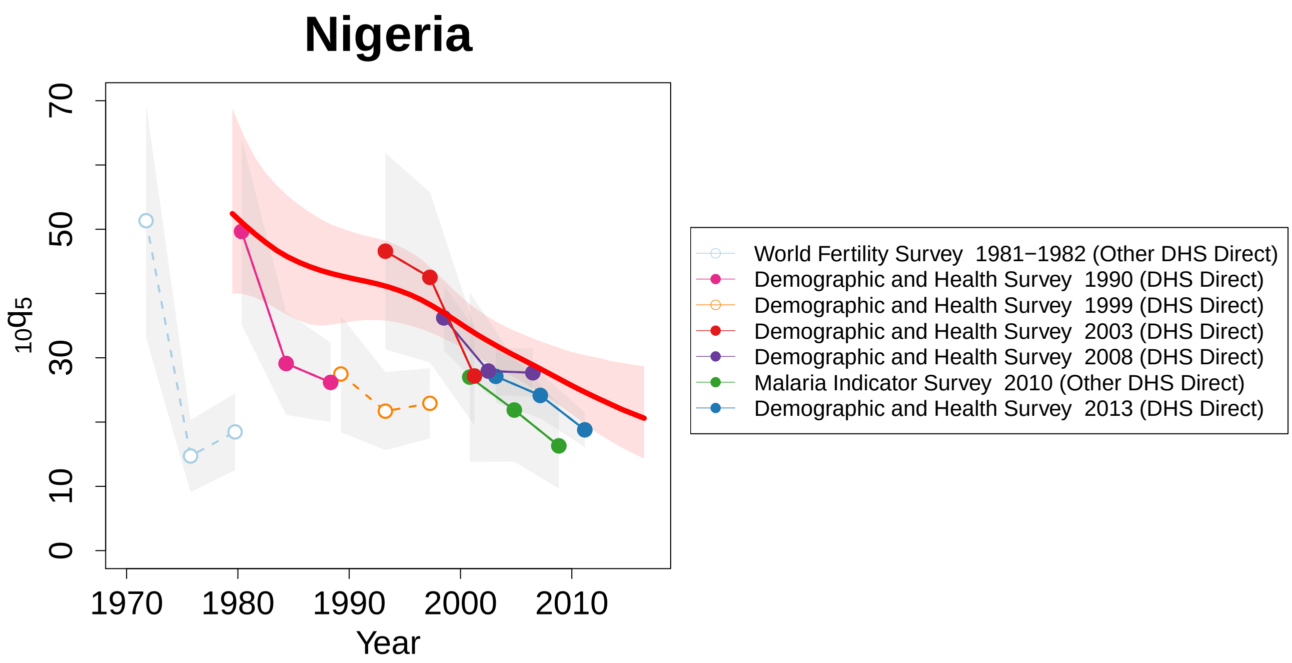
Nigeria
70
50
30
10
0
$_{10}q_5$
1970
1980
1990
2000
2010
Year
World Fertility Survey 1981−1982 (Other DHS Direct)
Demographic and Health Survey 1990 (DHS Direct)
Demographic and Health Survey 1999 (DHS Direct)
Demographic and Health Survey 2003 (DHS Direct)
Demographic and Health Survey 2008 (DHS Direct)
Malaria Indicator Survey 2010 (Other DHS Direct)
Demographic and Health Survey 2013 (DHS Direct)

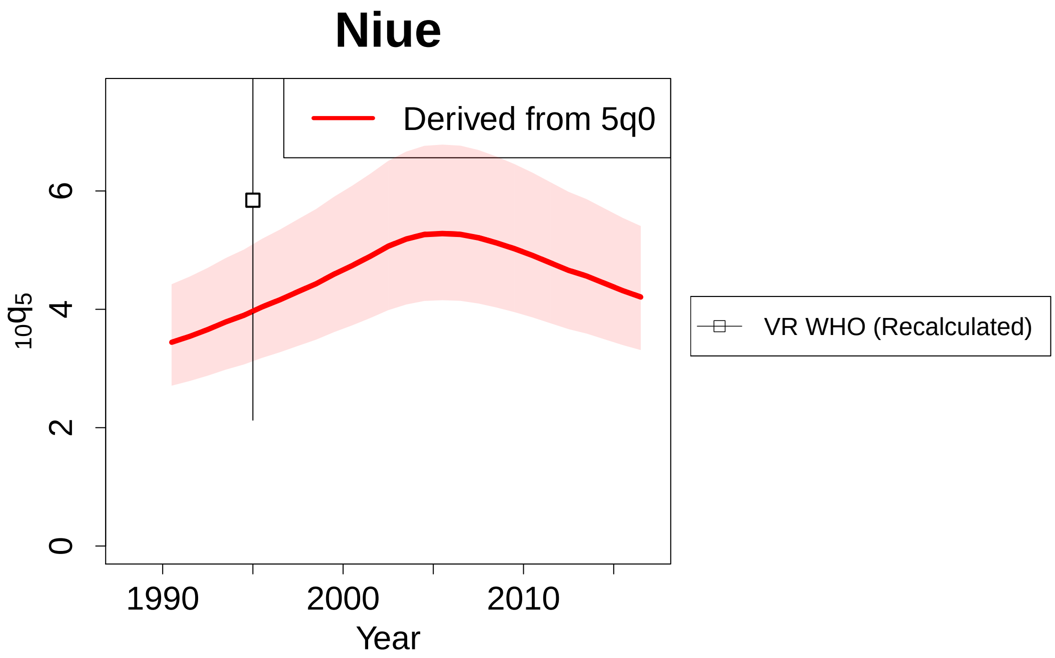
Niue
6
4
2
0
$_{10}q_5$
1990
2000
2010
Year
Derived from 5q0
VR WHO (Recalculated)

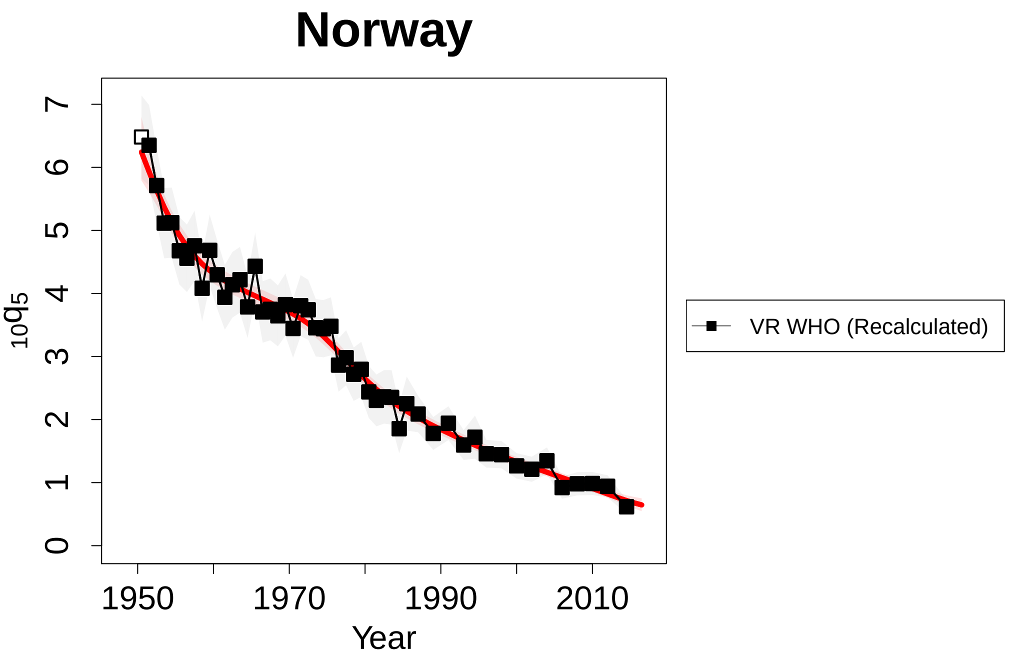
Norway
$_{10}q_5$
0
1
2
3
4
5
6
7
1950
1970
1990
2010
Year
VR WHO (Recalculated)

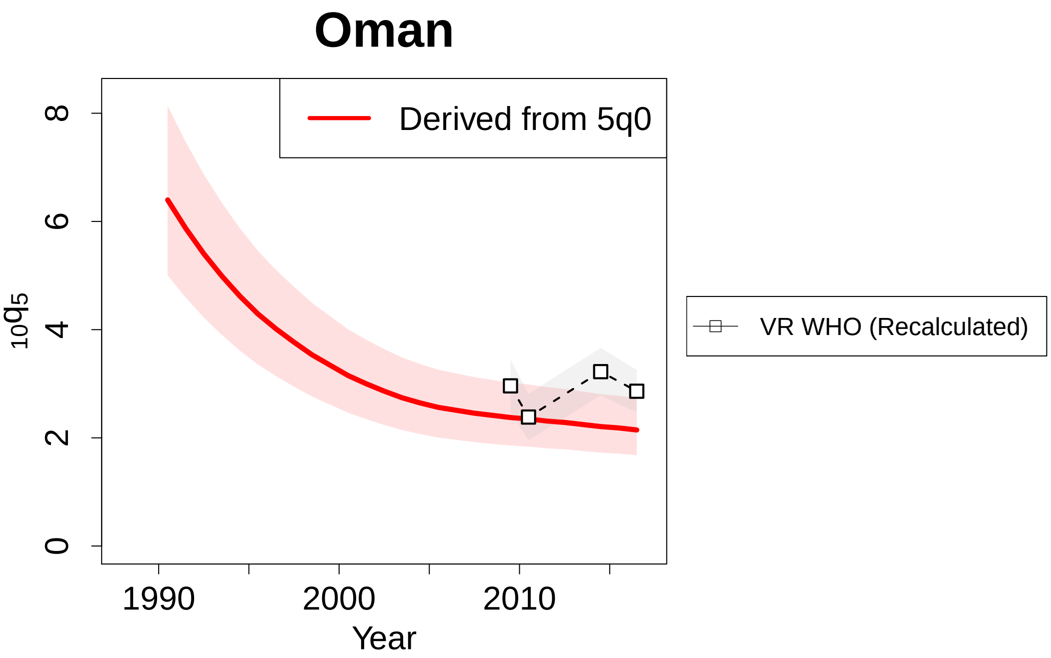
Oman
$_{10}q_5$
0
2
4
6
8
1990
2000
2010
Year
Derived from 5q0
VR WHO (Recalculated)

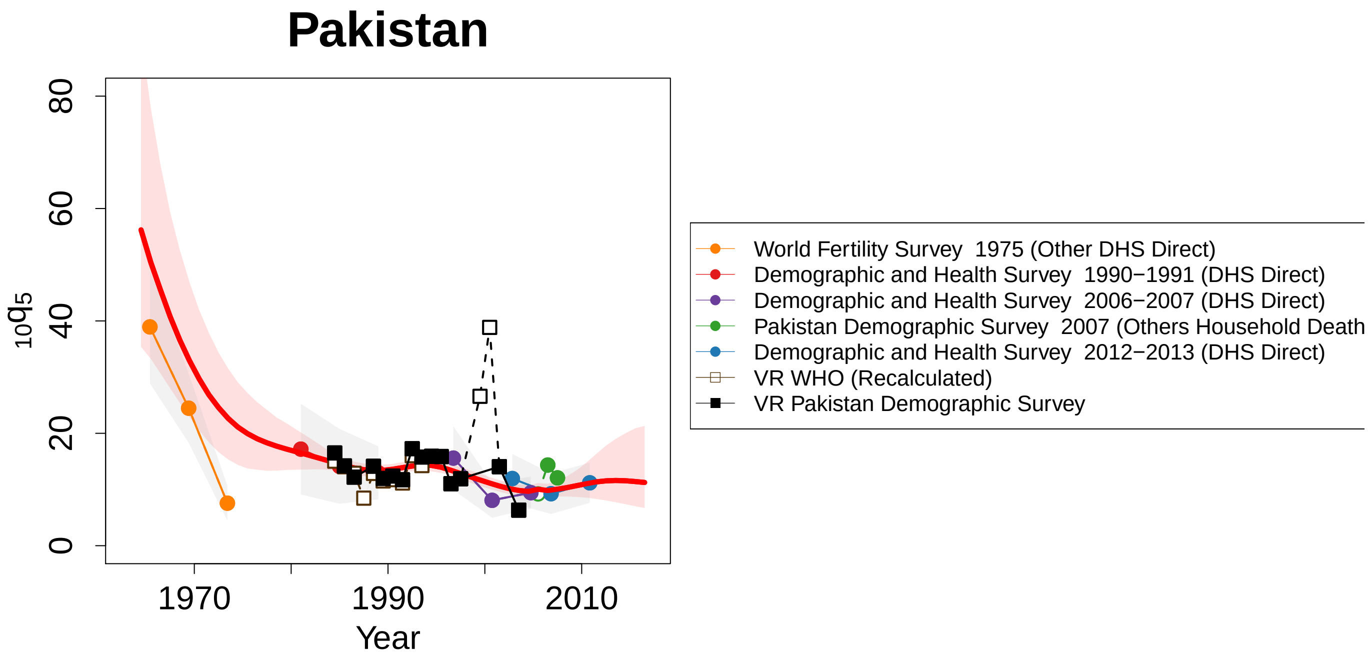
Pakistan
80
60
40
20
0
$_{10}q_5$
1970
1990
2010
Year
World Fertility Survey 1975 (Other DHS Direct)
Demographic and Health Survey 1990−1991 (DHS Direct)
Demographic and Health Survey 2006−2007 (DHS Direct)
Pakistan Demographic Survey 2007 (Others Household Death
Demographic and Health Survey 2012−2013 (DHS Direct)
VR WHO (Recalculated)
VR Pakistan Demographic Survey

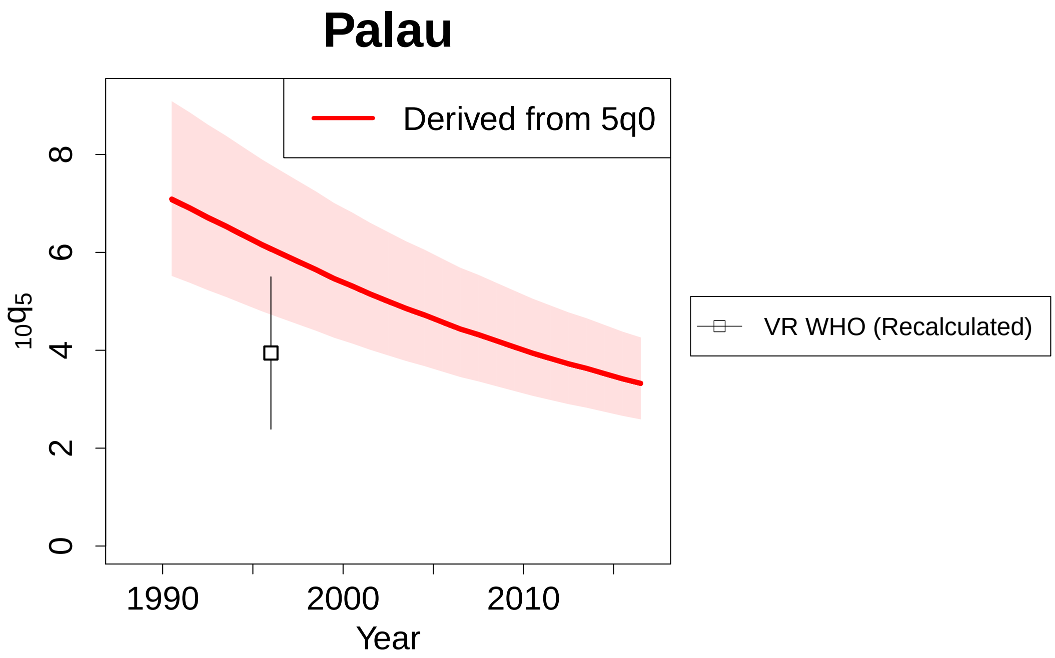
Palau
Derived from 5q0
8
6
4
2
0
$_{10}q_5$
1990
2000
2010
Year
VR WHO (Recalculated)

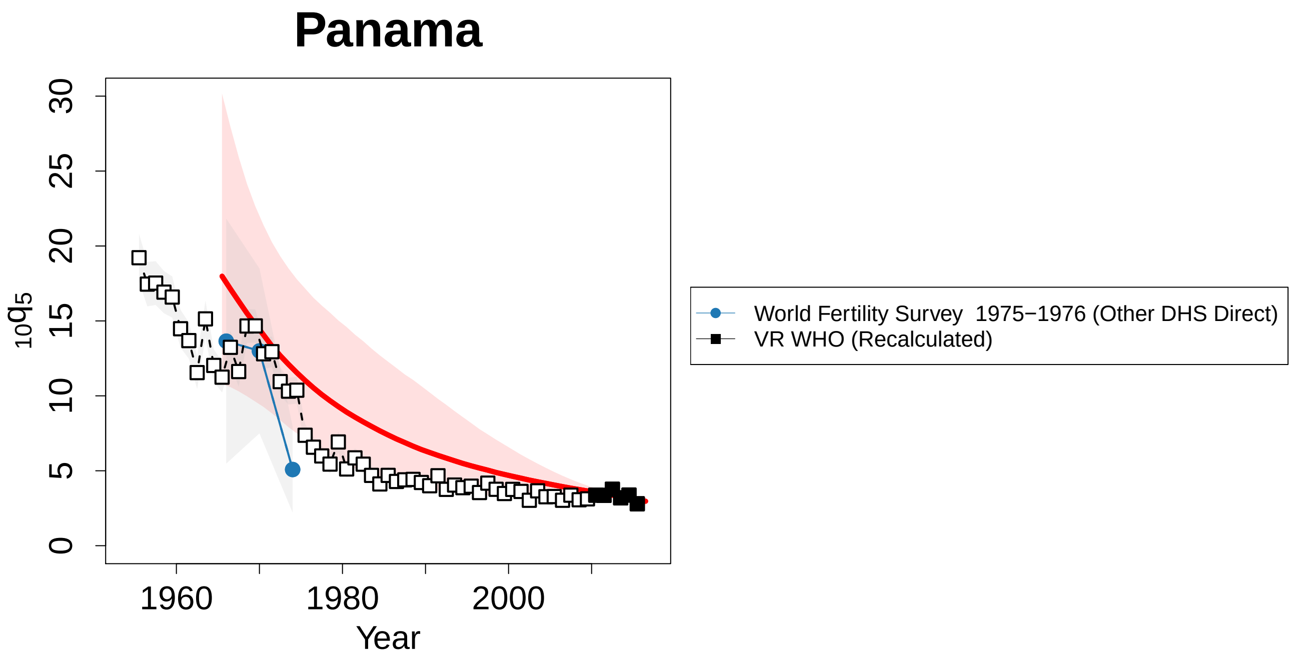
Panama
10q5
0
5
10
15
20
25
30
1960
1980
2000
Year
World Fertility Survey 1975−1976 (Other DHS Direct)
VR WHO (Recalculated)

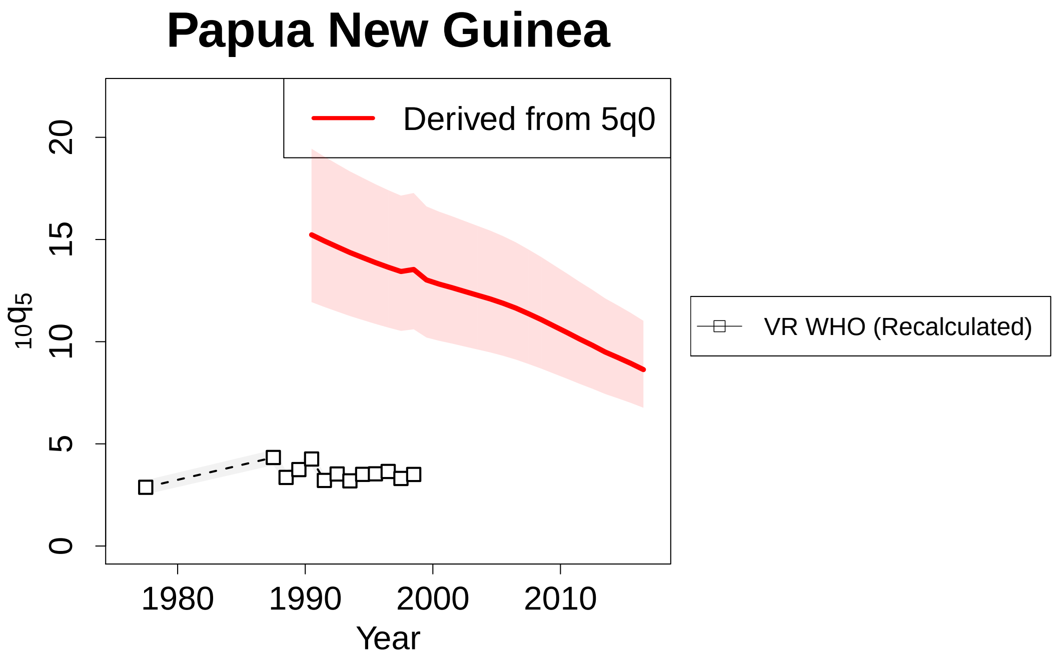
Papua New Guinea
Derived from 5q0
10q5
0
5
10
15
20
1980
1990
2000
2010
Year
VR WHO (Recalculated)

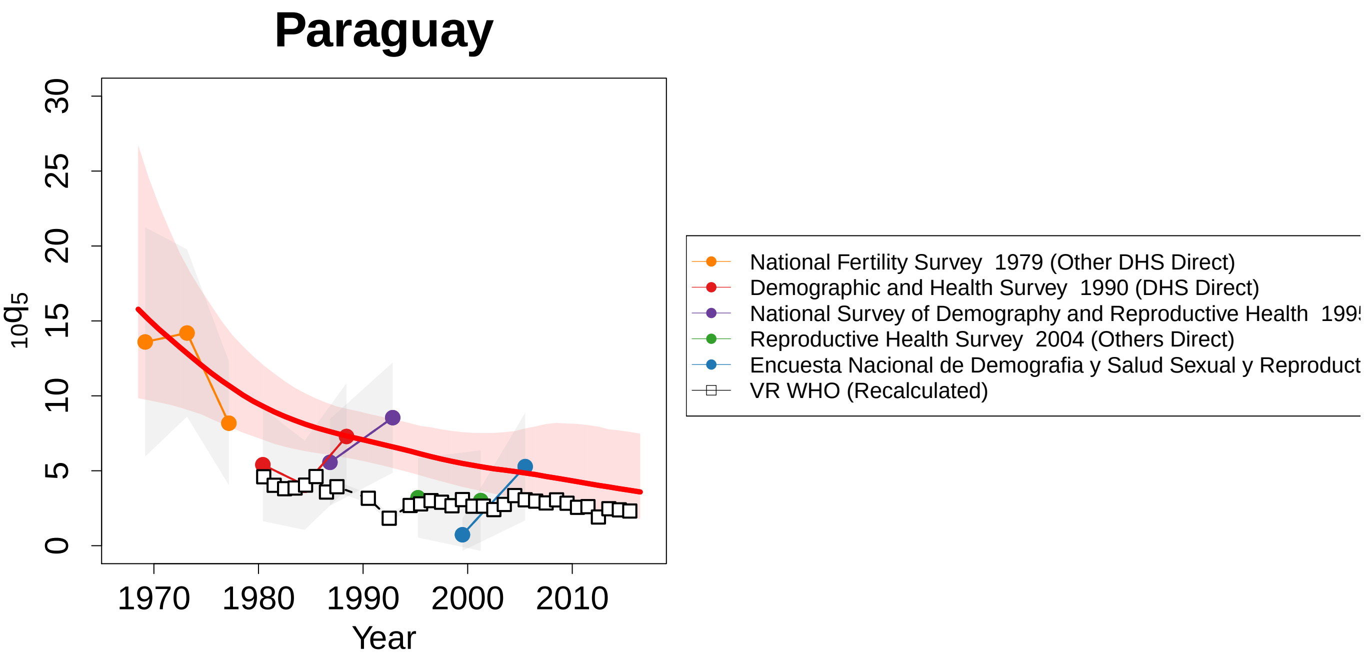
Paraguay
0
5
10
15
20
25
30
$_{10}q_5$
1970
1980
1990
2000
2010
Year
National Fertility Survey 1979 (Other DHS Direct)
Demographic and Health Survey 1990 (DHS Direct)
National Survey of Demography and Reproductive Health 199
Reproductive Health Survey 2004 (Others Direct)
Encuesta Nacional de Demografia y Salud Sexual y Reproduct
VR WHO (Recalculated)

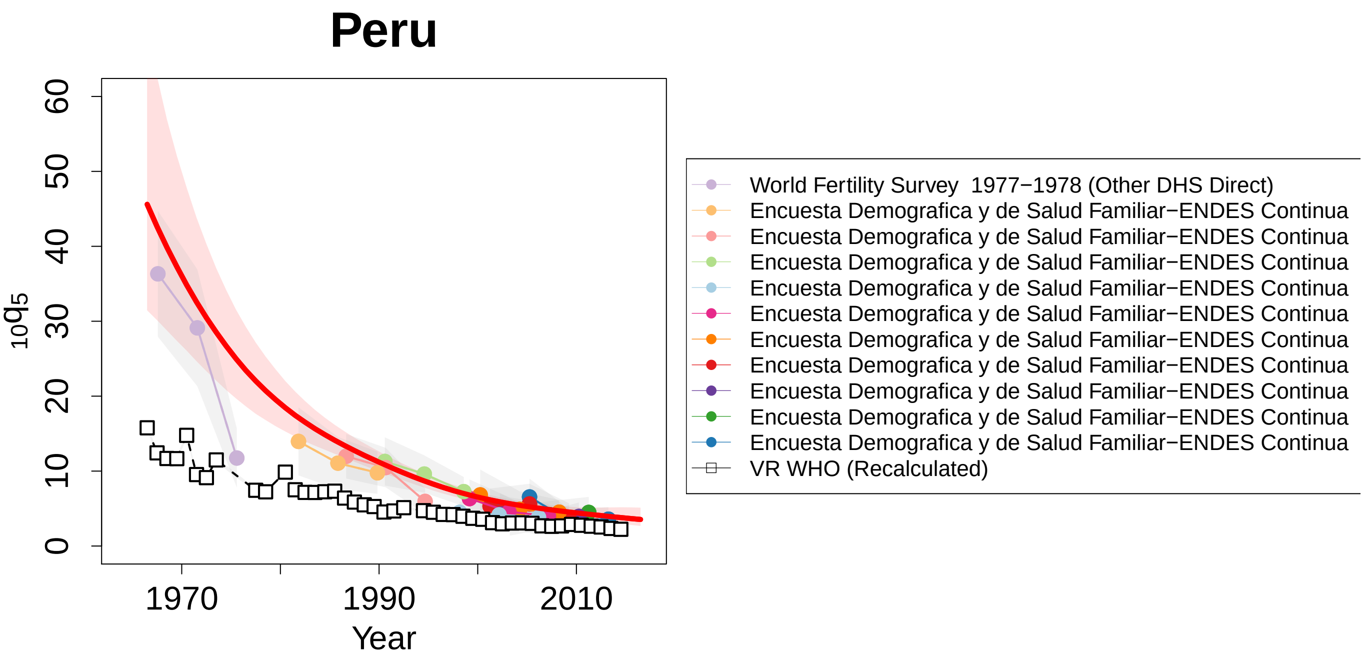
Peru
0
10
20
30
40
50
60
$_{10}q_5$
1970
1990
2010
Year
World Fertility Survey 1977−1978 (Other DHS Direct)
Encuesta Demografica y de Salud Familiar−ENDES Continua
Encuesta Demografica y de Salud Familiar−ENDES Continua
Encuesta Demografica y de Salud Familiar−ENDES Continua
Encuesta Demografica y de Salud Familiar−ENDES Continua
Encuesta Demografica y de Salud Familiar−ENDES Continua
Encuesta Demografica y de Salud Familiar−ENDES Continua
Encuesta Demografica y de Salud Familiar−ENDES Continua
Encuesta Demografica y de Salud Familiar−ENDES Continua
Encuesta Demografica y de Salud Familiar−ENDES Continua
Encuesta Demografica y de Salud Familiar−ENDES Continua
VR WHO (Recalculated)

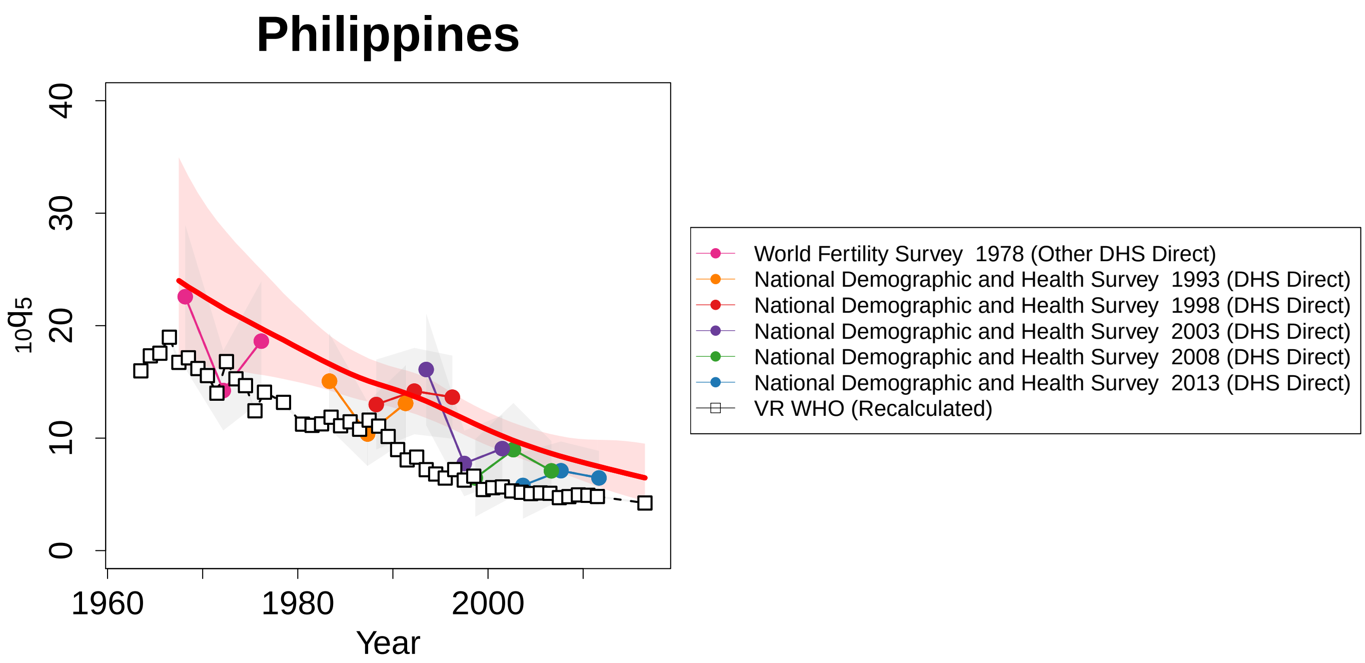
Philippines
Year
World Fertility Survey 1978 (Other DHS Direct)
National Demographic and Health Survey 1993 (DHS Direct)
National Demographic and Health Survey 1998 (DHS Direct)
National Demographic and Health Survey 2003 (DHS Direct)
National Demographic and Health Survey 2008 (DHS Direct)
National Demographic and Health Survey 2013 (DHS Direct)
VR WHO (Recalculated)

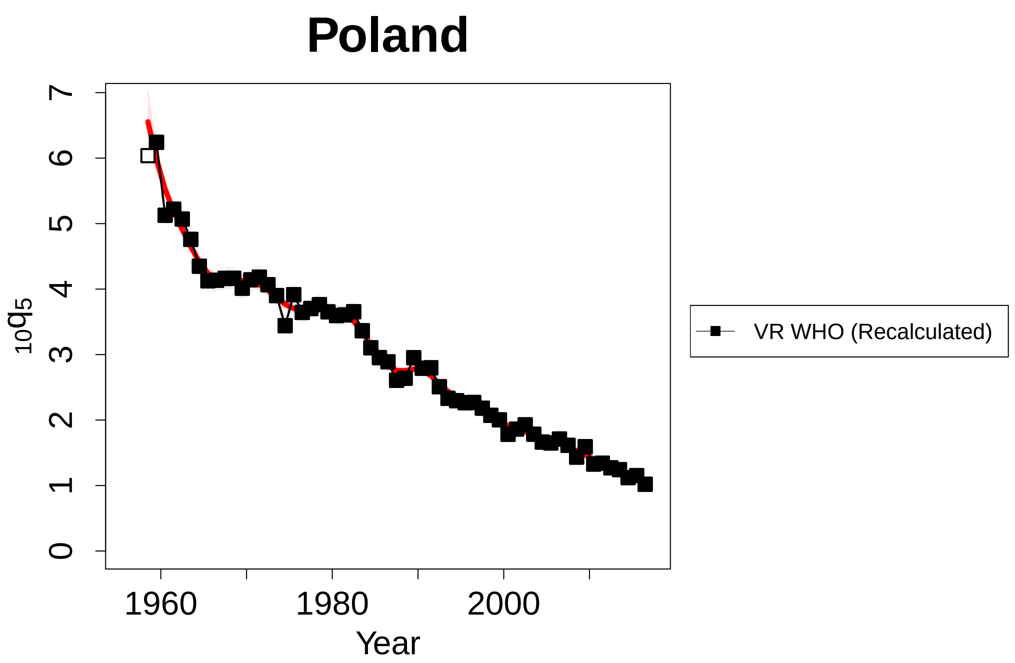
Poland
Year
VR WHO (Recalculated)

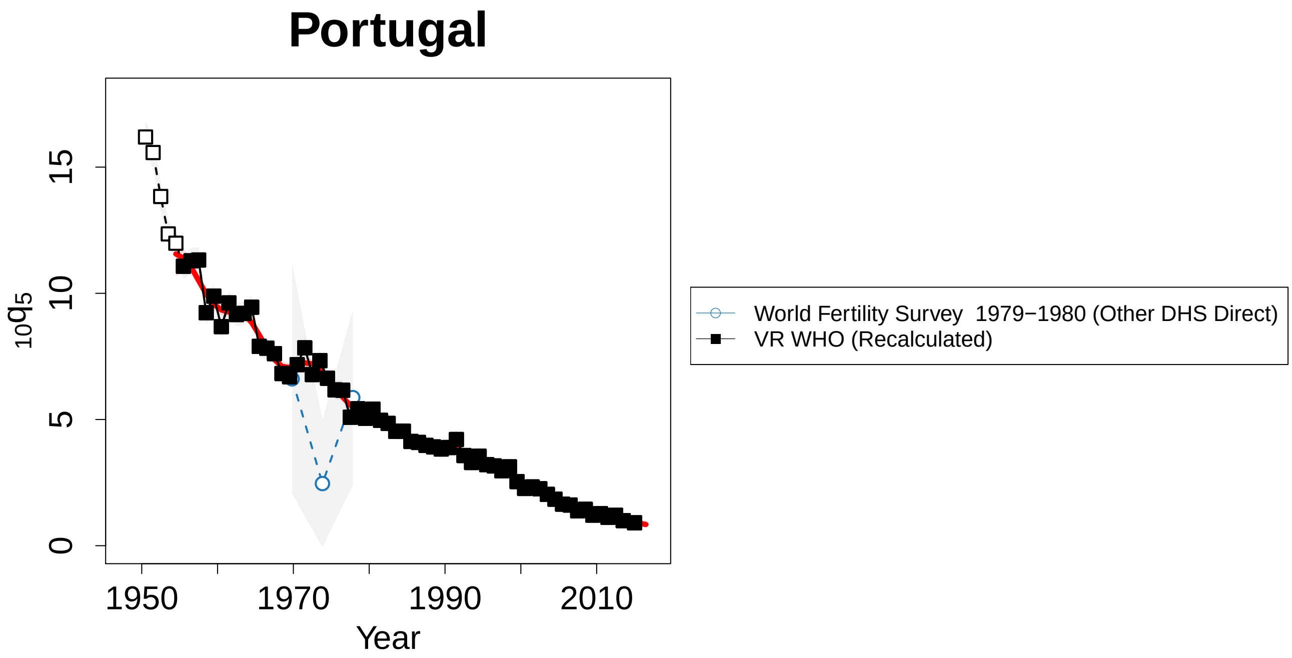
Portugal
$_{10}q_5$
0
5
10
15
1950
1970
1990
2010
Year
World Fertility Survey 1979−1980 (Other DHS Direct)
VR WHO (Recalculated)

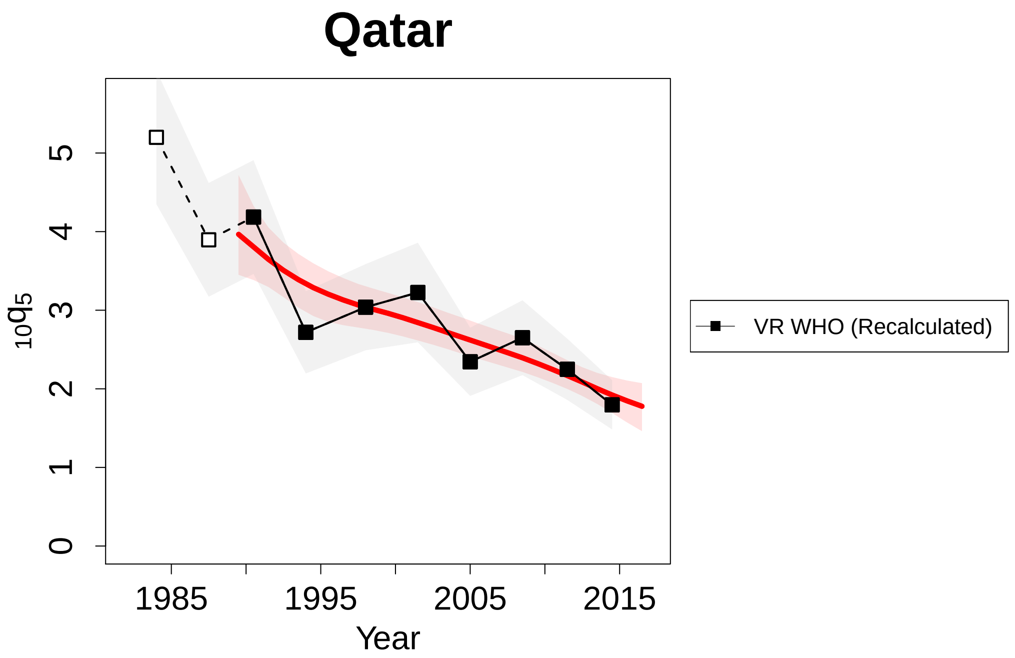
Qatar
$_{10}q_5$
0
1
2
3
4
5
1985
1995
2005
2015
Year
VR WHO (Recalculated)

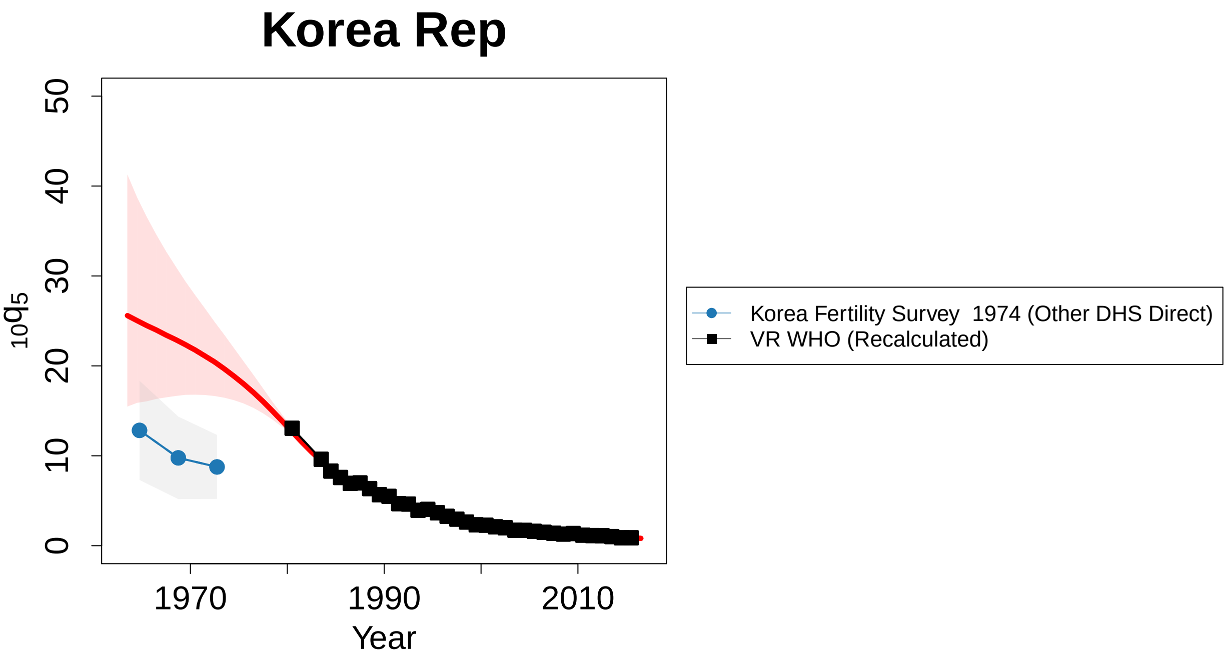
Korea Rep
50
40
30
20
10
0
$_{10}q_5$
1970
1990
2010
Year
Korea Fertility Survey 1974 (Other DHS Direct)
VR WHO (Recalculated)

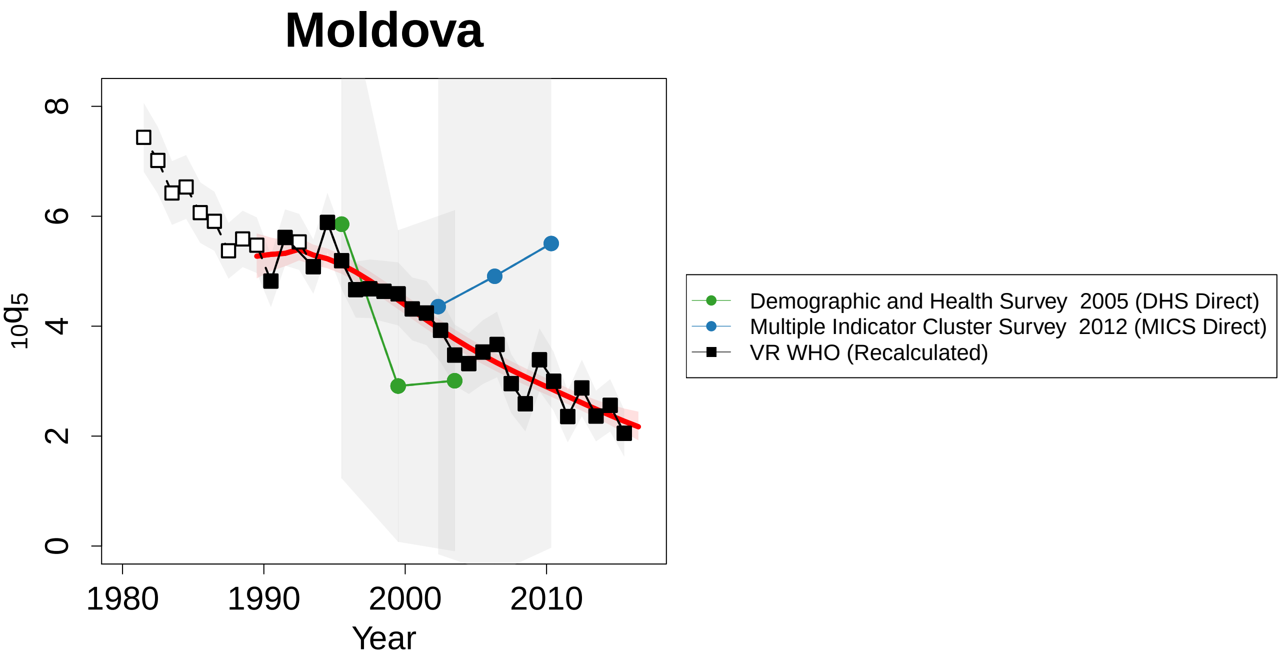
Moldova
8
6
4
2
0
$_{10}q_5$
1980
1990
2000
2010
Year
Demographic and Health Survey 2005 (DHS Direct)
Multiple Indicator Cluster Survey 2012 (MICS Direct)
VR WHO (Recalculated)

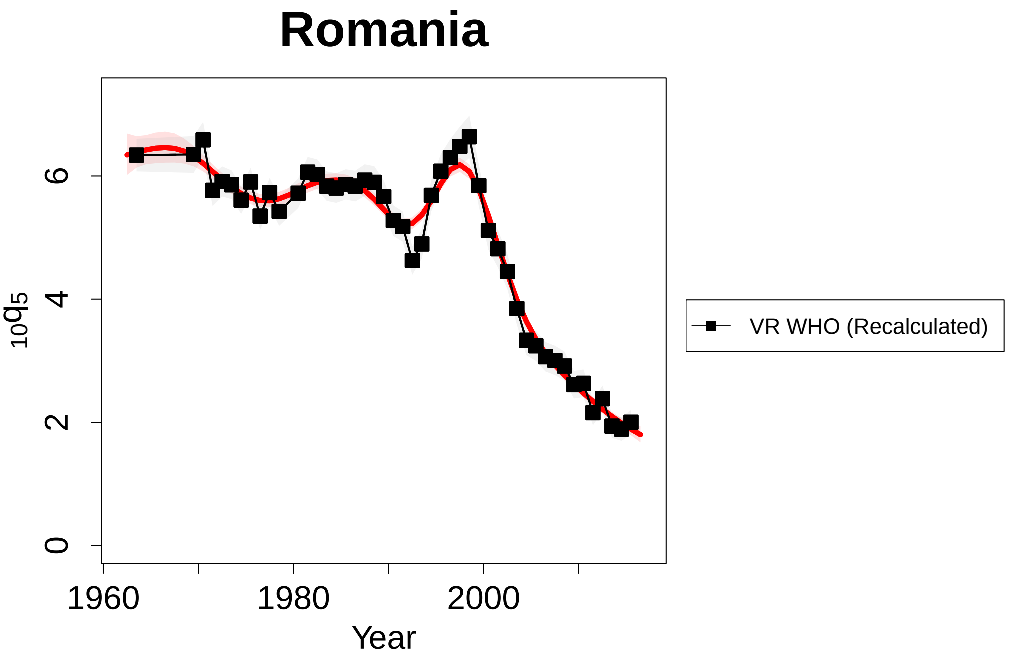
Romania
$_{10}q_5$
0
2
4
6
1960
1980
2000
Year
VR WHO (Recalculated)

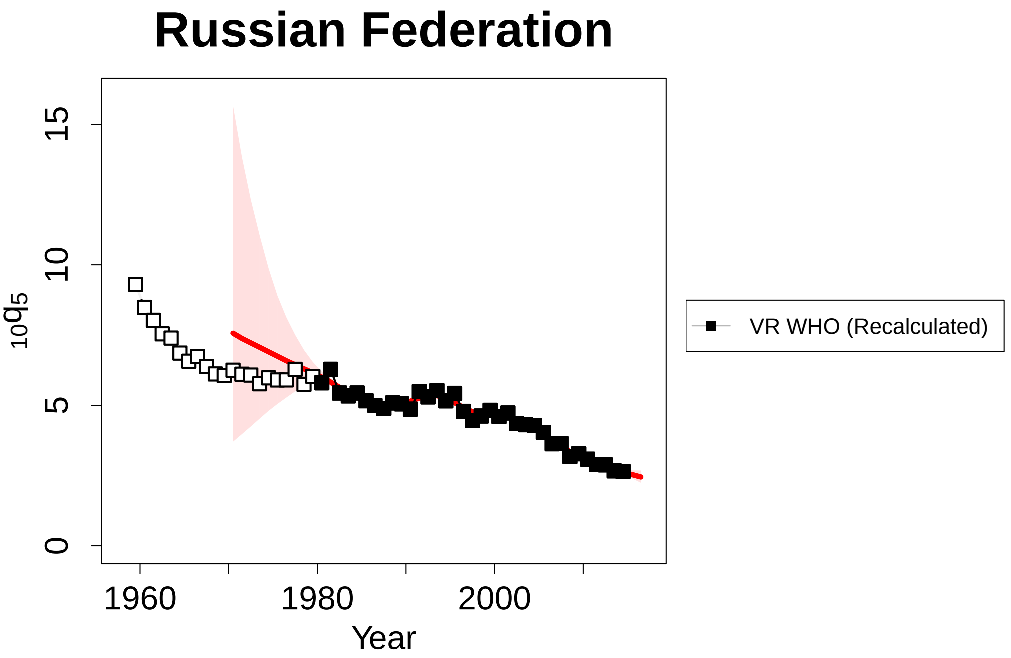
Russian Federation
$_{10}q_5$
0
5
10
15
1960
1980
2000
Year
VR WHO (Recalculated)

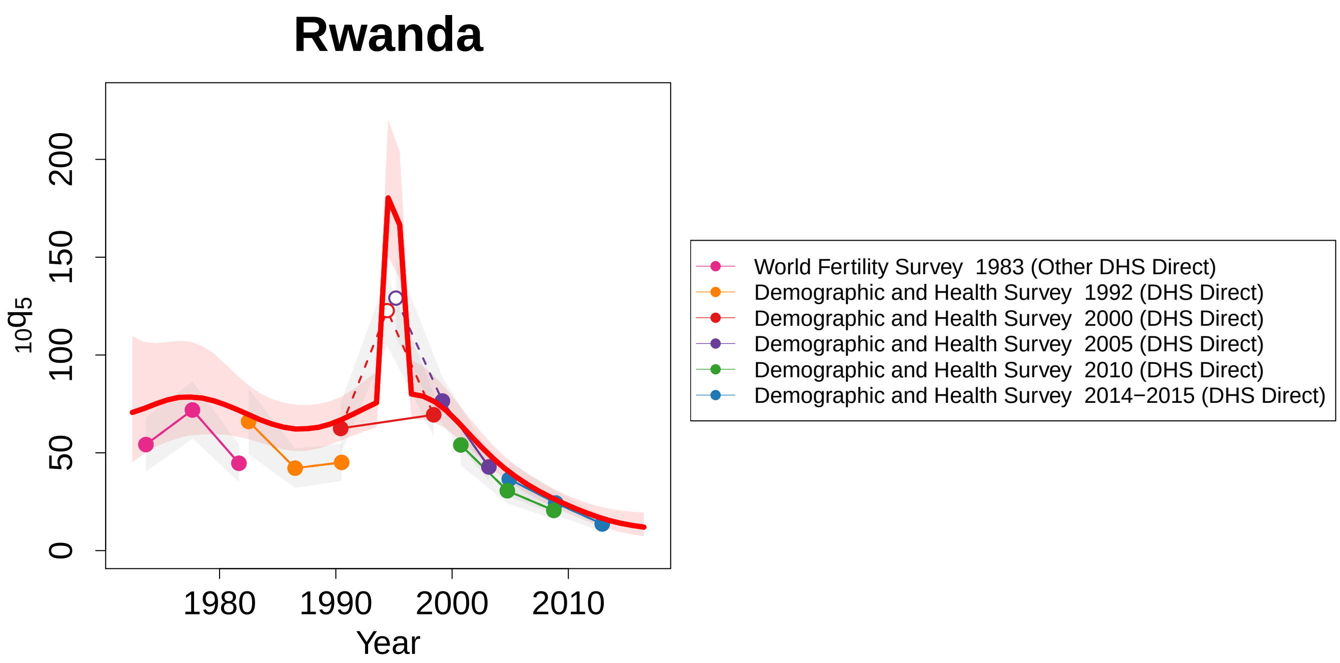
Rwanda
$_{10}q_5$
0
50
100
150
200
1980
1990
2000
2010
Year
World Fertility Survey 1983 (Other DHS Direct)
Demographic and Health Survey 1992 (DHS Direct)
Demographic and Health Survey 2000 (DHS Direct)
Demographic and Health Survey 2005 (DHS Direct)
Demographic and Health Survey 2010 (DHS Direct)
Demographic and Health Survey 2014−2015 (DHS Direct)

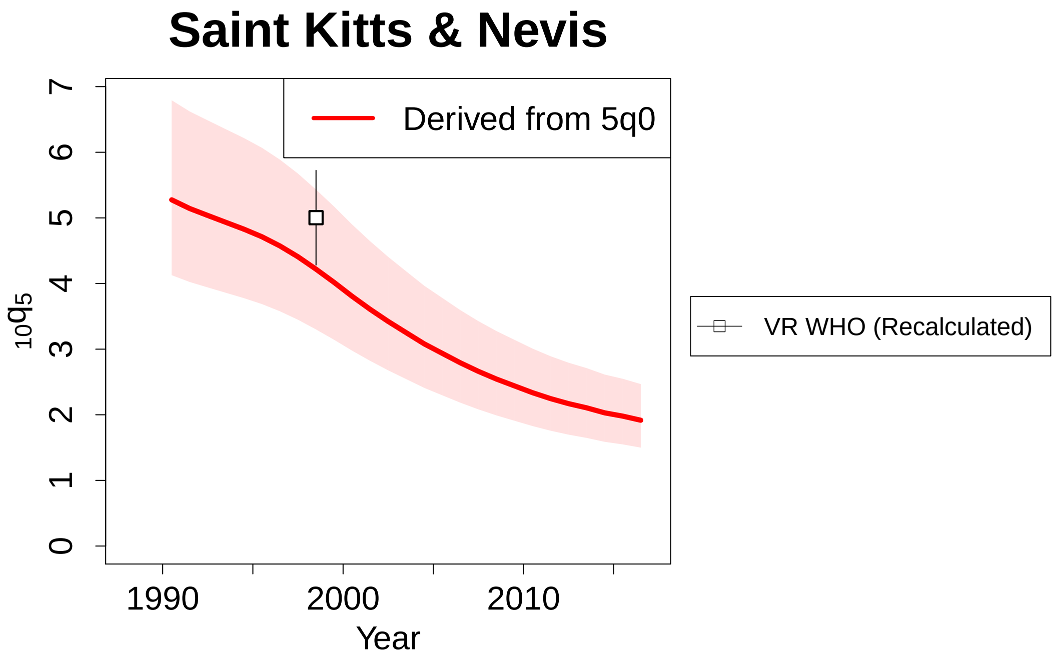
Saint Kitts & Nevis
$_{10}q_5$
0
1
2
3
4
5
6
7
1990
2000
2010
Year
Derived from 5q0
VR WHO (Recalculated)

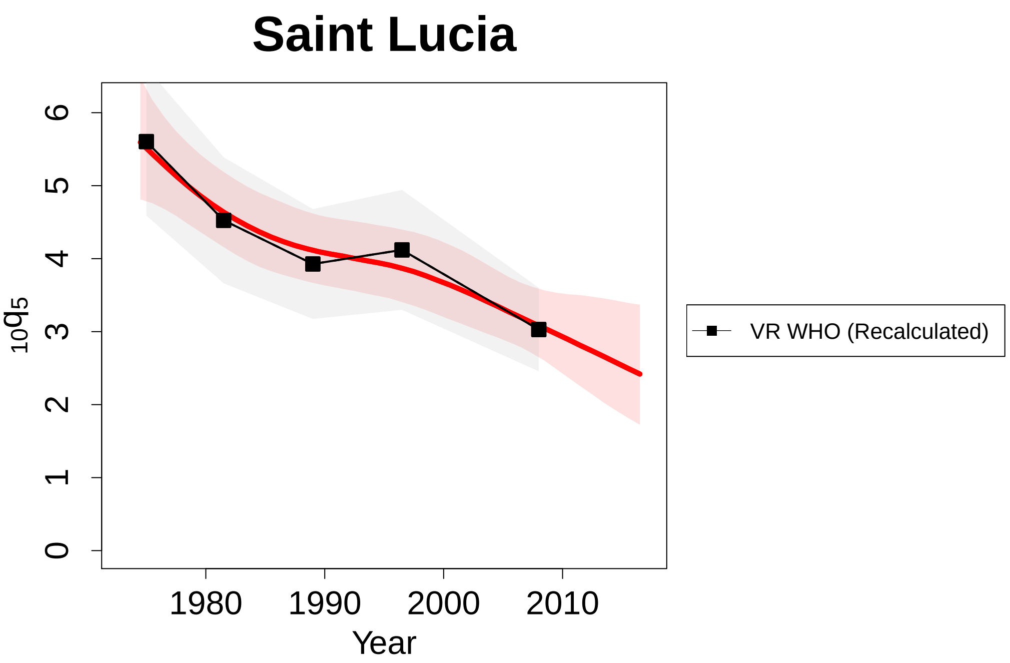
Saint Lucia
$_{10}q_5$
0
1
2
3
4
5
6
1980
1990
2000
2010
Year
VR WHO (Recalculated)

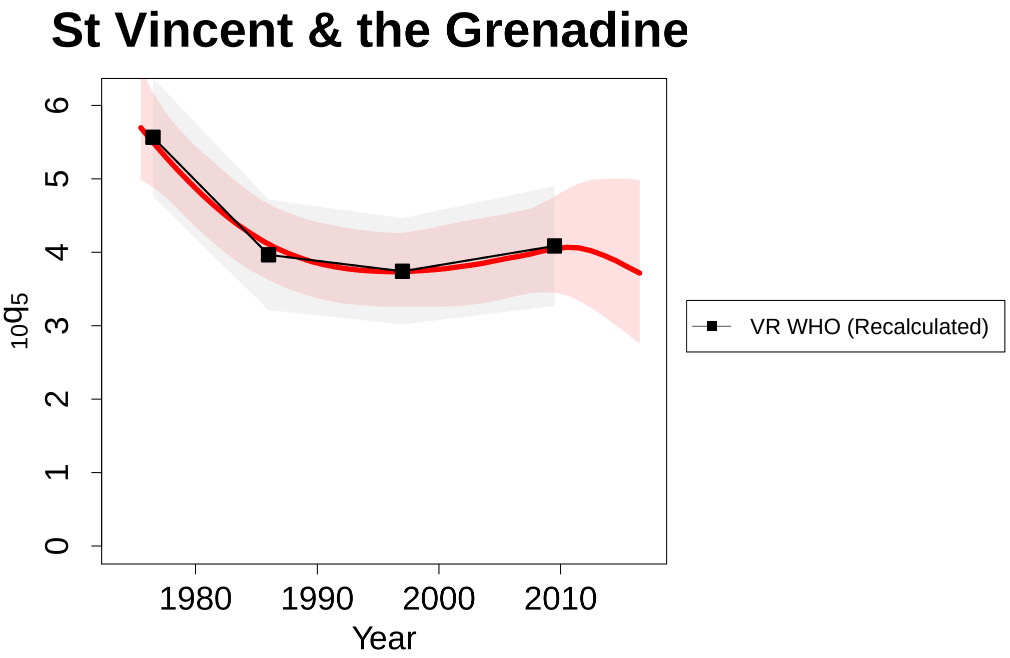
St Vincent & the Grenadine
$_{10}q_5$
0
1
2
3
4
5
6
1980
1990
2000
2010
Year
VR WHO (Recalculated)

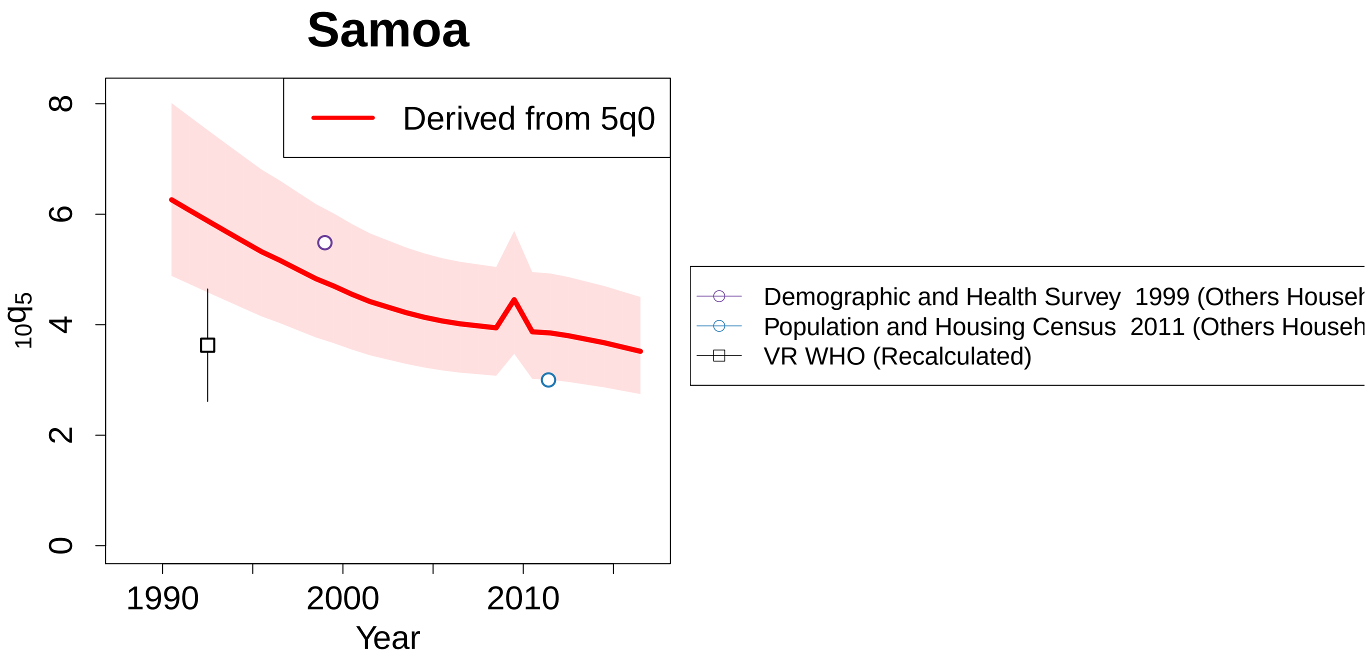
Samoa
Derived from 5q0
Demographic and Health Survey 1999 (Others Househ
Population and Housing Census 2011 (Others Househ
VR WHO (Recalculated)
10q5
0
2
4
6
8
1990
2000
2010
Year

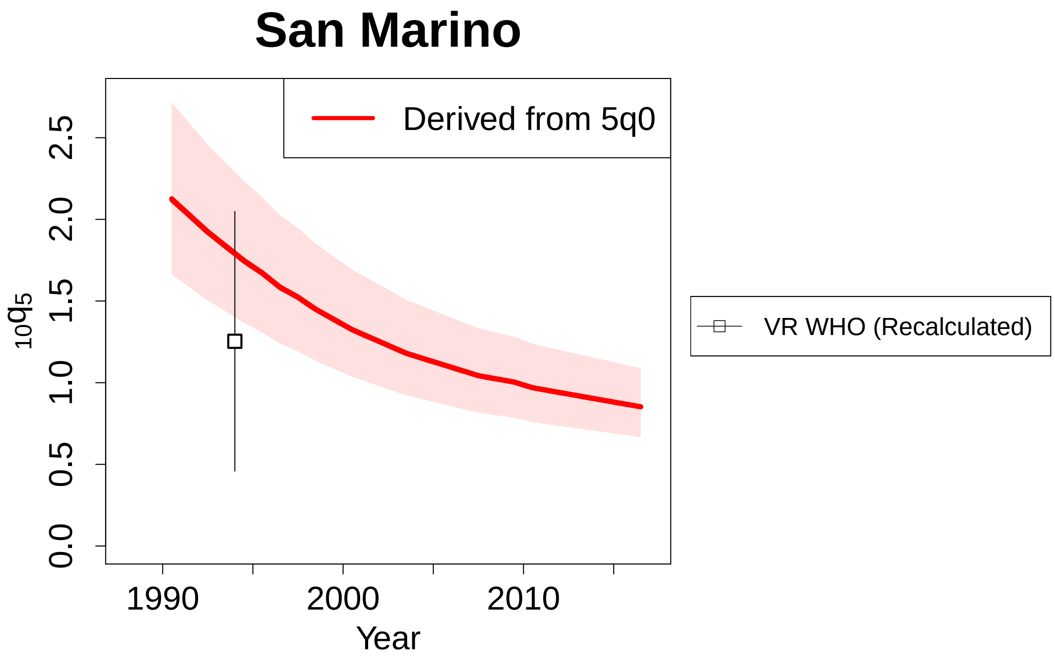
San Marino
Derived from 5q0
VR WHO (Recalculated)
10q5
0.0
0.5
1.0
1.5
2.0
2.5
1990
2000
2010
Year

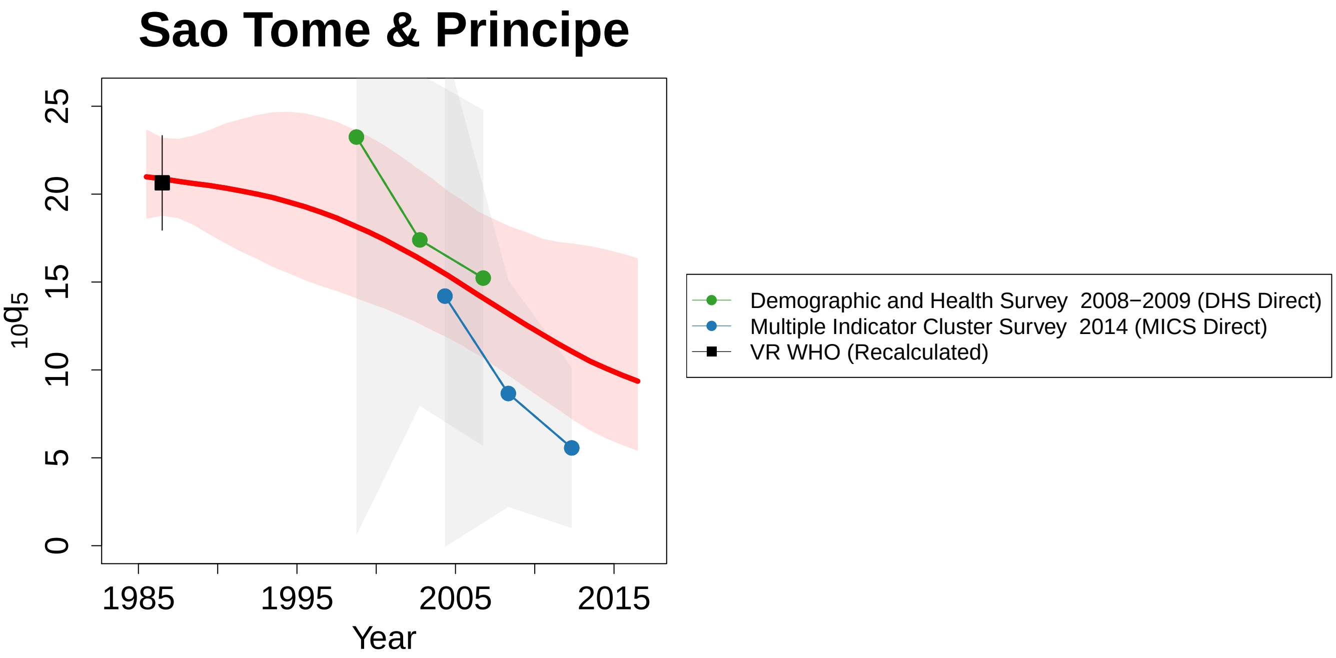
Sao Tome & Principe
0
5
10
15
20
25
$_{10}q_5$
1985
1995
2005
2015
Year
Demographic and Health Survey 2008−2009 (DHS Direct)
Multiple Indicator Cluster Survey 2014 (MICS Direct)
VR WHO (Recalculated)

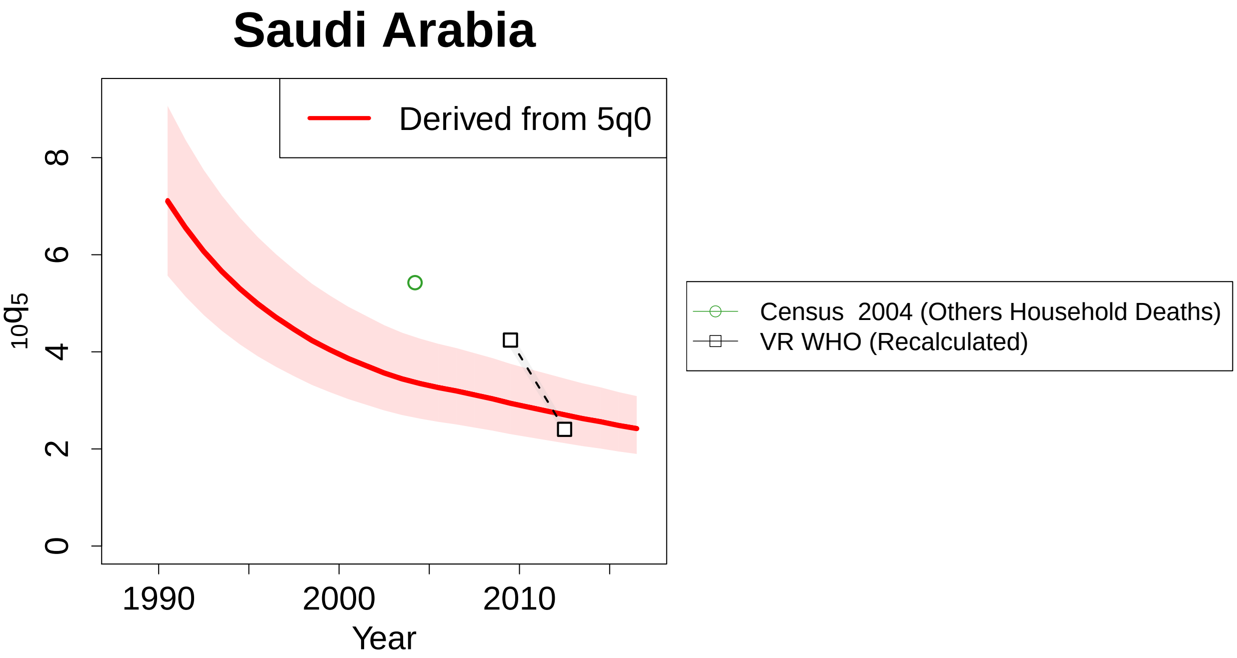
Saudi Arabia
Derived from 5q0
0
2
4
6
8
$_{10}q_5$
1990
2000
2010
Year
Census 2004 (Others Household Deaths)
VR WHO (Recalculated)

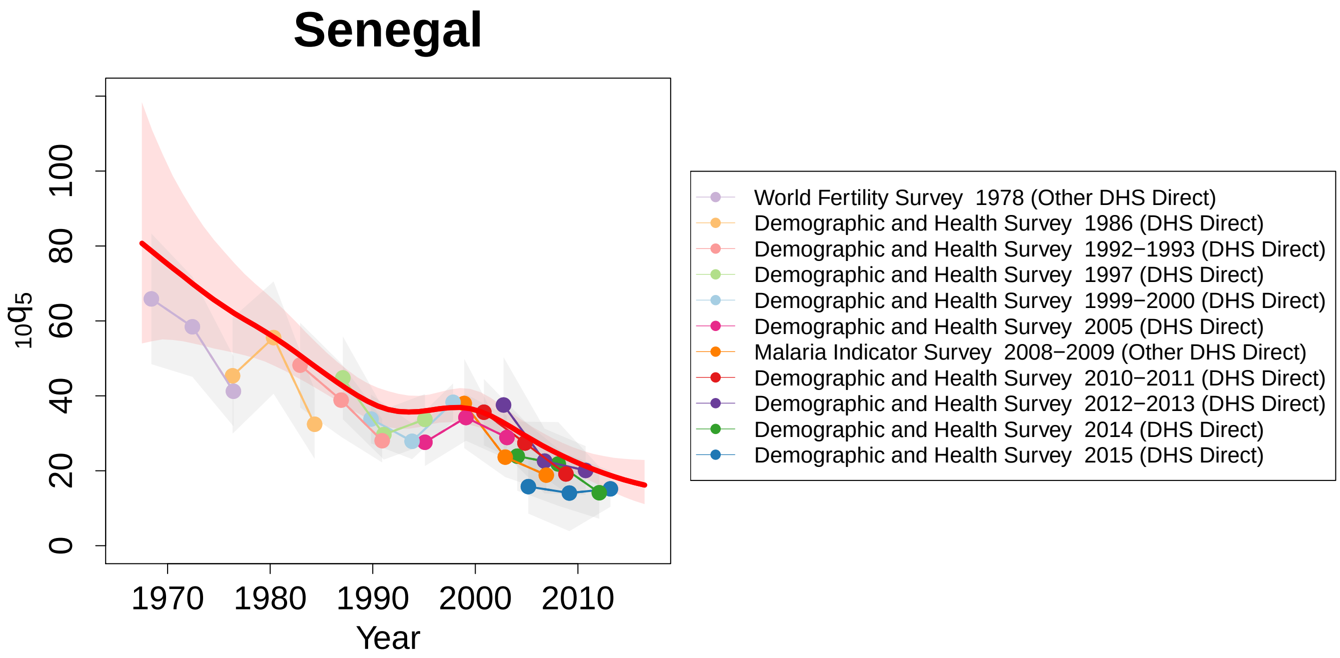
Senegal
$_{10}q_5$
0
20
40
60
80
100
1970
1980
1990
2000
2010
Year
World Fertility Survey 1978 (Other DHS Direct)
Demographic and Health Survey 1986 (DHS Direct)
Demographic and Health Survey 1992−1993 (DHS Direct)
Demographic and Health Survey 1997 (DHS Direct)
Demographic and Health Survey 1999−2000 (DHS Direct)
Demographic and Health Survey 2005 (DHS Direct)
Malaria Indicator Survey 2008−2009 (Other DHS Direct)
Demographic and Health Survey 2010−2011 (DHS Direct)
Demographic and Health Survey 2012−2013 (DHS Direct)
Demographic and Health Survey 2014 (DHS Direct)
Demographic and Health Survey 2015 (DHS Direct)

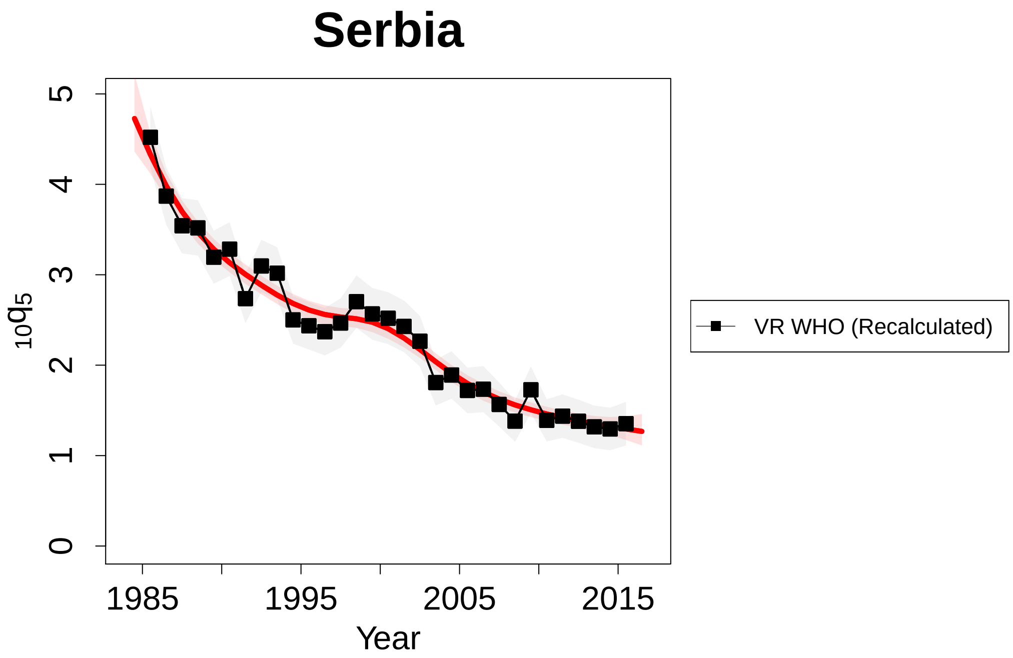
Serbia
$_{10}q_5$
0
1
2
3
4
5
1985
1995
2005
2015
Year
VR WHO (Recalculated)

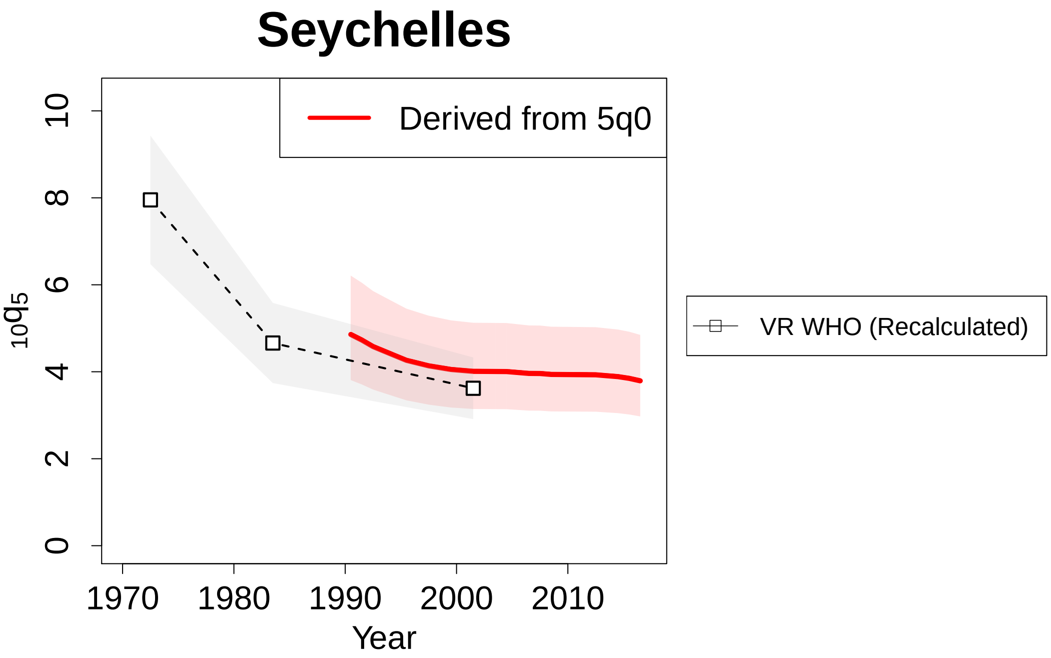

Seychelles
Derived from 5q0
VR WHO (Recalculated)
$_{10}q_5$
0
2
4
6
8
10
1970
1980
1990
2000
2010
Year

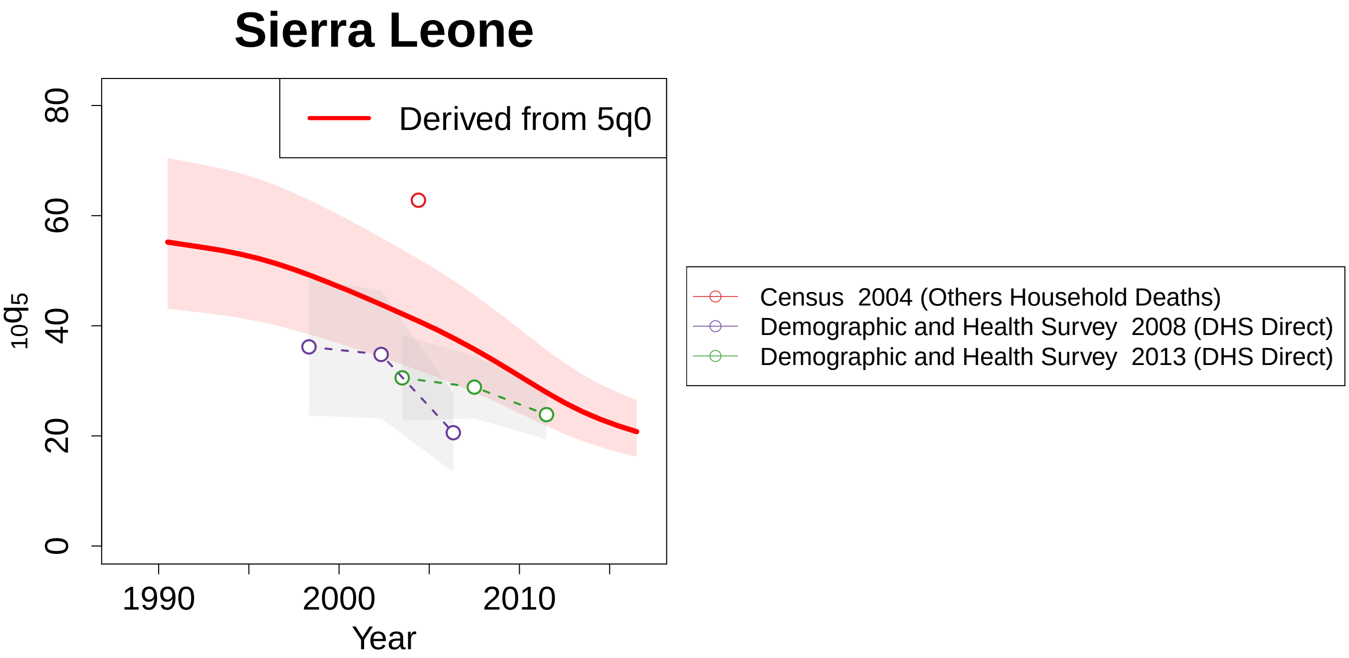

Sierra Leone
Derived from 5q0
Census 2004 (Others Household Deaths)
Demographic and Health Survey 2008 (DHS Direct)
Demographic and Health Survey 2013 (DHS Direct)
$_{10}q_5$
0
20
40
60
80
1990
2000
2010
Year

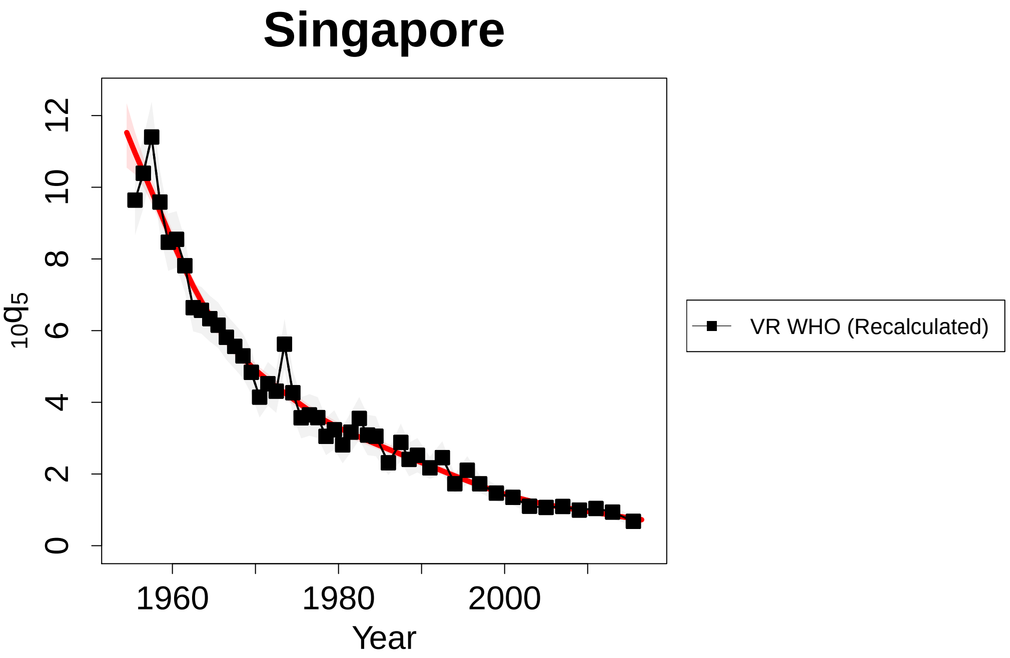
Singapore
12
10
8
6
4
2
0
$_{10}q_5$
1960
1980
2000
Year
VR WHO (Recalculated)

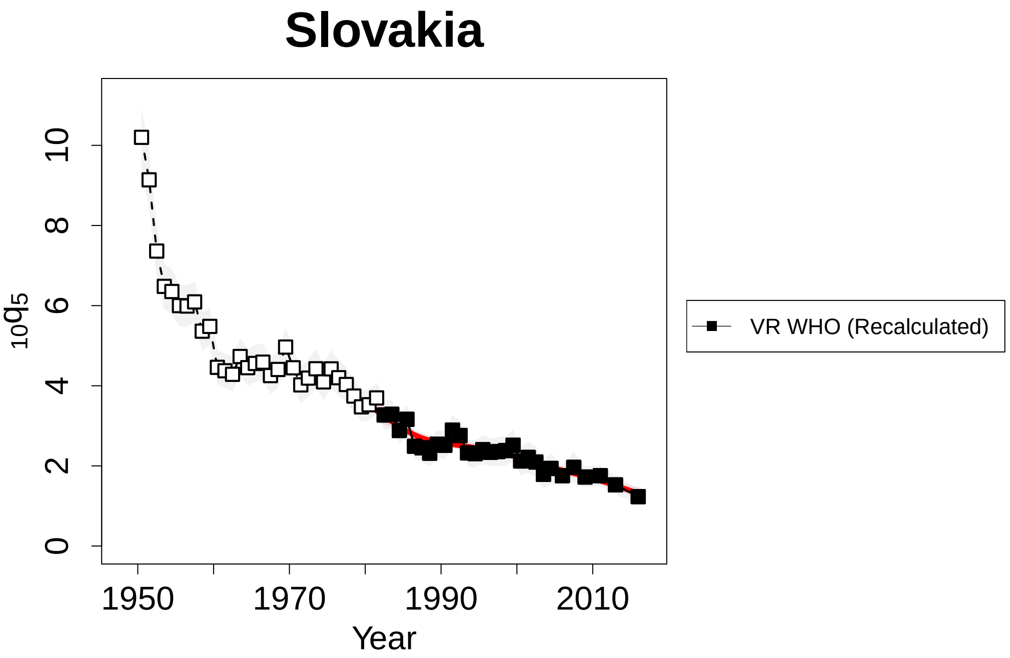
Slovakia
10
8
6
4
2
0
$_{10}q_5$
1950
1970
1990
2010
Year
VR WHO (Recalculated)

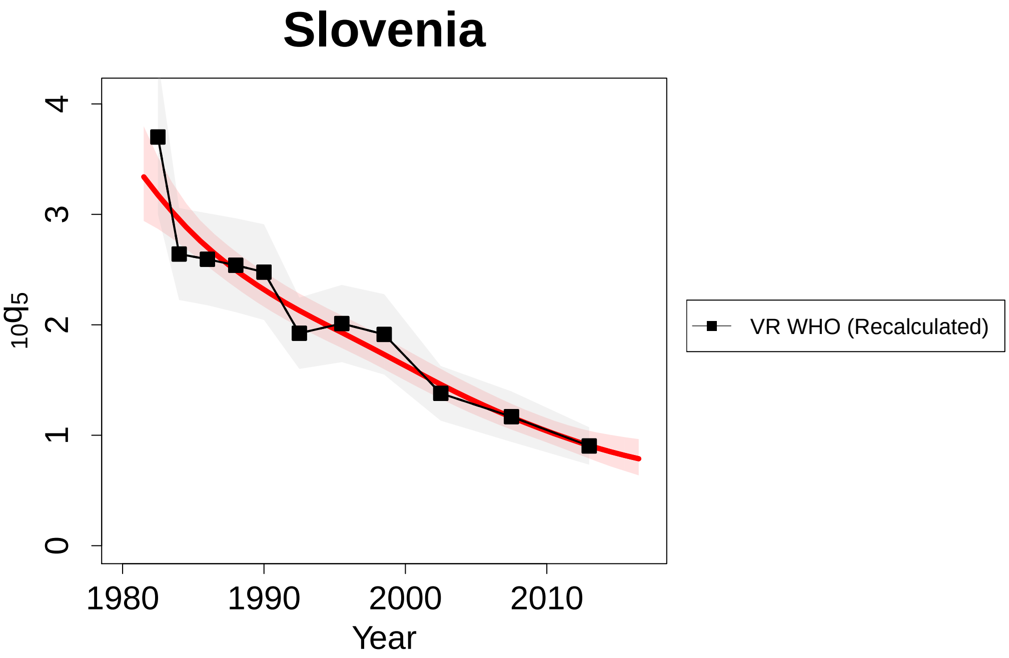
Slovenia
$_{10}q_5$
0
1
2
3
4
1980
1990
2000
2010
Year
VR WHO (Recalculated)

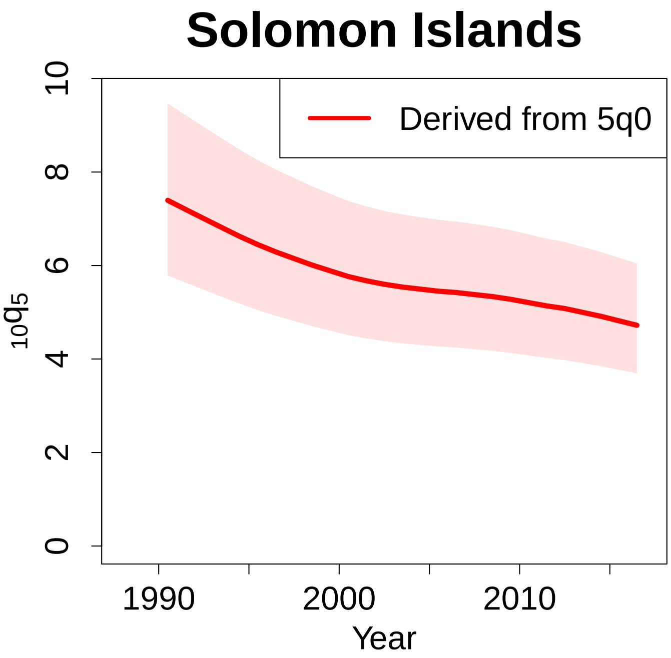
Solomon Islands
$_{10}q_5$
0
2
4
6
8
10
1990
2000
2010
Year
Derived from 5q0

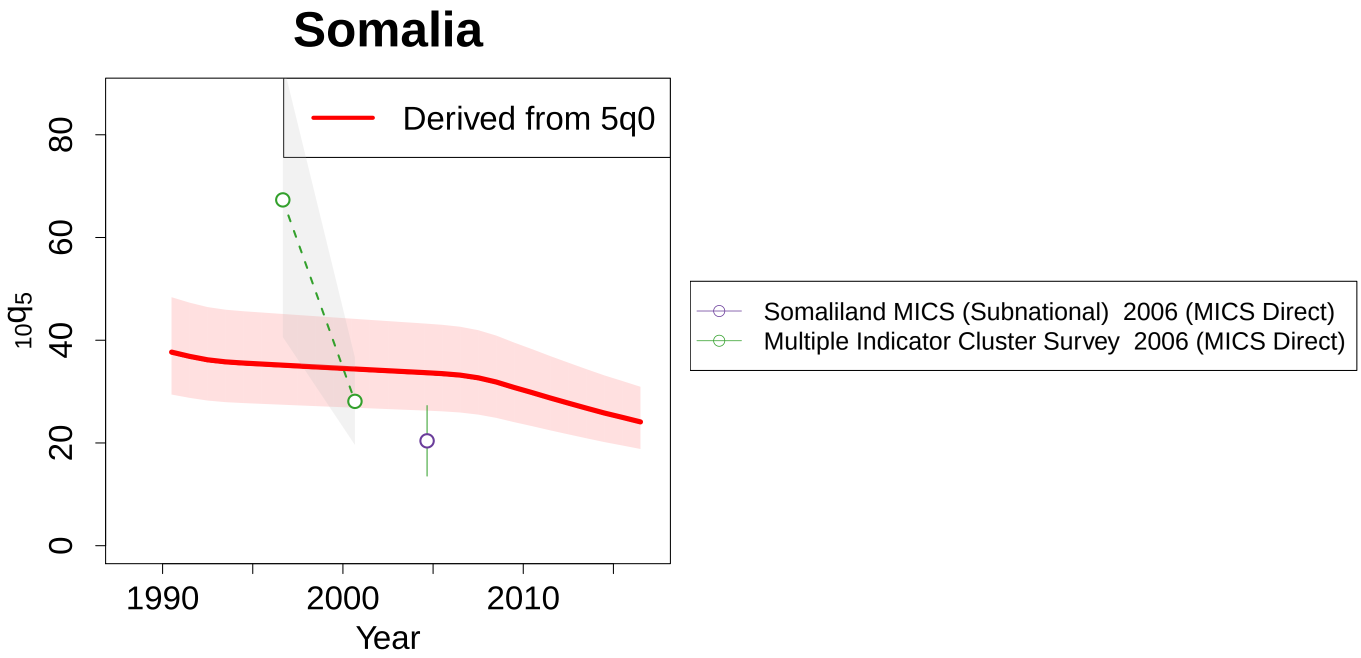
Somalia
Derived from 5q0
$_{10}q_5$
0
20
40
60
80
1990
2000
2010
Year
Somaliland MICS (Subnational) 2006 (MICS Direct)
Multiple Indicator Cluster Survey 2006 (MICS Direct)

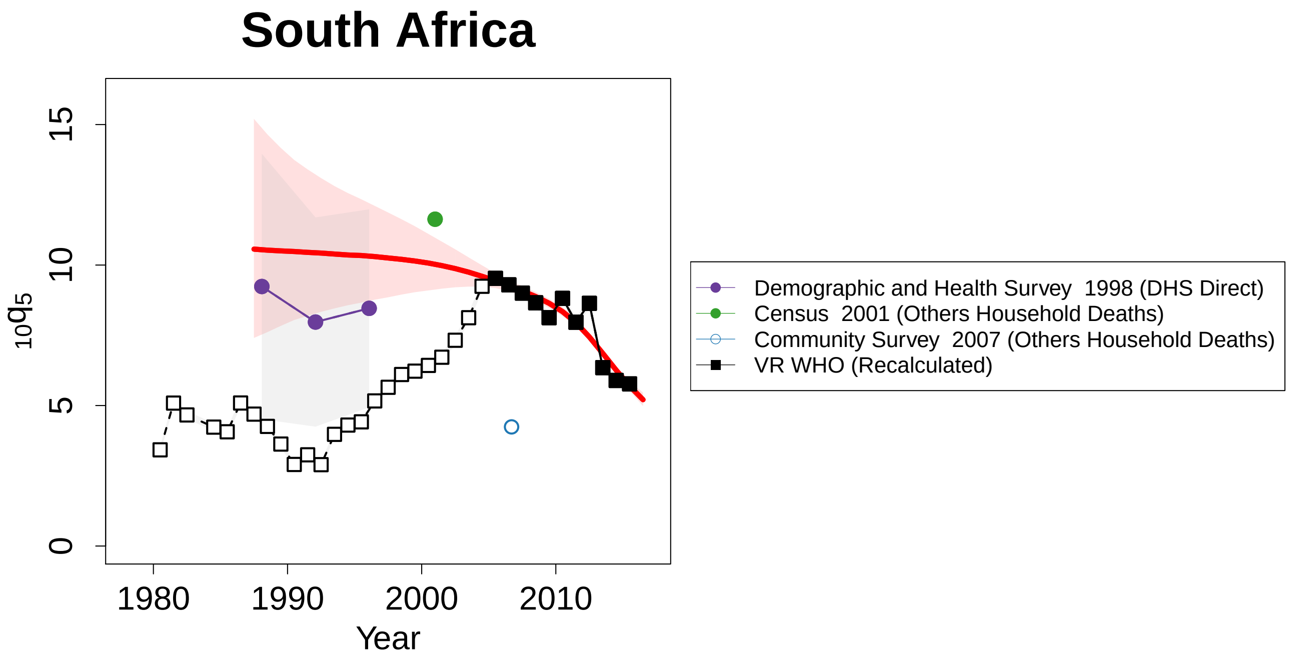
South Africa
$_{10}q_5$
0
5
10
15
1980
1990
2000
2010
Year
Demographic and Health Survey 1998 (DHS Direct)
Census 2001 (Others Household Deaths)
Community Survey 2007 (Others Household Deaths)
VR WHO (Recalculated)

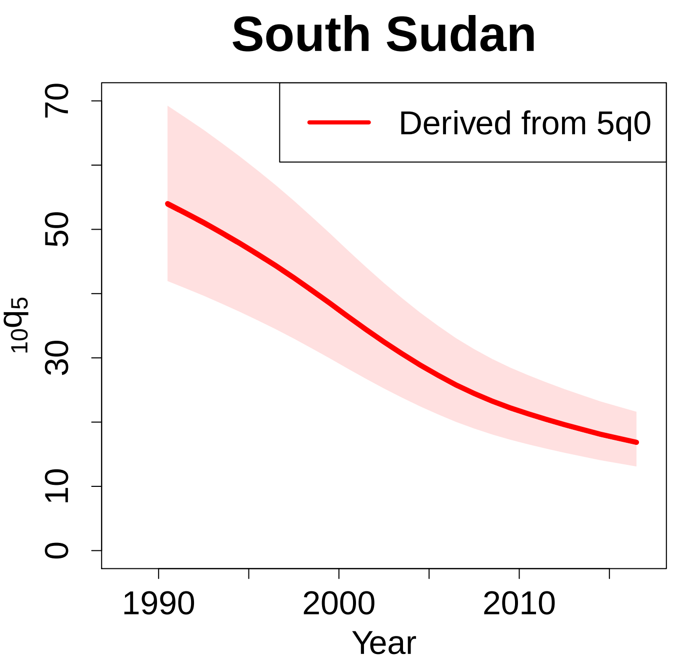
South Sudan
Derived from 5q0
70
50
30
10
0
$_{10}q_5$
1990
2000
2010
Year

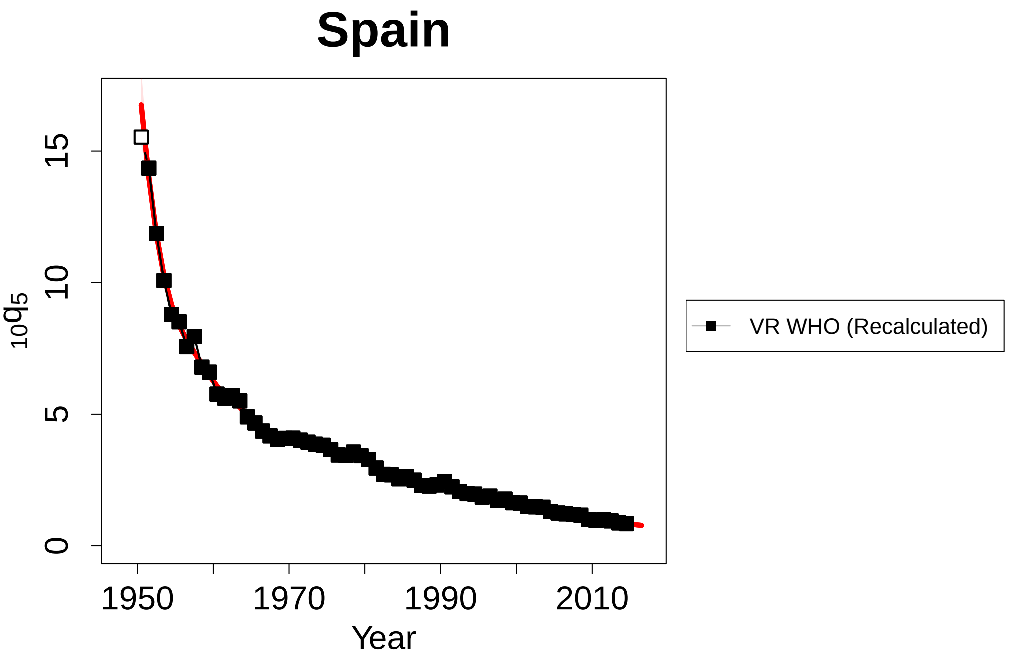
Spain
15
10
5
0
$_{10}q_5$
1950
1970
1990
2010
Year
VR WHO (Recalculated)

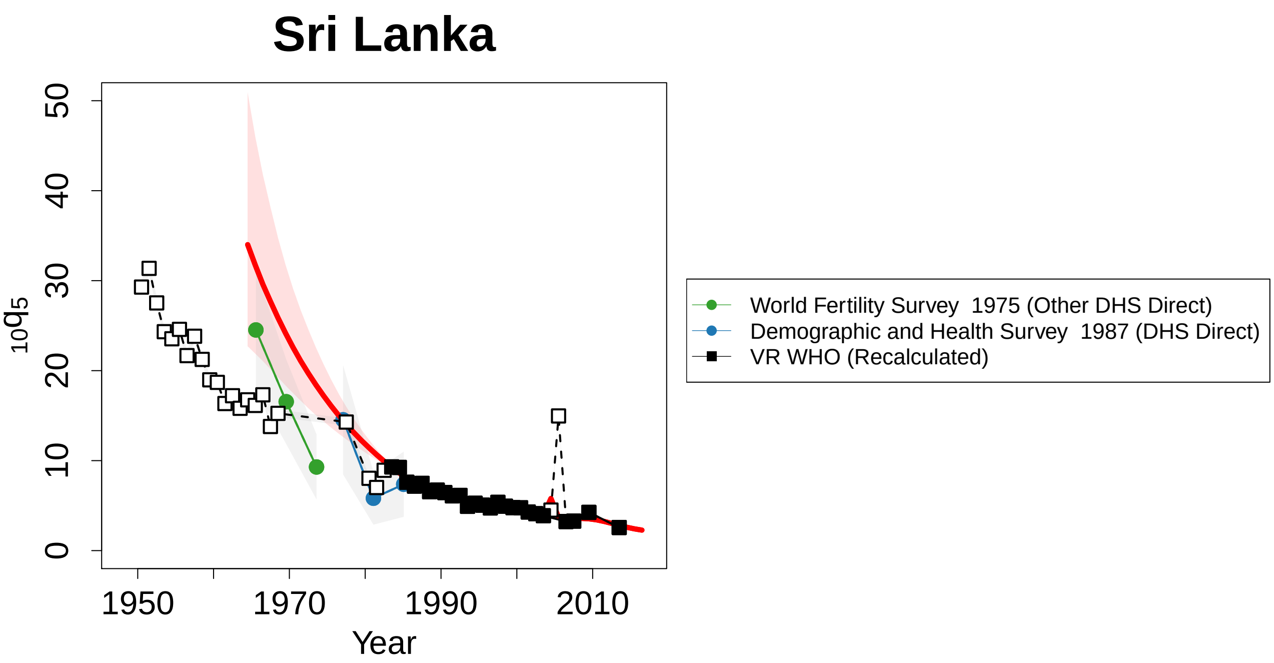
Sri Lanka
$_{10}q_5$
0
10
20
30
40
50
1950
1970
1990
2010
Year
World Fertility Survey 1975 (Other DHS Direct)
Demographic and Health Survey 1987 (DHS Direct)
VR WHO (Recalculated)

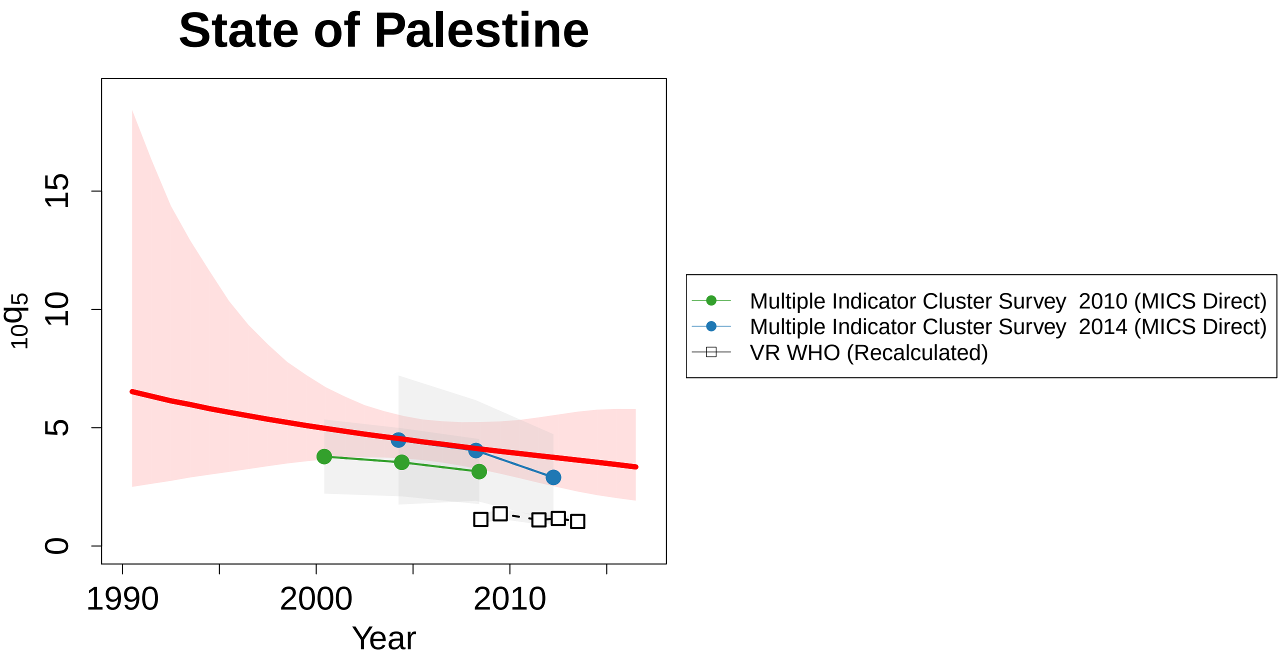
State of Palestine
$_{10}q_5$
0
5
10
15
1990
2000
2010
Year
Multiple Indicator Cluster Survey 2010 (MICS Direct)
Multiple Indicator Cluster Survey 2014 (MICS Direct)
VR WHO (Recalculated)

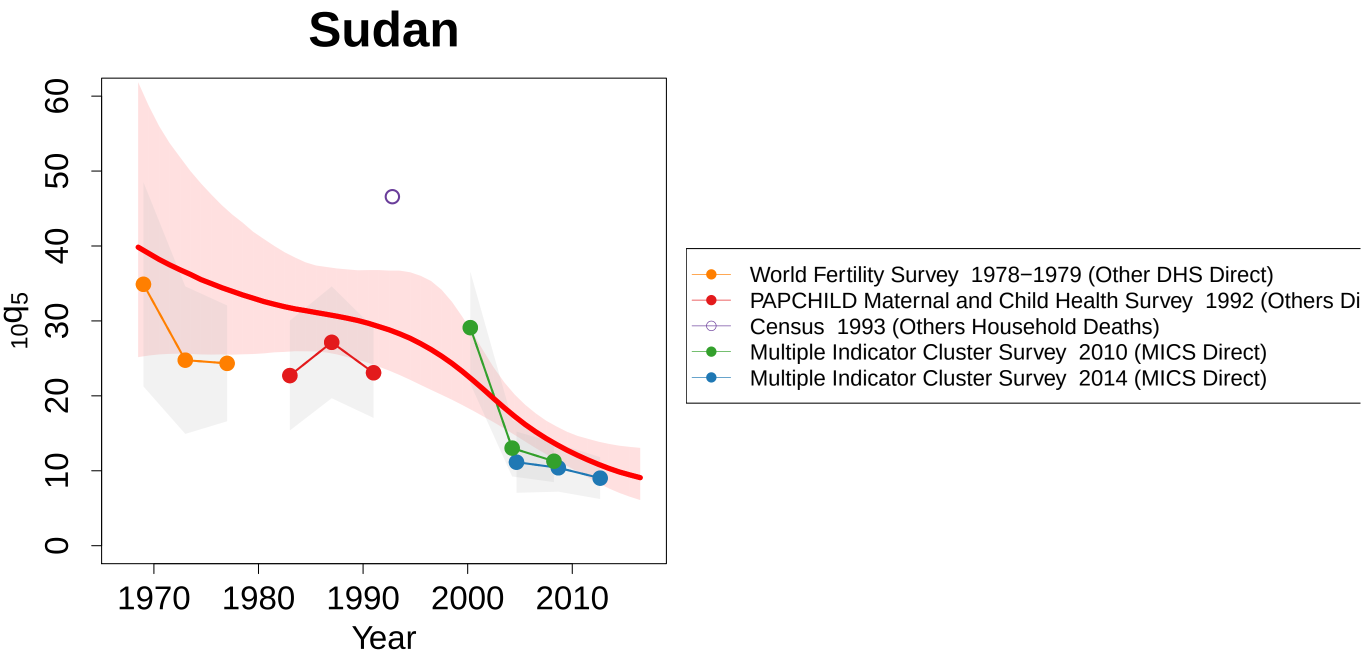
Sudan
$_{10}q_5$
0
10
20
30
40
50
60
1970
1980
1990
2000
2010
Year
World Fertility Survey 1978−1979 (Other DHS Direct)
PAPCHILD Maternal and Child Health Survey 1992 (Others Di
Census 1993 (Others Household Deaths)
Multiple Indicator Cluster Survey 2010 (MICS Direct)
Multiple Indicator Cluster Survey 2014 (MICS Direct)

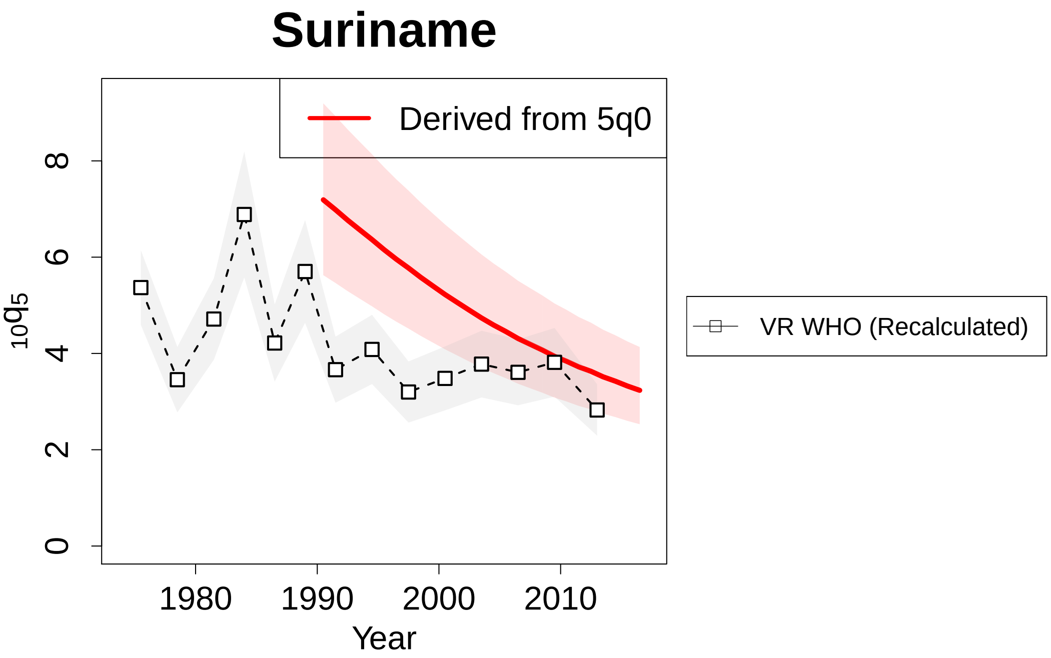
Suriname
Derived from 5q0
$_{10}q_5$
0
2
4
6
8
1980
1990
2000
2010
Year
VR WHO (Recalculated)

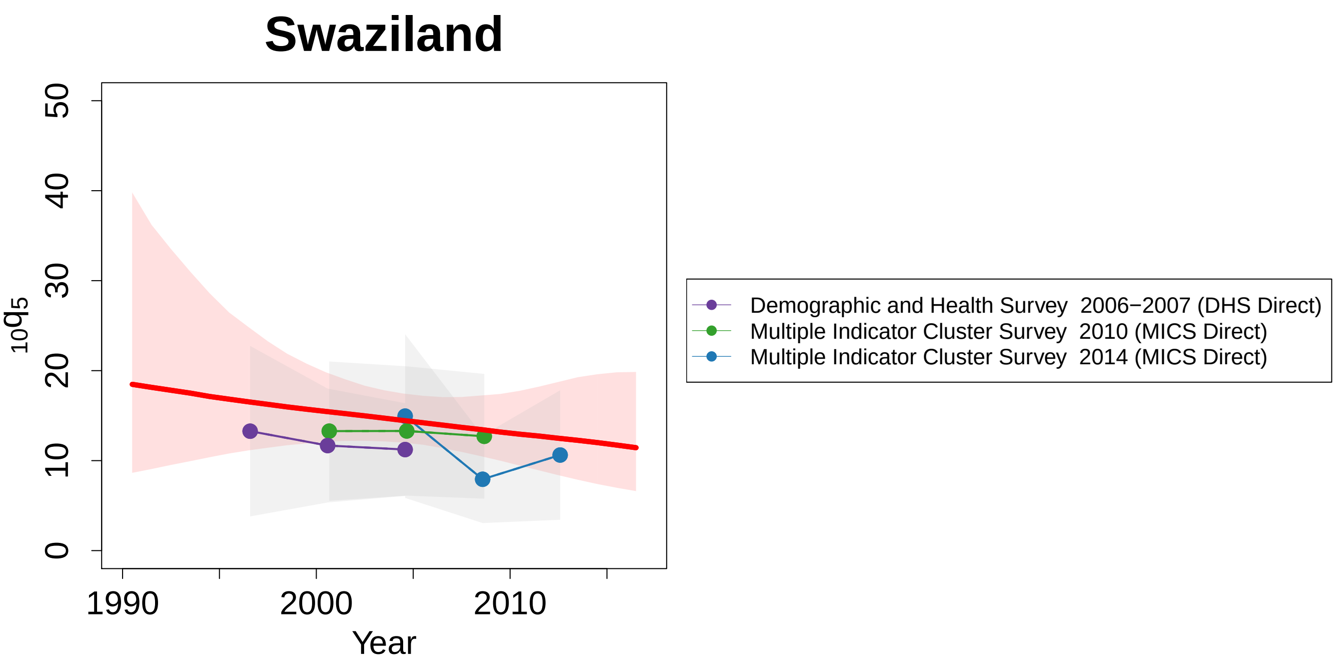
Swaziland
$_{10}q_5$
0
10
20
30
40
50
1990
2000
2010
Year
Demographic and Health Survey 2006−2007 (DHS Direct)
Multiple Indicator Cluster Survey 2010 (MICS Direct)
Multiple Indicator Cluster Survey 2014 (MICS Direct)

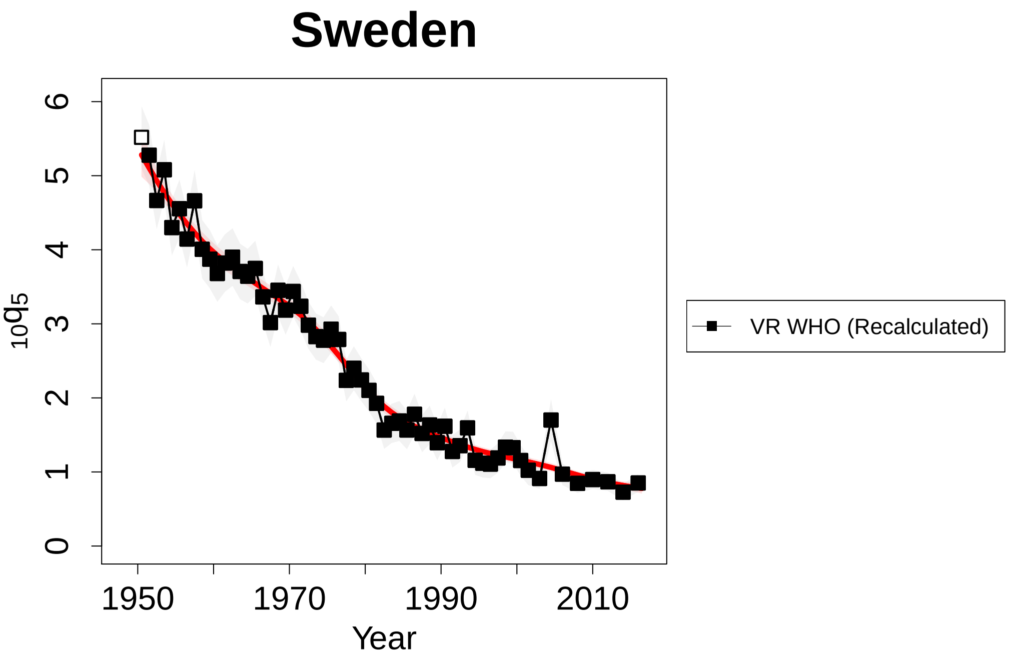
Sweden
$_{10}q_5$
0
1
2
3
4
5
6
1950
1970
1990
2010
Year
VR WHO (Recalculated)

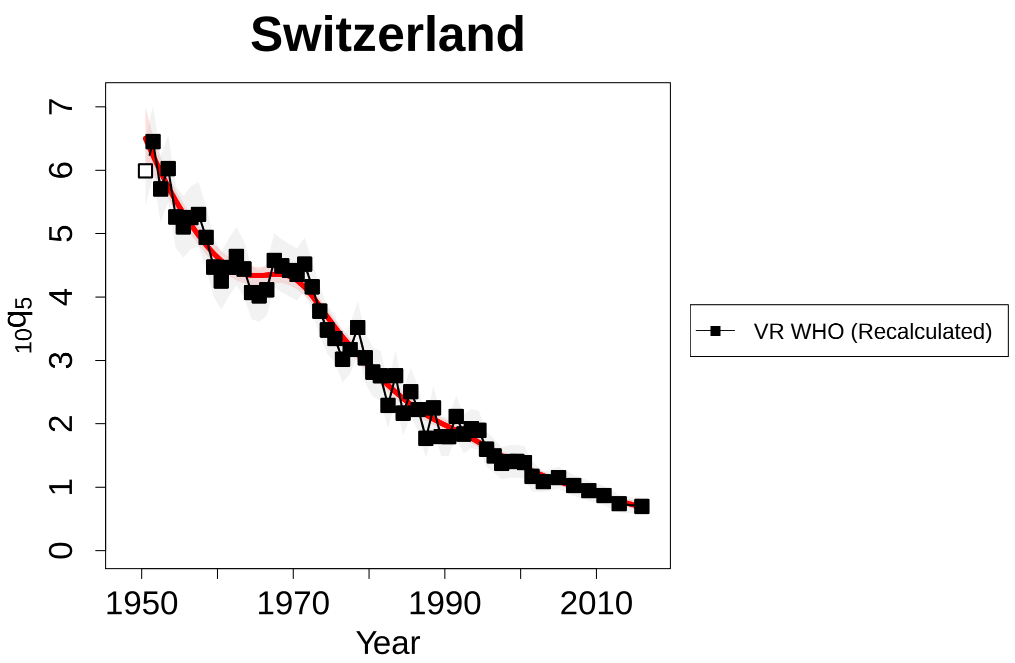
Switzerland
$_{10}q_5$
0
1
2
3
4
5
6
7
1950
1970
1990
2010
Year
VR WHO (Recalculated)

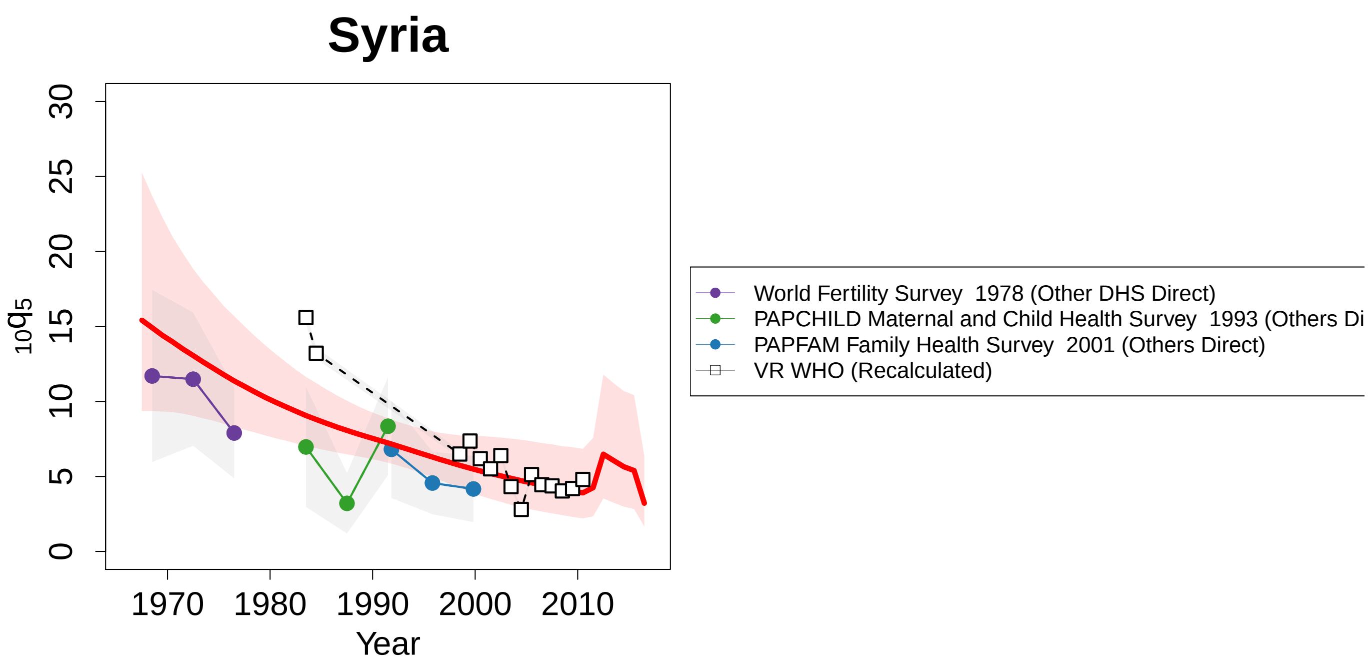
Syria
$_{10}q_5$
0
5
10
15
20
25
30
1970
1980
1990
2000
2010
Year
World Fertility Survey 1978 (Other DHS Direct)
PAPCHILD Maternal and Child Health Survey 1993 (Others Di
PAPFAM Family Health Survey 2001 (Others Direct)
VR WHO (Recalculated)

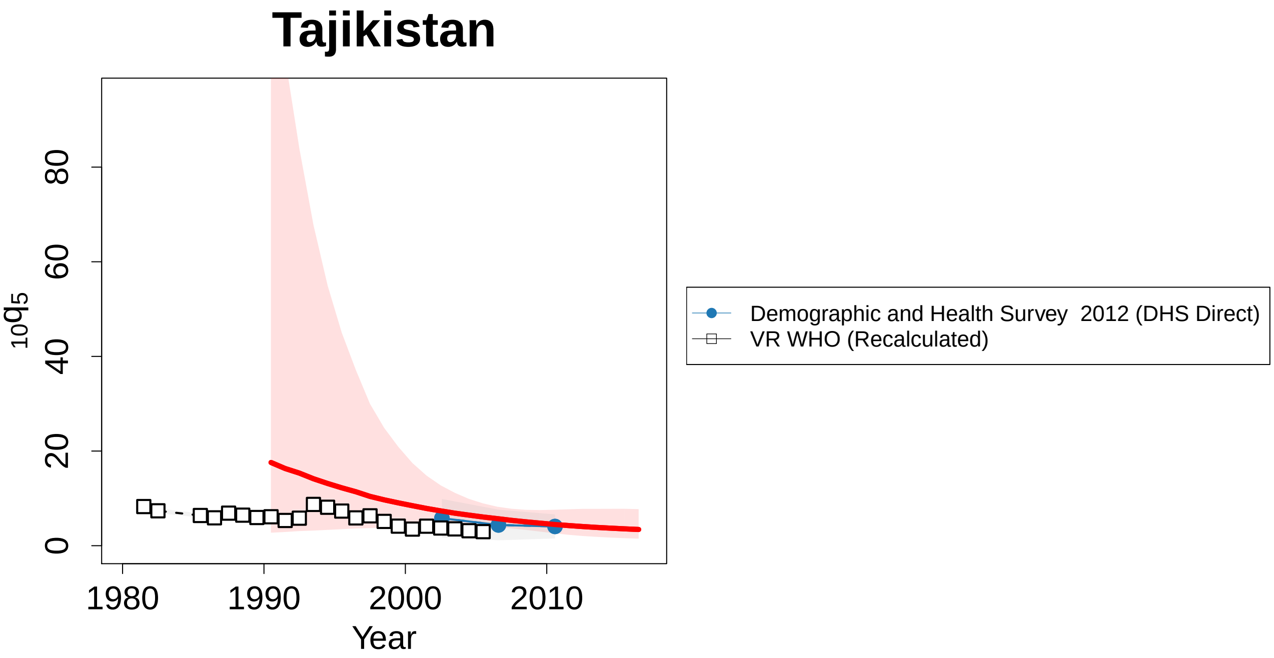
Tajikistan
$_{10}q_5$
0
20
40
60
80
1980
1990
2000
2010
Year
Demographic and Health Survey 2012 (DHS Direct)
VR WHO (Recalculated)

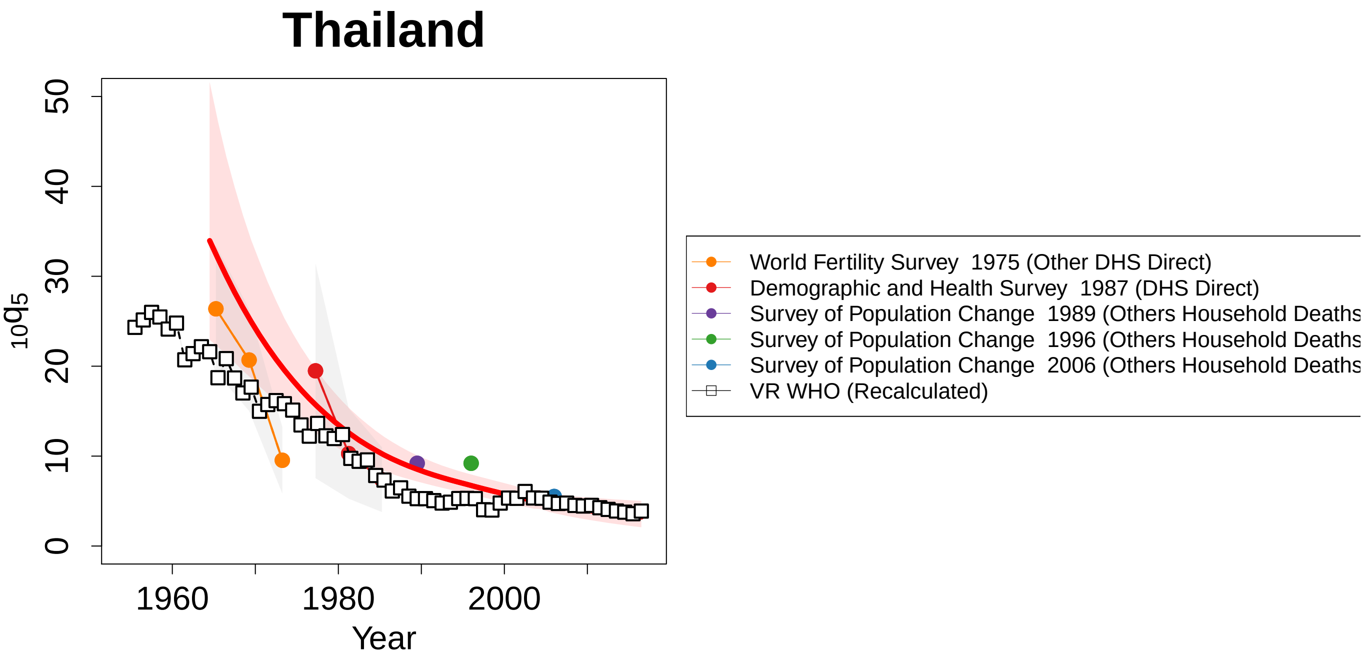
Thailand
$_{10}q_5$
0
10
20
30
40
50
1960
1980
2000
Year
World Fertility Survey 1975 (Other DHS Direct)
Demographic and Health Survey 1987 (DHS Direct)
Survey of Population Change 1989 (Others Household Deaths
Survey of Population Change 1996 (Others Household Deaths
Survey of Population Change 2006 (Others Household Deaths
VR WHO (Recalculated)

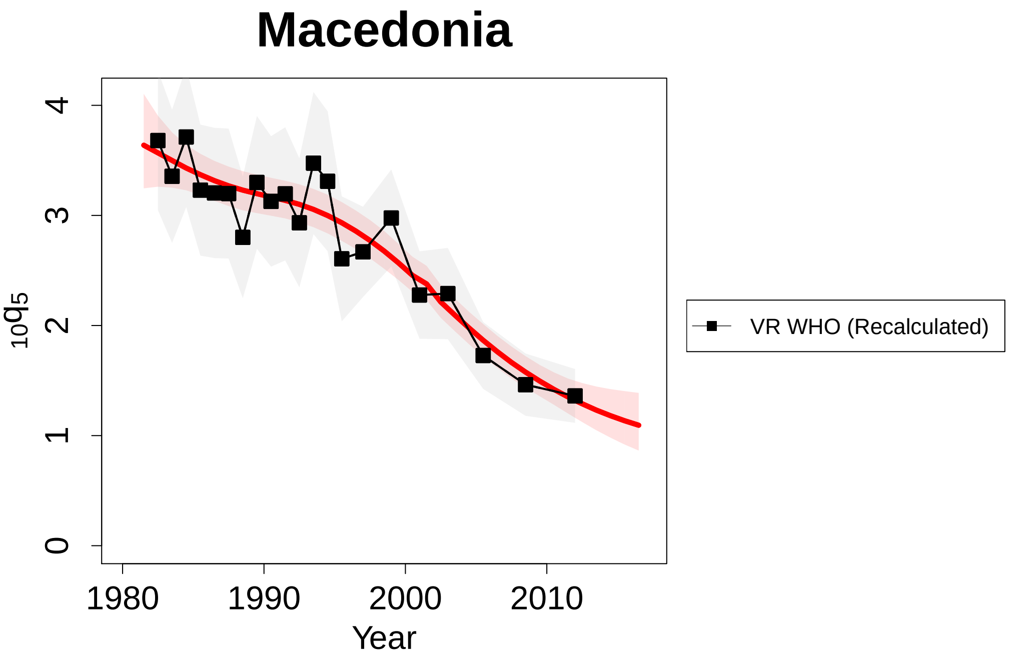
Macedonia
VR WHO (Recalculated)
$_{10}q_5$
Year

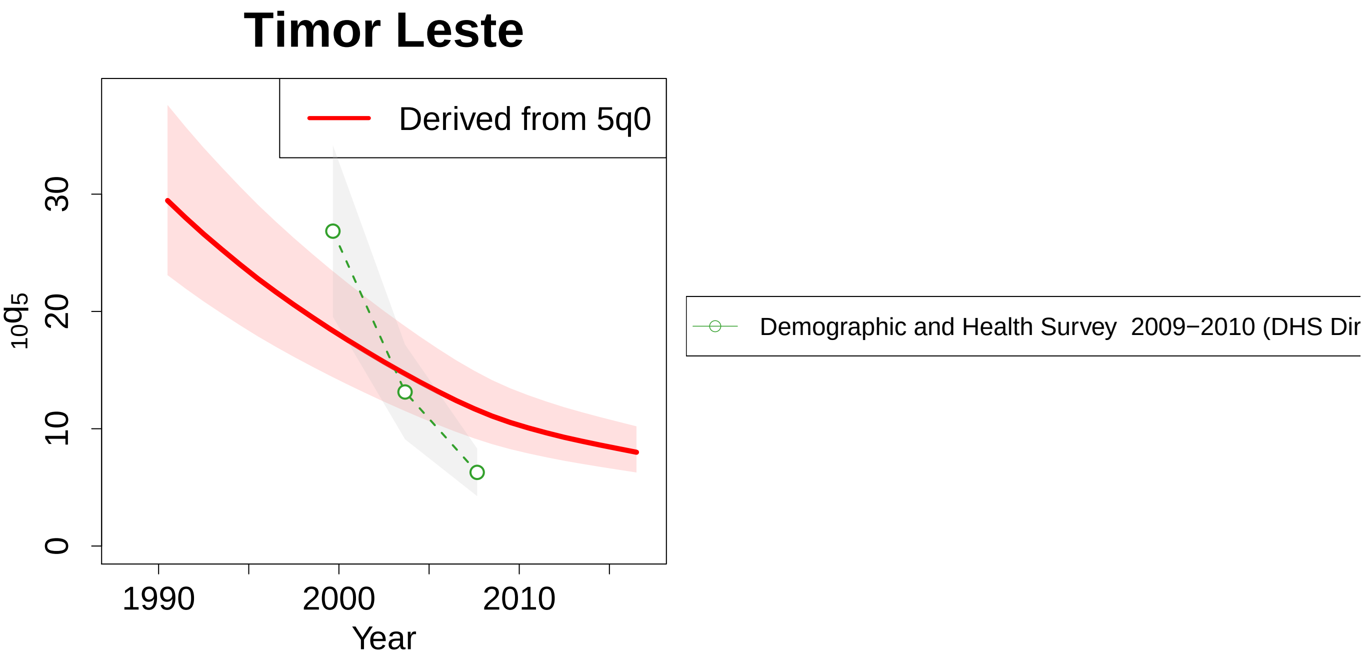
Timor Leste
Derived from 5q0
Demographic and Health Survey 2009−2010 (DHS Dir
$_{10}q_5$
Year

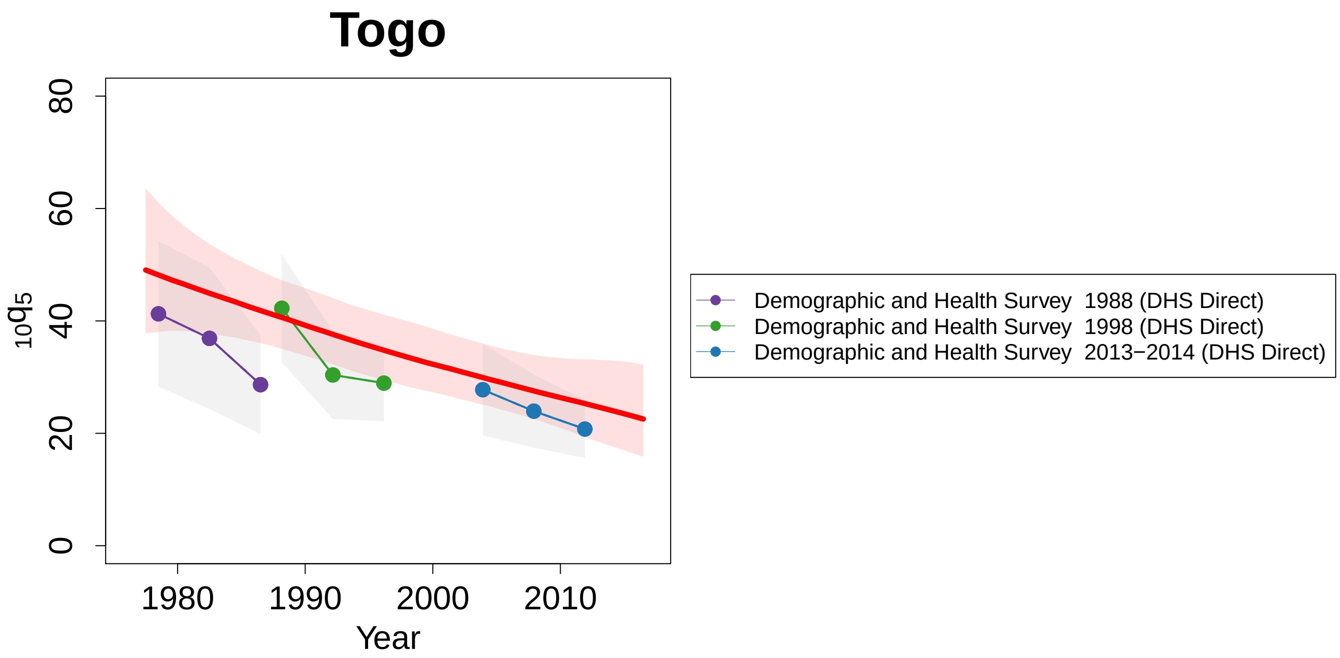
Togo
80
60
40
20
0
10q5
1980
1990
2000
2010
Year
Demographic and Health Survey 1988 (DHS Direct)
Demographic and Health Survey 1998 (DHS Direct)
Demographic and Health Survey 2013−2014 (DHS Direct)

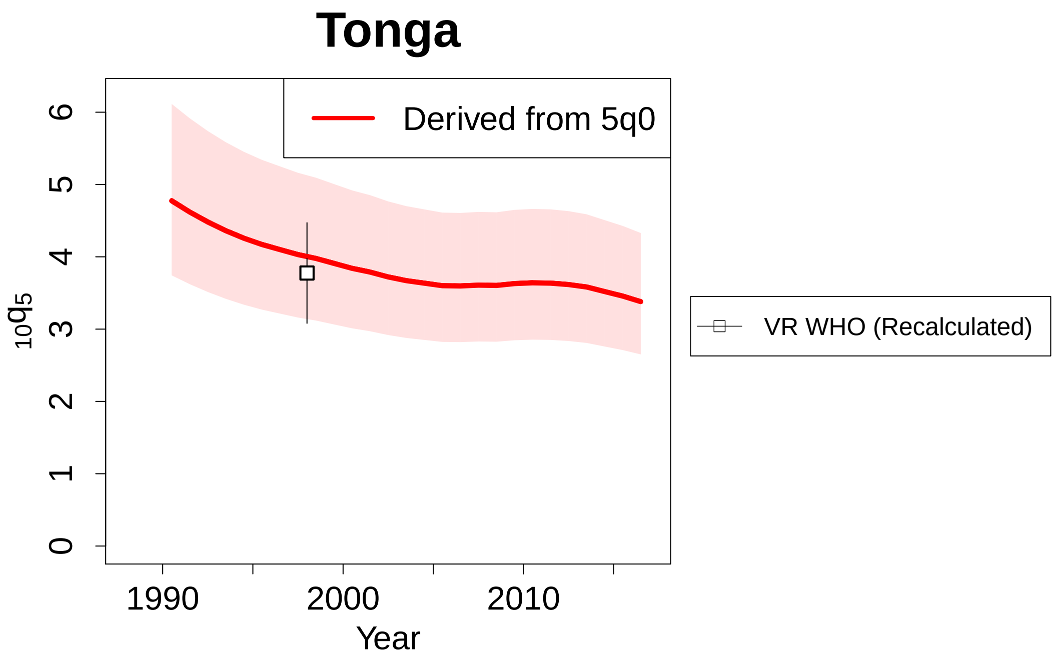
Tonga
Derived from 5q0
6
5
4
3
2
1
0
10q5
1990
2000
2010
Year
VR WHO (Recalculated)

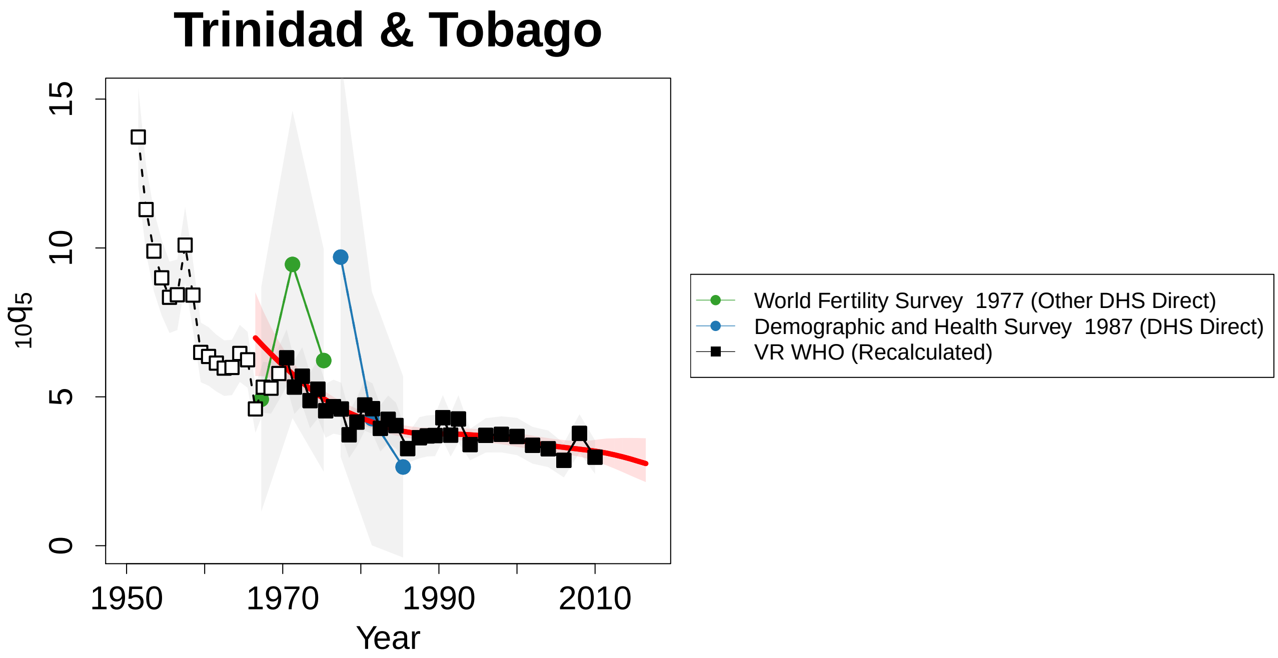
Trinidad & Tobago
$_{10}q_5$
Year
World Fertility Survey 1977 (Other DHS Direct)
Demographic and Health Survey 1987 (DHS Direct)
VR WHO (Recalculated)

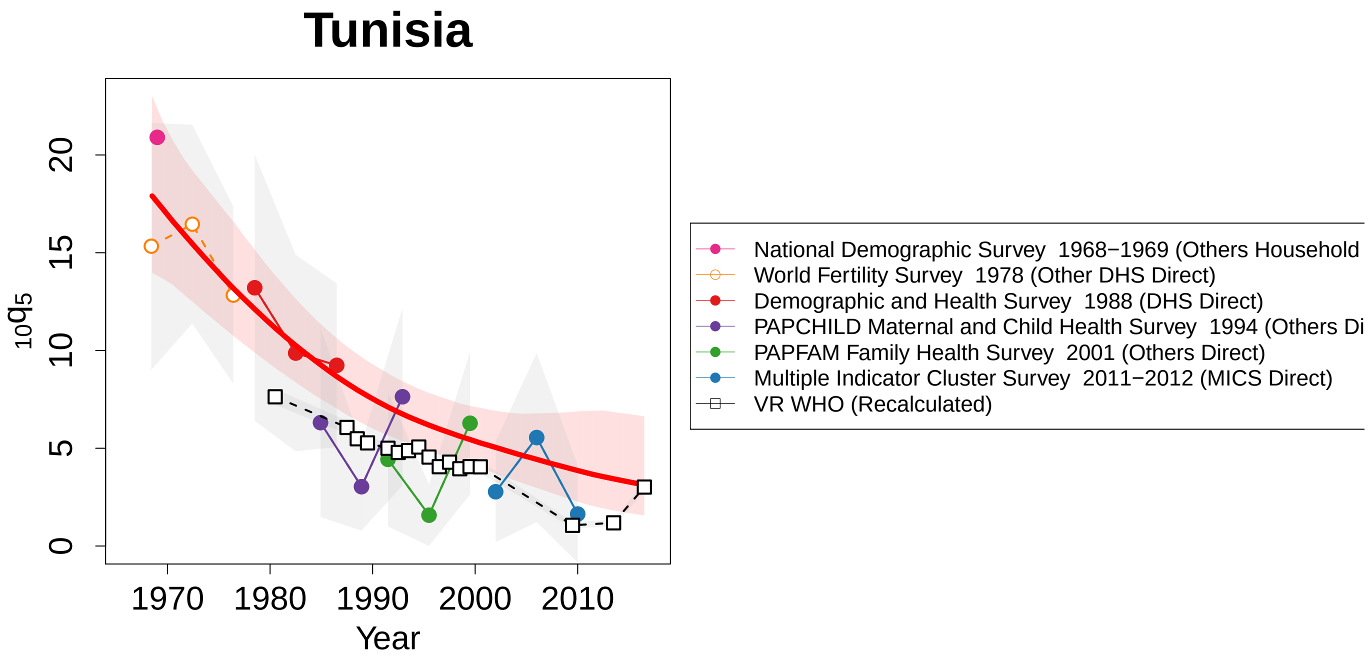
Tunisia
$_{10}q_5$
Year
National Demographic Survey 1968–1969 (Others Household
World Fertility Survey 1978 (Other DHS Direct)
Demographic and Health Survey 1988 (DHS Direct)
PAPCHILD Maternal and Child Health Survey 1994 (Others Di
PAPFAM Family Health Survey 2001 (Others Direct)
Multiple Indicator Cluster Survey 2011–2012 (MICS Direct)
VR WHO (Recalculated)

Turkey

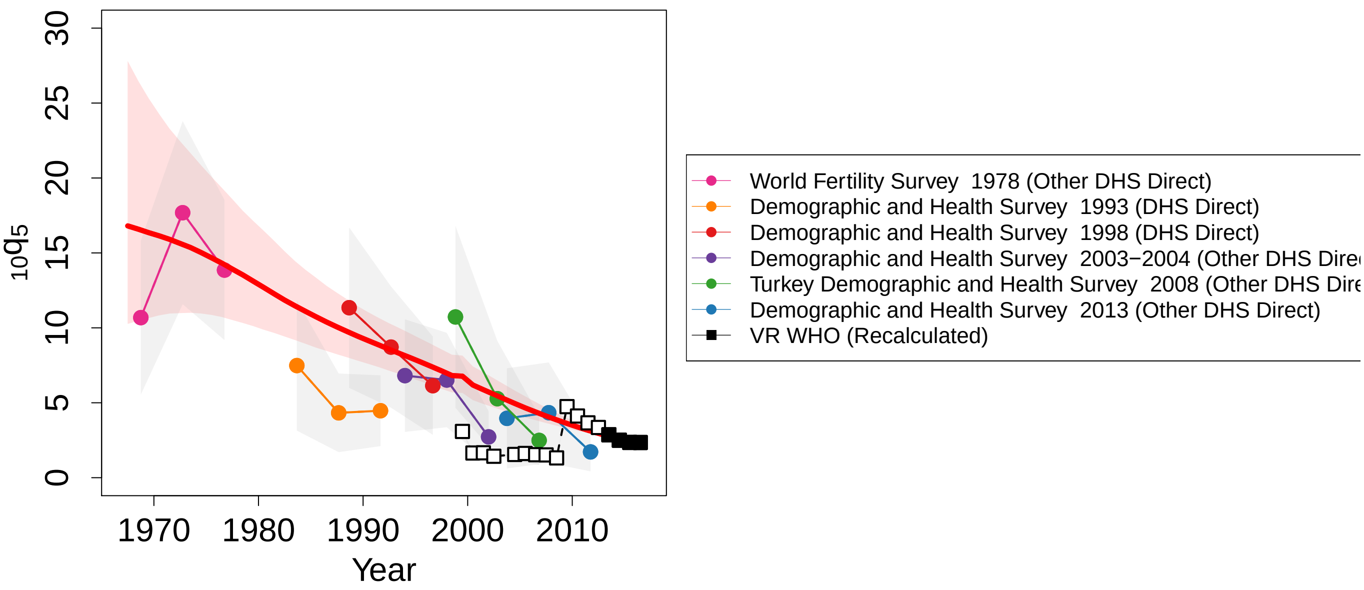

Turkmenistan

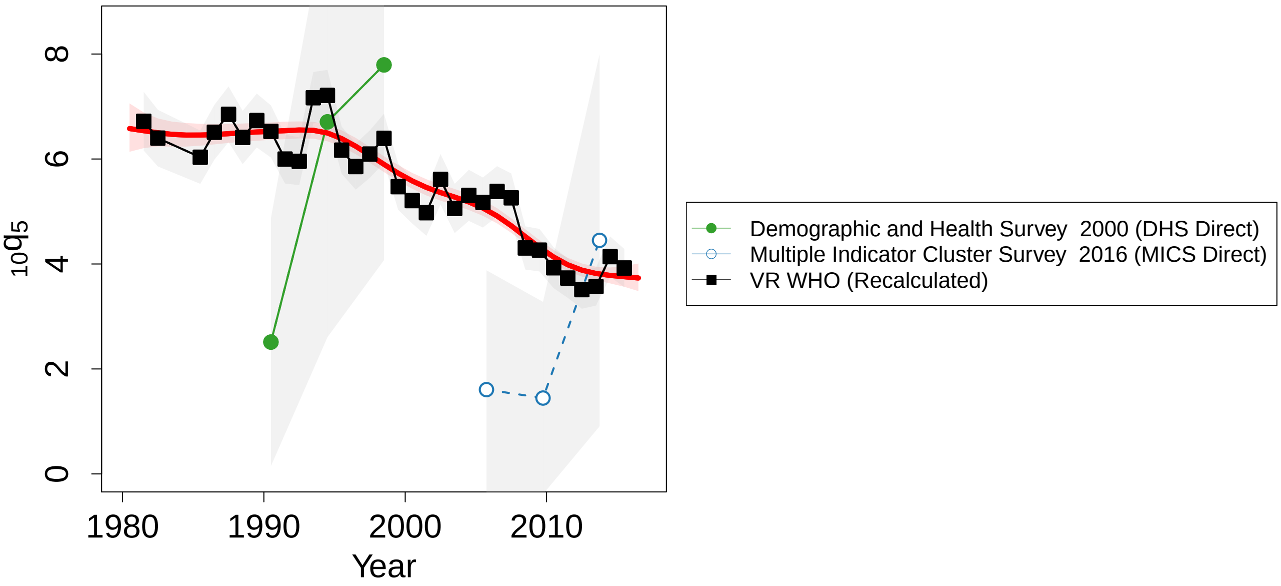

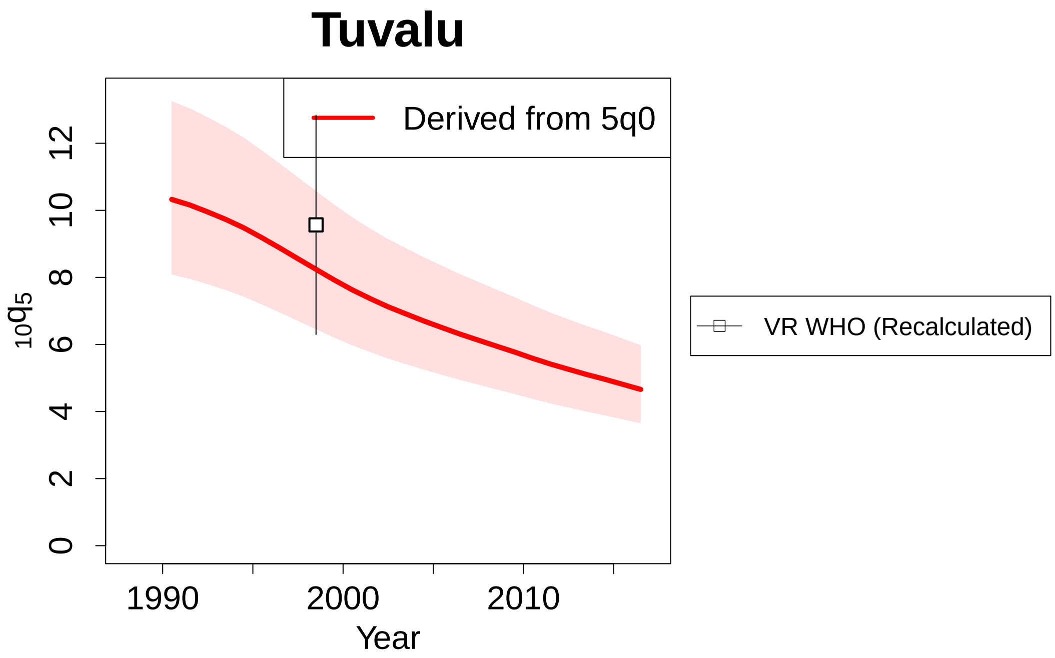
Tuvalu
Derived from 5q0
VR WHO (Recalculated)
$_{10}q_5$
Year

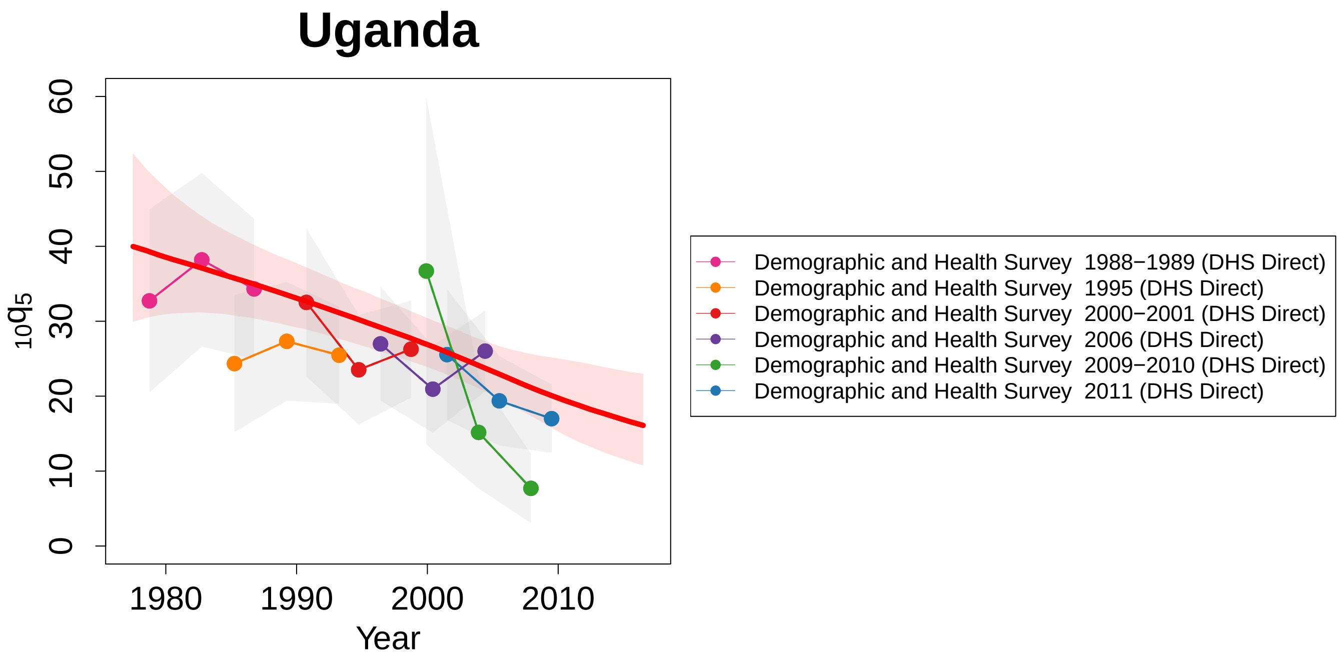
Uganda
Demographic and Health Survey 1988−1989 (DHS Direct)
Demographic and Health Survey 1995 (DHS Direct)
Demographic and Health Survey 2000−2001 (DHS Direct)
Demographic and Health Survey 2006 (DHS Direct)
Demographic and Health Survey 2009−2010 (DHS Direct)
Demographic and Health Survey 2011 (DHS Direct)
$_{10}q_5$
Year

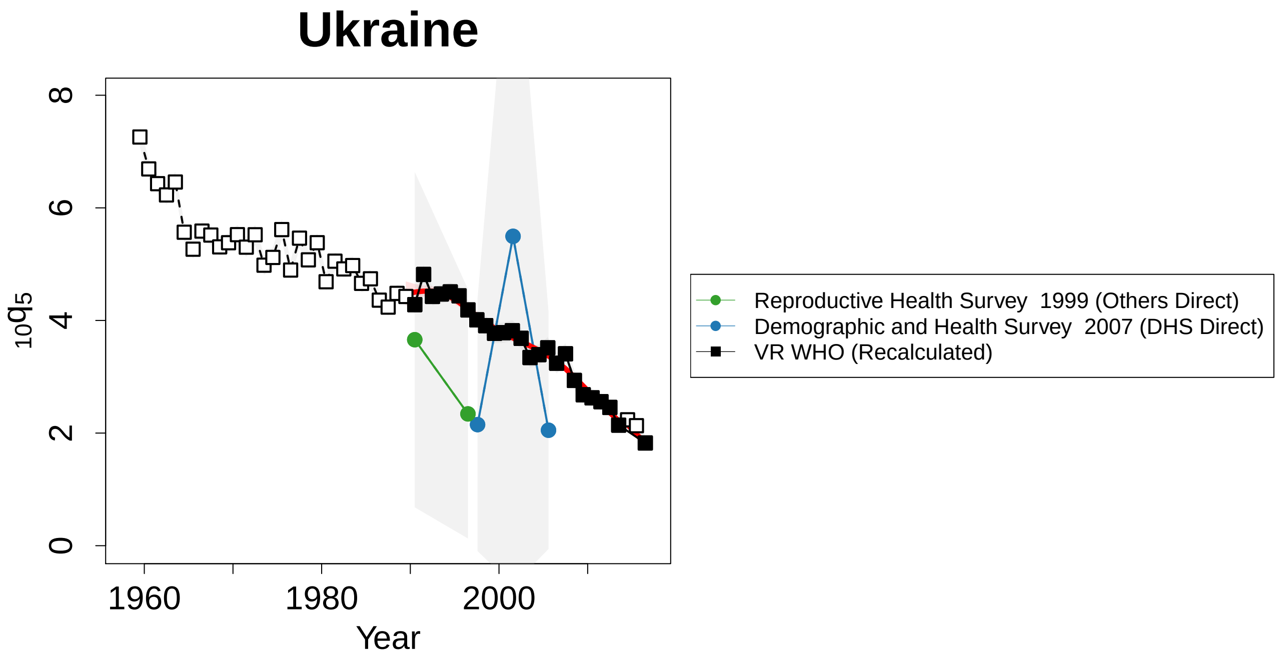
Ukraine
8
6
4
2
0
$_{10}q_5$
1960
1980
2000
Year
Reproductive Health Survey 1999 (Others Direct)
Demographic and Health Survey 2007 (DHS Direct)
VR WHO (Recalculated)

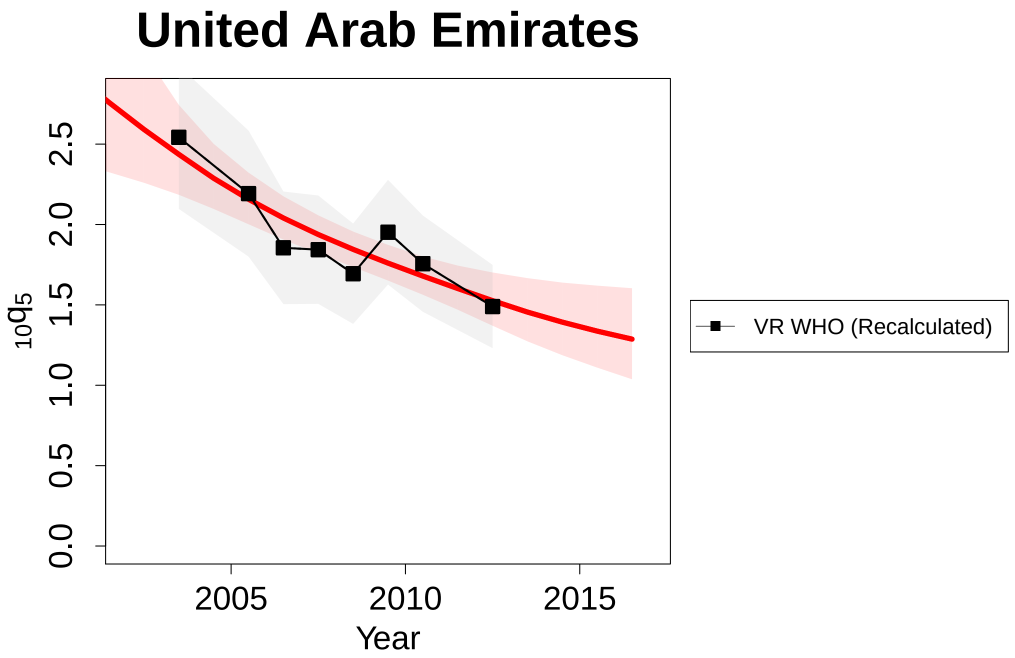
United Arab Emirates
2.5
2.0
1.5
1.0
0.5
0.0
$_{10}q_5$
2005
2010
2015
Year
VR WHO (Recalculated)

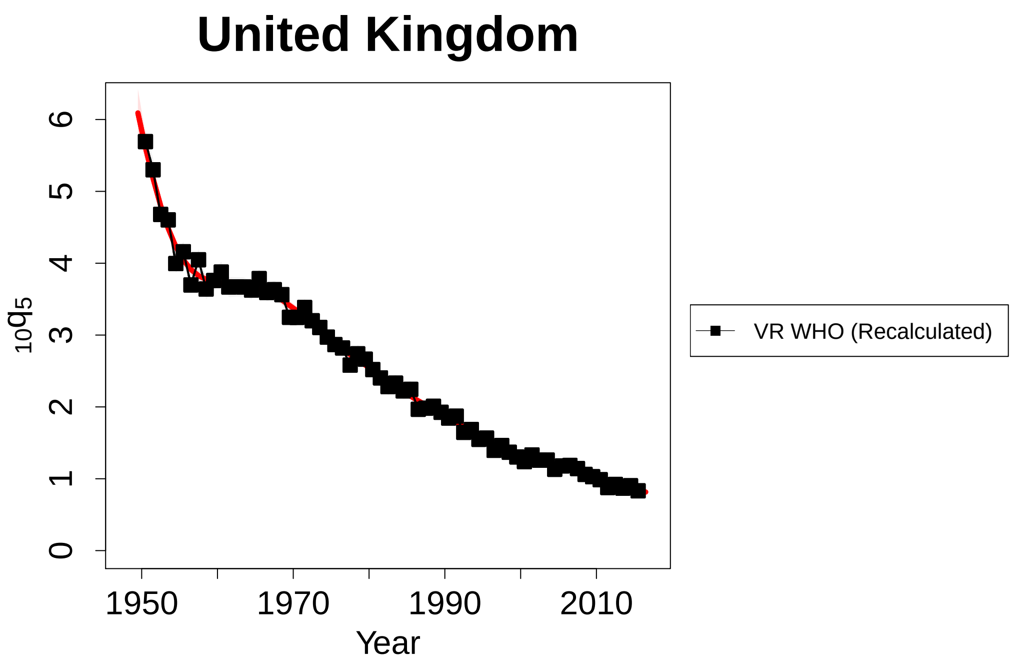
United Kingdom
$_{10}q_5$
Year
VR WHO (Recalculated)

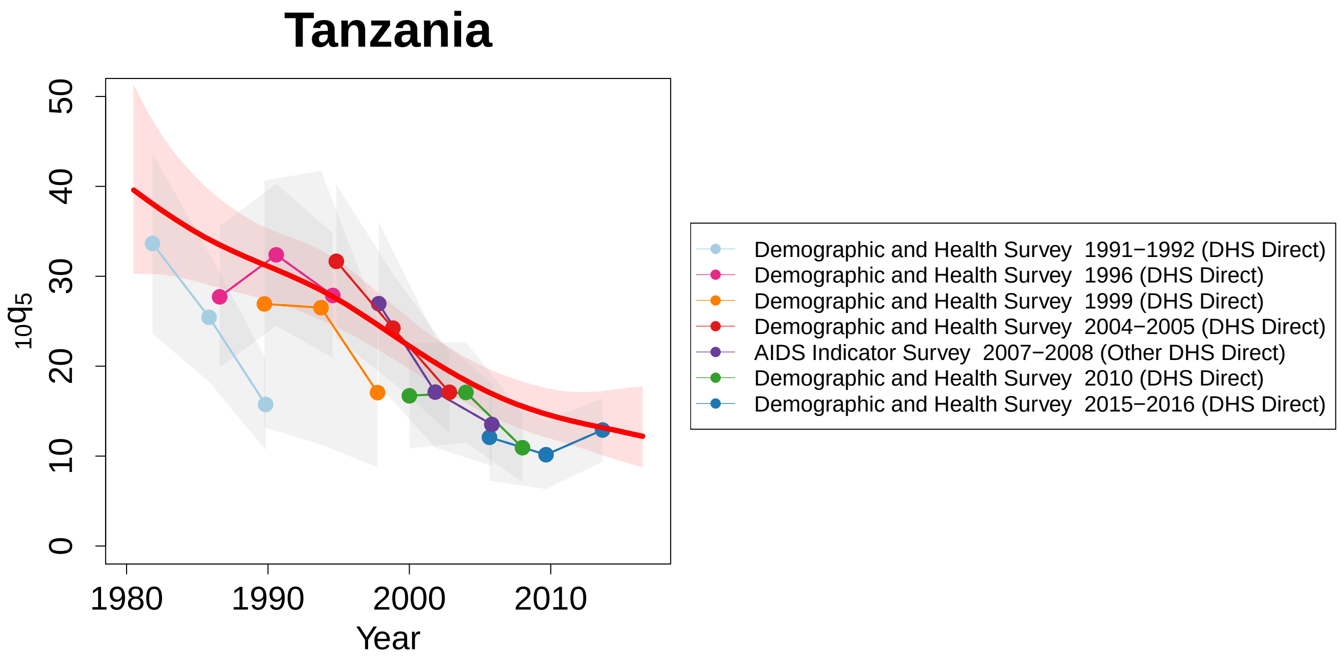
Tanzania
$_{10}q_5$
Year
Demographic and Health Survey 1991−1992 (DHS Direct)
Demographic and Health Survey 1996 (DHS Direct)
Demographic and Health Survey 1999 (DHS Direct)
Demographic and Health Survey 2004−2005 (DHS Direct)
AIDS Indicator Survey 2007−2008 (Other DHS Direct)
Demographic and Health Survey 2010 (DHS Direct)
Demographic and Health Survey 2015−2016 (DHS Direct)

United States of America

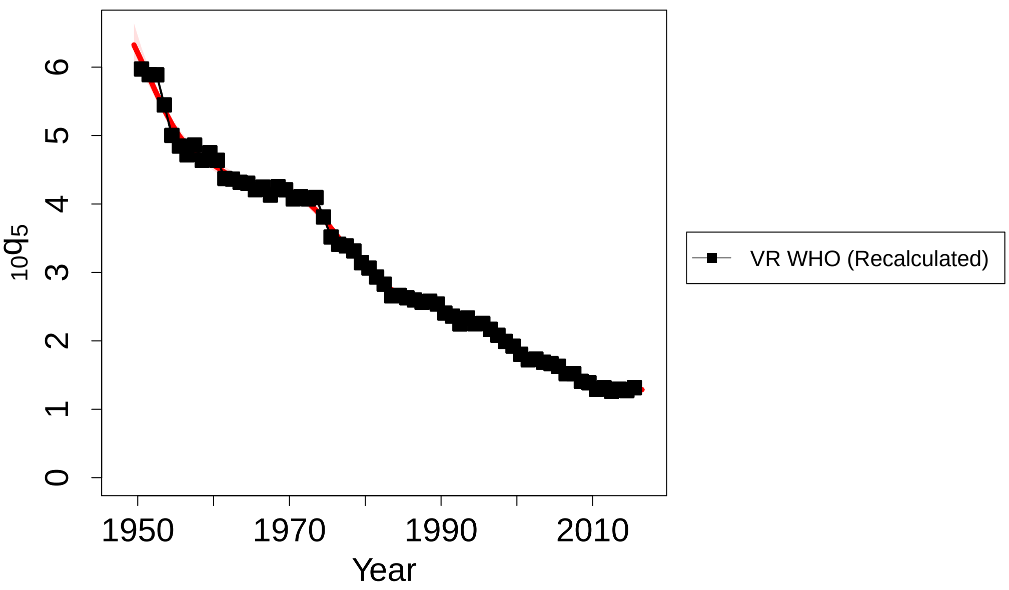

Uruguay

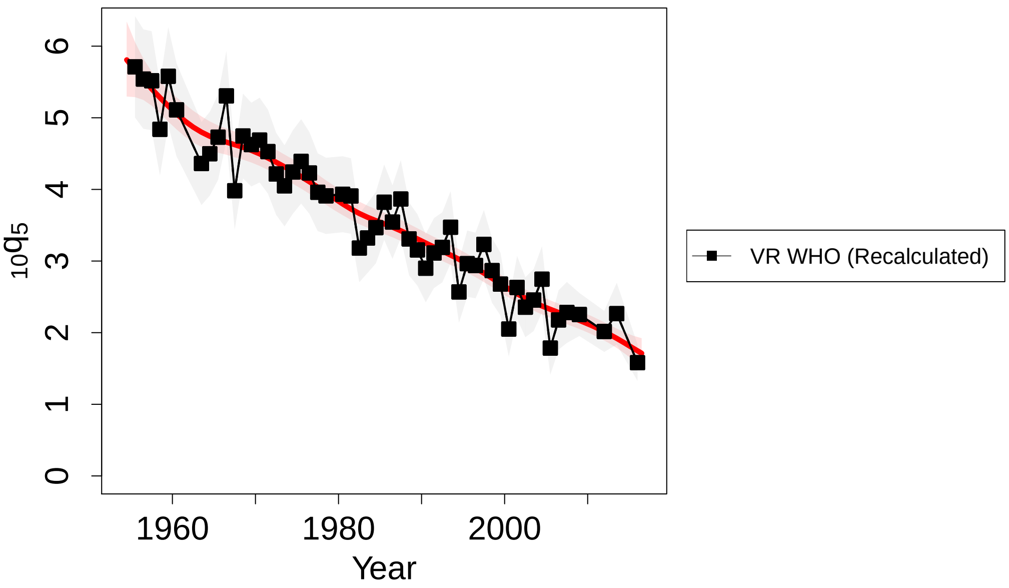

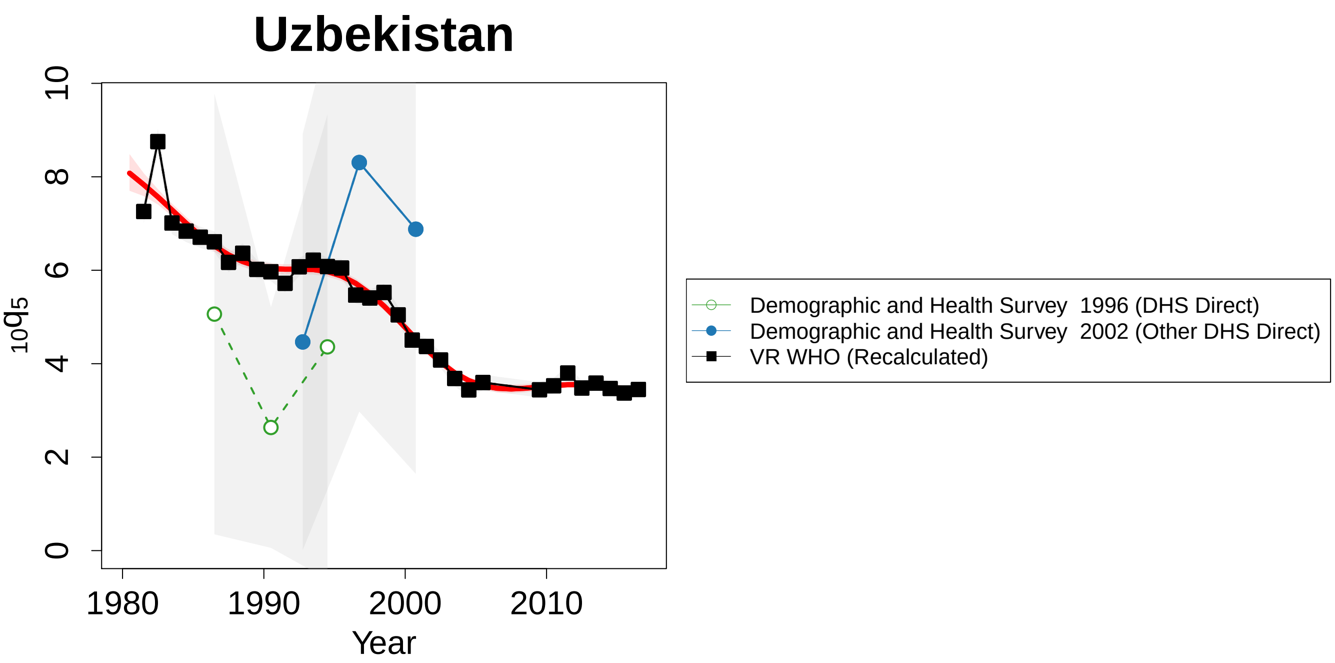
Uzbekistan
10
8
6
4
2
0
$_{10}q_5$
1980
1990
2000
2010
Year
Demographic and Health Survey 1996 (DHS Direct)
Demographic and Health Survey 2002 (Other DHS Direct)
VR WHO (Recalculated)

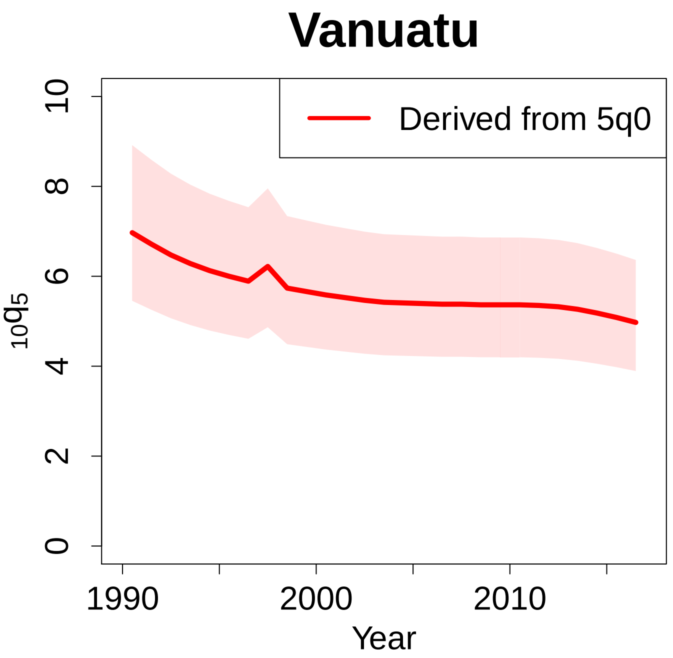
Vanuatu
Derived from 5q0
10
8
6
4
2
0
$_{10}q_5$
1990
2000
2010
Year

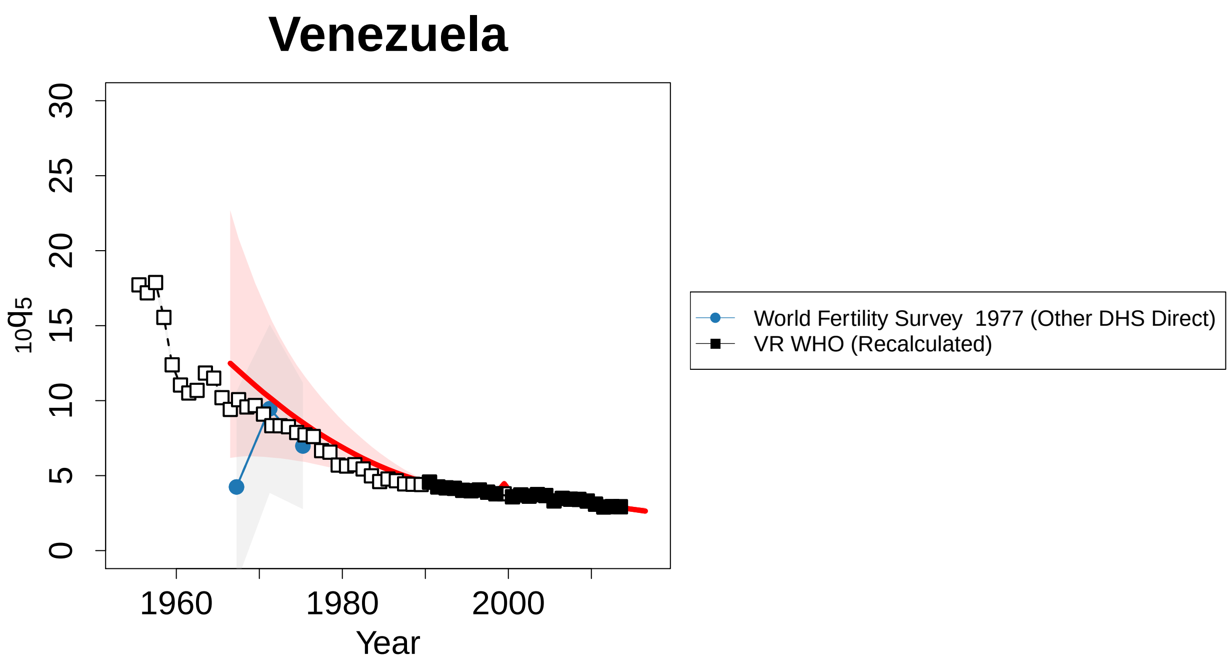
Venezuela
$_{10}q_5$
0
5
10
15
20
25
30
1960
1980
2000
Year
World Fertility Survey 1977 (Other DHS Direct)
VR WHO (Recalculated)

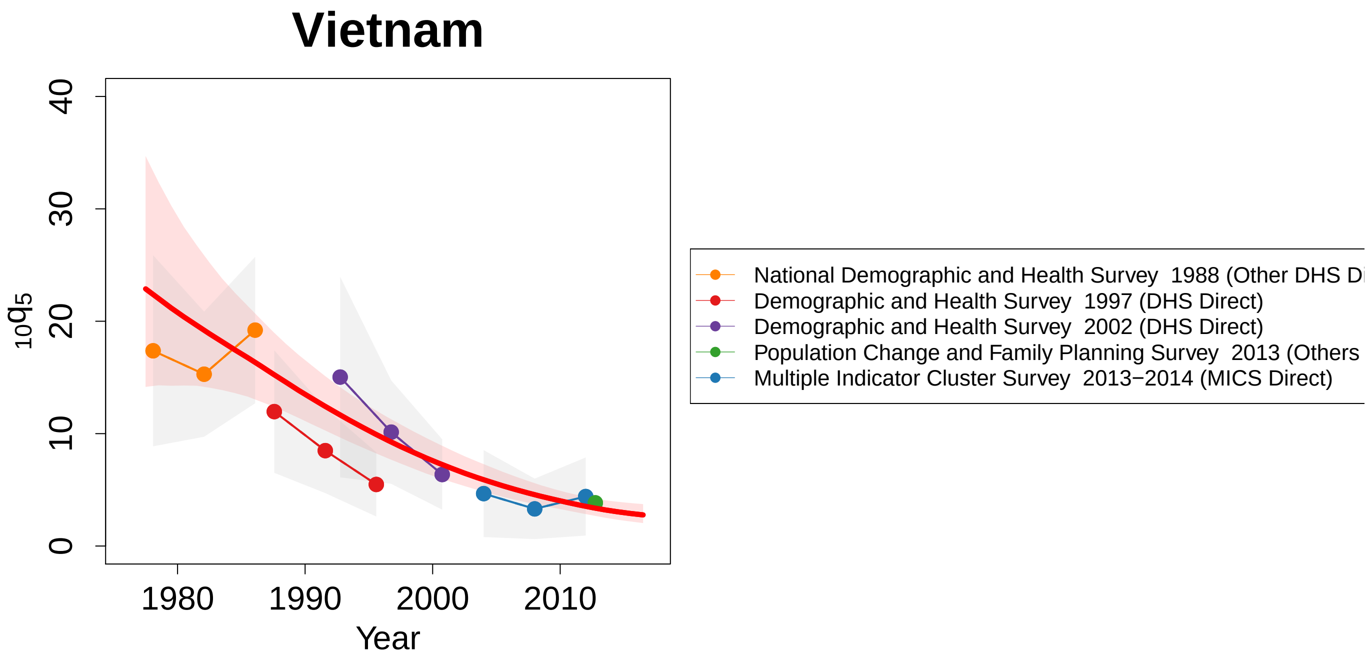
Vietnam
$_{10}q_5$
0
10
20
30
40
1980
1990
2000
2010
Year
National Demographic and Health Survey 1988 (Other DHS Di
Demographic and Health Survey 1997 (DHS Direct)
Demographic and Health Survey 2002 (DHS Direct)
Population Change and Family Planning Survey 2013 (Others
Multiple Indicator Cluster Survey 2013−2014 (MICS Direct)

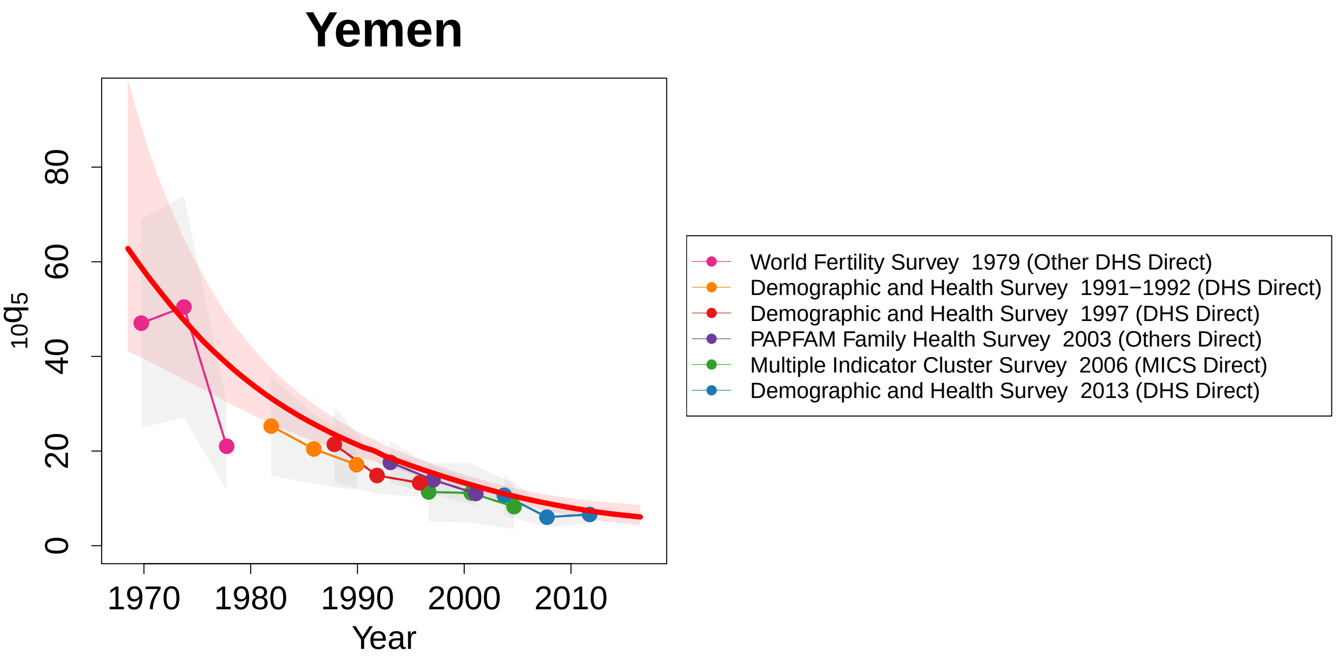
Yemen
0
20
40
60
80
$_{10}q_5$
1970
1980
1990
2000
2010
Year
World Fertility Survey 1979 (Other DHS Direct)
Demographic and Health Survey 1991−1992 (DHS Direct)
Demographic and Health Survey 1997 (DHS Direct)
PAPFAM Family Health Survey 2003 (Others Direct)
Multiple Indicator Cluster Survey 2006 (MICS Direct)
Demographic and Health Survey 2013 (DHS Direct)

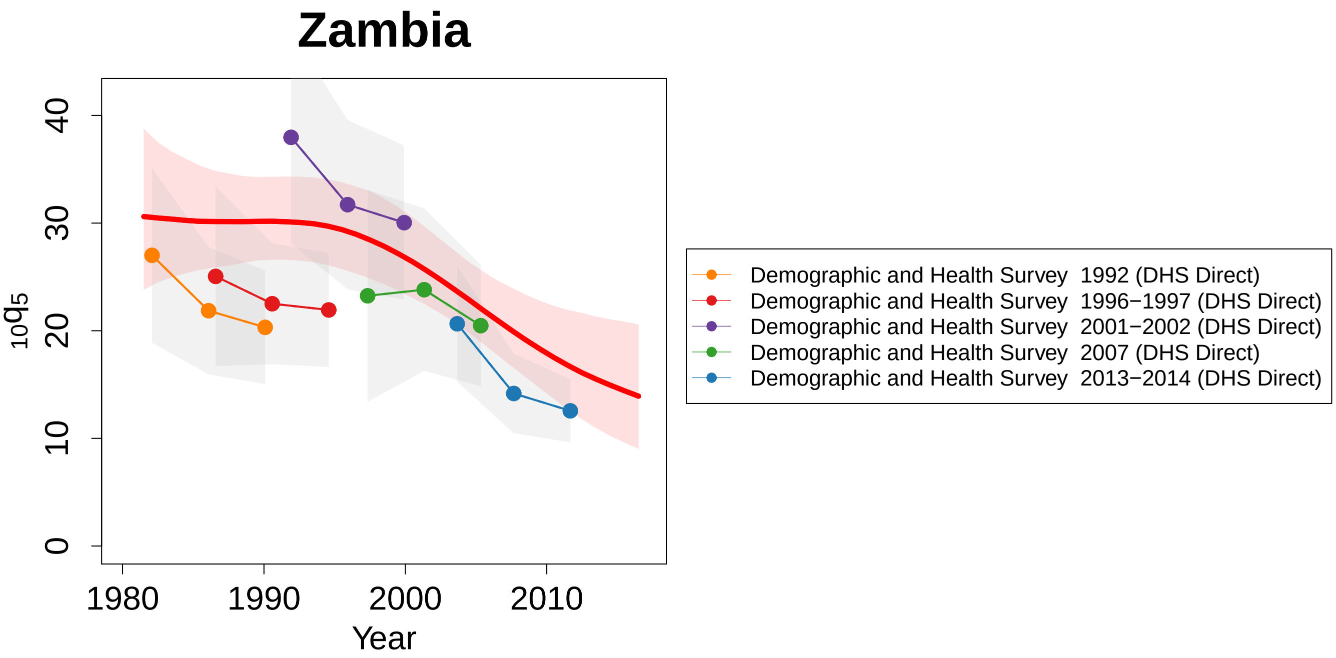
Zambia
0
10
20
30
40
$_{10}q_5$
1980
1990
2000
2010
Year
Demographic and Health Survey 1992 (DHS Direct)
Demographic and Health Survey 1996−1997 (DHS Direct)
Demographic and Health Survey 2001−2002 (DHS Direct)
Demographic and Health Survey 2007 (DHS Direct)
Demographic and Health Survey 2013−2014 (DHS Direct)

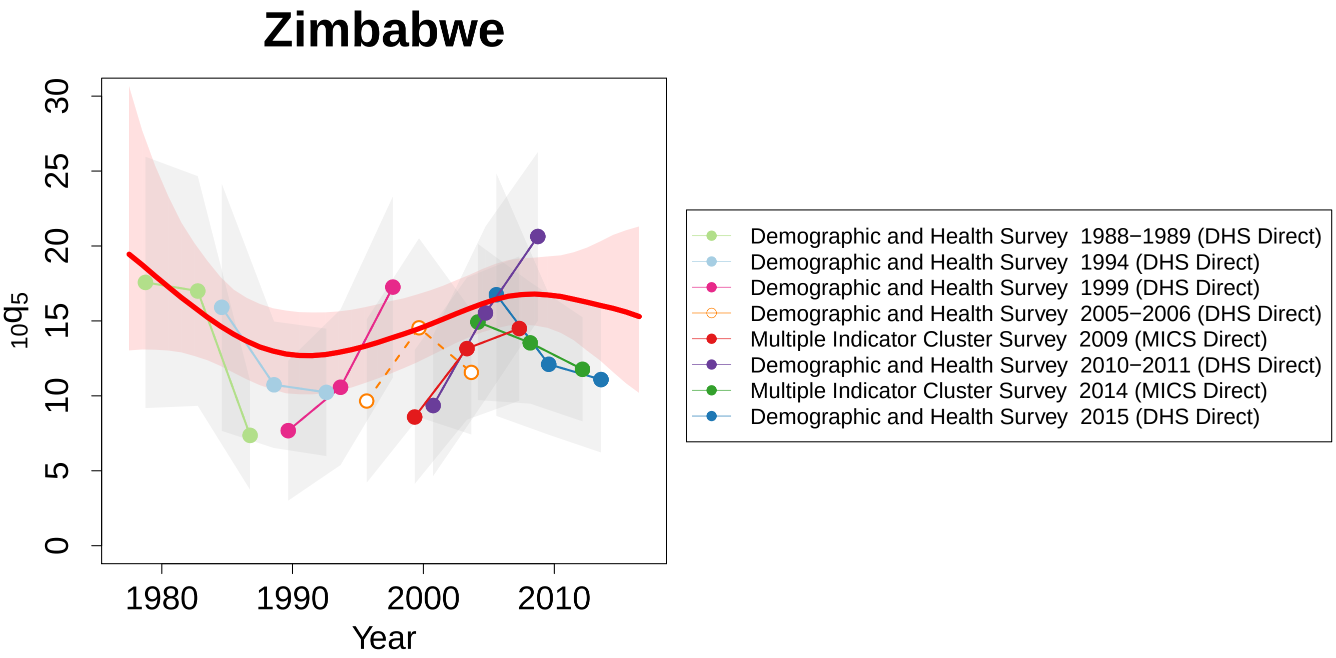
Zimbabwe
0
5
10
15
20
25
30
$_{10}q_5$
1980
1990
2000
2010
Year
Demographic and Health Survey 1988−1989 (DHS Direct)
Demographic and Health Survey 1994 (DHS Direct)
Demographic and Health Survey 1999 (DHS Direct)
Demographic and Health Survey 2005−2006 (DHS Direct)
Multiple Indicator Cluster Survey 2009 (MICS Direct)
Demographic and Health Survey 2010−2011 (DHS Direct)
Multiple Indicator Cluster Survey 2014 (MICS Direct)
Demographic and Health Survey 2015 (DHS Direct)

References

[1] United Nations, *World Population Prospects: The 2017 Revision.* UN, 2017.

[2] J. Banister and K. Hill, "Mortality in China 1964-2000," *Popul Stud (Camb)*, vol. 58, no. 1, pp. 55–75, 2004.

[3] R. Dorrington, "The generalized growth balance method," in *Tools for demographic estimation* (T. . Moultrie, R. Dorrington, A. Hill, K. Hill, I. Timæus, and B. Zaba, eds.), Paris: International Union for the Scientific Study of Population, 2013.

[4] L. Alkema and J. R. New, "Global estimation of child mortality using a bayesian b-spline bias-reduction model," *The Annals of Applied Statistics*, vol. 8, no. 4, pp. 2122–2149, 2014.

[5] P. H. C. Eilers and B. D. Marx, "Flexible smoothing with b -splines and penalties," *Statist. Sci.*, vol. 11, pp. 89–121, 05 1996.

[6] P. H. C. Eilers and B. D. Marx, "Splines, knots, and penalties," *Wiley Interdisciplinary Reviews: Computational Statistics*, vol. 2, no. 6, pp. 637–653, 2010.

[7] I. D. Currie and M. Durban, "Flexible smoothing with p-splines: a unified approach," *Statistical Modelling*, vol. 2, no. 4, pp. 333–349, 2002.

[8] A. Coale, P. Demeny, and B. Vaughn, *Regional model life tables and stable populations (2nd ed.).* New York: Academic Press, 1983.

[9] K. Hill, L. Zimmerman, and D. T. Jamison, "Mortality risks in children aged 5-14 years in low-income and middle-income countries: a systematic empirical analysis," *The Lancet Global Health*, vol. 3, no. 10, pp. e609–e616, 2015.
